# Person-centered practice in the Portuguese healthcare system: A documentary study

**DOI:** 10.1371/journal.pone.0343419

**Published:** 2026-03-03

**Authors:** Diana Vareta, Elaine Santana, Célia Oliveira, Cristina Baixinho, Filipa Ventura

**Affiliations:** 1 PhD Program, University of Lisbon (UL) and Nursing School of Lisbon (ESEL), Lisbon, Portugal; 2 Egas Moniz Interdisciplinary Research Centrse (CiiEM), Egas Moniz Universitary Institute, Caparica, Almada, Portugal; 3 The Health Sciences Research Unit: Nursing (UICISA:E), Nursing School of Coimbra (ESEnfC), Coimbra, Portugal; 4 Nursing School of Lisbon (ESEL), Lisbon, Portugal; 5 Nursing Research, Innovation and Development Centre of Lisbon (CIDNUR), Lisbon, Portugal; 6 Center for Innovative Care and Health Technology, ciTechCare, Leiria, Portugal; Faculty of Health Sciences - Universidade da Beira Interior, PORTUGAL

## Abstract

**Background:**

Person-centered practice has increasingly become a key structural component of health systems, driving the progressive reformulation of public health policies internationally. Despite broad consensus regarding its importance, its integration into clinical practice continues to face significant challenges. The Person-Centred Practice Framework identifies the macro context as a critical domain for sustainable implementation, as it encompasses the structural and strategic factors that shape healthcare delivery.

**Objective:**

To characterize the person-centered practice framing within the macro context of the Portuguese healthcare system.

**Methodology:**

Qualitative, descriptive, and retrospective documentary study. A systematic literature search was conducted on the websites of entities responsible for defining, guiding, and regulating healthcare in Portugal. Using predefined eligibility criteria, 40 documents were selected. Textual analysis was performed using IRAMUTEQ® software and guided by the constructs of the macro context domain of the Person-Centred Practice Framework.

**Results:**

The lexicometric analysis identified five classes, grouped into two thematic fields: i) *Structural and organizational determinants of person-centered practice*, comprising the classes *Systemic vision and integrated response*, *Organizational culture and participation*, *Digital transformation and information management*, and *Political vision and governance structures*; and ii) *Operationalization of person-centered practice*, represented solely by the class *Care approach*, reflecting its thematic specificity. Factorial analysis revealed distinct and poorly connected discursive patterns associated with different action levels within the healthcare system. Similarity analysis highlighted a discourse centered on the health-care-person nucleus, showing relations with service organization, care integration, and person participation, albeit with word dispersion suggesting misalignment between organizational and practice levels.

**Conclusion:**

The macrocontextual framing of person-centered practice in the Portuguese healthcare system demonstrates significant political and strategic advances, as seen in the emphasis on humanized care, investment in health literacy and digitalization, and the introduction of Integrated Care Pathways. However, implementation remains limited due to the absence of clear formative guidelines, biomedical paradigm persistence, and lack of evaluation mechanisms sensitive to care experience.

## 1 Introduction

In light of emerging health challenges, such as the rising prevalence of chronic diseases, population aging, increasing demand for healthcare services, persistent fragmentation of care that challenges patient navigation, and the associated economic burden, the development of person-centered health systems is essential to ensure more effective, personalized, and sustainable responses [1,2]. Person-centered care, defined by the World Health Organization (WHO) [3] as “an approach to care that consciously adopts individuals’, carers’, families’, and communities’ perspectives as participants in and beneficiaries of trusted health systems that respond to their needs and preferences in humane and holistic ways” (p. 5), has increasingly been established as a foundational pillar of health systems, inspiring the progressive transformation of health policies internationally [3–5]. Despite widespread acknowledgment of its importance, substantial challenges to the implementation and further development of person-centered care persist, limiting its effective integration into routine care practices [1,6–8].

According to the WHO [3], each country should develop a strategy for the implementation and advancement of person-centered care that is tailored to the specific characteristics of its health system, care context, and healthcare workforce. The health system encompasses all services involved in the promotion, maintenance, and restoration of health for both individuals and populations [2]. The Person-Centred Practice Framework (PCPF), developed by McCormack et al. [9], offers a robust theoretical model that facilitates the translation of person-centered care principles into clinical practice. The framework comprises five interrelated domains: macro context, prerequisites, the practice environment, person-centered processes, and outcomes. These domains interact dynamically and correspond to different levels of intervention, namely structural, organizational, and individual [9,10]. The PCPF is grounded in the premise that the macro context exerts a direct influence on both organizational and individual domains. When structural elements are aligned with the principles of Person-Centred Practice (PCP), they foster enabling conditions for the development of person-centered organizational cultures, thereby supporting the flourishing of all those involved, which represents the intended outcome of the framework [9].

The PCPF identifies the macro context as a critical starting point for the sustainable implementation of PCP. This domain encompasses sociopolitical factors that shape the legal, ethical, and operational frameworks of healthcare systems and directly influence strategic priorities. The macro context comprises four key constructs: strategic leadership, policy frameworks, strategic frameworks, and workforce developments [9].

Describing the extent to which health systems are aligned with PCP contributes for informing the definition of targeted strategies for its development and consolidation [1,2]. In this context, the present study aims to characterize how PCP is framed within the macro context of the Portuguese healthcare system.

## 2 Methodology

### 2.1 Study design

This research is part of a clinical study protocol [11] designed to provide recommendations for enhancing PCP in the daily care of hospitalized older adults with chronic illness in an internal medicine unit. The present study contributes to this protocol by specifically characterizing the Portuguese healthcare system. To this end, a qualitative, descriptive, and retrospective documentary approach was adopted, defined as a systematic procedure for identifying, selecting, and analyzing documents relevant to the topic under study [12]. This approach focuses on the examination of policy and strategic documents to explore how a phenomenon is framed within a specific context, rather than on synthesizing empirical research evidence as in a traditional literature review [12].

### 2.2 Search strategy

A preliminary exploratory search was conducted to identify the terminology commonly used in Portugal to refer to the concept under study, thereby ensuring terminological consistency and sensitivity. Based on this search, the following keywords were defined: citizen-centeredness, person-centeredness, citizen-centered, patient-centered, service user-centered, person-centered, family-centered, integrated care, holistic care, centered care, focus on the patient, focus on the service user, focus on the citizen, focus on the person, shared decision-making, and citizen engagement. Subsequently, the Population–Concept–Context (PCC) framework was applied to guide the search strategy and eligibility criteria. The population comprised healthcare professionals and users of healthcare services, the core concept was PCP, and the context corresponded to healthcare delivery in Portugal. Based on this framework, inclusion and exclusion criteria were defined (S1 Appendix) to ensure the relevance and appropriateness of the selected documents in addressing the research question.

Documentary sources were identified from entities responsible for the definition, regulation, governance, and strategic guidance of healthcare in Portugal, including the Diário da República (official journal of the Portuguese Republic), the National Health Service (SNS), the Directorate-General of Health (DGS), professional regulatory bodies (Medical, Nursing, Psychology, Physiotherapy, and Nutrition), the Health Regulatory Authority, the National Health Council, the Portuguese Health System Observatory, the Calouste Gulbenkian Foundation, and the Regional Health Administrations. Grey literature was also identified through targeted searches using Google®.

A temporal criterion was applied to include documents published between 2012 and 2024, as 2012 marks the formal introduction of PCP into national health policy [13]. Including documents up to 2024 allowed for an examination of the evolution of PCP over more than a decade and ensured that all relevant strategies and recommendations are considered.

The predefined keywords were applied to the websites of the afore mentioned institutions, and document and reference management were conducted using Mendeley Desktop® (v. 2.132.2, Elsevier, released 2020, London, United Kingdom). To ensure linguistic consistency of the text corpus, documents published in languages other than Portuguese were excluded.

All retrieved documents were independently screened by two researchers for relevance based on the established eligibility criteria. Discrepancies were discussed and resolved through consensus, and when necessary, a third researcher was consulted.

### 2.3 Data analysis

Data analysis was carried out using statistical analysis of the textual corpus through the IRAMUTEQ® software (Interface de R pour les Analyses Multidimensionnelles de Textes et de Questionnaires) (v. 0.8 alpha 7, LERASS Laboratory. Released 2009, Toulouse, France). This software enables several types of textual data analysis via R and Python, including basic lexicography (word frequency analysis), lemmatization (reducing words to their base form considering the meaning and grammatical context), and multivariate analysis, which includes descending hierarchical classification (DHC) (iterative Reinert method that partitions elementary context units into lexical classes based on shared vocabularies, retaining forms associated with each class by χ²), correspondence factorial analysis (CFA) (projects the lexical classes obtained with DHC and the active forms onto a factorial plane to visualize semantic proximities and the contribution of supplementary variables), and similarity analysis (graph-based mapping of word co-occurrences to reveal central nuclei and peripheral branches in the text’s structure) [14].In DHC, IRAMUTEQ® uses the chi-square (χ²) test to create a word dictionary, indicating the strength of association between words and their respective classes. This association is considered statistically significant when the χ² value exceeds 3.84, corresponding to *p* < .0001 [15]. For lexical analysis, the software segments the text into 40-character units, referred to as text segments (TS), which form the basis for classification. These segments are grouped into classes based on vocabulary similarity and distinction from other classes [14,16]. Each class is named through an exhaustive reading and interpretation of its most representative words [17]. Interpretation requires theoretical reflection and contextual understanding by the researchers, retrieving the original TS from the corpus to determine meaning in context [17].

The CFA enables the visualization of the semantic distance between content words (in their reduced form) within the factorial plane based on lexical classes and their corresponding TS [14,16]. Similarity analysis identifies the co-occurrence of lexical forms within the corpus to generate graphical representations of the central discursive nuclei, revealing the structure of meaning networks and lexical articulations among central concepts [14,16].

The textual corpus was built from selected excerpts without losing the context of the concept under study. It was then subjected to in-depth reading to extract relevant content for lexical analysis, following the guidelines of Bowen [18]. Preparation followed the procedures recommended by Camargo and Justo [14], including standardization of acronyms, clitic verb forms, numerical formatting, and removal of special characters. The text was also adjusted according to the latest Portuguese orthographic agreement.

For each document, variables of interest, such as document type, year, author, and thematic area, were identified to support multivariate analysis.

A pilot test was conducted with 13 documents to assess the data's compatibility with IRAMUTEQ® functionalities, explore the software's potential to address the study questions, test different analysis methods, correct coding errors, and familiarize the research team with its operation.

For the final corpus analysis, the criteria for including elements in each class were frequency above twice the average word occurrence and a class association based on a χ² value of ≥ 3.84 [15], corresponding to one degree of freedom and a 95% significance level.

### 2.4 Ethical considerations

The principles of scientific integrity were upheld, ensuring the rigorous, transparent, and ethical use of all sources, with proper citation and respect for intellectual property. Data collection was conducted exclusively through documents publicly available on open-access websites. Therefore, no ethics approval was required.

## 3 Results

**Fig 1:**
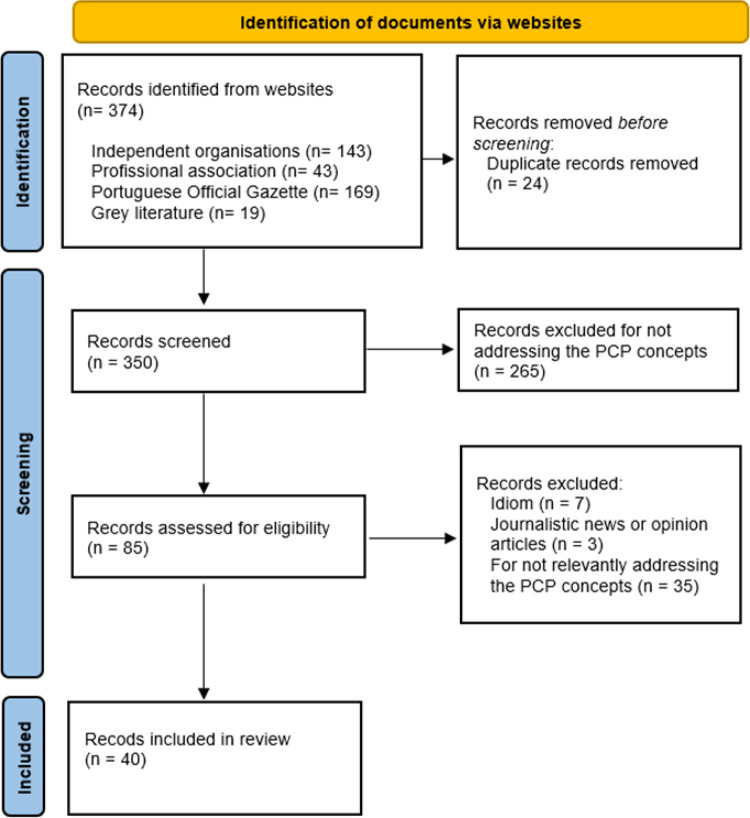
Document selection process [19].

Of the 374 documents identified, 40 met the predefined inclusion criteria (Fig 1). The types of documents included in the analysis were institutional reports (n = 12), legislative documents (n = 9), clinical guidelines (n = 6), national plans or strategies (n = 12), and explanatory documents (n = 1).

Regarding authorship, the documents were produced by governmental entities (n = 30), independent organizations (n = 7), and professional regulatory bodies (n = 3). The analysis of the documents’ nature revealed a predominance of strategic content focused on public policies (n = 25), followed by normative content in the form of technical guidelines and recommendations (n = 15). It is worth noting that no documents were identified that addressed the educational or research dimensions.

The temporal distribution of the analyzed documents shows a progressive increase over the period from 2013 to 2024, with a higher concentration in more recent years: 2013 (n = 3), 2014 (n = 4), 2015 (n = 1), 2016 (n = 2), 2017 (n = 2), 2018 (n = 3), 2019 (n = 3), 2020 (n = 4), 2021 (n = 2), 2022 (n = 5), 2023 (n = 6), and 2024 (n = 5).

The textual corpus was processed using IRAMUTEQ® in 1 minute and 5 seconds, identifying 33.377 occurrences, 3.665 distinct words, and 1.057 active forms with a frequency higher than 3.

It was found that the segments not incorporated into the analysis corresponded to repeated text segments TS across different documents that did not contribute to the dataset's lexical variability. Therefore, their exclusion helped to highlight the corpus's discursive diversity.

### 3.1 Descending hierarchical classification

In the DHC, 920 TS were analyzed, with 853 classified, representing a retention rate of 92.72%. According to the software manual [14], the retention is the percentage of TS assigned to lexical classes, with values exceeding 75% being recommended. Our result indicates a few unclassified TS and coherent, stable classes suitable for interpretation.

**Fig 2:**
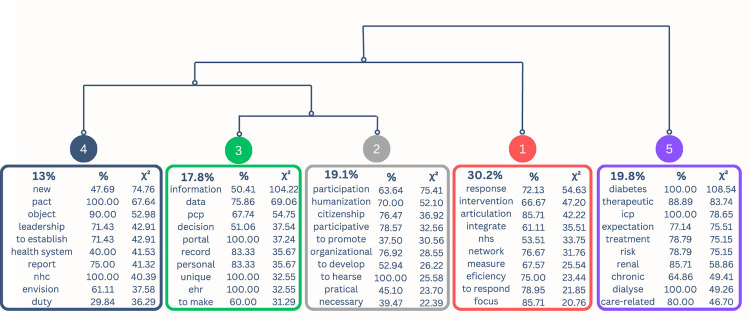
DHC dendrogram of PCP in the macro context of the Portuguese healthcare system. NHC – National health council; PCP – Personalized care plan; EHR – Electronic health record; NHS – National health system; ICP – Integrated care pathway.

The dendrogram identifies five thematic classes and illustrates their discursive differences through the most characteristic and specific linguistic forms in each class (Fig 2). The analyzed content was categorized into two overarching thematic fields: Structural and organizational determinants of PCP and Operationalization of PCP, which were further subdivided into smaller thematic units. The names assigned to the thematic fields reflect TS's meanings within the corpus.

The thematic field *Structural and organizational determinants of PCP* is subdivided into two classes, with the first branch corresponding to Class 1 (Systemic vision and integrated response), composed of 258 TS (30.2%). This class is centrally positioned, indicating a strong influence or connection with the other classes. The second branch corresponds to class 2, 3 and 4. Class 2 (Organizational culture and participation), composed of 163 TS (19.1%), Class 3 (Digital transformation and information management), with 152 TS (17.8%), and Class 4 (Political vision and governance structures), with 111 TS (13%), derive from the same dendrogram branch and form an interconnected subgroup, suggesting shared themes or common vocabulary among them.

The thematic field *Operationalization of PCP* is composed solely of Class 5 (Care approach), which includes 169 TS (19.8%). This class is positioned on an isolated branch, distant from the others, indicating that its lexical content is distinct.

Considering the size of the classes and aiming to preserve the richness of the corpus in relation to the study topic, the analysis will not be limited to the most strongly associated words in each class. Other statistically significant words (S1 Table) will also be considered, as their presence in the analyzed discourse contributes to complementing, deepening, or contrasting the core meaning of each class.

#### 3.1.1 Class 1 – Systemic vision and integrated response.

Class 1, the most predominant in the corpus (30.2%), highlights a systemic approach to the organization of healthcare, with key concepts such as *response* (χ² = 54.63), *intervention* (χ² = 47.20), and *coordination* (χ² = 42.22). The themes address the complexity of current health and social issues, the need for intersectoral coordination, and the emphasis on principles such as equity, universality, and co-production of care.

The identification of social issues, such as *aging* (χ² = 19.44) and *homelessness* (χ² = 8.04), underscores the need to *integrate* (χ² = 35.51) the *social* sector (χ² = 15.46) into health responses and helps identify populations in situations of greater vulnerability. To address this, the healthcare system must operate in an interconnected way, as reflected in terms like *coordination* (χ² = 42.22), *integration* (χ² = 35.51), and *network* (χ² = 31.76). This approach is illustrated by expressions such as *“multidisciplinary and intersectoral intervention aims to respond to new challenges”* and *“its main objectives include the continuous and integrated provision of healthcare and social support”*.

The strategic vision is grounded in shared values and principles, such as *equity* (χ² = 20.76), *equality* (χ² = 11.60), and *proximity* (χ² = 13.54), and promotes collaboration among organizations, professionals, service users, and their representatives in a logic of shared responsibility, conveyed through terms such as *coordination* (χ² = 8.91), *co-production* (χ² = 6.94), and *co-responsibility* (χ² = 6.94).

Beyond healthcare organization, Class 1 includes several terms focused on the value of outcomes, both at the organizational and individual levels, such as *efficiency* (χ² = 23.44), *well-being* (χ² = 19.32), and *gain* (χ² = 9.74). These elements reinforce the idea that an integrated health system response should ultimately aim to generate value in health.

#### 3.1.2 Class 2 – Organizational culture and participation.

The graphical proximity between Classes 2 and 3 (Digital Transformation and Information Management) reflects a shared perspective on the organizational conditions necessary for PCP, particularly regarding organizational culture and digital transformation.

Within this relationship highlighted by the DHC, Class 2 presents themes that emphasize the person’s value as an active participant in care interactions. Notable keywords include *participation* (χ² = 75.41), *citizenship* (χ² = 36.92), and *participatory* (χ² = 32.56). Citizenship is framed as a strategic objective, expressed in proposals such as *“promoting a culture of citizenship”* and *“empowering individuals for informed citizenship”.*

A discourse aimed at the development of organizational culture is evident, reflected in the frequency of terms like *organizational* (χ² = 28.55), *develop* (χ² = 26.22), and *culture* (χ² = 18.35), supported by structured and intentional actions for transformation. This is illustrated by terms such as *initiative* (χ² = 15.37), *national health plan* (χ² = 7.51), and *SNS Proximity* (χ² = 5.46). There are also signs of a collective and broad-based movement, with references to territorial levels such as *local* (χ² = 18.20), *regional* (χ² = 13.31), and *Portugal* (χ² = 8.79).

Class 2 also includes themes aimed at healthcare professionals relating to the continuous personal and professional development needed to drive change. This is reflected in terms such as *competence* (χ² = 22.15), *reflection* (χ² = 20.26), and *communication* (χ² = 13.98). Additionally, it highlights the need to consolidate shared values among those involved in care interactions, evidenced by terms like *humanization* (χ² = 52.10) and *respect* (χ² = 12.64). The focus on PCP is illustrated by proposals such as: *“promote training for healthcare professionals aimed at enhancing competencies that support person-centeredness”* and *“encourage reflection on the centrality of healthcare professionals in change processes”.*

The evaluation of processes and outcomes is identified as an essential component for monitoring organizational change, as shown by the presence of terms like *evaluation* (χ² = 17.70), *instrument* (χ² = 11.41), and *monitoring* (χ² = 11.35).

#### 3.1.3 Class 3 – Digital transformation and information management.

Class 3 addresses themes related to digital transformation to empower individuals in the informed management of their health. In alignment with the participatory culture discourse in Class 2 (Organizational Culture and Participation), this class demonstrates thematic proximity through a shared branch in the hierarchical structure.

There is a strong presence of terms such as *information* (χ² = 104.22), *data* (χ² = 69.06), and *personalized care plan* (χ² = 54.75), highlighting a focus on digitalization as a tool that enables access to health knowledge.

Information management mechanisms are characterized by their ability to integrate data, as shown by terms such as *electronic health record* (χ² = 32.55) and *integrative* (χ² = 18.53), as well as *centralization* (χ² = 18.53) of information throughout an individual’s journey in the health system. This perspective is illustrated by excerpts such as: *“high priority should be given to the full implementation of an electronic health record as an essential foundation for integrated service provision”* and *“the centralization of clinical and social health information is essential to support care processes and decision-making”.*

In this context, individuals assume an active role in the care process, evidenced by terms such as *user* (χ² = 37.54), *protagonist* (χ² = 37.54), and *citizen* (χ² = 11.72). Digital transformation emerges as a facilitator of informed participation in health decisions, reflected in terms such as *decision* (χ² = 37.54) and *inform* (χ² = 18.03), by ensuring access to clinical records, supporting information management, and promoting health literacy. This is evidenced in statements such as: *“strengthen health literacy by providing citizens with information, knowledge, and skills to make informed decisions”* and *“to promote health literacy, the citizen area of the SNS portal already provides access to the health literacy library”.*

#### 3.1.4 Class 4 – Political vision and governance structures.

The discourse identified in Class 4 refers to a macro-level perspective grounded in the decision-making structures responsible for shaping health governance policies and strategies.

The terms *new* (χ² = 74.76) and *pact* (χ² = 67.64) highlight a discourse on transforming the health system. This transformation is portrayed as a shared process between institutions and citizens, as indicated by the *health system* (χ² = 41.53), *Ministry of Health* (χ² = 30.95), *society* (χ² = 34.48), and *individual* (χ² = 26.93), and is expected to occur in an integrated manner involving all system levels. This vision is reflected in excerpts such as: *“Leadership is described as a transversal process, requiring an active and coordinated presence at all levels of the health system (…) ensuring intersectoral coordination and continuity of governance structures that already promote safety and quality in healthcare.”*

The proposed change is based on a shift from perceiving the person as an *object* (χ² = 52.98) to viewing them as the *target* (χ² = 32.96) of care. This transformation is illustrated by statements such as: *“the shift from a system based on hospitals and health professionals to one based on the community and people”* and *“a future-ready health system must be people-centered and capable of delivering integrated healthcare to all individuals, which entails major changes”.*

The discourse also includes concepts related to formal governance instruments, such as *accountability* (χ² = 19.94) and *deliberation* (χ² = 13.64), as well as the roles of various stakeholders in the system, highlighted by terms like *leadership* (χ² = 42.91) and *represent* (χ² = 26.40). These elements contribute to defining responsibilities, functions, and participation channels in designing and implementing public policies.

The placement of Class 4 within the same branch as Classes 2 (Organizational Culture and Participation) and 3 (Digital Transformation and Information Management) suggests that implementing these dimensions depends on political and governance strategies aligned with PCP principles. Thus, the political vision guiding the health system must align with the organizational vision, creating structural, cultural, and operational conditions that enable PCP across all levels of practice. However, it is worth noting that Class 1 (Systemic Vision and Integrated Response) appears in an opposite branch to Class 4, revealing a structural distance between the political-strategic discourse and operational discourse. This separation suggests that despite strategic orientations promoting PCP transformation, significant challenges remain in translating these intentions into effective practices.

#### 3.1.5 Class 5 – Care approach.

The discourse identified in Class 5 reveals a tension between different approaches to healthcare delivery. On the one hand, there is a strong presence of biomedical language, reflected in terms such as *diabetes* (χ² = 108.54), *therapeutic* (χ² = 83.74), and *treatment* (χ² = 75.15). On the other hand, although less prominent, the discourse also values the human and holistic dimensions of care, as expressed through words such as *spiritual* (χ² = 16.27), *psychological* (χ² = 15.35), and *humanized* (χ² = 7.70).

The terms used to designate the care recipient vary between *patient* (χ² = 35.99), *adult* (χ² = 30.91), and *person* (χ² = 16.75), reflecting different perspectives on those receiving care, ranging from clinically oriented to more person-focused approaches.

The concept of the *integrated care process* (χ² = 78.65) is strongly associated with this class, emphasizing the importance of a coordinated and articulated approach across different levels of care.

Class 5 also highlights themes related to care delivery settings, illustrated by terms such as *domiciliary* (χ² = 30.93), *hospitalization* (χ² = 20.36), and *clinic* (χ² = 20.31), reflecting the diversity of environments in which care takes place. Key actors in the care process include *family* (χ² = 37.07), *team* (χ² = 26.56), and *multidisciplinary* (χ² = 29.39), reinforcing the collaborative and interprofessional nature of care, as well as the involvement of informal support networks in addressing the person’s needs.

Regarding the healthcare workforce, Class 5 underscores the importance of multidisciplinary teamwork to optimize the person’s journey through the health system, as shown in the excerpt: *“requires the action of multidisciplinary health professional teams, demanding appropriate communication and cooperation to avoid fragmented and uncoordinated activities”.* The presence of terms linked to relational aspects, such as *adjust* (χ² = 32.69) and *guide* (χ² = 30.91), as well as responsiveness to individual needs, such as *expectations* (χ² = 75.51) and *needs* (χ² = 13.69), emphasizes care as a dynamic process tailored to the person’s uniqueness.

Additionally, Class 5 reveals a set of desired outcomes, including *cost-effective* (χ² = 15.35), *satisfaction* (χ² = 8.18), and *safe* (χ² = 7.31), reflecting concerns with the impact of care and valuing feedback from those involved.

The isolated positioning of Class 5 in the dendrogram illustrates its thematic autonomy, grounded in a micro-level care delivery perspective. In contrast, the other classes adopt a macro perspective, such as the systemic vision of healthcare provision (Class 1), public policies (Class 4), and organizational transformation (Classes 2 and 3).

It is also important to note that the terminological variability in the corpus regarding how individuals interact with the health system is referred to (Table 1). Various designations associated with the concept of PCP emerged, reflecting different conceptions of the individual in the healthcare context concerning the system and the underlying care philosophy. Notably, Class 2 did not contain any statistically significant designation.

**Table 1:** Terminology used to refer to individuals interacting with the healthcare system.

	*X* ^*2*^	Frequency	Class
**Service user**	5.03	42.42%	1
**Patient**	4	20.55%	4
	35.99	46.58%	5
**Person**	16.75	28.29%	5
**Individual**	26.93	56.25%	4
**Citizen**	5.95	18.60%	4
	11.72	26.74%	3
**User**	18.53	100%	3

### 3.2 Post-factorial correspondence analysis

The analysis of the semantic structure of the classes revealed three distinct and poorly connected discursive patterns, with thematic groupings that reflect different levels of action within the healthcare system. This fragmentation is identified in the factorial plane (S3 Appendix), which explains 59.29% of the total variance (31.52% on Factor 1 and 27.77% on Factor 2), and highlights the separation between different semantic fields. When the variables of interest are considered (S4 Appendix), differentiated patterns in document production are also observed, reinforcing thematic segmentation.The absence of a common vocabulary core suggests that the classes exhibit specific lexicons with low lexical integration.

In the upper and lower left quadrants, the classes related to direct care delivery (Classes 1 and 5) occupy distinct positions within the factorial space. Class 5 (purple), associated with biomedical care and technical standards, appears isolated, reflecting a specialized approach that is less integrated at the clinical practice level. It includes documents classified as guidelines (doc_4), published in 2013 and 2016, and related to practice guidance (area_1), such as the Integrated Care Pathways, which display different semantic features compared to more recent documents. In contrast, Class 1 (red) reflects a systemic perspective focused on an articulated response across sectors. This division suggests the coexistence of two discourses, one technical-clinical and one organizational, with limited lexical articulation.

Classes 2 (grey), 3 (green), and 4 (blue), grouped in the upper right quadrant, reflect an integrated vision that connects organizational culture, digital transformation, and strategic policy. The proximity between the marker's participation and information reinforces the interdependence between user involvement and access to knowledge. Noteworthy are reports (doc_2) produced by independent entities (author_3), such as A Future for Health (id_49) [20]. Lastly, the lower quadrants highlight the isolated position of very recent productions (2018–2024), predominantly authored by governmental bodies (author_2), which remain only marginally integrated into the broader discourse, reflecting emerging trends in political and operational innovation (area_3).

The correspondence analysis results reveal a progressive evolution in institutional discourse: from an initial phase focused on standardized clinical norms and practices to a more recent approach emphasizing systemic transformation, user engagement, and technological innovation. This discursive shift appears to reflect the growing recognition of PCP as a key pillar of health policy in Portugal.

### 3.3 Similarity analysis

The similarity analysis presents the results in the form of a graph (S5 Appendix), where words represent the vertices and the edges indicate both the presence and the strength of the relationship between semantic fields: the thicker the line, the stronger the connection between terms [14,16].

The core themes of the corpus are at the centre of the graph, with the word *health* showing the highest prominence and centrality. Surrounding it are terms such as *access, promotion, citizen, information, health system, service, NHS, process, integrate, user,* and *management*, revealing a strong orientation of the discourse toward the organization of the health system and the assurance of accessibility, service integration, and citizen involvement in care processes, articulating entities, processes, and recipients.

This central structure connects directly to a robust branch leading to the semantic field of *care*, closely associated with concepts such as *need*, *integration*, *delivery*, *continuity*, and *response*. The terms *care plan* and *integrated care process* suggest the presence of planning instruments and strategic guidance. These terms reflect concern with the coordination and alignment across services and levels of care, expressing a health vision centered on persons concrete needs.

From the *care* core, a second branch emerges and converges around the word *person*, forming a new semantic pole where terms such as *health professional*, *caregiver*, *family* *member*, *centered*, and *intervention* prevail. This cluster highlights the importance of involving professionals and informal support networks in the PCP delivery.

The distance observed between the *health-care* and *care-person* clusters suggests a potential misalignment between the system’s organizational logic and the person-centered clinical practice. Additionally, the isolation of the words *scientific* and *evidence*, positioned peripherally and opposite to *health professionals*, may indicate that scientific knowledge, though present, remains marginal and poorly integrated into the discourse on direct care delivery.

Words such as *humanization*, *relationship*, *centrality*, and *decision* appear scattered around *health*, possibly reflecting a discursive intention to value humanistic principles, even if they are not fully integrated into clinical practice.

## 4 Discussion

The research question will guide the discussion of the results, exploring the constructs that, according to the PCPF, shape the macro context of PCP: strategic leadership, policy frameworks, strategic frameworks, and workforce developments [9].

### 4.1 Cross-sectional analysis of the document corpus

The corpus analysis revealed relevant cross-cutting aspects that characterise how PCP is framed within the Portuguese health system. Only 11% of the initially identified documents met the inclusion criteria, highlighting a scarcity in national production on PCP. Nevertheless, the high retention rate (92.72%) and the substantial number of active forms (1.057) provide statistical robustness and reliability for the lexicometric analysis [14,21].

There was a predominance of strategic documents (62.5%) produced mainly by governmental entities (75%). This suggests that PCP has been primarily framed at the public policy level. While this indicates political commitment, it raises concerns regarding translating these strategies into concrete practices within care settings. The absence of education-related documents is particularly notable, reflecting the lack of explicit guidelines for integrating PCP into academic curricula and ongoing professional development. This gap undermines professional capacity-building and poses a barrier to the cultural shift required to implement PCP successfully [7,22,23].

The chronological distribution of documents shows a trend between 2013 and 2024, with a greater concentration in recent years (2022–2024), indicating a growing political commitment to PCP.

Lexical analysis revealed a fragmented and ambiguous use of the PCP concept within individual documents and across publications from the same institution. This inconsistency, coupled with the absence of a shared definition, compromises discursive coherence and hinders the development of a shared vision among professionals, managers, and service users [7,24,25].

The DHC identified five thematic classes organized around two main dimensions that capture different levels of the health system and reveal the degree of integration in the operationalization of PCP. The systemic dimension (Class 1) reflects a collaborative vision of the health system, conceived as an interconnected and coordinated network of sectors, institutions, and professionals aiming to deliver integrated care. The political dimension (Class 4) reveals the involvement of various stakeholders in shaping public health policies and highlights the momentum for reform. It is complemented by organizational culture (Class 2) and digital transformation (Class 3), which represent essential prerequisites for implementing change. The practical dimension (Class 5) focuses on care delivery and illustrates tensions between biomedical and humanistic approaches to care.

This analysis highlights a gap between strategic discourse and clinical practice, underscoring the need to strengthen integration mechanisms between macro- and micro-levels. Such articulation is essential to sustaining meaningful cultural change, supported by strategic leadership, participatory public policy, clear operational frameworks, and investment in workforce development. The following sections discuss these dimensions based on the discursive evidence drawn from the analysis.

### 4.2 Strategic leadership

Class 2 revealed a discourse oriented toward the development of organizational culture, highlighting a set of shared values essential to PCP, namely, humanization, participation, and citizenship, and identifying strategies to support the empowerment of healthcare professionals. Complementarily, Class 3 emphasized digital transformation as a strategic resource not only for empowering professionals but also for equipping health service users.

The emphasis placed on organizational culture is particularly relevant, considering its potential to act as either a facilitator or a barrier to the effective implementation of PCP [26,27]. Organizational culture refers to shared values, beliefs, norms, and practices that shape team members’ behaviors in a given context. In healthcare systems, it profoundly influences how care is delivered, the quality of interpersonal relationships, and the decision-making structures [28,29].

Humanization of care is defined as the appreciation of the human being and respect for their dignity, manifesting as “an attitude that values individuality (...) beyond care and treatment” [30] (p. 1). Investment in this area is evidenced by the creation of the Commitment to Hospital Humanization in 2019 [30], the National Commission for the Humanization of Healthcare in the NHS [31], and its integration into recent legislative frameworks, namely, base 20 of the Basis Health Law [32]. This reflects the formal recognition of the importance of humanized care and the need to address the challenges associated with its practical implementation in everyday healthcare services.

### 4.3 Health and social policies

The involvement of individuals and various stakeholders in defining, monitoring, and evaluating health policy decisions is presented as a valued dimension, though it is still in the consolidation process. The active participation of individuals in the planning and delivery of care is regarded as one of the foundational pillars of exercising the right to health citizenship [33]. By emphasizing the need to empower citizens to exercise their rights in an informed manner, the discourse reveals a commitment to building a health system rooted in informed and responsible participation. In this regard, the creation of the National Health Council [34] emerges as an independent entity representing citizens, while the Charter for Public Participation in Health [35] serves as a guiding instrument, outlining the participatory process for individuals, whether living with illness or not, and their representatives in shaping health policy. Participation is positioned as a democratic principle that fosters partnership in decision-making processes and priority setting across sectors, ensuring participatory governance. However, while strategic frameworks promote participation, it is essential to identify structural barriers that hinder its effective exercise [36,37].

Digital transformation is referenced as a tool for promoting citizens’ active participation in informed health decisions through access to health records, support for information management, and enhanced health literacy. The existence of national strategies for health literacy in Portugal is understood as a top-down initiative with the potential to drive the development of citizen empowerment programs. However, their effectiveness depends on implementation across all health system levels [38]. Likewise, digital transformation is seen as a strategic asset for healthcare professionals, as it enables centralized access to information gathered by different actors, improves data access, and supports personalized care.

Recognizing limited access to information as a barrier to PCP is critical, as developing health information support structures is considered fundamental to its realization [2,39,40]. However, it is important to highlight that informed participation does not depend solely on individuals’ ability to access information and make decisions about their health. It also requires a power balance transformation, which has historically been dominated by healthcare professionals, something that may be particularly challenging in more deferential cultural contexts [1,41,42]. Shifting power relations involves creating spaces for shared decision-making that are culturally sensitive and adapted to each person’s individuality [43].

Within the health and social policies domain, Class 4 reflects a political vision of the decision-making structure, emphasizing the need for health system reform. In contrast, Class 1 offers a more operational and systemic political perspective, focused on coordination and collaboration across sectors, institutions, and all actors in care interactions. Although for over a decade, national strategic documents have acknowledged the need for structural and cultural transformation of the health system, such as the National Health Plan 2012–2016 [13], SNS+ Proximity: Person-Centered Change [44] and the National Health Plan 2030 [45], the pace of change has been slow and incomplete. The repeated launch of new plans reinforcing the same principles suggests ongoing challenges in implementation. Resistance from professionals, organizational barriers, and the fragmentation of the care delivery system may hinder PCP implementation [26,46].

A collaborative discourse is reinforced in Class 1, highlighting that collaborative health policies address the need for coordinated interventions across services and levels of care, ensuring an integrated response for vulnerable populations. Intersectoral integration and shared accountability emerge as essential pillars for advancing PCP, enabling the co-construction of appropriate responses based on mutual commitment [2].

### 4.4 Strategic framework

The operationalization of PCP depends on strategic frameworks that translate political vision into concrete practices supported by mechanisms for evaluation and continuous improvement [9].

Class 1 revealed that the implementation of health policies focused on person-centredness has been framed through various strategic plans, such as the National Strategy for Kidney Health [47], the Action Plan for Active and Healthy Ageing [48], and the National Plan for the Reduction of Addictive Behaviors and Dependencies 2030 [49]. These documents apply the PCP concept more concretely, establishing clear goals, priority areas, and guiding principles that serve as reference points for implementing integrated, participatory, and PCP. Additionally, the Integrated Care Pathways [50–53] were identified as operational models that support the transition from political vision to clinical practice. Each pathway is designed for a specific clinical condition, involves multidisciplinary teams, and promotes personalized care plans, ensuring coordinated transitions throughout the patient journey across the health system. By providing concrete guidance for the individualization and coordination of care, with strategies adapted to local realities, these instruments can enhance PCP implementation [2,40].

Within this framework, the factorial analysis and the DHC revealed discursive coherence in articulating public policies, organizational culture, and technological transformation as key structural pillars for implementing PCP. This alignment between political discourse and proposals for organizational innovation is promising for creating systemic conditions conducive to paradigm change. However, the isolated positioning of Class 5 suggests that the macro-level vision of the health system remains relatively disconnected from the operational dynamics of care necessary to realize this transformation. Its function appears to be more guiding than integrative. Thus, while the need for change is widely recognized, its realization requires more than strategies outlined in normative documents, it demands coherent and sustained actions across all levels of intervention [2,54].

The strategic framework also includes the development of tools to assess practices and outcomes, as well as the implementation of mechanisms for continuous improvement. Although the potential for health gains resulting from an integrated system response is acknowledged, and the need for systematic practice evaluation is emphasized, no specific assessment instruments are referenced. Significant gaps exist in the capacity to consistently and comprehensively monitor the progress or impact of PCP implementation. While the importance of evaluating processes and outcomes and incorporating stakeholder feedback is recognized, evaluation often prioritizes biomedical and efficiency indicators, overlooking subjective dimensions such as service user experience [38,55]. Given that the development of PCP has the potential to generate meaningful benefits for all stakeholders, it is essential to establish participatory evaluation instruments systematically to support the ongoing advancement of this approach [38,56].

The availability of tools aligned with the PCPF, translated, adapted, and culturally validated for the Portuguese population, such as the Person-centred Practice Inventory-Staff (PCPI-S) [57] and Person-centred Practice Inventory-Care (PCPI-C) [58], may represent a valuable contribution to addressing this gap.

### 4.5 Workforce development

Workforce development is a critical pillar for sustaining PCP, yet the analysis revealed structural gaps that hinder its consolidation. The absence of a structured framework focused on academic training, or the strategic management of human resources represents a significant weakness in the operationalization of PCP. As healthcare professionals are the main drivers in building organizational cultures aligned with PCP values, the lack of guidelines at this level undermines the effective transformation of care practices and structures.

Class 2 identified support strategies for healthcare professionals to facilitate the integration of PCP, particularly through continuous professional development, the cultivation of specific competencies, and the promotion of reflective practice. Continuous training and support for multidisciplinary teams through strategies that foster critical reflection on practice and ensure the sharing of values are essential for the effective implementation of PCP [59,60]. However, it is important to note that the discourse analyzed lacks clear guidance on implementing these strategies, revealing a gap between declared intentions and operationalization.

This gap may be linked to the tension between biomedical and humanistic approaches highlighted in Class 5. While the biomedical paradigm emphasizes disease, technical expertise, and efficiency, PCP demands relational, ethical, and reflective competencies, elements often undervalued in health professionals’ academic curricula [39]. The absence of a structured framework for workforce development is not only a planning issue; it also reflects a paradigmatic resistance that continues to shape how care is conceptualized, taught, and practised.

In the similarity analysis, healthcare professionals appeared connected to the person node, suggesting some recognition of their individuality as participants in care relationships. However, in the DHC, they were predominantly represented in a functional role as system resources. The lack of recognition for the personal dimension of all those involved contradicts the foundational values of PCP, ultimately compromising its sustainability [9]. Supporting and valuing healthcare professionals is essential to ensure the successful implementation of PCP.

### 4.6 Limitations

The corpus analyzed consisted exclusively of documents written in Portuguese, which may have excluded relevant sources published in other languages, even if focused on the Portuguese healthcare context. The analysis predominantly focused on governmental documents and reports from independent entities without directly incorporating healthcare professionals’ perspectives or individuals interacting with the healthcare system. This limitation constrains the characterization of the macro context of PCP, as it does not capture the experiences, perceptions, and challenges faced by the main stakeholders involved.

## 5 Conclusion

The analysis enabled a comprehensive characterization of the framing of PCP within the Portuguese healthcare system, based on national strategic documents published between 2013 and 2024 and guided by the macrocontextual constructs of the PCPF.

The methodological robustness of the lexicometric analysis, ensured by the high retention rate and the substantial number of active forms, allowed for the identification of relevant discursive trends. However, including only 11% of the initially identified documents highlights a significant gap in national documentation, particularly concerning implementation models, operational challenges, and evaluation strategies related to PCP in the Portuguese context.

The results reveal several characteristics that pose considerable challenges to the effective implementation of PCP. These include conceptual and terminological inconsistencies that hinder the development of a shared understanding of care; a disconnect between political directives and everyday practice, reflecting difficulties in translating policy into fundamental changes in care delivery; a lack of explicit guidance on academic and professional training; the persistence of a biomedical paradigm with asymmetrical power dynamics; and the scarcity of evaluation tools sensitive to the subjective experiences of both service users and professionals.

At the same time, several promising developments were identified. The growing trend of integrating PCP into legislative and strategic documents reflects a strong political commitment to healthcare system transformation. Initiatives such as establishing structures to support the humanization of care, implementing health literacy promotion strategies, investments in digital transformation, and defining practical models for implementing PCP, such as the Integrated Care Pathways, indicate progressive change. The availability of validated monitoring instruments like the PCPI-S and PCPI-C inventories may help address the identified evaluation gap.

Identifying and addressing the structural and cultural barriers that persist is essential for the political commitment to PCP to be consistently translated into daily care. The realization of PCP relies on organizational contexts that promote person-centered values and create the conditions for professionals to act in alignment with these principles. Investment in academic training, mechanisms for professional recognition, and support structures for developing multidisciplinary teams are indispensable strategic elements to drive sustainable transformation aligned with PCP's goals.

## Supporting information

S1 Appendix Inclusion criteria. (DOCX)

S1 Table Descending hierarchical classification profiles. (DOCX)

S2 Appendix Textual corpus. (DOCX)

S3 Appendix Factorial representation of the semantic structure of the classes. (DOCX)

S4 Appendix Factorial representation of the semantic structure of the variables of interest. (DOCX)

S5 Appendix Similarity graph. (DOCX)
