# Supplementary material for: Person-centered practice in the Portuguese healthcare system: A documentary study
Source: PLoS One. 2026 Mar 3;21(3):e0343419. doi: 10.1371/journal.pone.0343419 (PMC12956081; doi:10.1371/journal.pone.0343419)
Supplement: S1 Appendix — (DOCX) [file pone.0343419.s001.docx]

**Eligibility criteria**

**This appendix summarizes the eligibility criteria that were defined according to the PCC framework, Population, Concept, and Context.**

**Population:** Healthcare professionals and healthcare service users

**Inclusion criteria:**

- Official documents addressing person-centered practice in the context of healthcare delivery.
- Texts published by regulatory, professional, and governmental institutions in Portugal.
- Reports, strategic plans, legislation, and guidelines involving healthcare professionals and service users in defining or implementing person-centered practice.

**Exclusion criteria:**

- Documents focused exclusively on other populations (e.g., individual case studies, personal experiences without systemic impact).
- Publications discussing person-centered practice outside healthcare (e.g., standalone social services, education, or corporate sectors).

**Concept:** Person-centered practice

**Inclusion criteria:**

- Documents explicitly referencing terms related to person-centered practice, such as citizen/person-centeredness, person-/user-/patient-centered care, shared decision-making, and citizen engagement.
- Texts describing principles, strategies, or evaluation indicators of person-centered practice.

**Exclusion criteria:**

- Documents that do not directly mention person-centered practice or use the term without defining or applying it in a relevant manner.
- Texts addressing care centrality without connection to policies, strategies, or institutional guidelines.

**Context:** Healthcare in Portugal

**Inclusion criteria:**

- Publications related to the organization and regulation of healthcare in Portugal.
- Documents issued by institutions responsible for setting clinical practice standards and health policy recommendations.
- Publication period between 2012 and 2024, considering the 2012–2016 National Health Plan as the formal introduction of the concept.

**Exclusion criteria:**

- Documents published prior to 2012 as they do not reflect the formal introduction of the concept in the Portuguese context.
- Opinion pieces, blogs, or journalistic news without regulatory or normative basis.
- International studies without reference to the Portuguese context, unless cited as a reference for national policies.
