# Supplementary material for: Person-centered practice in the Portuguese healthcare system: A documentary study
Source: PLoS One. 2026 Mar 3;21(3):e0343419. doi: 10.1371/journal.pone.0343419 (PMC12956081; doi:10.1371/journal.pone.0343419)
Supplement: S1 Table — (DOCX) [file pone.0343419.s002.docx]

| **Table 1** – Profiles from the Descending Hierarchical Classification (with significance, i.e., χ² equal to or greater than 3.84). | | | | | | | | | | | | | | |
| --- | --- | --- | --- | --- | --- | --- | --- | --- | --- | --- | --- | --- | --- | --- |
| **Class 1** | | | **Class 2** | | | **Class 3** | | | **Class 4** | | | **Class 5** | | |
| **word** | **%** | **χ²** | **word** | **%** | **χ²** | **word** | **%** | **χ²** | **word** | **%** | **χ²** | **word** | **%** | **χ²** |
| response | 72.13 | 54.63 | participation | 63.64 | 75.41 | information | 50.41 | 104.22 | new | 47.69 | 74.76 | diabetes | 100.00 | 108.54 |
| intervention | 66.67 | 47.20 | humanization | 70.00 | 52.10 | data | 75.86 | 69.06 | pact | 100.00 | 67.64 | therapeutic | 88.89 | 83.74 |
| coordination | 85.71 | 42.22 | citizenship | 76.47 | 36.92 | indvidualized care plan | 67.74 | 54.75 | object | 90.00 | 52.98 | integrated care pathway | 100.00 | 78.65 |
| integrate | 61.11 | 35.51 | participative | 78.57 | 32.56 | decision | 51.06 | 37.54 | leadership | 71.43 | 42.91 | expectation | 77.14 | 75.51 |
| nhs | 53.51 | 33.75 | promote | 37.50 | 30.56 | portal | 100.00 | 37.24 | establish | 71.43 | 42.91 | treatment | 78.79 | 75.15 |
| network | 76.67 | 31.76 | organizational | 76.92 | 28.55 | record | 83.33 | 35.67 | health system | 40.00 | 41.53 | risk | 78.79 | 75.15 |
| measure | 67.57 | 25.54 | develop | 52.94 | 26.22 | personal | 83.33 | 35.67 | report | 75.00 | 41.32 | renal | 85.71 | 58.86 |
| eficiency | 75.00 | 23.44 | rehearse | 100.00 | 25.58 | unique | 100.00 | 32.55 | national_health_council | 100.00 | 40.39 | chronic | 64.86 | 49.41 |
| answer | 78.95 | 21.85 | practical | 45.10 | 23.70 | registo_de_saúde_eletrónico | 100.00 | 32.55 | envision | 61.11 | 37.58 | dialysis | 100.00 | 49.26 |
| focus | 85.71 | 20.76 | necessary | 39.47 | 22.39 | tomar | 60.00 | 31.29 | duty | 29.84 | 36.29 | assistential | 80.00 | 46.70 |
| equity | 85.71 | 20.76 | competence | 60.00 | 22.15 | constitute | 60.87 | 29.91 | future | 57.89 | 34.58 | vascular | 100.00 | 45.10 |
| prevention | 62.50 | 20.69 | profissional | 53.85 | 20.93 | path | 50.00 | 29.68 | society | 45.71 | 34.48 | familiar | 63.33 | 37.07 |
| to the | 50.00 | 20.48 | reflection | 85.71 | 20.26 | system | 41.10 | 29.54 | alliance | 100.00 | 33.62 | moment | 100.00 | 36.81 |
| form | 53.42 | 20.33 | politic | 51.85 | 19.34 | electronic | 100.00 | 27.87 | target | 85.71 | 32.96 | patient | 46.58 | 35.99 |
| aging | 90.91 | 19.44 | culture | 54.55 | 18.35 | my_health_agenda | 100.00 | 23.20 | change | 45.45 | 31.92 | always | 76.47 | 35.05 |
| well_being | 69.23 | 19.32 | local | 45.00 | 18.20 | health | 23.95 | 22.28 | ministry_of_health | 66.67 | 30.95 | pre_obesity | 100.00 | 32.69 |
| namely | 66.67 | 17.53 | action | 45.95 | 18.02 | essential | 50.00 | 21.99 | big | 70.00 | 29.03 | adjust | 100.00 | 32.69 |
| long | 68.00 | 17.40 | exercise | 66.67 | 17.81 | clinic | 54.55 | 20.80 | vision | 43.75 | 27.75 | domiciliary | 78.57 | 30.93 |
| target | 71.43 | 17.31 | aplication | 66.67 | 17.81 | good | 55.00 | 19.33 | base | 61.54 | 27.46 | receive | 83.33 | 30.91 |
| increase | 90.00 | 17.12 | evaluation | 58.82 | 17.70 | centralization | 100.00 | 18.53 | function | 75.00 | 27.41 | adult | 83.33 | 30.91 |
| garantee | 100.00 | 16.28 | letter | 75.00 | 16.32 | library | 100.00 | 18.53 | individual | 56.25 | 26.93 | guide | 83.33 | 30.91 |
| nhs_24 | 100.00 | 16.28 | representative | 75.00 | 16.32 | user | 100.00 | 18.53 | fund | 100.00 | 26.86 | multidisciplinar | 61.54 | 29.39 |
| alternative | 100.00 | 16.28 | coletive | 83.33 | 16.12 | collection | 100.00 | 18.53 | respetive | 100.00 | 26.86 | modality | 88.89 | 27.32 |
| reinforcement | 78.57 | 15.76 | iniciative | 61.54 | 15.37 | integrator | 100.00 | 18.53 | friend | 100.00 | 26.86 | physical | 76.92 | 27.10 |
| diverse | 65.38 | 15.69 | foster | 61.54 | 15.37 | plataform | 75.00 | 18.03 | ministry | 100.00 | 26.86 | team | 48.94 | 26.56 |
| context | 66.67 | 15.53 | active | 45.16 | 14.13 | inform | 75.00 | 18.03 | lead | 100.00 | 26.86 | diagnosis | 68.75 | 24.58 |
| social | 53.57 | 15.46 | comunication | 46.43 | 13.98 | home | 75.00 | 18.03 | emphasis | 83.33 | 26.40 | circuit | 100.00 | 24.46 |
| cycle | 81.82 | 14.05 | mecanismo | 50.00 | 13.94 | clear | 75.00 | 18.03 | represente | 83.33 | 26.40 | cardiovascular | 100.00 | 24.46 |
| prioritize | 100.00 | 13.94 | create | 42.11 | 13.61 | book | 83.33 | 17.71 | organism | 63.64 | 25.23 | nutricion | 100.00 | 24.46 |
| community_based | 100.00 | 13.94 | regional | 66.67 | 13.31 | achieve | 83.33 | 17.71 | government | 66.67 | 23.13 | sight | 75.00 | 23.33 |
| provision | 50.00 | 13.67 | representative | 66.67 | 13.31 | relevant | 56.25 | 16.44 | perform | 66.67 | 23.13 | carer | 53.33 | 21.99 |
| proximidity | 66.67 | 13.54 | forum | 100.00 | 12.74 | service | 31.78 | 16.27 | hospital | 47.62 | 22.78 | hospitalization | 100.00 | 20.36 |
| duration | 87.50 | 12.55 | dissemination | 100.00 | 12.74 | source | 63.64 | 15.97 | agente | 58.33 | 22.08 | enshrine | 100.00 | 20.36 |
| establishing | 87.50 | 12.55 | channel | 100.00 | 12.74 | thematic | 100.00 | 13.88 | propose | 71.43 | 21.28 | clinic | 50.00 | 20.31 |
| growing | 87.50 | 12.55 | respect | 50.00 | 12.64 | provision | 100.00 | 13.88 | leader | 71.43 | 21.28 | experience | 85.71 | 19.29 |
| acessibility | 87.50 | 12.55 | governance | 50.00 | 12.64 | inteligente | 100.00 | 13.88 | group | 45.45 | 21.00 | available | 57.14 | 18.89 |
| acess | 49.25 | 12.45 | training | 47.83 | 12.61 | information_tecnology | 100.00 | 13.88 | system | 30.14 | 20.68 | use | 66.67 | 16.82 |
| principle | 57.58 | 12.15 | qualitative | 80.00 | 12.06 | informed | 71.43 | 13.85 | consultant | 100.00 | 20.12 | person | 28.29 | 16.75 |
| fenomenon | 80.00 | 11.87 | interpersonal | 80.00 | 12.06 | behavior | 58.33 | 13.64 | accountability | 80.00 | 19.94 | based | 58.82 | 16.62 |
| internal | 100.00 | 11.60 | instrument | 45.83 | 11.41 | improved | 80.00 | 13.28 | advisory | 80.00 | 19.94 | case | 58.82 | 16.62 |
| discrimination | 100.00 | 11.60 | monitoring | 50.00 | 11.35 | area | 41.18 | 13.19 | municipality | 80.00 | 19.94 | parameter | 100.00 | 16.27 |
| equality | 100.00 | 11.60 | organization | 39.47 | 10.67 | citizen | 26.74 | 11.72 | general | 50.00 | 17.20 | metabolic | 100.00 | 16.27 |
| inclusive | 100.00 | 11.60 | facilitate | 53.85 | 10.31 | importance | 53.85 | 11.70 | role | 42.86 | 16.94 | individualization | 100.00 | 16.27 |
| functional | 100.00 | 11.60 | involvement | 45.45 | 10.14 | make_available | 53.85 | 11.70 | part | 40.00 | 16.57 | focal | 100.00 | 16.27 |
| focus | 100.00 | 11.60 | already | 50.00 | 10.07 | digital | 42.31 | 10.98 | education | 40.91 | 15.53 | spiritual | 100.00 | 16.27 |
| emphasys | 100.00 | 11.60 | institution | 38.89 | 9.51 | enable | 33.93 | 10.62 | association | 66.67 | 15.37 | correctly | 100.00 | 16.27 |
| decree_law | 100.00 | 11.60 | ansure | 36.17 | 9.37 | older_adult | 50.00 | 10.07 | administration | 66.67 | 15.37 | advanced | 100.00 | 16.27 |
| consequent | 100.00 | 11.60 | good | 45.00 | 8.88 | utilize | 50.00 | 10.07 | council | 55.56 | 14.54 | respect | 70.00 | 16.04 |
| complexity | 100.00 | 11.60 | significant | 66.67 | 8.84 | particularly | 66.67 | 9.85 | current | 42.11 | 14.53 | activity | 52.17 | 15.58 |
| increase | 75.00 | 11.55 | value_enhancement | 66.67 | 8.84 | care | 66.67 | 9.85 | center | 35.48 | 14.35 | psychological | 83.33 | 15.35 |
| guarantee | 50.94 | 11.47 | procedure | 66.67 | 8.84 | more | 30.77 | 9.83 | community | 30.61 | 14.23 | cost_effective | 83.33 | 15.35 |
| dimension | 71.43 | 11.44 | mobilization | 66.67 | 8.84 | management | 29.89 | 9.63 | deliberation | 75.00 | 13.64 | palliative | 55.56 | 14.79 |
| improve | 60.00 | 10.81 | interest | 66.67 | 8.84 | instrument | 41.67 | 9.59 | defend | 75.00 | 13.64 | same | 55.56 | 14.79 |
| deepening | 85.71 | 10.29 | portugal | 50.00 | 8.79 | purpose | 75.00 | 8.97 | requirement | 75.00 | 13.64 | scope | 42.86 | 14.77 |
| transversal | 85.71 | 10.29 | maximum | 75.00 | 8.12 | currently | 75.00 | 8.97 | prepare | 75.00 | 13.64 | necessity | 32.23 | 13.69 |
| guiding | 85.71 | 10.29 | purpose | 75.00 | 8.12 | process | 28.43 | 8.91 | chapter | 57.14 | 12.14 | effective | 45.16 | 13.01 |
| hospital_related | 57.14 | 9.93 | internacional | 75.00 | 8.12 | manager | 55.56 | 8.85 | partnership | 42.86 | 11.20 | itself | 50.00 | 12.95 |
| fundamental | 64.71 | 9.76 | stimulate | 75.00 | 8.12 | contribute | 37.50 | 8.79 | committee | 36.36 | 10.88 | performance | 66.67 | 12.57 |
| associated | 77.78 | 9.74 | decision_maker | 75.00 | 8.12 | central | 50.00 | 8.61 | present | 45.45 | 10.36 | transplantation | 100.00 | 12.18 |
| given | 77.78 | 9.74 | self_management | 75.00 | 8.12 | protection | 42.11 | 7.83 | sector | 33.33 | 10.17 | questionnaire | 100.00 | 12.18 |
| gain | 77.78 | 9.74 | plan | 36.84 | 08.09 | security | 38.46 | 7.80 | environment | 40.00 | 9.82 | peritoneal | 100.00 | 12.18 |
| effectiveness | 72.73 | 9.53 | healthcare_professional | 28.57 | 08.01 | follow | 57.14 | 7.45 | partner | 40.00 | 9.82 | nuclear | 100.00 | 12.18 |
| innovative | 72.73 | 9.53 | existence | 55.56 | 7.82 | full | 57.14 | 7.45 | independent | 60.00 | 9.81 | induction | 100.00 | 12.18 |
| analyze | 100.00 | 9.27 | exist | 55.56 | 7.82 | goal | 57.14 | 7.45 | who | 60.00 | 9.81 | equal | 100.00 | 12.18 |
| inequality | 100.00 | 9.27 | depend | 55.56 | 7.82 | concept | 57.14 | 7.45 | perform | 60.00 | 9.81 | hospitalization | 100.00 | 12.18 |
| simplification | 100.00 | 9.27 | confidentiality | 55.56 | 7.82 | evaluation | 34.21 | 7.30 | involve | 50.00 | 9.76 | schedule | 100.00 | 12.18 |
| resolution | 100.00 | 9.27 | national_health_plan | 50.00 | 7.51 | recognize | 46.15 | 7.24 | become | 41.67 | 8.83 | pharmacological | 100.00 | 12.18 |
| premise | 100.00 | 9.27 | institutional | 50.00 | 7.51 | provider | 50.00 | 7.16 | sustainable | 41.67 | 8.83 | potential | 100.00 | 12.18 |
| advocate | 100.00 | 9.27 | transformation | 46.67 | 7.50 | result | 30.91 | 6.88 | central | 41.67 | 8.83 | cumpliance | 100.00 | 12.18 |
| positively | 100.00 | 9.27 | literacy | 37.50 | 7.27 | also | 31.91 | 6.75 | support | 35.00 | 8.75 | complication | 100.00 | 12.18 |
| metodology | 100.00 | 9.27 | commission | 40.91 | 6.94 | addictive_behaviors_and_dependencies | 41.18 | 6.46 | transition | 37.50 | 8.64 | timely | 100.00 | 12.18 |
| introduce | 100.00 | 9.27 | country | 57.14 | 6.61 | literacy | 34.38 | 6.22 | recomendation | 44.44 | 7.94 | antecipation | 100.00 | 12.18 |
| indispensable | 100.00 | 9.27 | individualized | 57.14 | 6.61 | record | 60.00 | 6.11 | still | 33.33 | 7.85 | offer | 71.43 | 11.84 |
| duplication | 100.00 | 9.27 | exemple | 57.14 | 6.61 | personalization | 45.45 | 5.81 | orient | 66.67 | 7.66 | live | 80.00 | 11.47 |
| combat | 100.00 | 9.27 | device | 57.14 | 6.61 | priority | 50.00 | 5.71 | suggest | 66.67 | 7.66 | recognized | 80.00 | 11.47 |
| expand | 100.00 | 9.27 | improvement | 31.25 | 6.60 | multiple | 50.00 | 5.71 | representation | 66.67 | 7.66 | departure | 80.00 | 11.47 |
| to_center | 42.59 | 8.93 | proces | 28.43 | 6.51 | complete | 50.00 | 5.71 | nominate | 66.67 | 7.66 | condition | 80.00 | 11.47 |
| coordination | 59.09 | 8.91 | portuguese | 46.15 | 6.25 | participate | 38.89 | 5.57 | insufficient | 66.67 | 7.66 | action | 58.33 | 11.37 |
| situation | 50.00 | 8.58 | identify | 46.15 | 6.25 | partner | 40.00 | 5.13 | configure | 66.67 | 7.66 | scientific | 42.42 | 11.05 |
| integration | 45.59 | 8.24 | community | 32.65 | 6.17 | review | 66.67 | 4.91 | add | 66.67 | 7.66 | define | 45.83 | 10.52 |
| approach | 51.35 | 8.17 | continuos | 37.04 | 5.80 | resident | 66.67 | 4.91 | attention | 38.46 | 7.55 | style | 60.00 | 10.29 |
| concern | 83.33 | 08.07 | nhs_closer_to_people | 41.18 | 5.46 | key_actor | 66.67 | 4.91 | highlight | 50.00 | 7.30 | point | 53.85 | 9.62 |
| maintenance | 83.33 | 08.07 | substance | 60.00 | 5.44 | periodical | 66.67 | 4.91 | need | 50.00 | 7.30 | give | 47.37 | 9.29 |
| homeless | 62.50 | 08.04 | relationship | 60.00 | 5.44 | step | 66.67 | 4.91 | also | 25.53 | 6.89 | high | 62.50 | 9.26 |
| state | 62.50 | 08.04 | introduction | 60.00 | 5.44 | market | 66.67 | 4.91 | cost | 40.00 | 6.51 | dife | 62.50 | 9.26 |
| following | 64.29 | 7.82 | infrastructure | 60.00 | 5.44 | connection | 66.67 | 4.91 | demand | 40.00 | 6.51 | continuity | 40.62 | 09.06 |
| understand | 75.00 | 7.67 | incentive | 60.00 | 5.44 | it_related | 66.67 | 4.91 | citizen | 18.60 | 5.95 | condition | 38.89 | 8.61 |
| global | 66.67 | 7.65 | manage | 60.00 | 5.44 | statistical | 66.67 | 4.91 | program | 26.47 | 5.67 | want | 54.55 | 8.46 |
| different | 47.06 | 7.27 | deepen | 60.00 | 5.44 | document | 66.67 | 4.91 | structure | 33.33 | 5.57 | conduct | 54.55 | 8.46 |
| fact | 100.00 | 6.94 | commitment | 42.86 | 5.19 | design | 66.67 | 4.91 | vast | 42.86 | 5.55 | ensure | 36.17 | 8.38 |
| universality | 100.00 | 6.94 | reality | 50.00 | 4.99 | critical | 66.67 | 4.91 | make_available | 42.86 | 5.55 | referral | 66.67 | 8.35 |
| simultaneously | 100.00 | 6.94 | priority | 50.00 | 4.99 | contain | 66.67 | 4.91 | appeal | 42.86 | 5.55 | guide | 66.67 | 8.35 |
| simplicity | 100.00 | 6.94 | still | 50.00 | 4.99 | circular | 66.67 | 4.91 | health | 15.58 | 05.05 | timely | 66.67 | 8.35 |
| street | 100.00 | 6.94 | framework | 50.00 | 4.99 | azorean | 66.67 | 4.91 | put | 50.00 | 4.86 | satisfaction | 45.00 | 8.18 |
| regulate | 100.00 | 6.94 | trust | 50.00 | 4.99 | allocation | 66.67 | 4.91 | personality | 50.00 | 4.86 | physician | 50.00 | 8.16 |
| problematic | 100.00 | 6.94 | centrality | 32.50 | 4.87 | counseling | 66.67 | 4.91 | peer | 50.00 | 4.86 | evidence | 40.00 | 7.98 |
| privacy | 100.00 | 6.94 | part | 36.00 | 4.75 | access | 66.67 | 4.91 | modification | 50.00 | 4.86 | option | 75.00 | 7.70 |
| assumption | 100.00 | 6.94 | participate | 38.89 | 4.65 | update | 41.67 | 4.73 | less | 50.00 | 4.86 | negociation | 75.00 | 7.70 |
| remain | 100.00 | 6.94 | change | 33.33 | 4.49 | problema | 41.67 | 4.73 | legal | 50.00 | 4.86 | consideration | 75.00 | 7.70 |
| pandemic | 100.00 | 6.94 | right | 33.33 | 4.49 | pass | 41.67 | 4.73 | learning | 50.00 | 4.86 | suggestion | 75.00 | 7.70 |
| multiprofissional | 100.00 | 6.94 | population | 32.43 | 4.44 | capacity | 34.78 | 4.64 | very | 31.25 | 4.79 | complaint | 75.00 | 7.70 |
| multidimensionality | 100.00 | 6.94 | will | 66.67 | 4.41 | knowledge | 31.43 | 4.62 | function | 31.25 | 4.79 | preventive | 75.00 | 7.70 |
| geographic | 100.00 | 6.94 | reference | 66.67 | 4.41 | identified | 44.44 | 4.40 | policy | 33.33 | 4.44 | humanized | 75.00 | 7.70 |
| demographic | 100.00 | 6.94 | board | 66.67 | 4.41 | safe | 44.44 | 4.40 | pass | 33.33 | 4.44 | empathy | 75.00 | 7.70 |
| shared_accountability | 100.00 | 6.94 | path | 66.67 | 4.41 | provide | 37.50 | 4.31 | beyond | 33.33 | 4.44 | emotional | 75.00 | 7.70 |
| co_production | 100.00 | 6.94 | \|  \| \| --- \|  \| health_illness \| \| --- \| | 66.67 | 4.41 | comprehensive | 50.00 | 4.27 | population | 24.32 | 4.37 | ecosystem | 75.00 | 7.70 |
| circulation | 100.00 | 6.94 | rigorous | 66.67 | 4.41 | effect | 50.00 | 4.27 | place | 37.50 | 4.28 | thing | 75.00 | 7.70 |
| act | 100.00 | 6.94 | deadline | 66.67 | 4.41 | actively | 50.00 | 4.27 | term | 37.50 | 4.28 | annual | 75.00 | 7.70 |
| retain | 100.00 | 6.94 | pilot | 66.67 | 4.41 | based | 50.00 | 4.27 | interaction | 37.50 | 4.28 | care | 24.83 | 7.42 |
| affiliation | 100.00 | 6.94 | obtained | 66.67 | 4.41 | acess | 26.87 | 04.06 | signify | 37.50 | 4.28 | safe | 55.56 | 7.31 |
| search | 58.82 | 6.71 | médium | 66.67 | 4.41 |  |  |  | however | 37.50 | 4.28 | necessary | 31.58 | 7.27 |
| autonomy | 54.55 | 6.32 | municipal | 66.67 | 4.41 |  |  |  | framework | 37.50 | 4.28 | nurse | 50.00 | 6.98 |
| follow_up | 54.55 | 6.32 | mobilize | 66.67 | 4.41 |  |  |  | patient | 20.55 | 4.00 | particular | 50.00 | 6.98 |
| care | 35.43 | 5.96 | legislative | 66.67 | 4.41 |  |  |  | illness | 19.77 | 3.85 | elemento | 46.67 | 6.93 |
| treat | 80.00 | 5.90 | labor | 66.67 | 4.41 |  |  |  |  |  |  | adoption | 46.67 | 6.93 |
| telehealth | 80.00 | 5.90 | investigation | 66.67 | 4.41 |  |  |  |  |  |  | hospital_related | 39.29 | 6.91 |
| complementarity | 80.00 | 5.90 | inquiry | 66.67 | 4.41 |  |  |  |  |  |  | tend | 40.00 | 6.61 |
| socioeconomic | 80.00 | 5.90 | illicit | 66.67 | 4.41 |  |  |  |  |  |  | education | 40.91 | 6.33 |
| systematic | 80.00 | 5.90 | equitable | 66.67 | 4.41 |  |  |  |  |  |  | clinic | 40.91 | 6.33 |
| referred | 80.00 | 5.90 | dissemination | 66.67 | 4.41 |  |  |  |  |  |  | relevance | 57.14 | 6.19 |
| recovery | 80.00 | 5.90 | availability | 66.67 | 4.41 |  |  |  |  |  |  | appeal | 57.14 | 6.19 |
| execution | 80.00 | 5.90 | dedicate | 66.67 | 4.41 |  |  |  |  |  |  | identify | 46.15 | 5.77 |
| stage | 80.00 | 5.90 | believe | 66.67 | 4.41 |  |  |  |  |  |  | support | 36.67 | 5.56 |
| meeting | 80.00 | 5.90 | conceptual | 66.67 | 4.41 |  |  |  |  |  |  | ensure | 32.08 | 5.35 |
| diversify | 80.00 | 5.90 | fit | 66.67 | 4.41 |  |  |  |  |  |  | illness | 29.07 | 5.16 |
| directly | 80.00 | 5.90 | learn | 66.67 | 4.41 |  |  |  |  |  |  | quality | 28.16 | 5.13 |
| dependence | 80.00 | 5.90 | align | 66.67 | 4.41 |  |  |  |  |  |  | norm | 60.00 | 5.11 |
| occur | 80.00 | 5.90 | duty | 25.81 | 4.21 |  |  |  |  |  |  | barrier | 60.00 | 5.11 |
| cooperation | 80.00 | 5.90 | technology | 36.84 | 3.95 |  |  |  |  |  |  | acute | 60.00 | 5.11 |
| wide | 80.00 | 5.90 |  |  |  |  |  |  |  |  |  | primary | 37.50 | 4.86 |
| sense | 63.64 | 5.89 |  |  |  |  |  |  |  |  |  | objective | 42.86 | 4.76 |
| consolidate | 63.64 | 5.89 |  |  |  |  |  |  |  |  |  | place | 50.00 | 4.63 |
| erpi | 66.67 | 5.72 |  |  |  |  |  |  |  |  |  | concrete | 50.00 | 4.63 |
| only | 66.67 | 5.72 |  |  |  |  |  |  |  |  |  | rational | 50.00 | 4.63 |
| reorganization | 66.67 | 5.72 |  |  |  |  |  |  |  |  |  | coordinated | 50.00 | 4.63 |
| reduce | 66.67 | 5.72 |  |  |  |  |  |  |  |  |  | personalization | 45.45 | 4.61 |
| qualification | 66.67 | 5.72 |  |  |  |  |  |  |  |  |  | phase | 45.45 | 4.61 |
| holistic | 66.67 | 5.72 |  |  |  |  |  |  |  |  |  | provide | 33.33 | 4.33 |
| avoid | 66.67 | 5.72 |  |  |  |  |  |  |  |  |  | nursing | 66.67 | 4.16 |
| challenge | 66.67 | 5.72 |  |  |  |  |  |  |  |  |  | support | 66.67 | 4.16 |
| até | 66.67 | 5.72 |  |  |  |  |  |  |  |  |  | apropriate | 66.67 | 4.16 |
| functioning | 71.43 | 5.67 |  |  |  |  |  |  |  |  |  | wish | 66.67 | 4.16 |
| requirement | 71.43 | 5.67 |  |  |  |  |  |  |  |  |  | vulnerability | 66.67 | 4.16 |
| enipssa | 71.43 | 5.67 |  |  |  |  |  |  |  |  |  | so | 66.67 | 4.16 |
| efficient | 71.43 | 5.67 |  |  |  |  |  |  |  |  |  | technically | 66.67 | 4.16 |
| adopt | 71.43 | 5.67 |  |  |  |  |  |  |  |  |  | nhs_evidence | 66.67 | 4.16 |
| continuous | 56.25 | 5.23 |  |  |  |  |  |  |  |  |  | obstacle | 66.67 | 4.16 |
| orientation | 56.25 | 5.23 |  |  |  |  |  |  |  |  |  | minimization | 66.67 | 4.16 |
| level | 41.25 | 05.07 |  |  |  |  |  |  |  |  |  | specifically | 66.67 | 4.16 |
| user | 42.42 | 05.03 |  |  |  |  |  |  |  |  |  | listen | 66.67 | 4.16 |
| integrated | 50.00 | 4.96 |  |  |  |  |  |  |  |  |  | balance | 66.67 | 4.16 |
| intervene | 58.33 | 4.55 |  |  |  |  |  |  |  |  |  | emiti | 66.67 | 4.16 |
| quality_of_life | 58.33 | 4.55 |  |  |  |  |  |  |  |  |  | economy | 66.67 | 4.16 |
| contribute | 46.88 | 4.36 |  |  |  |  |  |  |  |  |  | decide | 66.67 | 4.16 |
| centrality | 45.00 | 4.33 |  |  |  |  |  |  |  |  |  | harm | 66.67 | 4.16 |
| proactive | 60.00 | 4.25 |  |  |  |  |  |  |  |  |  | comorbidities | 66.67 | 4.16 |
| identification | 60.00 | 4.25 |  |  |  |  |  |  |  |  |  | agreed | 66.67 | 4.16 |
| addictive_behaviors_and_dependencies | 52.94 | 4.23 |  |  |  |  |  |  |  |  |  |  |  |  |
| mechanism | 50.00 | 4.18 |  |  |  |  |  |  |  |  |  |  |  |  |
| mental | 62.50 | 3.98 |  |  |  |  |  |  |  |  |  |  |  |  |
| evolution | 62.50 | 3.98 |  |  |  |  |  |  |  |  |  |  |  |  |
| be_based_on | 62.50 | 3.98 |  |  |  |  |  |  |  |  |  |  |  |  |
| existing | 53.33 | 3.86 |  |  |  |  |  |  |  |  |  |  |  |  |
| structure | 53.33 | 3.86 |  |  |  |  |  |  |  |  |  |  |  |  |
