# Supplementary material for: Person-centered practice in the Portuguese healthcare system: A documentary study
Source: PLoS One. 2026 Mar 3;21(3):e0343419. doi: 10.1371/journal.pone.0343419 (PMC12956081; doi:10.1371/journal.pone.0343419)
Supplement: S2 Appendix — (DOCX) [file pone.0343419.s003.docx]

**Textual Corpus**

Portuguese version:

**** *id_9 *doc_2 *ano_2018 *autor_1 *área_3

O sns visa assegurar a proteção da saúde e garantir o acesso a cuidados a todos os cidadãos. Desta forma, a análise de qualquer alteração ou medida a implementar no setor da saúde deverá ser, naturalmente, centrada no cliente, nos potenciais efeitos na saúde e no acesso aos cuidados de saúde pela população, devendo garantir que as medidas a tomar estão não só alinhadas com aqueles que são os objetivos primordiais do sns, mas que têm também potencial de contribuir para a sua melhor concretização. A análise dos potenciais efeitos da especialização e da formação avançada em enfermagem deverá ser orientada nesse sentido, procurando averiguar se a prática especializada de enfermagem poderá, ou não, contribuir para obtenção de ganhos em saúde e para um melhor acesso aos cuidados de saúde.

**** *id_11 *doc_4 *ano_2013 *autor_2 *área_1

Os processo_assistencial_integrado colocam o cidadão, com as suas necessidades e expectativas, no centro do sistema. A continuidade assistencial e a coordenação entre os diferentes níveis de cuidados, são reconhecidos como elementos essenciais para garantir que o doente recebe os melhores cuidados de saúde, atempados e efetivos. A abordagem dos processo_assistencial_integrado é uma abordagem multidisciplinar, integral e integrada que pressupõe a reanálise de todas as atuações de que o doente é alvo em qualquer ponto do sns, do início ao fim do processo_assistencial_integrado. Por outro lado, as atividades assistenciais baseadas na melhor evidência científica disponível, respeitam o princípio do uso racional de tecnologias da saúde e orientam a adoção de atuações terapêuticas custo_efetivo, ao mesmo tempo que se garante ao cidadão a qualidade clínica que é consagrada como um dos seus principais direitos. Se pretende proporcionar a mudança organizacional, com base no envolvimento de todos os profissional_de_saúde implicados na prestação de cuidados, acreditando na sua capacidade e vontade de melhorar continuamente a qualidade e de centrar os seus esforços nas pessoas. Os processo_assistencial_integrado são ainda uma ferramenta que permite analisar as diferentes componentes que intervêm na prestação de cuidados de saúde e ordenar os diferentes fluxos de trabalho, integrando o conhecimento atualizado, homogeneizando as atuações e colocando ênfase nos resultados, a fim de dar resposta às expectativas quer dos cidadãos quer dos profissional_de_saúde. É pois necessário orientar a prática clínica para os resultados e para a qualidade, tendo como objetivo a utilização mais efetiva dos recursos. A gestão por processo_assistencial_integrado apela para a reorganização dos cuidados prestados aos doentes pelos serviços de saúde, os centrando no cidadão, e neste caso particular, nas necessidades e expectativas da pessoa com diabetes tipo 2, e tendo sempre em consideração 2 parâmetros fundamentais aos quais é preciso dar resposta: Para quem fazemos as coisas, e como as fazer corretamente. Se deseja que se efetue uma leitura flexível daquilo que no papel está definido, tendo em consideração os recursos disponíveis, para fazer uma melhor adaptação com vista à melhoria da prestação de cuidados às pessoas com diabetes tipo 2. Foi assim que foi pensado este documento: como material de trabalho, adaptável ao âmbito local específico de cada unidade prestadora de cuidados de saúde. O diabetes tipo 2 se encontrar em consonância com os objetivos estratégicos e os principais indicadores definidos no programa nacional para a diabetes, mas tem, especificamente, um âmbito circunscrito: se centra no circuito da pessoa com diagnóstico de diabetes, como é próprio do desenho geral dos processo_assistencial_integrado. As necessidades e expectativas da pessoa com diabetes, e dos seus familiares e cuidadores, são o elemento nuclear e o ponto de partida para o desenvolvimento deste processo_assistencial_integrado. Essas necessidades e expectativas foram identificadas através de várias fontes de informação, nomeadamente questionários de satisfação, sugestões, reclamações, e toda a experiência colhida do desenvolvimento dos programas nacionais, nomeadamente do programa nacional para a diabetes e grupos focais. Elas foram tidas em conta no desenvolvimento das diferentes componentes deste processo_assistencial_integrado. De igual modo, os profissional_de_saúde que atendem a pessoa com diabetes têm as suas próprias expectativas e necessidades, cuja satisfação condiciona a qualidade dos cuidados prestados. Em cada nível de cuidados ou âmbito de atuação os profissional_de_saúde envolvidos deverão procurar e identificar expectativas específicas da pessoa com diabetes e seus familiares relacionadas com o momento da prestação de cuidados e o ambiente concreto em que os mesmos se desenvolvem. O médico de família e enfermeiro, com apoio da restante equipa multidisciplinar, a trabalhar de forma coordenada, promovem, colaboram e realizam o rastreio anual das complicações crónicas adiante discriminadas. Confirmado o diagnóstico, a pessoa com diabetes recebe cuidados adequados nos cuidados primários com o objetivo da estabilização, pesquisa de eventuais processos intercorrentes e inicio de educação terapêutica, no âmbito do trabalho de equipa multidisciplinar, nomeadamente: promoção da autonomia e responsabilização no tratamento; tratamento não farmacológico: intervenção nos estilos de vida, prescrição de alimentação saudável e atividade física adaptada às características individuais, no sentido de alcançar os objetivos de controlo metabólico; início do tratamento farmacológico sempre que necessário, depois de avaliado o resultado das medidas de modificação de estilos de vida; autovigilância e autocontrolo: início da autovigilância e autocontrolo sempre que possível em função dos objetivos do controlo metabólico e da monitorização clínica. Ponderar e decidir sobre o tipo e modelo de glucómetro tendo em conta as necessidades e especificidades individuais. À pessoa com diabetes, familiar ou cuidador e durante o processo de educação terapêutica é assegurada pela equipa multidisciplinar, com clareza e adequação, a informação ou formação relativa ao uso seguro dos medicamentos prescritos, eventuais efeitos secundários, conservação, administração e horários das tomas, esclarecendo todas as dúvidas ou questões solicitadas. A equipa multidisciplinar dos cuidados primários assegura, periodicamente e nas condições ajustadas a cada pessoa com diabetes, o preenchimento correto e completo do guia, a preencher sempre que possível pelo próprio com a ajuda dos profissional_de_saúde. No último trimestre de cada ano civil, o médico de família e enfermeiro promovem a negociação com a pessoa com diabetes dos objetivos terapêuticos anuais para o ano seguinte, se assumindo com a pessoa com diabetes, familiar ou cuidador, o compromisso de que todos os profissional_de_saúde dos cuidados primários vão participar ativamente na sua concretização. Acordados que estejam os objetivos anuais, estes são registados no processo clínico médico e de enfermagem bem como no guia, se obrigando todos os profissional_de_saúde dos cuidados primários a promover as intervenções adequadas que lhes digam respeito para os atingir. No âmbito da educação terapêutica e no decurso do processo de educação terapêutica a equipa multidisciplinar assegura a informação e ensino adequados sobre o guia e promove a participação ativa da pessoa com diabetes na concretização dos objetivos terapêuticos acordados. O médico de família e enfermeiro procedem às intervenções relacionadas com o risco cardiovascular, nomeadamente, obesidade, hipertensão arterial e dislipidemia, ajustadas a cada caso em particular. A equipa multidisciplinar trabalha de forma coordenada e articulada, promove, colabora e garante a continuidade assistencial no âmbito das competências específicas de cada profissional_de_saúde. A equipa multidisciplinar assegura, entre si, a permuta adequada de toda a informação clínica e não clínica no âmbito da prestação de cuidados de saúde de qualidade à pessoa com diabetes. A equipa multidisciplinar avalia todos os fatores que influenciam os hábitos de saúde para intervir sobre aqueles que são determinantes para os estilos de vida saudáveis e para realizar educação terapêutica e adaptar o plano terapêutico e de monitorização clínica às características individuais da pessoa com diabetes: conhecimentos, experiências e crenças sobre diabetes e seu tratamento, incluindo a programação de todas as tarefas inerentes à sua adequada vigilância; nível de instrução e de compreensão; estado de ânimo, ansiedade e sensação de bem_estar; condição física: acuidade visual, capacidades psicomotoras; hábitos de vida: vida social, tempo de lazer, tabaco, álcool; alimentação saudável: intolerância, gosto e ou preferências, tipo, número e horário de refeições diárias; trabalho: horário, atividade física, deslocações; família: relações e apoio familiar; situação económica: equilíbrio financeiro; autocuidado e terapêutica: barreiras e ou dificuldades. A equipa multidisciplinar assegura a educação terapêutica, fornecendo a informação e educação necessárias e adequadas para um maior conhecimento da diabetes e treino das capacidades necessárias para colaborar ativamente no seu controlo metabólico. Esta fase deve se realizar tão precocemente quanto possível e deve ser personalizada, de acordo com as características individuais de cada pessoa com diabetes, nas consultas de vigilância, se ajustando e adequando ao longo do tempo. Apoio psicológico e apoio social com especial ênfase na integração social e familiar. A equipa multidisciplinar participa na educação terapêutica e na promoção de estilos de vida saudável, alimentação e prática de exercício físico. O nutricionista e o dietista asseguram a educação para a prática de alimentação equilibrada, variada e completa às pessoas em risco e com diabetes. A equipa multidisciplinar assegura educação terapêutica no âmbito do reconhecimento e prevenção das hipoglicemias bem como das complicações crónicas da diabetes. A equipa multidisciplinar assegura educação terapêutica no treino em técnicas de autoadministração e autovigilância. A equipa multidisciplinar assegura a comunicação de toda a informação e formação necessárias para capacitar a pessoa com diabetes ao preenchimento de todos os dados, sua descrição, interpretação e registo no guia. A equipa multidisciplinar promove a criação de associações de pessoas com diabetes a um nível de proximidade com o seu meio social, com envolvimento da comunidade. O médico e o enfermeiro trabalham em equipa, se articulando sempre que necessário com outros técnicos e profissional_de_saúde, e promovendo uma adequada e atempada prestação de cuidados de saúde à pessoa com diabetes proveniente dos cuidados de saúde primários, no âmbito de consulta específica de diabetes. Em particular, no que concerne às complicações de retinopatia diabética, nefropatia diabética, pé diabético e diabetes gestacional, os profissional_de_saúde dos cuidados hospitalares garantem a continuidade de cuidados de saúde nos termos definidos pelas normas e orientações da direção geral da saúde, de forma a responder às necessidades das pessoas com diabetes. No âmbito dos cuidados hospitalares, compete assegurar, quer na consulta, quer em caso de internamento, e qualquer que seja o motivo clínico, entre outros: apoio personalizado, suporte emocional e empatia adequados; avaliação do controlo metabólico, perfil glicémico, A1c, lípidos, assim como avaliação de índice de massa corporal e tensão arterial; avaliação, rastreio e tratamento de intercorrências. O médico e enfermeiro, e, sempre que necessário, se articulando com outros profissional_de_saúde, trabalham de forma coordenada, se garantindo uma avaliação global, incluindo a avaliação do risco cardiovascular, a avaliação de retinopatia, nefropatia e pé diabético. No relatório de alta a enviar à equipa dos cuidados hospitalares ou cuidados continuados integrados, devem constar as atividades realizadas durante o internamento, os diagnósticos e decisões clínicas assim como o plano terapêutico e de cuidados. Em nota de alta de enfermagem, há informação sobre a atividade educativa realizada e o plano de monitorização e dos cuidados recomendados para o nível de cuidados a que se destina. O médico, enfermeiro, assistente social, terapeuta trabalham em equipa, e promovem uma atempada referenciação por forma a assegurar a continuidade da prestação de cuidados de saúde à pessoa com diabetes proveniente de outro nível de cuidados. A equipa multidisciplinar estabelece e define um plano_individual_de_cuidados para cada pessoa com diabetes. A equipa multidisciplinar identifica, através das avaliações clínicas e sociais, a presença de limitações para o autocuidado. A equipa multidisciplinar faculta à pessoa com diabetes as ferramentas pertinentes que lhe facilitem o autocuidado: nas limitações sensoriais, nas limitações de mobilidade, nas limitações cognitivas.

**** *id_12 *doc_4 *ano_2014 *autor_2 *área_1

Os processo_assistencial_integrado colocam o cidadão, com as suas necessidades e expectativas, no centro do sistema. A continuidade assistencial e a coordenação entre os diferentes níveis de cuidados, são reconhecidos como elementos essenciais para garantir que o doente recebe os melhores cuidados de saúde, atempados e efetivos. A abordagem dos processo_assistencial_integrado é uma abordagem multidisciplinar, integral e integrada que pressupõe a reanálise de todas as atuações de que o doente é alvo em qualquer ponto do sns, do início ao fim do processo_assistencial_integrado. Por outro lado, as atividades assistenciais baseadas na melhor evidência científica disponível, respeitam o princípio do uso racional de tecnologias da saúde e orientam a adoção de atuações terapêuticas custo_efetivo, ao mesmo tempo que se garante ao cidadão a qualidade clínica que é consagrada como um dos seus principais direitos. Se pretende proporcionar a mudança organizacional, com base no envolvimento de todos os profissional_de_saúde implicados na prestação de cuidados, acreditando na sua capacidade e vontade de melhorar continuamente a qualidade e de centrar os seus esforços nas pessoas. Os processo_assistencial_integrado são ainda uma ferramenta que permite analisar as diferentes componentes que intervêm na prestação de cuidados de saúde e ordenar os diferentes fluxos de trabalho, integrando o conhecimento atualizado, homogeneizando as atuações e colocando ênfase nos resultados, a fim de dar resposta às expectativas quer dos cidadãos quer dos profissional_de_saúde. É necessário orientar a prática clínica para os resultados e para a qualidade, tendo como objetivo a utilização mais efetiva dos recursos. A gestão da prática clínica, tal como definida nos processo_assistencial_integrado, apela para a reorganização dos cuidados prestados às pessoas pelo sistema_de_saúde, os centrando no cidadão e, neste caso particular, nas necessidades e expectativas da pessoa com risco vascular e na antecipação e planeamento integral da continuidade dos cuidados. São 2 os parâmetros a considerar: para quem fazemos as coisas e como as fazer corretamente. O profissional_de_saúde proporciona a informação necessária para promover a participação e decisão da pessoa com risco vascular, incluindo o familiar e cuidador, lhe permitindo que exerça os seus direitos. As intervenções dirigidas à modificação do estilo de vida são tão importantes como as medidas farmacológicas e em muitos casos, com eficácia superior, melhorando o controlo dos fatores de risco vascular e a morbilidade e mortalidade vasculares, pelo que se devem implementar de forma personalizada na pessoa com risco cardiovascular. Conjunto de atividades através das quais se identifica na pessoa adulta a presença ou não de fator de risco vascular e ou doença vascular conhecida, se avalia o risco cardiovascular global e em função do qual se programa e planifica, com a pessoa e ou cuidador as atividades preventivas, o acompanhamento terapêutico, de monitorização e de cuidados de saúde. Toda a atuação clínica implica a continuidade de cuidados de saúde e a existência de coordenação entre os diferentes profissional_de_saúde e os diferentes níveis de cuidados de saúde. As necessidades e expectativas da pessoa com risco cardiovascular e dos seus familiares e cuidadores são o elemento nuclear e o ponto de partida para o desenvolvimento deste processo_assistencial_integrado. Essas necessidades e expectativas foram identificadas através de várias fontes de informação, nomeadamente resultados de questionários de satisfação, sugestões, reclamações, desenvolvimento dos programas nacionais e grupos focais. Elas estão presentes nas diferentes componentes deste processo_assistencial_integrado. De igual modo, os profissional_de_saúde que atendem a pessoa com risco vascular têm as suas próprias expectativas e necessidades, cuja satisfação condiciona a qualidade dos cuidados prestados. Em cada nível de cuidados ou âmbito de atuação os profissional_de_saúde envolvidos deverão procurar e identificar expectativas específicas da pessoa com risco vascular e dos seus familiares e cuidadores, relacionadas com o momento da prestação de cuidados e o ambiente concreto em que os mesmos se desenvolvem. A corresponsabilização ativa das pessoas no tratamento e gestão da sua doença é imprescindível ao sucesso da atividade clínica baseada nas recomendações e consensos científicos existentes. A intervenção de todos os profissional_de_saúde e da comunidade na implementação de medidas de prevenção das doenças cérebro_cardiovasculares é primordial. Por todas estas razões, a efetividade e eficiência das intervenções em relação à pessoa com risco vascular requer a ação de equipas multidisciplinares de profissional_de_saúde, se exigindo uma apropriada comunicação e cooperação a fim de se evitarem atividades episódicas não interligadas, com duplicação de atos e desperdício de recursos, de que resultam cuidados desestruturados, mal controlados e sem se encontrar a melhor solução para os problemas das pessoas, originando a sua insatisfação. O profissional_de_saúde proporciona a informação necessária para promover a participação e decisão da pessoa com risco vascular, incluindo o familiar ou cuidador, lhe permitindo que exerça os seus direitos. O profissional_de_saúde assegura a comunicação mais eficaz com cada pessoa com risco vascular, aplicando as técnicas de comunicação próprias do seu âmbito profissional. O profissional_de_saúde assegura cuidados clínicos de qualidade, atualizados e baseados na melhor evidência científica, no âmbito profissional da sua especialidade. O profissional_de_saúde assegura continuidade de cuidados assistenciais à pessoa com risco vascular no cumprimento deste processo_assistencial_integrado.

**** *id_14 *doc_4 *ano_2016 *autor_2 *área_1

Os processo_assistencial_integrado colocam o cidadão, com as suas necessidades e expectativas, no centro do sns. A continuidade assistencial e a coordenação entre os diferentes níveis de cuidados, são reconhecidos como elementos essenciais para garantir que o doente recebe os melhores cuidados de saúde, atempados e efetivos. A abordagem por processo_assistencial_integrado é multidisciplinar, integral e integrada, que pressupõe a reanálise de todas as atuações de que o doente é alvo em qualquer ponto do sns, do início ao fim do processo_assistencial_integrado. Por outro lado, as atividades assistenciais baseadas na melhor evidência científica disponível, respeitam o princípio do uso racional de tecnologias da saúde e orientam a adoção de atuações terapêuticas custo_efetivo, ao mesmo tempo que se garante ao cidadão a qualidade clínica, que é consagrada como um dos seus principais direitos. Se pretende proporcionar a mudança organizacional, com base no envolvimento de todos os profissional_de_saúde implicados na prestação de cuidados, acreditando na sua capacidade e vontade de melhorar continuamente a qualidade e de centrar os seus esforços nas pessoas. Os processo_assistencial_integrado são, ainda, uma ferramenta que permite analisar as diferentes componentes que intervêm na prestação de cuidados de saúde e ordenar os diferentes fluxos de trabalho, integrando o conhecimento atualizado, homogeneizando as atuações e colocando ênfase nos resultados, a fim de dar resposta às expectativas, quer dos cidadãos, quer dos profissional_de_saúde. É necessário orientar a prática clínica para os resultados e para a qualidade, tendo como objetivo a utilização mais efetiva dos recursos. A gestão da prática clínica, tal como definida nos processo_assistencial_integrado, apela para a reorganização dos cuidados prestados às pessoas pelo sns, os centrando no cidadão e, neste caso particular, nas necessidades e expectativas da pessoa adulta com pré_obesidade e na antecipação e planeamento integral da continuidade de cuidados. São 2 os parâmetros a considerar: para quem fazemos as coisas e como as fazer corretamente. O processo_assistencial_integrado da pré_obesidade no adulto se encontra em consonância com os objetivos estratégicos e os principais indicadores definidos no programa nacional para a promoção da alimentação saudável mas tem, especificamente, um âmbito circunscrito: se centra no circuito da pessoa adulta com diagnóstico de pré_obesidade, como é próprio do desenho geral dos processo_assistencial_integrado. As necessidades e expectativas da pessoa adulta com pré_obesidade e dos seus familiares e cuidadores, são o elemento nuclear e o ponto de partida para o desenvolvimento deste processo_assistencial_integrado. Essas necessidades e expectativas foram identificadas através de várias fontes de informação, nomeadamente questionários de satisfação, sugestões, reclamações, e toda a experiência colhida do desenvolvimento dos programas nacionais, nomeadamente do programa nacional para a promoção da alimentação saudável e grupos focais. Elas foram tidas em conta no desenvolvimento das diferentes componentes deste processo_assistencial_integrado bem como, relativamente aos profissional_de_saúde que atendem a pessoa adulta com pré_obesidade, as suas próprias expectativas e necessidades, cuja satisfação condiciona a qualidade dos cuidados prestados. Em cada nível de cuidados ou âmbito de atuação os profissional_de_saúde envolvidos deverão procurar e identificar expectativas específicas da pessoa adulta com pré_obesidade, e seus familiares, relacionadas com o momento da prestação de cuidados e o ambiente concreto em que os mesmos se desenvolvem. O profissional_de_saúde proporciona a informação necessária para promover a participação e decisão da pessoa adulta com pré_obesidade incluindo o familiar e cuidador, lhe permitindo que exerça os seus direitos. O profissional_de_saúde assegura a comunicação eficaz com cada pessoa adulta com pré_obesidade, aplicando as técnicas de comunicação próprias do seu âmbito profissional. O profissional_de_saúde assegura cuidados clínicos de qualidade na pré_obesidade, atualizados e baseados na melhor evidência científica, no âmbito profissional da sua especialidade. O profissional_de_saúde assegura continuidade de cuidados assistenciais à pessoa adulta com pré_obesidade no cumprimento deste processo_assistencial_integrado.

**** *id_16 *doc_5 *ano_2015 *autor_2 *área_3

O relatório desenvolvido pela oms sobre a implementação do plano_nacional_de_saúde conclui que o plano_nacional_de_saúde 2012_2016 está alinhado com a estratégia Health_2020 da oms, devendo, no entanto, serem reforçados os instrumentos de implementação. O relatório Um Futuro para a Saúde assentou no desafio de criar uma visão para a saúde e para os cuidados de saúde em Portugal, para os próximos 25 anos, descrevendo o que isso poderia significar na prática, nomeadamente em termos de implementação e sustentabilidade. Este documento propõe uma visão simples, caracterizada pelo empowerment dos cidadãos, pela participação ativa da sociedade e por uma procura contínua de qualidade, apelando a um novo pacto para a saúde. O modelo de coprodução de saúde refere que por um lado a governação para a saúde partilhada pelos diferentes setores da sociedade, incluindo a administração pública e a governação da saúde, prestação de cuidados de saúde contribuem em simultâneo para o estado e ganhos em saúde na população. Este modelo é inspirador para a formulação das orientações deste plano_nacional_de_saúde. A estratégia Health_2020 da oms é o quadro de referência para as políticas europeias de saúde. As prioridades estratégicas são: investir na saúde ao longo do ciclo de vida, capacitando os cidadãos; combater as doenças transmissíveis e não transmissíveis; fortalecer os sistema_de_saúde centrados nas pessoas, bem como a capacidade de resposta em saúde_pública, nomeadamente a vigilância, preparação e resposta a ameaças; desenvolver comunidades resilientes e ambientes protetores. O plano_nacional_de_saúde tem como valores e princípios a transparência e a responsabilização que permitam a confiança e valorização dos agentes, bem como que o sistema se desenvolva aprendendo. Para além destes se destacam: o envolvimento e participação de todos os intervenientes nos processos de criação de saúde; a redução das desigualdades em saúde, como base para a promoção da equidade e justiça social; a integração e continuidade dos cuidados prestados aos cidadãos; um sistema_de_saúde que responda com rapidez às necessidades, utilizando da melhor forma os recursos disponíveis para evitar o desperdício; a sustentabilidade, de forma a preservar estes valores para o futuro, em que se possa conjugar: uma população saudável; comunidades resilientes que possam dispor de uma boa rede informal de cuidados; políticas e práticas de saúde bem integradas nas outras políticas e práticas sociais e económicas; um sistema de cuidados de saúde bem concebido e centrado nas pessoas, adequado aos objetivos, que seja eficiente, e tenha recursos humanos adequados, qualificados e a trabalhar em equipa, de modo a cuidados de saúde integrados. O cidadão, entendido como central no sistema_de_saúde, é um importante agente de participação e de mudança. Por isso, tem o direito e o dever de influenciar as decisões em política de saúde que afetam coletivamente a população, no desempenho dos seus diferentes papéis: o de doente com necessidades específicas; o de consumidor com expectativas e direito a cuidados seguros e de qualidade; e o de contribuinte do sns. Do mesmo modo, o cidadão deve ser capacitado para assumir a responsabilidade de pugnar pela defesa da sua saúde individual e da saúde coletiva. Para a exercer, o cidadão tem que estar informado, tem que interiorizar tal informação e a traduzir na alteração dos seus comportamentos menos saudáveis e, quando for o caso, na gestão da sua doença. Só assim o cidadão, individual ou coletivamente, estará capacitado para ser ouvido e participar nas decisões que lhe dizem respeito, contribuindo para o consenso quanto às prioridades em matéria de saúde e para um compromisso político estável e alargado que permita alcançar os objetivos deste plano_nacional_de_saúde. O plano_nacional_de_saúde propõe: A promoção de uma cultura de cidadania que vise a promoção da literacia e da capacitação dos cidadãos, de modo que se tornem mais autónomos e responsáveis em relação à sua saúde e à saúde de quem deles depende. A promoção da participação ativa das organizações representativas dos interesses dos cidadãos. O desenvolvimento de competências nos profissional_de_saúde que permitam desenvolver ações de cidadania em saúde; O desenvolvimento de programas de educação para a saúde e de autogestão da doença. O desenvolvimento de programas de utilização racional e adequada dos serviços de saúde.

**** *id_17 *doc_5 *ano_2022 *autor_2 *área_3

Segundo a oms, o compromisso da liderança envolve vários requisitos como: a transparência por parte dos doentes e dos profissional_de_saúde, tanto na partilha da informação como na redução de uma abordagem hierarquizada, numa perspetiva transversal, a comunicação aberta e com respeito, o desenvolvimento de uma cultura de aprendizagem com os erros e com as melhores práticas, o trabalho em equipa, a vontade de aprender, a valorização e apoio aos profissional_de_saúde, a par do equilíbrio judicioso entre a política de não culpabilização e a responsabilização. Assim sendo, estes requisitos, se tornam indispensáveis à cultura da segurança e sobrevêm da liderança, tendo presente que o doente deve estar no centro dos cuidados e do sistema. Este processo requer uma forte liderança a todos os níveis, por parte do ministério_da_saúde, das instituições de saúde, parceiros e todas as equipas envolvidas na prestação de cuidados. A introdução e desenvolvimento de ferramentas de telessaúde é já uma realidade nacional em alguns contextos. Porém, a sua expansão para os diferentes níveis de cuidados constitui ainda um desafio, pois a implementação de modelos de telessaúde só fará sentido se acrescentar valor à saúde das pessoas, garantindo, desta forma, os princípios da humanização, qualidade e segurança dos cuidados. A introdução de mecanismos inovadores deverá contribuir para o modelo de cuidados personalizados, centrados na pessoa, acompanhando as alterações demográficas e a inovação biotecnológica, as expectativas das pessoas e a capacitação dos serviços para a demonstração de resultados, assentes em princípios de qualidade na saúde. Neste contexto, no âmbito da inovação em saúde, para o desenvolvimento e execução de projetos de teleconsulta e telemonitorização de sucesso podem vir a ser tidos em conta, os seguintes pressupostos: Mais humanização na prestação de cuidados de saúde; Integração da melhor evidência científica de forma rápida e atempada; Definição clara e inequívoca de percursos de cuidados, para facilitar a navegabilidade do cidadão pelos diferentes níveis de cuidados; Máxima segurança na prestação de cuidados de saúde, o que inclui, a elaboração de procedimentos, registos, avaliação do risco e monitorização com segurança dos dados, entre outros. A integração dos conceitos e linhas orientadoras para o aumento da qualidade e segurança na saúde como os modelos da telessaúde, está condicionada por variáveis externas ao processo de desenvolvimento tecnológico, que deverão ser interpretadas como desafios para a implementação desta ferramenta, sendo eles: o nível de literacia digital dos cidadãos; a articulação dos profissionais da área informática com os profissional_de_saúde; a criação de programas de qualificação dos profissional_de_saúde neste âmbito; a definição do um plano_individual_de_cuidados em conjunto com o doente; a promoção do domicílio como um nível de cuidados; a definição de critérios que garantam a qualidade dos cuidados prestados e suportados pela telemonitorização; a não substituição da interação pessoal entre profissional_de_saúde e doente e cuidador e família; a definição de processos seguros; a proteção de dados pessoais. O plano nacional para a segurança dos doentes se constitui como uma ferramenta de apoio a gestores de topo, lideranças intermédias, colaboradores das comissões da qualidade e segurança, gestores da segurança do doente e profissional_de_saúde, exigindo um envolvimento ativo de responsabilidade de governação, coordenação e operacionalização nos diferentes níveis de cuidados, de modo a aumentar a segurança da prestação de cuidados de saúde, tendo presente o foco no doente e foco nos cuidadores.

**** *id_18 *doc_5 *ano_2014 *autor_2 *área_3

Tendo por base os 2 grandes domínios da procura e da oferta, se entende que, no domínio da procura, o cidadão constitui o centro da concetualização das políticas e intervenções nos comportamentos_aditivos_e_dependências, partindo do pressuposto que é fundamental responder às necessidades dos indivíduos, perspetivadas de forma dinâmica no decurso do seu ciclo de vida. Se pretende desenvolver intervenções globais e abrangentes que integrem um contínuo que vai da promoção da saúde, prevenção, dissuasão, redução de riscos e minimização de danos, ao tratamento e à reinserção social. Se incluem ainda neste domínio duas medidas estruturantes, o plano operacional de respostas integradas e a rede de referenciação ou articulação no âmbito dos comportamentos_aditivos_e_dependências, através das quais se pretende responder de forma eficaz e sustentável às necessidades atuais nesta área. O reconhecimento de que o cidadão é diverso, em etapas do ciclo de vida, género, nacionalidade, estatuto económico, social, educacional, cultural, familiar, estilos de personalidade, conhecimentos, conceções de bem_estar, entre outras dimensões possíveis, e que tem experiências diversas na sua utilização deste tipo de mediadores de bem_estar, em função de variáveis internas e externas, do produto ou atividade, da fase do ciclo de vida, se reflete na priorização das vertentes da atualização da informação, conhecimento e competências; do desenvolvimento de condições para a inovação e adequação das intervenções à diversidade; da cooperação nos planos do saber e da ação, visando uma intervenção personalizada, de qualidade, e integrada com outros setores, nos níveis local e nacional. Centralidade no cidadão: No plano nacional contra a droga e toxicodependências 2005_2012 se considerou que a intervenção em toxicodependências não constitui um fim em si mesmo, devendo se descentrar das substâncias e assumir a centralidade no cidadão e nas suas necessidades objetivas e subjetivas. Para o próximo ciclo estratégico se mantém este princípio, alargando o enfoque para além das toxicodependências, compreendendo o vasto leque de comportamentos_aditivos_e_dependências. Considerando ainda a atual reorganização dos serviços neste âmbito, compete ao estado assegurar a agilização da burocracia e dos processos que visam a promoção e prestação de cuidados de saúde, em função da satisfação global das necessidades dos indivíduos, tendo em conta que estes têm uma palavra ativa e uma corresponsabilidade na definição do seu projeto de vida, em termos equitativos de direitos e deveres de cidadania. Os serviços devem, pois, se constituir como gestores do capital de saúde dos cidadãos, cabendo a estes a decisão de a eles recorrer. Assim, para o atual ciclo estratégico se destaca a centralidade no cidadão, numa perspetiva dinâmica do seu ciclo de vida, que se desenvolve ao longo das diferentes etapas. O indivíduo é corresponsável e gestor das suas opções e comportamentos que visem a sua saúde, qualidade_de_vida e bem_estar, enquanto cidadão e utente dos serviços, assim como elemento ativo promotor do exercício de cidadania nos contextos que frequenta, ao longo das diferentes etapas da sua vida. Intervenção Integrada: Os processos que conduzem a comportamentos_aditivos_e_dependências são diversos e, muitas vezes, cumulativos. Têm um carácter estruturante e as suas manifestações podem ser económicas, sociais, políticas e até culturais. Se deve entender a integração como uma resposta global, concertada e de conjunto, que contempla as várias dimensões dos fenómenos, permitindo articular estrategicamente as ações a desenvolver. Assim, a complexidade inerente aos comportamentos_aditivos_e_dependências requer uma visão holística e desenvolvimental, onde se encara o indivíduo como o centro da abordagem, a desfocando da substância utilizada ou comportamentos manifestados. Fazer face a essa complexidade exige abordagens multidisciplinares e um leque de respostas e dispositivos que, de forma articulada e congruente, atuem nas várias vertentes deste fenómeno. Nesse sentido, a consolidação de um modelo integrado pressupõe um contínuo interdependente de respostas, designadamente de prevenção, dissuasão, redução de riscos e minimização de danos, tratamento e reinserção. O modelo de respostas integradas se baseia, portanto, numa leitura multidimensional da realidade dos comportamentos_aditivos_e_dependências e numa intervenção de proximidade, multissetorial e trans_setorial, que permite maximizar resultados e alcançar ganhos sociais e de saúde. Esta conceção se distancia da parcialidade da visão da mera soma e justaposição das intervenções, se tornando fundamental um forte investimento na articulação interinstitucional e a formulação de objetivos estratégicos transversais às intervenções, evitando assim a dispersão, aproveitando todo o conjunto dos recursos disponíveis e as potenciais sinergias. No âmbito do tratamento, a intervenção deve se centrar em abordagens que impliquem um diagnóstico individualizado e uma resposta assente na oferta de uma rede que garanta cuidados adequados e continuados, em função da patologia apresentada e eventuais comorbilidades. Se realça, assim, a relevância do seu carácter abrangente e transversal em toda a intervenção na problemática da toxicodependência. Deste modo, interessa adequar as estratégias de intervenção à situação em que o indivíduo se encontra, o que se traduz na integração das intervenções entre os vários tipos de intervenção, procurando sempre garantir uma abordagem baseada numa lógica de satisfação das necessidades do indivíduo, adequando as respostas disponíveis às intervenções diagnosticadas como necessárias. O enfoque da intervenção deverá se centrar nas diferentes perspetivas de abordagem, designadamente nas áreas da prevenção, redução de riscos e minimização de danos, tratamento, reinserção, favorecendo a proximidade e acessibilidade das respostas aos indivíduos que delas necessitam. Para o efeito é fundamental a sensibilização e capacitação de profissional_de_saúde e de outros interventores e a criação de condições para o desenvolvimento de uma intervenção articulada e intersetorial, necessária para que os serviços de resposta especializados, designadamente prestação de cuidados de saúde primários e a rede social, providenciem um acompanhamento adequado desta problemática na etapa final do ciclo de vida. Considerando, ainda, a centralidade no cidadão, enquanto valor fundamental da intervenção, em que as necessidades de formação são identificadas junto dos serviços, por via de diagnósticos rigorosos, tendo em vista a melhoria e eficácia do serviço público.

**** *id_23 *doc_3 *ano_2019 *autor_2 *área_3

O direito à proteção da saúde é o direito de todas as pessoas gozarem do melhor estado de saúde físico, mental e social, pressupondo a criação e o desenvolvimento de condições económicas, sociais, culturais e ambientais que garantam níveis suficientes e saudáveis de vida, de trabalho e de lazer. O direito à proteção da saúde constitui uma responsabilidade conjunta das pessoas, da sociedade e do estado e compreende o acesso, ao longo da vida, à promoção, prevenção, tratamento e reabilitação da saúde, a cuidados continuados e a cuidados paliativos. Base 2: Todas as pessoas têm direito: à proteção da saúde com respeito pelos princípios da igualdade, não discriminação, confidencialidade e privacidade; a aceder aos cuidados de saúde adequados à sua situação, com prontidão e no tempo considerado clinicamente aceitável, de forma digna, de acordo com a melhor evidência científica disponível e seguindo as boas práticas de qualidade e segurança em saúde; a escolher livremente a entidade prestadora de cuidados de saúde, na medida dos recursos existentes; a receber informação sobre o tempo de resposta para os cuidados de saúde de que necessitem; a ser informadas de forma adequada, acessível, objetiva, completa e inteligível sobre a sua situação, o objetivo, a natureza, as alternativas possíveis, os benefícios e riscos das intervenções propostas e a evolução provável do seu estado de saúde em função do plano de cuidados a adotar; a decidir, livre e esclarecidamente, a todo o momento, sobre os cuidados de saúde que lhe são propostos, salvo nos casos excecionais previstos na lei, a emitir diretivas antecipadas de vontade e a nomear procurador de cuidados de saúde; a aceder livremente à informação que lhes respeite, sem necessidade de intermediação de um profissional_de_saúde, exceto se por si solicitado; a ser acompanhadas por familiar ou outra pessoa por si escolhida e a receber assistência religiosa e espiritual; a apresentar sugestões, reclamações e a obter resposta das entidades responsáveis; a intervir nos processos de tomada de decisão em saúde e na gestão participada das instituições do sns; a constituir entidades que as representem e defendam os seus direitos e interesses, nomeadamente sob a forma de associações para a promoção da saúde e prevenção da doença, de ligas de amigos e de outras formas de participação que a lei preveja; à promoção do bem_estar e qualidade_de_vida durante o envelhecimento, numa perspetiva inclusiva e ativa que favoreça a capacidade de decisão e controlo da sua vida, através da criação de mecanismos adaptativos de aceitação, de autonomia e independência, sendo determinantes os fatores socioeconómicos, ambientais, da resposta social e dos cuidados de saúde. São fundamentos da política de saúde: a promoção da saúde e a prevenção da doença, devendo ser consideradas na definição e execução de outras políticas públicas; a melhoria do estado de saúde da população, através de uma abordagem de saúde_pública, da monitorização e vigilância epidemiológica e da implementação de planos de saúde nacionais, regionais e locais; as pessoas, como elemento central na conceção, organização e funcionamento de estabelecimentos, serviços e respostas de saúde; a igualdade e a não discriminação no acesso a cuidados de saúde de qualidade em tempo útil, a garantia da equidade na distribuição de recursos e na utilização de serviços e a adoção de medidas de diferenciação positiva de pessoas e grupos em situação de maior vulnerabilidade; a promoção da educação para a saúde e da literacia para a saúde, permitindo a realização de escolhas livres e esclarecidas para a adoção de estilos de vida saudável; a participação das pessoas, das comunidades, dos profissional_de_saúde e dos órgãos municipais na definição, no acompanhamento e na avaliação das políticas de saúde; a gestão dos recursos disponíveis segundo critérios de efetividade, eficiência e qualidade; todos os profissional_de_saúde que trabalham no sns têm direito a uma carreira profissional que reconheça a sua diferenciação na área da saúde. Base 13: O estado promove a melhoria da saúde mental das pessoas e da sociedade em geral, designadamente através da promoção do bem_estar mental, da prevenção e identificação atempada das doenças mentais e dos riscos a elas associados. Os cuidados de saúde mental devem ser centrados nas pessoas, reconhecendo a sua individualidade, necessidades específicas e nível de autonomia, e ser prestados através de uma abordagem interdisciplinar e integrada e prioritariamente a nível da comunidade. As pessoas afetadas por doenças mentais não podem ser estigmatizadas ou negativamente discriminadas ou desrespeitadas em contexto de saúde, em virtude desse estado. Base 17: A utilização das tecnologias da saúde deve reforçar a humanização e a dignidade da pessoa. Base 20: O sns pauta a sua atuação pelos seguintes princípios: Qualidade, visando prestações de saúde efetivas, seguras e eficientes, com base na evidência, realizadas de forma humanizada, com correção técnica e atenção à individualidade da pessoa;

**** *id_24 *doc_3 *ano_2019 *autor_2 *área_3

A carta para a participação pública em saúde, doravante designada por carta, pretende fomentar a participação por parte das pessoas, com ou sem doença e seus representantes, nas decisões que afetam a saúde da população, e incentivar a tomada de decisão em saúde assente numa ampla participação pública. A carta pretende ainda promover e consolidar a participação pública a nível político e dos diferentes órgãos e entidades do estado, em Portugal, através do aprofundamento dos processos de participação já existentes e da criação de novos espaços e mecanismos participativos. Desta forma, a carta contribui para: promover e defender os direitos das pessoas com ou sem doença, em especial no que respeita à proteção da saúde, da informação e da participação; Informar as entidades públicas sobre as prioridades, necessidades e preocupações das pessoas com ou sem doença e seus representantes; tornar as políticas de saúde mais eficazes e, consequentemente, obter melhores resultados em saúde; a participação pública em saúde deve assentar nos seguintes princípios: reconhecimento da participação pública como direito das pessoas com ou sem doença e seus representantes; reconhecimento das pessoas com ou sem doença e seus representantes como parceiros nos processos de tomada de decisão; reconhecimento da importância do conhecimento e da experiência específicos da pessoa com ou sem doença; autonomia e independência das pessoas com ou sem doença e seus representantes nos processos; transparência e divulgação pública dos processos participativos; criação das condições necessárias à participação; complementaridade e integração entre instituições e mecanismos da democracia representativa e da democracia participativa. A participação pública deve ainda ser operacionalizada de forma sistemática, através de mecanismos diversos, de forma a ir ao encontro das especificidades de todas as partes interessadas e afetadas e promover uma participação ampla e diversificada, nomeadamente através de: reuniões públicas; audições públicas; consultas públicas; representação em conselhos consultivos, comissões ou grupos de trabalho especializados ou setoriais, no âmbito da política de saúde e políticas relacionadas, tanto a nível nacional como regional e municipal; conselhos da comunidade, junto das diversas entidades e serviços relevantes no âmbito da política de saúde e políticas relacionadas; comissões de utentes; conselhos municipais de saúde; conselho nacional para a participação em saúde; fórum nacional sobre participação em saúde; plataformas digitais para a participação pública em saúde.

**** *id_25 *doc_3 *ano_2020 *autor_2 *área_3

Decorridos mais de 10 anos desde a implementação do modelo de gestão integrada da doença renal crónica importa revalidar o modelo e progredir com uma renovada agenda estratégica, focada na melhor gestão do doente e do seu percurso, desde a prevenção da doença renal crónica até ao seu diagnóstico e abordagem terapêutica, para a melhoria da qualidade e da segurança da prestação de cuidados de saúde ao doente renal crónico, num modelo verdadeiramente integrado, efetivo e centrado na pessoa. Assim, considerando que o atual modelo de governação integrada, cuja intervenção multidisciplinar e intersetorial, prevê responder aos novos desafios demográficos e epidemiológicos da prestação de cuidados de saúde à pessoa com doença renal crónica, determino o seguinte: a comissão nacional de acompanhamento da diálise, tem por missão: promover a inovação centrada na pessoa, com transformação de processos para a efetiva integração de modalidades de diálise, individualização, domiciliação de tratamentos, empowerment do doente e capacitação da equipa clínica; promover a melhoria contínua na gestão do acesso de diálise, da alocação à diálise e da transição de métodos de tratamento, garante de melhores resultados clínicos e mais valor percecionado pelo doente; promover a melhoria contínua dos sistemas de informação de suporte à governação clínica e dos meios tecnológicos de comunicação em saúde; avaliar os ganhos em saúde desde modelo de governação clínica, numa perspetiva de melhoria contínua da qualidade; acompanhar e avaliar a prestação de cuidados de saúde à pessoa com doença renal crónica, designadamente no que diz respeito ao acesso e oferta de cuidados específicos de saúde, à qualidade dos cuidados e segurança dos doentes, ao grau de satisfação dos doentes em diálise crónica, aos modelos de financiamento dos cuidados e aos resultados da prestação de cuidados de saúde; emitir pareceres técnicos e científicos sempre que lhe seja solicitado; acompanhar tecnicamente a aplicação do modelo de gestão integrada da doença renal crónica, que inclui o modelo de pagamento por preço compreensivo.

**** *id_26 *doc_5 *ano_2020 *autor_2 *área_3

Consolidar uma abordagem estratégica e holística de prevenção e intervenção, centrada nas pessoas em situação de sem_abrigo, por forma a que ninguém tenha de permanecer na rua por ausência de alternativas. Princípios: Promoção de uma abordagem centrada nos direitos humanos e na realização da dignidade da pessoa humana; realização dos direitos e deveres de cidadania; promoção da não discriminação e da igualdade, nomeadamente igualdade entre mulheres e homens; promoção do conhecimento reflexivo e atualizado da dimensão e natureza do fenómeno que sustente o desenvolvimento de estratégias de intervenção; promoção do reconhecimento e aprofundamento da multidimensionalidade e complexidade do fenómeno e consequente necessidade de adequação e persistência na implementação de medidas; definição e implementação de medidas de prevenção, intervenção e acompanhamento; corresponsabilização e mobilização do conjunto das entidades públicas e privadas, numa lógica de subsidiariedade, para uma intervenção integrada e consistente, no sentido de garantir a acessibilidade aos serviços, respostas e cuidados existentes; reconhecimento e adequação às especificidades locais e dos diversos grupos que compõem as pessoas em situação de sem_abrigo; reconhecimento e adequação às especificidades de mulheres e de homens; garantia de uma intervenção de qualidade centrada na pessoa, salvaguardando a reserva da sua privacidade, ao longo de todo o processo de apoio e acompanhamento; participação pró_ativa e promoção da capacitação da pessoa em situação de sem_abrigo em todos os níveis do processo de inserção social; educação e mobilização da comunidade; monitorização do processo e avaliação dos resultados de implementação da enipssa 2017_2023. O modelo de intervenção a utilizar na implementação da enipssa 2017_2023 assenta na centralidade da pessoa como um todo e no seu contexto de vida, que se pretende integrado e integral, e visa a prevenção de novas situações, um acompanhamento de proximidade, e assenta numa premissa de qualificação e rentabilização de recursos humanos e financeiros, nomeadamente para evitar a duplicação de respostas. Este modelo implica uma abordagem multidimensional na elaboração do diagnóstico das situações e no acompanhamento dos casos, com desenho de um projeto de vida individual com vista à inserção e autonomização face aos serviços de apoio, sempre que possível, construído na relação entre o utente e o gestor de caso com o qual mantém uma relação privilegiada. Eixo 2: Reforço de uma intervenção promotora da integração das pessoas em situação de sem_abrigo. As medidas incluídas neste eixo, visam o reforço de uma intervenção promotora da integração das pessoas em situação de sem_abrigo de forma a garantir a qualidade, eficácia e eficiência em duas vertentes fundamentais: A intervenção técnica, através da formação dos técnicos e dos dirigentes de respostas sociais e serviços de atendimento dos serviços públicos, com base na adoção de metodologias de intervenção integrada a partir de modelo específico. A implementação de um referencial de formação específica para intervenção com a multidimensionalidade que este problema requer implica que o mesmo seja concebido e dirigido aos diferentes níveis de interventores, não só para os dirigentes e para os profissional_de_saúde que acompanham diretamente as pessoas em situação de sem_abrigo, mas também para aqueles que podem garantir a acessibilidade aos serviços. A metodologia de intervenção e acompanhamento integrado pressupõe a articulação entre os diferentes serviços locais e a promoção e a garantia da eficácia e da eficiência da intervenção, rentabilizando os recursos existentes na comunidade com base na aplicação das medidas e programas existentes das várias áreas de ação de forma integrada e centrada na pessoa em situação de sem_abrigo.

**** *id_28 *doc_3 *ano_2022 *autor_2 *área_3

A recente pandemia da doença covid_19, à qual foram os serviços públicos de saúde que responderam ao desafio de identificar casos, isolar contactos, testar e vacinar pessoas e tratar doentes, sem exceção, reforçou a importância de apostar num sistema_de_saúde forte, que tenha as pessoas no seu centro e como pilar essencial o serviço público de saúde, acessível a todos e tendencialmente gratuito. Atualmente, o sns enfrenta importantes desafios associados à evolução das necessidades em saúde e ao aumento das exigências e expectativas da população. Num quadro de recuperação da pandemia, as propostas para o sns estão enquadradas numa abordagem centrada nas necessidades das pessoas, de forma a proteger e melhorar a sua qualidade_de_vida desde que nascem até ao final da vida. Também com esse objetivo, e considerando a relevância que os recursos humanos assumem no sns, como garante principal da sua qualidade, foi aprovado um programa de gestão estratégica dos recursos humanos do sns, assente numa visão multidimensional, com especial enfoque na valorização dos recursos humanos e no recrutamento planeado dos profissional_de_saúde necessários às exigências da organização das respostas. Visando um sns mais justo e inclusivo que responda melhor às necessidades da população, o Governo irá: melhorar o acesso a consultas e atividades de promoção da saúde e prevenção da doença, através de intervenções multidisciplinares adequadas às características de cada cidadão, nomeadamente de acordo com as estratégias e orientações do plano_nacional_de_saúde 21_30; melhorar a organização e articulação dos serviços de saúde_pública, criando mecanismos de maior integração entre as estruturas do sns, a proteção_civil, o setor social e os atores da sociedade civil com intervenção direta e indireta na saúde; fomentar a utilização da telessaúde como resposta de proximidade às necessidades dos cidadãos e criar um centro nacional de telemedicina e uma rede nacional de telemedicina; otimizar o acesso ao medicamento, o aproximando do utente, e a utilização dos medicamentos ao longo de toda a sua cadeia, garantindo maior eficiência nos processos aquisitivos, reforçando o papel das comissões de farmácia e terapêutica, apoiando os prescritores e incluindo os utentes e seus representantes nas diferentes fases do processo; promover a integração e continuidade de cuidados centrada no utente, através dos sistemas de informação, em especial através da criação do processo clínico eletrónico único, que integre os diferentes níveis de prestação de cuidados e setores, permita o acesso à informação clínica relevante do cidadão em qualquer ponto da rede sns e promova a autonomia do cidadão na gestão do seu processo de saúde; promover projetos de gestão integrada dos percursos dos cidadãos no sns, reforçando a continuidade de cuidados e os mecanismos de integração dos serviços mediante o seguimento dos doentes com doenças crónicas; reforçar a autonomia na gestão hospitalar, nomeadamente em matéria de contratação de profissional_de_saúde, com maior responsabilização e avaliação da satisfação pelos utentes e profissional_de_saúde;

**** *id_31 *doc_3 *ano_2023 *autor_2 *área_1

Os cuidados de longa duração compreendem uma gama de serviços e atividades assistenciais, combinados e integrados, que adotam uma visão holística do processo de envelhecimento e da proteção da dependência, através dos quais se conjugam não só as necessidades mas também os cuidados prestados pelos diversos atores, de acordo com a capacidade funcional da pessoa que é o foco dos cuidados e com a capacidade dos cuidadores, formais e informais. Os cuidados previstos no número anterior são uma resposta de base comunitária e devem compreender: a reinserção para preservar ou recriar a rede relacional de pertença; o reabilitar e ou readaptar a autonomia e a independência; o manter e retardar a perda de autonomia e de independência; o promover o bem_estar e a qualidade_de_vida. Os serviços de cuidados especializados integrados podem ser acoplados às erpi, existentes ou futuras, e às unidades de cuidados continuados integrados de longa duração e manutenção integradas na rede, aumentando o seu significado social e de saúde. Constitui objetivo geral dos cuidados de longa duração da Região Autónoma da Madeira assegurar os melhores cuidados às pessoas, com eficácia e eficiência em termos de adequação e integração, podendo ser adaptados e direcionados, a qualquer momento, para as necessidades funcionais que decorrem do processo de envelhecimento ou da perda progressiva da autonomia. Os cuidados de longa duração são desenvolvidos de acordo com os seguintes princípios orientadores: Os cuidados de longa duração da Região Autónoma da Madeira respeitam a dignidade e outros direitos e liberdades fundamentais das pessoas com necessidade de cuidados de longa duração, as suas famílias e os seus cuidadores; Os cuidados de longa duração da Região Autónoma da Madeira visam restaurar, tanto quanto possível, ou prevenir, a deterioração do estado físico e ou saúde mental das pessoas que necessitam de cuidados de longa duração e fortalecer a sua capacidade de viver de forma independente, ao mesmo tempo que aliviam a sua experiência de solidão ou isolamento social; centralização na pessoa: Os serviços de cuidados de longa duração da Região Autónoma da Madeira são prestados sem qualquer discriminação e abordam as necessidades específicas de cada indivíduo, devendo respeitar a integridade da pessoa e ter em consideração o seu género e a sua condição física, diversidade intelectual, cultural, étnica, religiosa, linguística e social e, quando apropriado, o das suas famílias ou do seu círculo social imediato; Compreensividade e continuidade: Os cuidados de longa duração da Região Autónoma da Madeira são concebidos e prestados de forma integrada com todos os outros serviços, incluindo cuidados de saúde e telessaúde, com uma coordenação eficaz entre os níveis regional e local, sendo organizados para que as pessoas que necessitam dos mesmos possam contar com uma gama ininterrupta de serviços, quando necessário e durante o tempo que for indispensável, devendo as transições entre diferentes serviços de cuidados prolongados ser suaves e evitar interrupção do serviço ou qualquer impacto negativo no atendimento recebido; Foco nos resultados: Os cuidados de longa duração da Região Autónoma da Madeira se centram, sobretudo, nos benefícios para quem recebe os cuidados, em termos da sua qualidade_de_vida e capacidade de viver de forma independente, levando em consideração, quando apropriado, os benefícios para as suas famílias, cuidadores informais e comunidade; Transparência: As informações sobre as opções e os provedores de cuidados de longa duração da Região Autónoma da Madeira disponíveis, qualidade, padrões e acordos de garantia de qualidade devem ser fornecidas na íntegra, de forma acessível e compreensível, para as pessoas que necessitam de cuidados especializados e prolongados, suas famílias ou cuidadores; Qualificação da prestação: Os cuidados de longa duração da Região Autónoma da Madeira são prestados por trabalhadores qualificados, e são respeitadas em proporções apropriadas de trabalhadores, de acordo com os respetivos rácios, refletindo o número e as necessidades das pessoas que vão receber aqueles cuidados, nos diferentes contextos onde os cuidados são prestados, devendo ser respeitados os direitos dos trabalhadores, a confidencialidade, a ética profissional e a autonomia profissional e disponibilizada formação contínua a todos os profissional_de_saúde envolvidos.

**** *id_32 *doc_3 *ano_2023 *autor_2 *área_3

O aumento das necessidades em saúde e bem_estar da população, associados ao envelhecimento, à carga de doença, assim como às suas crescentes exigências e expectativas, exige que o sns continue a aumentar o acesso e a eficiência na prestação de cuidados de saúde, fomentando modelos organizacionais que promovam a gestão integrada de cuidados de saúde primários e cuidados hospitalares, assegurando o foco nas pessoas. Esta alteração visa a prestação integrada de cuidados de saúde primários e hospitalares, o reforço dos cuidados primários na resposta de proximidade e continuidade na assistência em saúde e a aposta na promoção da saúde. Se refira que o amadurecimento deste modelo organizativo permite que as unidades locais de saúde beneficiem de novos instrumentos de gestão, designadamente: uma estratificação pelo risco, que identifique a distribuição da carga de doença na população; sistemas de informação que potenciem a integração de cuidados, como o registo_de_saúde_eletrónico único; incentivos ao desempenho, financeiros e não financeiros, focados nos resultados e na criação de valor; e modelos inovadores de prestação de cuidados, baseados em equipas que assumem compromissos centrados na resposta às pessoas, com destaque para as unidades de saúde familiar e as unidades de cuidados na comunidade, no âmbito dos cuidados primários, ou para área hospitalar, os centros de responsabilidade integrados. A integração dos agrupamento de centros de saúde, hospitais e centros hospitalares já existentes no modelo da unidade local de saúde constitui uma qualificação da resposta do sns, simplificando os processos, incrementando a articulação entre equipas de profissional_de_saúde, com o foco na experiência e nos percursos entre os diferentes níveis de cuidados, aumentando a autonomia gestionária, melhorando a participação dos cidadãos, das comunidades, dos profissional_de_saúde e das autarquias na definição, acompanhamento e avaliação das políticas de saúde, maximizando o acesso e a eficiência do sns.

**** *id_33 *doc_5 *ano_2023 *autor_2 *área_1

O 23 Governo Constitucional assumiu no seu programa o compromisso de um sns mais justo e inclusivo, que responda melhor às necessidades da população, reforçando a importância da manutenção de um sistema_de_saúde forte, com uma abordagem centrada nas necessidades das pessoas, de forma a proteger e melhorar a sua qualidade_de_vida desde que nascem até ao final da vida. A gestão integrada dos percursos dos cidadãos no sns, reforçando a continuidade de cuidados e os mecanismos de integração dos serviços, tem um papel essencial na centralidade das pessoas. A Agenda de Desenvolvimento Sustentável das Nações Unidas para 2030 apresentou, como um dos objetivos, diminuir o impacto das doenças não transmissíveis nos indivíduos e na sociedade, nomeadamente reduzir a mortalidade prematura por doenças não transmissíveis em um terço até 2030, para o qual é essencial fornecer uma resposta integrada que se concentre na promoção da saúde e prevenção de doenças em setores relevantes, que a doença renal crónica tipifica. Considerando a evidência científica e as melhores práticas, se torna necessário implementar planos de cuidados preventivos e terapêuticos que viabilizem maior personalização, integração e proximidade, maior uso de tratamentos domiciliários, maior acesso a transplantação renal, melhor gestão de informação, oportuno uso de saúde digital, bem como instrumentos jurídicos de financiamento e contratualização de produção e qualidade, alinhados com uma visão de serviço centrada no cidadão. Com vista a promover a inovação centrada na pessoa, com transformação de processos para a efetiva integração e individualização de resposta a pessoas com insuficiência renal crónica, a estratégia é definida de acordo com os seguintes 5 eixos: prevenção e acesso; personalização e segurança na prestação de cuidados; integração de cuidados; gestão de informação e avaliação de resultados; comunicação em saúde. Na personalização e segurança na prestação de cuidados, com vista a garantir um modelo assistencial centrado na pessoa com doença renal crónica, é necessário promover a individualização terapêutica, com efetiva gestão da qualidade dos processos de opção e consentimento do doente para a modalidade de tratamento, a par de um circuito cirúrgico seguro para a realização dos acessos vascular e peritoneal para diálise, e de segurança e personalização, tanto na indução de diálise, como na transição entre modalidades de tratamento, sempre ajustados aos objetivos e ao plano terapêutico individualizado. Importa, também, diferenciar a oferta terapêutica à luz da mais recente informação científica e da criação de valor em saúde, promovendo um maior desenvolvimento de programas de diálise domiciliária e transplantação de dador vivo. A integração de cuidados, para a sustentabilidade da prestação de cuidados na doença renal crónica, e da sua qualidade, é necessário implementar linhas de qualidade organizacional e assistencial promotoras de integração de cuidados. O modelo assistencial deve atender a um grupo de variáveis que, inequivocamente, sendo basilares na integração de cuidados, condicionem a qualidade do percurso do doente: referenciação para cuidados hospitalares, acompanhamento dos doentes após alta hospitalar, internamentos evitáveis, urgências evitáveis, telemedicina, plano_individual_de_cuidados, processo_assistencial_integrado, referenciação para cuidados continuados, referenciação para cuidados paliativos, referenciação para cuidados de saúde mental, referenciação para cuidados de medicina física de reabilitação, cuidados em rede, apoio no fim de vida, consultas hospitalares descentralizadas e consultas domiciliárias. Para melhor gestão de transição de modalidades de tratamento no circuito do doente em diálise crónica, com otimização de recursos, são desejáveis unidades integradas de diálise, que ofereçam planos de tratamento individualizados, integrando hemodiálise em centro, hemodiálise domiciliária, diálise peritoneal, tratamentos assistidos em fases de perda de autonomia e tratamento conservador não dialítico em doentes eletivos, deste modo promovendo percursos que melhoram a qualidade dos cuidados prestados e a resposta às necessidades dos doentes e famílias. Gestão de informação e avaliação de resultados: A recolha e gestão de dados em saúde são requisitos cruciais para a qualidade da governação e adequada tomada de decisão nos sistema_de_saúde. A gestão de informação deve suportar modelos de financiamento baseados na qualidade e no valor em saúde, alinhados com o reconhecimento da relevância do ecossistema nacional de saúde para a economia da saúde, no setor da doença renal crónica, num modelo de cuidados integrados centrado na pessoa. A estratégia define objetivos e metas para o triénio que potenciam a melhoria da qualidade clínica, a eficiência da prestação e a centralidade no doente no percurso de cuidados nas unidades de saúde, correspondentes aos eixos estratégicos. Objetivo: personalização e segurança na prestação de cuidados: promover a melhoria da qualidade assistencial nos serviços hospitalares de nefrologia com vista a aumentar a sobrevivência livre de diálise, diminuir o número de internamentos e diminuir a indução não planeada de diálise na trajetória da doença renal crónica avançada. Assegurar o acesso a modalidades de tratamento substitutivo renal crónico. Implementar a gestão dos processos de opção, acesso e indução de tratamento substitutivo renal crónico, com foco na melhoria dos resultados clínicos e na promoção, suportada pela evidência, de transplantação renal e diálise domiciliária. Definir planos de tratamento e individualização terapêutica, no seio de um processo de decisão partilhada. Integração de cuidados: Promover, no âmbito do sns, a integração dos cuidados de saúde na doença renal crónica e a melhoria contínua dos processos, focada na evicção de internamentos e de eventos adversos. Reforçar a hospitalização domiciliária no tratamento de pessoas com doença renal crónica. Promover unidades integradas de tratamento dialítico com oferta de hemodiálise, diálise peritoneal e tratamento conservador não dialítico, em doentes eletivos. Promover a capacitação dos doentes com recurso a serviços de proximidade e de diálise domiciliária, incluindo tratamentos assistidos em fases de perda de autonomia e fim de vida. Promover a continuação de cuidados e a gestão de transição de modalidades de tratamento, no circuito do doente com otimização de recursos. Potenciar os cuidados de saúde primários, o programa de saúde mental do sns, a rede nacional de cuidados continuados integrados e a rede de cuidados paliativos do sns, no âmbito dos tratamentos da doença renal crónica e na gestão do fim de vida. Gestão de informação e avaliação de resultados: Garantir o conhecimento da epidemiologia da doença renal crónica e estratificação de risco. Promover o uso de telemonitorização na prestação de serviços a doentes com insuficiência renal avançada e em tratamento domiciliário, com vista a mais proximidade e eficiência. Investir na interoperacionalidade digital na gestão de informação na doença renal crónica. Integrar medidas de resultados reportados pelos doentes nas avaliações da qualidade e do custo_benefício dos tratamentos. Contratualizar a produção e qualidade no setor da doença renal crónica, no âmbito dos cuidados de saúde primários, hospitalares e de unidades de diálise. Rever o modelo de financiamento das unidades de diálise e o ajustar à diferenciação e integração de cuidados. Obter o reconhecimento da relevância do ecossistema nacional de saúde para a economia da saúde e das populações, no setor da doença renal crónica. Comunicação em saúde: Implementar medidas de gestão de risco de lesão renal aguda e doença renal crónica nos cuidados de saúde primários, com campanha de informação e alertas. Promover formação contínua dos clínicos que intervêm no circuito do doente com doença renal crónica. Usar os meios de comunicação digital com informação validada pelas sociedades científicas dirigida a doentes e a cidadãos em geral. Garantir a qualidade do processo de opção de modalidades de substituição renal na doença renal crónica avançada.

**** *id_34 *doc_3 *ano_2023 *autor_2 *área_3

Visando um sns mais justo e inclusivo que responda melhor às necessidades da população, o Governo prosseguirá a sua ação no sentido de: Melhorar o acesso a consultas e atividades de promoção da saúde e prevenção da doença, através de intervenções multidisciplinares adequadas às características de cada cidadão, nomeadamente de acordo com as estratégias e orientações do plano_nacional_de_saúde 21_30. Criar uma estrutura nacional para a promoção da saúde. Melhorar a organização e articulação dos serviços de saúde_pública, criando mecanismos de maior integração entre as estruturas do sns, a proteção_civil, o setor social e os atores da sociedade civil com intervenção direta e indireta na saúde. Aprovar um novo modelo de organização das respostas aos comportamentos_aditivos_e_dependências, mediante a integração das competências de planeamento, coordenação e intervenção. Fomentar a utilização da telessaúde como resposta de proximidade às necessidades dos cidadãos, criar um centro nacional de telemedicina e uma rede nacional de telemedicina bem como promover a utilização do centro de contacto sns_24 como porta de entrada e referenciação no sns. Promover a integração e continuidade de cuidados centrada no utente, através dos sistemas de informação, em especial através da criação do processo clínico eletrónico único, que integre os diferentes níveis de prestação de cuidados e setores, permita o acesso à informação clínica relevante do cidadão em qualquer ponto da rede sns e promova a autonomia do cidadão na gestão do seu processo de saúde. Reforçar as unidades de cuidados na comunidade, pela sua relevância no trabalho de apoio às pessoas mais vulneráveis, no domicílio e na comunidade.

**** *id_36 *doc_5 *ano_2023 *autor_2 *área_3

O âmbito de abrangência do Plano Nacional para a Redução dos comportamentos_aditivos_e_dependências 2030 são os comportamentos_aditivos_e_dependências, tendo em conta os novos desafios que se colocam nesta área e também o princípio base deste plano, que é a centralidade no cidadão. Estão incluídos no conceito de comportamentos_aditivos_e_dependências: o uso de substâncias psicoativas ilícitas e de novas substâncias psicoativas; o uso de substâncias psicoativas lícitas, como o álcool, o tabaco ou os medicamentos; padrões comportamentais excessivos associados a práticas de jogo, a dinheiro ou não; o uso problemático de internet; e outros comportamentos potencialmente aditivos, que possam ter a possibilidade de virem a ser consagrados como tal nas classificações de doenças, atendendo à evolução dos consumos e adições comportamentais verificadas. A política nacional em matéria de intervenção nos comportamentos_aditivos_e_dependências tem sido caracterizada pelos princípios do humanismo e pragmatismo como base de todas as orientações, e pela qualidade da intervenção desenvolvida pelas instituições públicas e privadas no âmbito dos comportamentos_aditivos_e_dependências. Considerando os direitos do cidadão, nomeadamente, o acesso a informação e serviços especializados e adequados a cada situação, a abordagem é desenvolvida com especial enfoque na centralidade do cidadão, garantindo ainda os princípios de igualdade e equidade. Reforçar a literacia em comportamentos_aditivos_e_dependências, dotando o cidadão de informação, conhecimento e competências com vista a tomar decisões informadas e gerar comportamentos saudáveis e seguros. A promoção da literacia em comportamentos_aditivos_e_dependências é determinante para que o cidadão esteja capacitado a tomar decisões adequadas em saúde, proporcionando uma utilização mais eficiente e racional dos recursos do sistema_de_saúde. A literacia em comportamentos_aditivos_e_dependências deve se constituir como uma prioridade na sociedade atual, na medida em que contribui para a mudança de comportamentos e atitudes e para o desenvolvimento saudável e seguro das crianças, dos jovens e jovens adultos, e consequentemente para uma diminuição do consumo de substâncias psicoativas lícitas e ilícitas. Promover a inclusão social e a capacitação para o exercício da cidadania informada das populações mais vulneráveis com comportamentos_aditivos_e_dependências. Os cidadãos com problemas de comportamentos_aditivos_e_dependências se encontram frequentemente em situação de exclusão social, de marcado isolamento e de rejeição social face ao meio onde se inserem. Na promoção da sua inclusão social é fundamental a existência de interações positivas com a sociedade, o que implica o estabelecimento de ligações entre as pessoas e o seu contexto social, de forma a criar ou reparar os laços sociais entre os cidadãos e os sistemas sociais. Ao longo dos últimos anos se têm preconizado modelos de intervenção integrados, centrados nas necessidades reais do cidadão que se encontra em risco de desinserção. Criar condições do ambiente externo para promover comportamentos de menor risco ou mais saudáveis. As iniciativas em literacia e uma cultura inclusiva e de diversidade ao nível da envolvente externa são um complemento imprescindível para as intervenções mais centradas no cidadão, contribuindo decisivamente para o remover de barreiras e para a promoção do acesso a uma verdadeira cidadania, com plena incorporação dos direitos humanos.

**** *id_37 *doc_3 *ano_2024 *autor_2 *área_3

A enipssa 2017_2023, criada pela Resolução do Conselho de Ministros número 107.2017, visava consolidar uma abordagem estratégica e holística de prevenção e intervenção, centrada nas pessoas em situação de sem_abrigo, por forma a que ninguém tenha de permanecer na rua por ausência de alternativas. A enipssa, que seguidamente se apresenta, pretende introduzir um conjunto de melhorias face aos ciclos programáticos anteriores, muito especialmente no que diz respeito a: dar enfoque às medidas de prevenção, dado que este domínio da prevenção do fenómeno é tão importante como a forma como intervimos coletivamente caso esta situação venha a ocorrer; avançar nas mudanças já iniciadas, evitando ruturas na implementação de uma política abrangente de prevenção e combate a este fenómeno, nomeadamente na adoção de um modelo centrado nas pessoas, garantindo o alargamento, a criação e a inovação de serviços de base comunitárias, a identificação e proliferação de ações de sensibilização, de ideias inovadoras e de partilha de práticas inspiradoras; reforçar, diversificar e atualizar as redes territoriais de apoio às pessoas em situação de risco ou que vivenciam a condição de sem_abrigo, através dos núcleos de planeamento e intervenção sem_abrigo, redimensionando a sua intervenção à tónica da prevenção, garantindo a sua estreita relação com outros serviços e equipas existentes no terreno; garantir uma intervenção especializada junto de públicos especialmente vulneráveis como população idosa, em situação de dependência, com deficiência, pessoas com necessidade de cuidados de saúde mental, pessoas com consumos e dependência do álcool ou substâncias ilícitas, pessoas lgbti_mais, população cigana, população migrante, entre outros; promover a assunção das responsabilidade e competências coletivas, intersetoriais, incluindo as relacionadas com o domínio preventivo, garantindo um acompanhamento multinível atento da execução da enipssa, o conhecimento da dinâmica social e o reforço ou atualização contínua das medidas e a sua interrelação, bem como a partilha efetiva de recursos; Redefinir, reforçar e desburocratizar o modelo de governação que permita um acompanhamento efetivo da enipssa e uma implementação progressiva e flexível; Melhorar os mecanismos e instrumentos de monitorização e avaliação e o desenvolvimento de metodologias que permitam um reporte contínuo e transparente da execução das medidas. Visão: Consolidar uma abordagem estratégica, integrada, multidisciplinar e holística de prevenção e intervenção face ao fenómeno, garantindo uma atuação eficiente e eficaz, centrada nas pessoas, por forma a que ninguém venha a vivenciar a situação de sem_abrigo, e muito menos permanecer na rua por ausência de alternativas. Assegurar que a população tem acesso a medidas de apoio e serviços habilitados a promover a prevenção da condição de sem_abrigo ou, sempre que ocorra, a um apoio integrado nas diferentes dimensões biopsicossocial e ambiental, para que, através de cuidados de qualidade, se alcance a sua inclusão social. Missão: Criar e implementar um ecossistema próximo das pessoas em situação de risco ou que vivenciam a condição de sem_abrigo, garantido o acesso equitativo, em quantidade e qualidade, a serviços de apoio, através de respostas territorializadas e de uma intervenção personalizada. Objetivo geral e princípios: Prevenção multinível do fenómeno, promovendo a definição e adoção de medidas preventivas e de intervenção junto de pessoas em situação de risco ou que vivenciam a condição de sem_abrigo, com vista à realização de uma intervenção precoce para evitar ou minimizar o risco e os seus efeitos; Desenvolvimento de uma abordagem centrada na pessoa, nos direitos humanos e na realização da autodeterminação e na dignidade da pessoa humana; Participação ativa das pessoas em situação de risco ou que vivenciam a condição de sem_abrigo em todo o processo de planeamento, intervenção e avaliação; Garantia dos direitos e deveres de cidadania; Promoção da não discriminação e da igualdade, nomeadamente a salvaguarda da identidade de género e o combate à discriminação em razão da orientação sexual, da identidade e expressão de género, e de características sexuais; Reforço de estratégias de conhecimento reflexivo, atualizado e inovador da dimensão e natureza do fenómeno que suporte o desenvolvimento de estratégias de prevenção e intervenção sustentáveis; Promoção do reconhecimento e aprofundamento da multidimensionalidade e complexidade do fenómeno e consequente necessidade de adequação e persistência na implementação de medidas; Definição, implementação e atualização permanente de medidas de prevenção, combate, intervenção e acompanhamento, garantindo a sua equidade; corresponsabilização e mobilização do conjunto de recursos e de atores, nomeadamente entidades públicas e privadas da sociedade civil, numa lógica de complementaridade e subsidiariedade, para uma prevenção e intervenção integrada e integral, consistente e efetiva, no sentido de garantir acessibilidade aos recursos, serviços, respostas e cuidados existentes, incluindo garantir uma distribuição racional e sustentável dos recursos e o combate ao desperdício, designadamente alimentar; Reconhecimento e adequação às especificidades e heterogeneidade dos diversos grupos que compõem as pessoas em situação de risco ou que vivenciam a condição de sem_abrigo, incluindo em função do género, do sexo, da deficiência e da vulnerabilidade; Garantia de uma prevenção e intervenção de qualidade centrada na pessoa, salvaguardando a reserva da sua privacidade, ao longo de todo o processo de apoio e acompanhamento, personalizando a intervenção e garantindo a oferta de soluções territorializadas, de acordo com as necessidades e potencialidades de cada pessoa; Participação pró_ativa e promoção da capacitação das pessoas em situação de risco ou que vivenciam a condição de sem_abrigo em todos os níveis do processo de inclusão social; Priorizar uma orientação para habitação, centrada na facilitação do acesso a soluções estáveis, preferencialmente definitivas, e a sua adequação ao projeto de vida da pessoa; Garantir uma desinstitucionalização planeada, promovendo processos de transição para a vida comunitária e a autodeterminação das pessoas aquando da saída de instituições, incluindo de estabelecimentos prisionais, unidades de saúde, casas de acolhimento e outras; Sensibilização, educação e mobilização da comunidade para o fenómeno; Monitorização contínua do processo e avaliação dos resultados de implementação da enipssa. O modelo proposto assenta, assim, na centralidade da pessoa como um todo e no seu contexto de vida, que se pretende integrado e integral, e visa a prevenção de novas situações, um acompanhamento de proximidade, se baseando numa premissa de qualificação e rentabilização de recursos humanos e financeiros, nomeadamente para evitar a duplicação de respostas.

**** *id_38 *doc_5 *ano_2024 *autor_2 *área_3

O Plano de Ação do Envelhecimento Ativo e Saudável constitui o guia e será o impulso da transformação na sociedade portuguesa, com o propósito de garantir as melhores condições para todos, visando maximizar a sua longevidade e qualidade_de_vida, sem deixar ninguém para trás e com foco nas pessoas. Subpilar cuidados integrados e de longa duração: se destacam medidas destinadas a facilitar o acesso aos cuidados recorrendo às novas tecnologias e linhas de apoio, como são exemplo a linha sns_24 e os balcões sns_24, além da criação de novas respostas como a linha 60_mais, para responder a necessidades específicas dos cidadãos seniores, e os gestores 60_mais. Se pretende tomar medidas concretas na melhoria da qualidade dos serviços prestados com foco no utente, incluindo algumas inovações para esta estrutura em rede com inclusão dos disfibrilhadores automáticos externos, o treino e a capacitação dos profissional_de_saúde, a melhoria das condições de trabalho e mais formação e diferenciação das equipas.

**** *id_40 *doc_5 *ano_2024 *autor_2 *área_3

Sendo assim, a experiência adquirida durante a pandemia demonstrou a importância da integração de cuidados, da necessidade de articulação, coordenação e colaboração entre os diferentes stakeholders do serviço regional de saúde e da sociedade, em especial do setor social. Comparando com 2020, verificamos que é possível uma articulação mais próxima dos serviços, uma articulação centrada no utente. A melhoria de algumas respostas digitais, tais como o recurso à linha de saúde açores, à telemedicina, a emissão digital do isolamento profilático, o sistema de notificação de casos positivos, entre outros, abriu as portas para a unificação do processo clínico do utente, para a melhoria da vigilância epidemiológica das doenças transmissíveis e ainda para o diagnóstico de situação da saúde dos açorianos, podendo se perspetivar a possibilidade de efetuar um planeamento em saúde que responda efetivamente às necessidades em saúde dos cidadãos e não seja centrado nos serviços de saúde. O modelo concetual do Plano Regional de Saúde 2030 é inspirado na Myosotis maritima, planta endémica dos Açores encontrada nas rochas e falésias marítimas das nossas ilhas. No centro está a pessoa, a família e a comunidade. Se pretende que todo o planeamento e ação se foque nesta tríade e nas suas necessidades concretas. Daí decorre que o primeiro passo para a melhoria da saúde dos açorianos é a identificação das necessidades concretas de cada pessoa. Apenas com esta identificação poderá se planear adequadamente o que lhe oferecer, satisfazendo as suas necessidades com uma utilização eficiente e eficaz dos recursos. Para isso é importante perguntar O que importa para si? e não O que se passa consigo? É também importante que as necessidades desta tríade central sejam o foco de atenção, não só do setor da saúde, mas também de toda a sociedade, na perspetiva de que a saúde depende de uma complexa interação de fatores. A camada imediatamente seguinte é da integração de cuidados. Na atual complexidade dos sistema_de_saúde e das circunstâncias individuais de saúde e doença, também elas cada vez mais complexas, é imperioso atuar de forma integrada entre todos os intervenientes, permitindo um continuum de cuidados, no qual o utente se sinta orientado, seguro e confiante de que todo o sistema trabalha e se organiza em torno das suas necessidades. Para o sistema_de_saúde a integração de cuidados permite que cada profissional_de_saúde se sinta valorizado e integrado numa cadeia de valor, da qual resultam melhores cuidados de saúde. É possível obter ganhos de eficiência ao eliminar duplicações ou intervenções desnecessárias. É também possível aumentar a segurança da prestação de cuidados pela integração da informação, permitindo decisões mais informadas e apoiadas. Pelo impacto negativo causado na sociedade açoriana pelos comportamentos_aditivos_e_dependências foi definido como estratégica a existência de um Plano Regional para a Redução dos comportamentos_aditivos_e_dependências. Este plano tem como visão consolidar e aprofundar uma política pública integrada e eficaz no âmbito da Prevenção dos comportamentos_aditivos_e_dependências, com base numa articulação intersetorial, visando ganhos em saúde e bem_estar na sociedade. Tem como princípios orientadores a centralidade no cidadão, o humanismo e pragmatismo, a intervenção integrada e o conhecimento e inovação. Recorre a diferentes estratégias de intervenção, universal, seletiva, indicada, global, específica e dirigida, se organizando ao longo do ciclo de vida. O plano local de saúde tem como missão: Ser um mecanismo de operacionalização das estratégias regionais de saúde, nomeadamente no que concerne às intervenções prioritárias consideradas em cada programa elegível localmente; Identificar os problemas e necessidades de saúde locais da população; Ser um quadro de referência para as políticas de saúde da comunidade, adotando as estratégias adequadas às necessidades de saúde da população; Promover e consolidar a colaboração das distintas entidades locais de saúde; Integrar e articular os esforços dos parceiros em torno de objetivos comuns, reforçando uma abordagem intersetorial e de saúde em todas as políticas; Se assumir como compromisso social, incentivando os cidadãos a serem coprodutores das políticas de saúde; Permitir a monitorização e avaliação do plano regional de saúde, como um todo. No contexto atual, a promoção da literacia em saúde ganha redobrada importância junto das pessoas, das comunidades, das organizações e dos profissional_de_saúde, se constituindo como uma resposta necessária e uma ferramenta da saúde_pública. Assim, a literacia em saúde constitui neste plano um desígnio e uma oportunidade de promover a saúde ao longo do ciclo de vida, se operacionalizando em programa próprio, com a visão de capacitar e responsabilizar cada indivíduo pelo seu percurso de saúde.

**** *id_46 *doc_3 *ano_2021 *autor_1 *área_1

A relação interpessoal entre um enfermeiro e uma pessoa ou grupo de pessoas, família ou comunidades, constitui o cerne do exercício profissional. Neste contexto, quer a pessoa enfermeiro, quer a pessoa cliente, possuem quadros de valores, crenças e desejos da natureza individual que os tornam seres únicos e cada interação singular e própria. A pessoa, cliente dos cuidados, é entendida como um agente intencional com comportamentos baseados nos valores, nas crenças e nos desejos da natureza individual, com dignidade própria e direito a se autodeterminar. No âmbito dos cuidados especializados em enfermagem de saúde materna e obstétrica, a pessoa cliente que de imediato se destaca é a mulher. No regulamento das Competências Específicas do Enfermeiro Especialista em Enfermagem de Saúde Materna e Obstétrica, é defendido que os cuidados de enfermagem tomam por foco de atenção a promoção dos projetos de saúde que cada mulher, sendo esta entendida numa perspetiva individual e numa perspetiva coletiva. A mulher é, então, entendida como a pessoa no seu todo, considerando a interação com os conviventes significativos e com o ambiente no qual vive e se desenvolve. O ambiente é constituído pelos elementos humanos, físicos, políticos, económicos, culturais e organizacionais que influenciam a saúde. Enquanto cliente dos cuidados do enfermeiro especialista em enfermagem de saúde materna e obstétrica, a mulher pode assumir diferentes especificidades em termos de necessidades em cuidados: mulher grávida, mulher parturiente, mulher mãe, mulher puérpera, mulher adolescente ou adulta, em processo de envelhecimento. Neste contexto, a mulher influencia e é influenciada, quer pelo ambiente onde cresce e desenvolve, quer pelas pessoas com quem interage, quer pela sociedade onde se integra o respeito pelas capacidades, crenças, valores e desejos da natureza individual da mulher e das pessoas que lhe são significativas; o respeito pelas expectativas relacionadas com o trabalho de parto e nascimento do filho; o respeito pelas expectativas e desejos de natureza individual relacionadas com o projeto de maternidade e paternidade; o estabelecimento de parcerias com o cliente no planeamento do processo de cuidados; o estabelecimento de uma relação terapêutica com a mulher e as pessoas que lhe são significativas, tendo por referências as competências de comunicação clínica. Ao longo do período de gravidez, o homem_pai é também entendido como cliente dos cuidados, considerado individualmente, também ele com valores, crenças e desejos, com dignidade própria e direito a se autodeterminar. Neste contexto, podemos, ainda, considerar o casal grávido como cliente dos cuidados, enquanto um conjunto de duas pessoas, com necessidades próprias e individuais, mas que partilham o projeto de parentalidade e conjugalidade. Durante o período pós_parto, a puérpera e a mãe constituem duas vertentes relevantes de necessidades de cuidados centrados na mulher. Neste período também emergem como clientes dos cuidados o recém_nascido e o homem_pai e ou companheiro. Os cuidados de enfermagem visam a promoção da consecução do projeto de saúde de cada pessoa cliente. A relação terapêutica entre o enfermeiro especialista em enfermagem de saúde materna e obstétrica e a cliente é um dos elementos centrais para que a cliente seja pró_ativa na promoção da sua saúde, na prevenção da doença e na adaptação às novas circunstâncias de vida. Um outro elemento_chave é o domínio de competências para responder às necessidades específicas de cada cliente, em cada momento e contexto de cuidados. Na procura permanente da excelência no exercício profissional, o enfermeiro especialista em enfermagem de saúde materna e obstétrica ajuda as clientes a alcançar o máximo potencial de saúde, constituindo elementos relevantes face à promoção da saúde, entre outros: a avaliação e o diagnóstico das necessidades em cuidados específicas das clientes; a identificação dos recursos de saúde e sociais disponíveis. O enfermeiro especialista em enfermagem de saúde materna e obstétrica é aquele que demonstra níveis elevados de julgamento clínico e de tomada de decisão, traduzidos num conjunto de competências específicas em Enfermagem de saúde materna e obstétrica e que assume a responsabilidade pelo diagnóstico diferencial e pela implementação das intervenções, no âmbito dos seguintes domínios o estabelecimento de parceria com a pessoa significativa como aliado no planeamento de cuidados centrado na cliente; A promoção da saúde visa a capacitação e o controlo de cada pessoa para atuar na melhoria da sua qualidade_de_vida e saúde. O modelo da assistência pré_natal é centrado na mulher grávida, na pessoa com quem partilha o projeto de maternidade e na família. Se assumem como princípios gerais que orientam a conceção de cuidados especializados na assistência pré_natal centrados na grávida e na pessoa com quem partilha o projeto de maternidade e família. A comunicação é a base da relação terapêutica, a comunicação clínica entre enfermeiro especialista em enfermagem de saúde materna e obstétrica e cliente é essencial, devendo sempre ser tratada com gentileza, respeito e dignidade. As opiniões, crenças e valores relacionados com ao seu autocuidado e cuidado ao seu filho devem ser identificados e respeitados a cada momento. A mulher deve ter a oportunidade de tomar decisões informadas, em parceria com o enfermeiro especialista em enfermagem de saúde materna e obstétrica, em relação aos cuidados prescritos, apoiadas por informações baseadas em evidências e adaptadas às necessidades específicas. Padrões de qualidade dos cuidados especializados em enfermagem de saúde materna e obstétrica: o respeito pelas capacidades, crenças, valores e desejos da natureza individual da mulher e das pessoas que lhe são significativas; o respeito pelas expectativas relacionadas com o trabalho de parto e nascimento do filho; o respeito pelas expectativas e desejos de natureza individual relacionadas com o projeto de maternidade ou paternidade; o empenho do enfermeiro especialista em enfermagem de saúde materna e obstétrica na capacitação da cliente para a tomada de decisão e para a ação; o empenho do enfermeiro especialista em enfermagem de saúde materna e obstétrica no estabelecimento de parcerias com a cliente e pessoa significativa na capacitação para a tomada de decisão e para a ação; o envolvimento da pessoa significativa no processo de cuidados; o empenho do enfermeiro especialista em enfermagem de saúde materna e obstétrica em considerar no processo de cuidados as necessidades específicas da pessoa significativa enquanto cliente dos cuidados; o estabelecimento de parceria com a pessoa significativa como aliado no planeamento de cuidados centrado na cliente; aproveitar todas as oportunidades para fornecer à mulher, pessoa com quem partilha o projeto de maternidade e família as informações e o apoio de que precisam. O cuidado deve ser centrado na mulher: o foco do cuidado deve ser atender às necessidades da mulher e de seu filho; cada mulher deve negociar o nível de envolvimento que deseja por parte da pessoa significativa e familiares ou amigos. Consulta de enfermagem pré_natal é definida como contexto de cuidados, concebidos e implementados por enfermeiro especialista em enfermagem de saúde materna e obstétrica, centrada nas necessidades da grávida e da pessoa com quem partilha o projeto de maternidade e família. Em cada consulta importa definir, em parceria, um plano de cuidados individualizado, que promova a vivência saudável da gravidez, facilite a transição para a parentalidade e favoreça a participação ativa do casal grávido em todo o processo.

**** *id_48 *doc_4 *ano_2022 *autor_3 *área_1

Assumindo a proteção da pessoa idosa e as suas necessidades de cuidados como elemento central desta vertente de um sistema que congrega a proteção social e a proteção na saúde de uma população particularmente vulnerável, o presente referencial pretende contribuir para implementar, em cada erpi, uma abordagem vocacionada para a recuperação e manutenção dos melhores níveis de saúde possíveis de cada pessoa idosa, bem como para o desenvolvimento de uma cultura de aprendizagem nesta área. A saúde envolve, simultaneamente, diferentes dimensões: biológica, psicológica, social, cultural, entre outras, e componentes, exigindo uma abordagem multicausal e multidimensional, assente num sistema prestador organizado em torno de um elemento central, a pessoa idosa, entendida como um todo no contexto de uma visão holística e de abordagem mais ampla e dirigida para a recuperação ou manutenção dos melhores níveis de saúde possíveis face aos processos de envelhecimento. Para se alcançar uma melhoria do impacto dos cuidados, é imprescindível o investimento na articulação entre a saúde e segurança social, de forma coordenada e centrada na pessoa e nas suas necessidades de cuidado, para que seja encontrada a melhor resposta à satisfação das suas necessidades. A intervenção integrada a nível local permite, ainda, um melhor conhecimento da realidade, respostas mais rápidas e eficientes e o desenvolvimento de soluções mais adequadas e de proximidade, que são essenciais para os processos de tomada de decisão relativos ao bem_estar das pessoas idosas. Os vários níveis de cuidados não devem ser encarados como substitutos uns dos outros, mas como complementares, interligados, cobrindo eficazmente as necessidades individualizadas de cada utente, o qual deve ter a possibilidade de transitar numa dinâmica interdisciplinar, em função das necessidades de saúde. Dependendo das necessidades específicas de cada pessoa e das condições subjacentes que conduzem à perda de funcionalidade, será necessária uma combinação de todos ou de alguns destes serviços, que vão desde o apoio ambulatório, domiciliário, passando pelas instituições residenciais para pessoas idosas, serviços de saúde e as unidades de cuidados continuados integrados. As boas práticas neste domínio reconhecem que as pessoas idosas não são um grupo homogéneo, têm diferentes necessidades, preferências e oportunidades ao longo da sua vida, que a violência contra estas pessoas pode se manifestar de diversas formas, incluindo o abandono e a negligência, ocorrendo frequentemente no ambiente doméstico ou em contexto institucional, e que as expectativas e necessidades das pessoas idosas devem ser contempladas nos diferentes níveis de planeamento e decisão, assim como deve estar assegurado o acesso a cuidados de proximidade, individualizados, centrados na pessoa e integrados nos diferentes níveis de cuidados, com a sua participação e envolvimento. A desejada abordagem integrada do envelhecimento implica não apenas combater o idadismo nas suas diversas formas, mas também promover o acesso a cuidados de qualidade e de longa duração, centrados na integralidade das pessoas e não na doença, garantindo uma intervenção integrada das áreas da saúde e da segurança social que contemple, também, as medidas previstas na estratégia para os direitos das pessoas com deficiência 2021_2030, dando assim particular atenção às pessoas idosas com deficiência. Para garantir o acesso a cuidados centrados nas pessoas idosas, os sistemas devem conhecer e se organizar em torno das suas necessidades e preferências, garantir o envolvimento da família e consequente assunção da sua responsabilidade, de outros cuidadores e da comunidade, integrar diferentes serviços e assegurar cuidados de longa duração. As principais ações que podem ajudar a alcançar esse objetivo são: a avaliação abrangente da saúde das pessoas idosas, a elaboração de plano_individual_de_cuidados para otimizar sua capacidade, o desenvolvimento de serviços de proximidade do local da sua residência pessoal ou institucional, o fornecimento de serviços comunitários, a criação de estruturas de serviços que promovam cuidados por equipas multidisciplinares, o apoio no autocuidado e o aumento da literacia em saúde. Para prestarem cuidados de saúde de qualidade e integrados, os profissional_de_saúde devem desenvolver competências gerontogeriátricas, bem como as competências necessárias para trabalhar com sistema_de_saúde, incluindo trabalho em equipa, tecnologias de informação e comunicação. As erpi devem, na sua atuação, se articular com diferentes serviços ou equipas comunitárias de saúde mental contribuindo, deste modo, para que a prestação de cuidados de saúde mental seja centrada na pessoa idosa, reconhecendo a sua individualidade, necessidades específicas e nível de autonomia, assim como evitando a sua estigmatização, discriminação negativa ou desrespeito em contexto de saúde, tal como se enuncia no decreto_lei número 113_2021, de 14 de dezembro. De forma a melhorar a qualidade dos cuidados de saúde prestados nas erpi, tem sido evidenciada a necessidade de implementar uma alteração efetiva do modelo de prestação de cuidados, abandonando uma visão reativa e implementando uma abordagem pró_ativa, centrada nas necessidades da pessoa idosa, suas famílias e cuidador e profissional_de_saúde envolvidos, definida pela equipa de saúde da erpi, atualizada de forma permanente e espelhada nos registos existentes no plano_individual_de_cuidados de cada pessoa. Esta visão pressupõe o reconhecimento da importância da articulação com os cuidados de saúde primários em cuja área geográfica as erpi se encontram inseridas, em particular no que se refere à identificação e compreensão das necessidades de saúde da população residente nas diferentes erpi, permitindo, deste modo, potenciar a capacidade de resposta às necessidades de saúde identificadas. Os sistemas de informação constituem um instrumento central na prestação de cuidados de saúde, contribuindo para uma melhoria contínua da sua qualidade, através de registos mais claros e completos. A integração da informação de saúde e de informação clínica em sistemas de informação pelos profissional_de_saúde devidamente habilitados, contribui, igualmente, para a continuidade de cuidados centrados nas necessidades da população idosa e de cada residente individualmente considerado. As erpi são, face ao envelhecimento populacional e à crescente necessidade de cuidados continuados em contexto domiciliário, uma componente crescente no sistema de proteção social e de saúde. Neste âmbito, será essencial o desenvolvimento e implementação de uma plataforma digital que permita, através de uma política de acessos desenhada de acordo com a legislação de proteção e acesso a dados pessoais e de saúde, que contribuía para a centralização de informação de saúde, clínica e social, necessária à sustentação dos processos de cuidar e de tomada de decisão. Num modelo que se propõe centrado nas necessidades da população residente em erpi os sistemas de informação devem integrar 3 áreas essenciais. Os registos clínicos são um elemento central essencial na prestação de cuidados de saúde, constituindo um dever deontológico das diferentes profissões de saúde, que se encontram obrigadas a registar de forma clara, completa e precisa as observações, diagnóstico, intervenções e informação considerada adequada, relativamente a cada pessoa. No que se refere às erpi, e dada a centralidade dos cuidados de saúde, os sistemas de informação deverão comportar dados que resultam de avaliação a partir da pessoa idosa, a formulação de diagnósticos, objetivos e intervenções. O registo completo das observações e intervenções realizadas, constitui um dever essencial à qualidade, segurança e continuidade dos cuidados.

**** *id_49 *doc_2 *ano_2014 *autor_3 *área_1

No presente relatório se propõe uma nova abordagem à promoção da saúde, centrada decisivamente na iniciativa dos cidadãos e da sociedade em geral. Visa reduzir a incidência e a duração de doenças crónicas como a diabetes, ambos os parâmetros são mais elevados em Portugal do que na maioria dos países da Europa Ocidental, e mostrar como as metodologias para o incremento da qualidade e um acrescido acesso às evidências científicas melhoram os serviços de saúde e reduzem a despesa. O relatório propõe uma transição do sistema atual, centrado no hospital e na doença, em que todas as ações têm como objeto e alvo o doente, para um sistema centrado nas pessoas e baseado na saúde, em que os cidadãos são parceiros na promoção da saúde e nos cuidados de saúde. O sistema utilizará os conhecimentos e as tecnologias mais atualizados e proporcionará aconselhamento e serviços de elevada qualidade, no domicílio e na comunidade, tal como em hospitais e em centros especializados. Esta visão integra os valores fundadores do sns e se desenvolve com base nos pontos fortes do sistema atual, na competência dos profissional_de_saúde e nas realizações do passado, mas exige novas abordagens, uma infraestrutura diferente e uma base de custos mais baixa e mais sustentável. Uma mudança a esta escala exige uma liderança visionária e corajosa, capaz de unir as pessoas em torno de um novo pacto para a saúde, fixar a rota a seguir e concitar apoio político e da opinião pública. Exige ainda um vasto programa de mudança, liderado por uma aliança alicerçada nos diversos setores da sociedade, com novos sistemas de aprendizagem contínua, avaliação das evidências científicas e implementação das melhorias. Esta mudança tem de ser apoiada por um fundo de transição temporário para financiar o custo da redundância de instalações durante o desenvolvimento do novo sistema. Participação dos cidadãos. A saúde começa em casa: as pessoas terão de intervir muito mais ativamente na gestão da sua própria saúde e contribuir para moldar todo o sistema. Os cidadãos terão de estar na posse dos seus registos de saúde, de dispor de informações sobre a qualidade e os custos dos serviços e de participar nos processos de decisão. Um sistema_de_saúde centrado nas pessoas e baseado em equipas. São necessários novos modelos de serviços que prestem cuidados de saúde integrados a todos os indivíduos, com especial ênfase em gestão de doenças crónicas; desenvolvimento de mais serviços de apoio domiciliário e de proximidade; e criação de redes de especialidades, associadas a centros de referência mas cobrindo todas as regiões do país através da tecnologia e de protocolos partilhados. Novas funções e uma liderança reforçada a todos os níveis. Os profissional_de_saúde estão vocacionados para se tornarem agentes da mudança e da melhoria, mas é necessária liderança nas comunidades e nos serviços de saúde, e os dirigentes deverão cooperar entre si. Todos os profissional_de_saúde deverão passar a desempenhar novos papéis, devendo a sua formação ser adaptada nessa conformidade, enquanto os doentes e as organizações da comunidade carecem de apoio para assumir funções acrescidas de liderança. O presente relatório propõe a transição do sistema atual, centrado no hospital e na doença, em que todas as ações têm como objeto e alvo o doente, para um sistema centrado nas pessoas e na saúde, em que os cidadãos são parceiros na promoção da saúde e nos cuidados de saúde. O sistema utilizará os conhecimentos e as tecnologias mais atualizados e proporcionará aconselhamento e serviços de elevada qualidade, no domicílio e na comunidade, tal como em hospitais e em centros especializados. Uma mudança a esta escala exige uma liderança visionária e corajosa, capaz de unir as pessoas em torno de um novo pacto para a saúde, estabelecer a rota a seguir e concitar apoio político e da opinião pública. Visão: A Comissão encontrou um grande consenso tanto quanto aos traços gerais da visão para o futuro como quanto à necessidade de reformas radicais. As pessoas ouvidas referiram designadamente: a necessidade de dar maior ênfase à prevenção das doenças; a mudança de um sistema baseado nos hospitais e nos profissional_de_saúde para um sistema baseado na comunidade e nas pessoas; o empowerment dos cidadãos e a literacia da saúde; a telemedicina e a medicina personalizada; as sociedades e os ambientes saudáveis; a transição cultural de um puro paternalismo médico para uma parceria responsável. Muitas pessoas advogaram também a necessidade de melhor informação e maior transparência. Os cidadãos estão em condições de desempenhar um papel muito maior em relação à sua saúde e à da sua família, assim como na melhoria da saúde da sociedade. Todas as estruturas e setores da sociedade, educação, ambiente, segurança social, comércio e emprego, assim como a saúde, trabalham em conjunto para promover a saúde e o bem_estar. Há um empenhamento para a melhoria contínua da qualidade e para a aplicação sistemática do conhecimento científico e das tecnologias no apoio à saúde e ao bem_estar. Esta visão representa uma transformação do sistema atual, centrado nos hospitais e na doença, em que todas as ações têm por objeto e alvo o doente, para um sistema centrado nas pessoas e baseado na saúde, em que os cidadãos são parceiros na promoção da saúde e nos cuidados de saúde. Este sistema utilizará os conhecimentos e tecnologias mais atualizados para apoiar a promoção e a melhoria da saúde e prestar serviços de elevada qualidade, com fiabilidade, em todo o país. Estas são as estratégias_chave para a mudança, estando as primeiras 3 explicitamente incorporadas nesta visão: Um novo pacto para a saúde. Uma mudança a esta escala exige uma liderança visionária e corajosa, capaz de unir as pessoas em torno de um novo pacto para a saúde, fixar a rota a seguir e concitar apoio político e da opinião pública. Exige ainda um vasto programa de mudança de gestão, apoiado por novos sistemas de aprendizagem contínua, avaliação das evidências científicas e implementação das melhorias. Esta mudança tem ainda de ser apoiada por um fundo de transição temporário destinado a financiar o custo das redundâncias durante o desenvolvimento do novo sistema. Participação dos cidadãos. A saúde começa em casa, as pessoas terão de intervir muito mais ativamente na gestão da sua própria saúde e contribuir para modelar todo o sistema. Os cidadãos terão de estar na posse dos seus registos de saúde, de dispor de informações sobre a qualidade e os custos dos serviços e de participar nos processos de decisão. Contribuição dos diversos setores da sociedade. As sociedades modernas promovem ativamente o consumo de estilos de vida pouco saudáveis, e os ambientes modernos dificultam as escolhas saudáveis. Para melhorar a saúde são necessárias iniciativas por parte de todos os setores da sociedade e dos cidadãos, bem como do Governo e das autarquias. Os serviços de tratamento atempado, de qualidade elevada e baseados nas evidências científicas beneficiam os doentes e, além disso, reduzem o desperdício e a despesa. A tónica deve estar na melhoria contínua da qualidade, se aplicando sistematicamente a evidência de práticas comprovadas, e em toda a parte. Portugal deve se posicionar para tirar o máximo proveito dos progressos futuros no conhecimento e na tecnologia e desenvolver ainda mais a sua própria investigação biomédica. Um sistema_de_saúde centrado nas pessoas e baseado em equipas. São necessários novos modelos de serviços que prestem cuidados de saúde integrados a todos os indivíduos, com especial ênfase em: gestão de doenças crónicas; desenvolvimento de mais serviços de apoio domiciliário e de proximidade; e criação de redes de especialidades, associadas a centros de referência, mas cobrindo todas as regiões do país através da tecnologia e de protocolos partilhados. Novas funções e uma liderança reforçada a todos os níveis. Os profissional_de_saúde estão vocacionados para se tornarem agentes da mudança e da melhoria, mas é necessária liderança nas comunidades e nos serviços de saúde, e os dirigentes deverão cooperar entre si. Todos os profissional_de_saúde deverão passar a desempenhar novos papéis, devendo a sua formação ser adaptada nessa conformidade, enquanto os doentes e as organizações da comunidade carecem de apoio para assumir funções acrescidas de liderança. A principal fração dos custos do sistema_de_saúde decorre, sobretudo, dos cuidados a pessoas com doenças crónicas de longa duração. Só se alcançará sustentabilidade financeira pela redução da incidência destas doenças e da morbilidade que lhes está associada, desenvolvendo para elas novos modelos de cuidados de saúde e assegurando que as evidências científicas são aplicadas sistematicamente em toda a parte e que o desperdício é reduzido ao mínimo. A introdução de novos mecanismos financeiros e de incentivos baseados nos resultados, se bem geridos, poderá ser útil; mas a viabilidade financeira do sistema irá depender da vontade para incluir a saúde em todas as políticas, da promoção eficaz da saúde e das iniciativas concertadas dos cidadãos, da sociedade em geral e dos profissional_de_saúde. No futuro, tal como defende este relatório, os cidadãos deverão ser parceiros ativos na promoção e na proteção da saúde e na prestação e conceção dos cuidados de saúde. O antigo contrato social implícito, segundo o qual a população era o objeto e o alvo de todas as ações, deve ser substituído por um novo pacto para a saúde no qual todos são parte ativa. A comissão sugere que o Governo dê início à criação de um novo pacto, mas que o conselho_nacional_de_saúde aqui proposto seja incumbido do seu desenvolvimento e funcione como consultor quanto à sua forma final. Os cidadãos como parceiros e parte ativa; profissional_de_saúde que disponibilizam os seus conhecimentos e competências: assegurando a aplicação da evidência e do rigor científicos, bem como funcionando com padrões elevados de qualidade; Organizações em toda a sociedade que alinham as suas políticas e práticas para promover a saúde e prevenir as doenças; Criação pelo Governo da estrutura e do enquadramento para um sistema_de_saúde sustentável e de elevada qualidade; conselho_nacional_de_saúde: uma aliança de toda a sociedade, que terá a tutela do pacto e da visão para o futuro e providenciará aconselhamento sobre as políticas de saúde. Esta proposta para criar o conselho_nacional_de_saúde complementa os papéis desempenhados pelo ministério_da_saúde e outras entidades na liderança e gestão do sns. Uma última recomendação deste relatório apela à clarificação da responsabilização das estruturas de supervisão e gestão do sistema_de_saúde. É necessário que estes 2 elementos, um organismo consultivo que reúna todos estes setores e uma estrutura executiva que supervisione e realize a gestão, funcionem bem, por forma a proporcionarem a liderança e a orientação necessárias para todo o sistema_de_saúde, concretizando um vasto programa de mudança. Deverá ser estabelecido um novo pacto na saúde, que configure as novas interações e os desempenhos necessários para a transformação do sistema atual, centrado no hospital e na doença, em que o doente é objeto e alvo de todas as ações, num sistema centrado nas pessoas e baseado na saúde, em que os cidadãos são parceiros na promoção da saúde e nos respetivos cuidados. O conselho_nacional_de_saúde deverá ser estabelecido como uma aliança de toda a sociedade com a incumbência de tutelar o pacto para a saúde e definir a visão para o futuro, ter uma perspetiva de conjunto do sistema e funcionar como consultor para as políticas que se integrem nessa visão. Esse organismo deverá ser representativo dos cidadãos e de todos os setores da sociedade, ser politicamente independente e responsável perante a Assembleia da República, o ministro_da_saúde e a população em geral. O sns_evidência será um novo organismo que conjugará o programa existente de normas de orientação clínica com novos processos de avaliação das novas tecnologias e terapias, assegurando que o sistema faculta, de forma sempre atualizada e crítica, o melhor conhecimento científico disponível. Este organismo deverá divulgar as suas deliberações e os resultados de que disponha junto dos cidadãos para que estes, assim como os médicos e outros profissional_de_saúde, possam conhecer essas evidências. Criar um fundo de transição que possa ajudar a suportar os custos de redundância e outros custos provisórios do desenvolvimento de um sistema_de_saúde, em que haja melhor gestão das doenças crónicas, mais serviços domiciliários e de proximidade e redes de especialidades cobrindo todas as regiões do país. Este capítulo se centra na criação de saúde e naquilo que os indivíduos e as suas famílias, os cuidadores e os amigos podem fazer. Estes 2 objetivos, que os cidadãos se tornem mais ativos relativamente à saúde e que as estruturas e setores da sociedade colaborem para promover a saúde e o bem_estar, estão no centro da visão que a Comissão tem do futuro. Em conjunto podem coproduzir melhor saúde em Portugal. Este relatório sublinha a importância de os cidadãos assumirem maior controlo e responsabilidade. Reconhece, no entanto, que há questões culturais, de mercado e ambientais que podem levantar dificuldades e constituir barreiras que devem ser ultrapassadas. Quase todos os contactos prévios da Comissão destacaram estes e outros aspetos com ele relacionados, mas também refletiram sobre a dificuldade de efetuar esta mudança na prática. A Comissão foi informada de que em Portugal a população é geralmente muito passiva nas suas relações com o sistema_de_saúde; não se sente capaz de questionar os médicos e utiliza de forma muito limitada as fontes de informação sobre saúde. Houve muitos apelos para promover uma modificação desta cultura, quer por parte da população, quer por parte dos profissional_de_saúde. O grupo 2 da comissão defendeu uma mudança de paradigma, de um sistema paternalista de saúde para um sistema que coloque os cidadãos no seu centro. A comissão também foi informada de que há, na prática, muito pouca informação disponível para os cidadãos, por exemplo no que respeita à qualidade dos serviços, e à transparência em matéria de dados estatísticos e práticas clínicas. Esta falta de dados estatísticos e de transparência é um problema relevante em Portugal e será referido frequentemente neste relatório. O modo como abordamos a medicina nestes últimos 100 ou mais anos, à medida que fomos entregando cada vez mais o controlo aos profissional_de_saúde, consistiu em procurar obter salvaguardas do Estado através da regulamentação e da fiscalização, confiando nos profissional_de_saúde, no seu Juramento de Hipócrates e na sua missão social. Esta abordagem já não é por si própria suficiente, e exige maior transparência e uma participação mais ativa dos cidadãos e dos doentes na governação dos sistema_de_saúde e no apoio e na definição das suas prioridades. Muitos profissional_de_saúde e representantes dos doentes afirmaram à Comissão que é necessário abandonar o paternalismo tradicional dos profissional_de_saúde e do sistema_de_saúde, evoluindo para um relacionamento mais equitativo. Isto terá de ser apoiado por alterações na formação profissional e no curriculum académico, e exigirá também maiores níveis de literacia da saúde por parte de todos os cidadãos, se iniciando a educação para a saúde numa fase precoce da vida das crianças. Esta nova abordagem vai, sem dúvida, suscitar resistências por parte de alguns profissional_de_saúde, que foram formados num ambiente diferente e que poderão a encarar como uma ameaça à sua posição e autoridade. Outros, no entanto, verão como uma evolução positiva e reconhecerão o potencial de cidadãos melhor informados e dispostos a desempenhar um papel mais abrangente nos seus próprios cuidados de saúde. Estas 4 recomendações ajudarão a criar um novo ambiente e uma nova cultura nos quais os cidadãos sejam o centro do sistema_de_saúde, conforme refere o plano_nacional_de_saúde, e onde possam ser o centro de gestão das suas próprias vidas, como sugere o Grupo de Trabalho 2. Deverá haver um novo programa intersetorial do Governo para a educação e literacia da saúde que prepare, em termos práticos, os cidadãos para se manterem saudáveis e para, quando doentes, participarem na tomada de decisões. Este programa deverá ter grande visibilidade e ser liderado por um grupo de Embaixadores, incluindo personalidades dos meios de comunicação, em coordenação com os ministério_da_saúde e da educação. Deverá ser dada elevada prioridade à plena implementação de um registo_de_saúde_eletrónico, como base essencial para um serviço integrado de alta qualidade que faculte aos cidadãos, em formato eletrónico ou outro, toda a informação sobre a sua saúde. Deverá haver uma fonte única de informação acreditada acessível a todos os cidadãos, eventualmente integrada no Portal da Saúde. Conterá informações sobre saúde, prevenção de doenças, serviços prestados e sua qualidade. As organizações de saúde deverão estar obrigadas a fornecer determinadas informações para esse fim e as entidades reguladoras deverão assegurar­ que os cidadãos têm acesso a elas. O ministério_da_saúde deverá nomear não_profissionais para representar a perspetiva dos cidadãos e dos doentes perante os órgãos de gestão das instituições de saúde. A integração dos serviços e a personalização dos cuidados de saúde são temas recorrentes neste relatório. Estes temas se tornam ainda mais importantes à medida que a população envelhece e que mais pessoas sofrem de doenças crónicas e de comorbilidades. É fundamental que os cuidados de saúde para todos os indivíduos sejam bem planeados e coordenados, e não fragmentados e incongruentes. Neste capítulo se aborda a forma como se podem desenvolver cuidados de saúde verdadeiramente integrados e centrados nas pessoas, por meio de 3 grupos principais de ações estratégicas: Criação de novos modelos de prestação de serviços; Reestruturação do sistema_de_saúde; Parcerias no planeamento e na prestação de serviços. Um sistema_de_saúde para o futuro tem de estar centrado nas pessoas e ter capacidade para prestar cuidados de saúde integrados a todos os indivíduos, o que implica grandes mudanças. Estas mudanças proporcionam a oportunidade para uma grande criatividade no desenvolvimento de novos modelos de serviços e para uma nova conceção do sistema_de_saúde, por forma a ir ao encontro das necessidades do século 21. Tal como este capítulo mostrou, Portugal tem já muitas das características_chave que lhe irão permitir a melhoria dos serviços e dos sistemas. São precisas, no entanto, algumas medidas imediatas para acelerar e consolidar as mudanças necessárias. Este capítulo se baseou, em grande parte, nas deliberações do Grupo de Trabalho 1 e nas contribuições de diversas partes interessadas. No seguimento das discussões com este Grupo e das deliberações que se seguiram, a Comissão decidiu efetuar 3 recomendações nesta área. Têm de ser desenvolvidos mais programas de liderança no interior do sistema_de_saúde e estes deverão ocorrer a par do desenvolvimento da liderança junto da população em geral. Os profissionais de educação, os cidadãos e os dirigentes autárquicos, por exemplo, poderão contribuir para melhorias na saúde e no bem_estar. Todos, incluindo os profissional_de_saúde, poderão também contribuir para melhorias mais vastas na sociedade. É também necessário que haja melhores capacidades de gestão em todo o sistema. Apesar de os principais gestores poderem ter as competências e a experiência necessárias para o desempenho das suas funções, são geralmente os gestores de nível intermédio e elementar, envolvidos nas decisões do quotidiano, que afetam o serviço que é efetivamente prestado aos doentes. A abordagem da melhoria de qualidade descrita no Capítulo 4 necessitará da participação desses gestores, a quem sejam dados poderes de decisão, bem como de profissional_de_saúde qualificados e abertos à mudança. Além disso, é também preciso que mais médicos tenham capacidades de gestão e assumam um papel reforçado na organização dos seus serviços. Características dos líderes transformativos ou agentes de mudança: Centrados nas pessoas; Paixão pelas melhorias; Disposição para assumir responsabilidade e liderar; Utilização de evidência científica; Pensamento criativo capaz de ultrapassar obstáculos; Gestão de impactos noutros indivíduos e práticas; Utilizar o sistema para alcançar o sucesso. A Comissão preparou sete grupos de recomendações destinadas a colocar Portugal no caminho da criação do futuro de um sistema_de_saúde sustentável e de elevada qualidade. Um novo pacto para a Saúde: Deverá ser estabelecido um novo pacto na Saúde, que configure as novas interações e os desempenhos necessários para a transformação do sistema atual, centrado no hospital e na doença, em que o doente é objeto e alvo de todas as ações, num sistema centrado nas pessoas e baseado na saúde, em que os cidadãos são parceiros na promoção da saúde e nos respetivos cuidados. conselho_nacional_de_saúde: O conselho_nacional_de_saúde deverá ser estabelecido como uma aliança de toda a sociedade com a incumbência de tutelar o pacto para a saúde e definir a visão para o futuro, ter uma perspetiva de conjunto do sistema e funcionar como consultor para as políticas que se integrem nessa visão. Esse organismo deverá ser representativo dos cidadãos e de todos os setores da sociedade, ser politicamente independente e responsável perante a Assembleia da República, o Ministro da Saúde e a população em geral. sns_evidência: o sns_evidência será um novo organismo que conjugará o programa existente de normas de orientação clínica com novos processos de avaliação das novas tecnologias e terapias, assegurando que o sistema faculta, de forma sempre atualizada e crítica, o melhor conhecimento científico disponível, as evidências científicas. Este organismo deverá divulgar as suas deliberações e os resultados de que disponha junto dos cidadãos para que estes, assim como os médicos e outros profissional_de_saúde, possam conhecer essas evidências. Fundo de Transição: Criar um fundo de transição que possa ajudar a suportar os custos de redundância e outros custos provisórios do desenvolvimento de um sistema_de_saúde, em que haja melhor gestão das doenças crónicas, mais serviços domiciliários e de proximidade e redes de especialidades cobrindo todas as regiões do país. Literacia da saúde: Deverá haver um novo programa intersetorial do Governo para a educação e literacia da saúde que prepare, em termos práticos, os cidadãos para se manterem saudáveis e para, quando doentes, participarem na tomada de decisões. Este programa deverá ter grande visibilidade e ser liderado por um grupo de Embaixadores, incluindo personalidades dos meios de comunicação, em coordenação com os ministério_da_saúde e ministério da educação. Propriedade da informação pessoal sobre saúde. Deverá ser dada elevada prioridade à plena implementação de um registo_de_saúde_eletrónico, como base essencial para um serviço integrado de alta qualidade que faculte aos cidadãos, em formato eletrónico ou outro, toda a informação sobre a sua saúde. Acesso à informação. Deverá haver uma fonte única de informação acreditada acessível a todos os cidadãos, eventualmente integrada no Portal da Saúde. Conterá informações sobre saúde, prevenção de doenças, serviços prestados e sua qualidade. As organizações de saúde deverão estar obrigadas a fornecer determinadas informações para esse fim e as entidades reguladoras deverão assegurar que os cidadãos têm acesso a elas. Representação: O ministério_da_saúde deverá nomear não profissionais para representar a perspetiva dos cidadãos e dos doentes perante os órgãos de gestão das instituições de saúde. Autarquias. As autarquias, algumas das quais desempenham papéis de liderança no bem_estar das populações que representam, devem incluir a saúde e as organizações de saúde nas suas parcerias locais de assistência social e apoiar o trabalho e o planeamento intersetoriais. Sociedade civil. As autarquias, as organizações de saúde, as organizações do mercado e os ministérios adequados devem trabalhar em conjunto para encontrar melhores formas de promover, apoiar e envolver no sistema_de_saúde e de cuidadores. saúde_pública. O Governo, os ministérios em causa, o sns e as instituições e associações de saúde_pública devem trabalhar em conjunto para reforçar a intervenção da saúde_pública a todos os níveis e em todo o sistema_de_saúde. Informação e competências em saúde_pública devem ser fortalecidos na formação e qualificação de todos os profissional_de_saúde. Poderão ser necessários novos modelos e enquadramentos legislativos em saúde_pública para alcançar esse propósito. Melhoria contínua da qualidade. Todos os parceiros no sistema_de_saúde, desde o ministério_da_saúde e os cidadãos até aos vários organismos do sns, municípios, universidades e indústria têm de assegurar que a abordagem à melhoria contínua da qualidade é adotada em todo o sistema_de_saúde como meio de promover a sua qualidade e garantir que os progressos científicos e tecnológicos são integrados e implementados de um modo sistemático e generalizado. As competências para a melhoria da qualidade devem fazer parte dos programas de formação e treino de todos os profissional_de_saúde. Para este fim, deverá ser criado um organismo de peritagem responsável por promover a melhoria da qualidade, identificar e disseminar as boas práticas e apoiar a sua implementação. Deve se articular com outros organismos, na saúde e em outras áreas da governação para criar modelos para a implementação da melhoria da qualidade, promover o treino, conselho e apoio, o que deve ser acompanhado pela normalização dos processos de acreditação, registo e reporte. Colaboração do sns com a investigação e a indústria. Os dirigentes do sns, da comunidade científica e da indústria devem colaborar para a criação de centros nacionais que, trabalhando em conjunto com os cidadãos, promovam o desenvolvimento de novas práticas, tecnologias e serviços. Novos modelos para os cuidados de saúde. Os decisores políticos, os responsáveis pelo planeamento e os prestadores de serviços devem trabalhar em conjunto para criar serviços de cuidados integrados com especial ênfase nos que se destinam à gestão de doenças crónicas, à disponibilização de mais cuidados domiciliários e de proximidade e à criação de redes de especialidades, associadas a centros de referência e cobrindo todas as zonas do país através da tecnologia e de protocolos partilhados. Responsabilização e custos administrativos. As responsabilidades precisam de ser bem estabelecidas em todo o sistema e é necessária uma análise sobre o número e as funções de muitos organismos públicos associados à saúde e respetivos cuidados, reduzindo o seu número e respetivos custos em pelo menos 25 porcento, com isso libertando financiamento para investimentos noutras áreas. Acordo público_privado. Deve ser estabelecido um acordo público_privado como enquadramento para a contratação de serviços privados pelo sns, salvaguardando o interesse público e trazendo, em simultâneo, novos recursos e potenciais inovações ao sistema_de_saúde. Formação profissional. Os Ministérios da Educação e ministério_da_saúde deverão estabelecer um projeto global para revisão da formação profissional à luz dos desenvolvimentos atuais e futuros da saúde e assegurar que essa reformulação inclui as novas necessidades de parceria com os doentes, de melhoria da qualidade e de reforço da saúde_pública. Enfermagem. Em Portugal, o estatuto dos enfermeiros deverá ser promovido através da nomeação de um diretor_geral de Enfermagem, reforçando o papel destes profissionais nas organizações e, tanto quanto o orçamento permita, aumentando o seu número. No seguimento da introdução da nova figura proposta de enfermeiro de família, deverá também haver uma reflexão sobre o alargamento do papel dos enfermeiros e de outros profissionais em outras áreas. Estratégia de sustentabilidade. Adotar uma estratégia de sustentabilidade para melhorar a qualidade e reduzir os custos, baseada em: Definição de três áreas principais de mudança: Redução da morbilidade, prevenção das doenças e diminuição do tempo de enfermidade; Aplicação eficaz das evidências científicas e adoção da melhoria contínua da qualidade, com o objetivo de diminuir o desperdício em saúde; Mudança da infraestrutura do sistema_de_saúde Desenvolver o processo de contratualização e contratação: centrar as medidas nos resultados; analisar continuamente a experiência internacional associada à introdução de concorrência; e assegurar flexibilidade suficiente desses mecanismos para fazer face às exigências da evolução dos cuidados de saúde, através de novos métodos e formas de prestação. Gestão financeira. Melhorar a gestão financeira, adotar uma melhor governação, disponibilizar amplamente a informação de dados estatísticos e reforçar a responsabilização. Se propõe ainda criar um fundo de maneio de 5 anos, o Fundo de Estabilização do sns, que funcionará como apoio ao planeamento, e estabelecer auditorias independentes para avaliar serviços na perspetiva da relação benefício_custo, que responderão perante o Parlamento e o conselho_nacional_de_saúde.

**** *id_51 *doc_2 *ano_2024 *autor_3 *área_1

O sistema de informação de saúde é um instrumento essencial para obter os resultados em saúde desejados, tanto a nível individual como populacional. Deve servir as pessoas que recebem cuidados e os profissional_de_saúde que delas cuidam, correspondendo às necessidades e exigências dos seus utilizadores, assente em unidades de saúde com maior eficácia e maior eficiência. A humanização, a personalização, a segurança, a qualidade e a integração de dados e informação de saúde são princípios essenciais a respeitar. Atualmente, a informação de saúde de cada pessoa está dispersa por silos desligados. O fluxo de informação terá de ser melhorado. Os dados e a informação deverão fluir, com segurança, dentro da mesma organização e entre os diferentes níveis e tipos de cuidados e diferentes organizações. A centralização dos cuidados de saúde no utente só será conseguida se a informação circular com o utente. Se considerarmos o percurso das pessoas no sistema_de_saúde, o sistema de informação tem de ser um instrumento facilitador da integração, da continuidade e da coordenação de cuidados, em especial quando a pessoa viva com doença crónica, independentemente dos serviços e instituições que lhe prestem os cuidados. Deve também ser um instrumento para a melhoria contínua da qualidade desses cuidados e dos seus resultados e promover o autocuidado. O atualmente designado registo_de_saúde_eletrónico, pode vir a constituir um ponto de partida e base para um futuro processo clínico pessoal inteligente, integrador e sumarizado. Porém, ainda está muito longe da visão desejada tanto na ótica do utente como dos profissional_de_saúde, na sua grande diversidade disciplinar e nos requisitos do trabalho multidisciplinar centrado nas necessidades e nos objetivos dos planos de cuidados para cada pessoa. A concretização dessa visão requer um complexo processo de implementação que deve ser acompanhado a par e passo pelo feedback do também complexo e diverso universo dos seus utilizadores. A pessoa é a principal protagonista da sua saúde, tendo de estar necessariamente no centro da informação e dos processos de tomada de decisão. Isto é, informação recolhida sobre a pessoa deve estar sempre disponível, independente do local onde foi produzida. Se considera, assim, essencial que as pessoas tenham acesso à sua informação e façam escolhas conscientes acerca da sua saúde e bem_estar. Com a premissa da centralidade da pessoa e no que se refere à informação para a sua saúde, importa refletir sobre determinados aspetos: os dados em saúde acompanham a vida da pessoa, a sua recolha apresenta oportunidades, mas também riscos, e a qualidade dos dados é crítica para a informação ser fidedigna. Num modelo de saúde colaborativo e participativo, é essencial que as pessoas tenham acesso à sua informação e façam escolhas acerca da sua saúde. É cada vez mais reconhecida a importância de a pessoa ser a principal protagonista e parceira dos profissional_de_saúde e dos serviços nos processos de tomada de decisão. Mas não é suficiente a pessoa estar envolvida ou ser parceira. Deve ser o centro da atenção e a principal protagonista em qualquer modelo de saúde e bem_estar. O conceito de centralidade da pessoa é usado com frequência crescente e apontado como princípio fundamental de todos os processos relacionados com cuidados de saúde. Importa ainda fazer referência aos objetivos de desenvolvimento sustentável, nomeadamente ao objetivo de desenvolvimento sustentável 3 que tem como meta assegurar uma vida saudável e promover o bem_estar para todas e todos, em todas as idades. Se considerar essencial para esta meta, o empowerment da pessoa, o acesso à sua informação, fomentando a sua capacidade individual na tomada de decisões e na mudança de comportamentos para uma vida saudável. Tendo como premissa a centralidade da pessoa e no que se refere à informação para a sua saúde, importa salvaguardar e refletir sobre alguns aspetos. Recomendações específicas: Garantir, na prática, que a pessoa está no centro do sistema, participa no processo e ou decisão, e tem acesso à informação e aos vários recursos de saúde ao longo da vida. Assegurar que a pessoa compreende e usa os vários recursos em benefício da sua saúde ou de quem dela depende, com a envolvência dos profissional_de_saúde ou os vários atores, para uma melhor navegabilidade no sistema_de_saúde e na gestão ativa da sua saúde_doença e tomada de decisão acertada, para os melhores resultados em saúde. Garantir que a pessoa tem controlo sobre os seus dados em saúde e que existem mecanismos efetivos que assegurem a confidencialidade e a manutenção da confiança que deposita nos profissional_de_saúde e nos serviços, na área da saúde. A transformação digital dos cuidados de saúde pode ser disruptiva; no entanto, tecnologias como a internet of things, as consultas virtuais, a monitorização remota, a inteligência artificial, a análise de big data, blockchain, smart wearables, plataformas, e outras ferramentas que permitem o intercâmbio e armazenamento de dados, a recolha remota e a partilha de informações relevantes em todo o ecossistema de saúde que criam um contínuo de cuidados, têm um potencial comprovado para melhorar os resultados em saúde. Quer através da melhoria do diagnóstico, das decisões baseadas em dados, dos ensaios clínicos, da autogestão de cuidados e cuidados centrados na pessoa, quer na criação de mais conhecimento baseado em evidência e em dados e no aumento das competências dos profissional_de_saúde. A ligação de dados em saúde, tanto entre diferentes fontes de dados na área da saúde quanto com conjuntos de dados externos, oferece um vasto potencial para avançar a compreensão, a gestão e a promoção da saúde. Essa abordagem integrada permite uma visão abrangente e holística, possibilitando informações relevantes e melhorias nos cuidados de saúde e nas políticas públicas. Informações abrangentes sobre o percurso do utente: ao vincular dados provenientes de diferentes fontes, como registo_de_saúde_eletrónico, históricos clínicos e resultados de exames, é possível obter uma visão mais completa do percurso do utente. Isso facilita uma compreensão mais profunda das doenças, tratamentos anteriores e respostas individuais a intervenções dos profissional_de_saúde. Personalização dos cuidados de saúde: com a ligação de dados, é possível desenvolver abordagens personalizadas para os cuidados de saúde. Ao entender melhor as características individuais dos utentes, como genética, estilos de vida e histórico de saúde, os profissional_de_saúde podem adaptar as intervenções para atender às necessidades específicas de cada pessoa. Um indicador de saúde pode ser concetualizado como uma ponte entre o conhecimento científico e a política de saúde. Devem existir orientações adequadas para interpretar as tendências reveladas por estes indicadores. É necessário um modelo concetual de saúde e de bem_estar para facilitar essa interpretação. Um modelo que se centre nas pessoas e nas comunidades, nos ganhos em saúde, na equidade do acesso ao sistema e no desenvolvimento qualitativo permanente. Recomendações específicas: Garantir uma maior integração dos princípios de interoperabilidade dos indicadores e da informação em saúde, envolvendo todos os atores do sistema_de_saúde, e a aquisição dos instrumentos informáticos mais eficientes. Considerar neste contexto a integração saúde e social, designadamente no que respeita aos cuidados continuados integrados, uma vez que persistem obstáculos no percurso de saúde de doentes com alta hospitalar, mas sem local de acolhimento na reabilitação, cuidados continuados, ou paliativos. Definir novos indicadores de saúde, como base para a avaliação do sistema, que se afastem da exclusiva contabilização de atos realizados e se centrem nos ganhos em saúde observados e que integrem a perspetiva da pessoa e da comunidade; incluir indicadores sociais no sistema de informação de saúde. Envolver, de forma efetiva, a pessoa na construção da sua saúde, integrando uma perspetiva salutogénica e de modificação comportamental, essencial para o envolvimento de pessoas e comunidades na gestão do sistema_de_saúde. Identificar a informação necessária e tecnicamente considerada como essencial para a gestão eficaz das doenças raras, crónicas ou complexas e da sua comorbilidade, para a adequação do sistema às necessidades e expectativas de pessoas e comunidades; dotar o sistema_de_saúde de uma estratégia efetiva de planeamento a médio e longo prazo dos profissional_de_saúde necessários ao sistema, em número e competências. É fundamental um modelo de governação de dados centrado nas pessoas, que possa assegurar a efetiva portabilidade da sua informação e rigorosos dispositivos de segurança e confidencialidade. Este modelo de governação de dados centrado nas pessoas é igualmente o que melhor serve a sua utilização secundária, garantindo a confiança necessária nas estruturas governamentais para assegurar a confidencialidade e a proteção de dados sensíveis, como os dados de saúde. Se Veja, recentemente, o lançamento da infraestrutura HealthData_PT. Recomendações específicas: Garantir que a pessoa pode aceder e deter controlo sobre os seus dados de saúde, permitindo aos profissional_de_saúde o acesso e a interação na sua gestão de saúde_doença. Assegurar que seja implementado um modelo de governação de dados centrado nas pessoas, que possa assegurar a efetiva portabilidade da sua informação e rigorosos dispositivos de segurança e de confidencialidade. Deve haver um compromisso político e social claro para transformar o sns, bem como o sistema_de_saúde como um todo, com base na evidência proveniente da informação e do conhecimento científico disponíveis, estimulando a inovação e a eficiência de um modo descentralizado e com a máxima adaptação às necessidades quer nacionais, quer locais, com envolvimento ativo das pessoas. Uma visão nacional, trazendo um excerto do capítulo do relatório de primavera do observatório português dos sistema_de_saúde, de 2022, sobre saúde digital. Se refere nele que a saúde digital, ao permitir armazenar, analisar e partilhar informação relevante, promove o estabelecimento de uma rede de partilha e colaboração, fundamental na implementação de projetos de integração de cuidados, contribuindo para a prestação de cuidados mais centrados no utente, atendendo às suas necessidades específicas, e para a melhoria da efetividade, eficiência e qualidade dos cuidados. Destaque referido por organizações de pessoas que vivem com doença: que o sistema de informação de saúde esteja centrado e orientado para responder às necessidades concretas das pessoas em cada situação e momento. Necessidade de redesenho, aprimoramento e generalização do uso do plano_individual_de_cuidados: Foi identificada a necessidade urgente de desenvolver e implementar, no dia_a_dia, o plano_individual_de_cuidados. Este é componente indispensável do processo clínico pessoal. Está, naturalmente, alicerçado e baseado no registo_de_saúde_eletrónico. Foi referido ter sido um projeto iniciado há anos, mas interrompido. Se espera que o seu redesenho e implementação seja feita, ainda que progressivamente, de modo sistemático e universal. Cada pessoa deve poder ter, conhecer e participar, com mediação profissional, se necessário, no seu plano_individual_de_cuidados. Este, deve ser o guia base para promover, proteger e manter a sua saúde. Tal inclui seguir corretamente os tratamentos que tenha em curso. O plano_individual_de_cuidados é aplicável a qualquer pessoa, com ou sem doença.

**** *id_52 *doc_2 *ano_2014 *autor_2 *área_1

A este nível, importa destacar, como iniciativas que permitem ao utente exercer o seu direito de liberdade de escolha informada, a parceria estabelecida entre a Administração Central do sistema_de_saúde e a Associação Nacional de Unidades de Saúde Familiar, para efeitos de disponibilização e comparação dos resultados alcançados pelas diferentes unidades prestadoras de cuidados de saúde primários, a criação do microsite de Monitorização do sns, que tem permitido a divulgação de informação sobre as diferentes dimensões de análise do sistema_de_saúde, acesso, eficiência, efetividade, produção e satisfação, e os dashboards da saúde que fornecem informação que permite a monitorização mensal do estado de saúde da população portuguesa. O esforço na produção de informação e conhecimento sobre os prestadores é, pois, essencial para o desenvolvimento futuro de um mercado público de cuidados de saúde em que se pretende que os utentes sejam a peça central do sistema enquanto consumidores informados.

**** *id_54 *doc_2 *ano_2016 *autor_2 *área_3

Por outro lado, se tornou essencial a promoção do recurso a boas práticas de utilização de instrumentos e sistemas de informação com importância no acesso a cuidados de saúde, vertidas na criação do siga sns, o qual permitirá nos próximos anos melhorar a integração e articulação dos sistemas de gestão clínica, de informação ao cidadão e de gestão global das instituições. O esforço na produção de informação e conhecimento sobre os prestadores é pois essencial para que os utentes sejam verdadeiramente agentes centrais do sistema_de_saúde em geral e do sns em particular.

**** *id_55 *doc_2 *ano_2017 *autor_2 *área_3

Os hospitais do sns têm respondido positivamente ao aumento crescente da procura, implementando diversas medidas de reorganização interna que permitam obter melhores resultados em saúde para os utentes e que promovam elevados níveis de eficácia e eficiência na gestão e na governação clínica, com maior transparência, responsabilização, prestação de contas e centralidade no cidadão e na sua família. De facto, foram iniciadas durante o ano de 2016 várias reformas que incidem sobre a organização interna dos hospitais, se destacando a implementação de diversos mecanismos de responsabilização e avaliação, a garantia da melhoria da informação clínica e de gestão, o aprofundamento das relações de parceria e complementaridade entre as várias estruturas do sns, o reforço da coordenação e a articulação com outros níveis de cuidados e outros agentes, da saúde e sociais, entre outras. Os Cuidados Continuados Integrados estão centrados na recuperação global da pessoa, promovendo a sua autonomia e a sua funcionalidade, no âmbito da sua situação de dependência. A Rede Nacional de Cuidados Continuados Integrados, criada através do decreto_lei número 101_2006, de 6 de junho, tem como principais objetivos a prestação de cuidados de saúde e de apoio social de forma continuada e integrada a pessoas que, independentemente da idade, se encontrem em situação de dependência e com perda de autonomia. Este inovador modelo de prestação de cuidados atravessa de forma transversal o sns e as respostas institucionais do setor social, assentando no funcionamento em rede, onde as diferentes tipologias de resposta fazem a articulação entre o nível hospitalar e o nível de cuidados primários, criando assim uma rede que interrelaciona a clássica organização parcelada que até ao aparecimento da Rede Nacional de Cuidados Continuados Integrados caracterizava as respostas do setor social e da saúde. Com a Rede Nacional de Cuidados Continuados é promovida a autonomia e a funcionalidade das pessoas, através da reabilitação, readaptação e reinserção familiar e social pelo que, para cada situação é preconizada uma abordagem integrada de saúde e ação social. O projeto piloto sns_mais_proximidade começou a ser preparado em 2016 e iniciou a sua implementação prática nos primeiros meses de 2017, tendo como principais objetivos: Desenvolver, ensaiar e avaliar um conjunto de procedimentos e instrumentos necessários para iniciar uma transformação qualitativa no sistema_de_saúde português através de uma melhoria significativa: Da integração de cuidados de saúde; Da gestão dos percursos das pessoas nos cuidados de saúde, quer em relação à doença aguda quer naquilo que diz respeito a pessoas com múltiplos problemas de saúde e do investimento na capacitação do cidadão na promoção e proteção da sua saúde e na boa utilização dos serviços de saúde. Ainda no âmbito deste projeto, e de forma a promover a literacia em saúde, a área do cidadão do portal sns já disponibiliza o acesso à biblioteca de literacia em saúde e a livros digitais. A área do cidadão disponibiliza, também, novos plano_individual_de_cuidados que permitem ao cidadão, em conjunto com a sua equipa de saúde, criar um plano com metas bem definidas, tendo como finalidade a promoção da saúde, a prevenção da doença e uma melhoria da qualidade_de_vida. Em setembro de 2016, a área do cidadão do portal sns foi revista, melhorada e aumentada, permitindo uma navegação mais intuitiva, uma mais clara alocação dos serviços eletrónicos por categorias e foi iniciado o processo de revisão dos serviços à luz conceito da centralização no cidadão e no seu percurso de vida, criando a componente de Planos de Cuidados e Percurso de Vida, que será ampliada e corporizada durante 2017. A área do profissional é uma plataforma centrada no utente, que permite o acesso, pelos profissional_de_saúde, médicos e enfermeiros, à sua informação clínica. A informação que o utente disponibiliza na plataforma de dados de saúde na área do cidadão e cuja consulta é por ele autorizada, permite ao profissional_de_saúde obter alguns indicadores que o podem auxiliar a um melhor conhecimento, diagnóstico e tratamento do utente. O acesso a esta área do profissional é efetuado através do sistema informático utilizado pelo prestador de serviços de saúde e está disponível em instituições públicas e privadas, disponibilizando acesso a informação constante das bases de dados locais. Este portal permite a intercomunicação entre os sistemas de informação de cada uma das instituições de saúde do sns, viabilizando, assim, a agregação e visualização da informação de saúde dos utentes registados, quando e onde for necessário. O Plano Estratégico para o Desenvolvimento dos Cuidados Paliativos para o biénio 2017_2018 preconiza o desenvolvimento de uma rede nacional de cuidados paliativos funcional, plenamente integrada no sns e implementada em todos os níveis de cuidados de saúde, que permita a equidade no acesso a cuidados paliativos de qualidade, adequados às necessidades holísticas, físicas, psicológicas, sociais e espirituais, e preferências dos doentes e suas famílias.

**** *id_56 *doc_2 *ano_2018 *autor_2 *área_3

Os hospitais do sns têm respondido positivamente ao aumento crescente da procura, implementando diversas medidas de reorganização interna que permitam obter melhores resultados em saúde para os utentes e que promovam elevados níveis de eficácia e eficiência na gestão e na governação clínica, com maior transparência, responsabilização, prestação de contas e centralidade no cidadão e na sua família. As melhorias da resposta tiveram por base várias reformas com incidência na organização interna dos hospitais, se destacando a implementação de diversos mecanismos de responsabilização e avaliação, a garantia da melhoria da informação clínica e de gestão, o aprofundamento das relações de parceria e complementaridade entre as várias estruturas do sns, o reforço da coordenação e a articulação com outros níveis de cuidados e outros agentes, da saúde e sociais, entre outras. O processo de contratualização hospitalar de 2017 procurou contribuir para a promoção do acesso ao sns, introduzindo um conjunto de orientações e de medidas inovadoras para a melhoria do desempenho assistencial das instituições do sns, com destaque para as seguintes: alargar o livre acesso e circulação do utente no sns, diversificando as alternativas e aumentando a sua capacidade de intervir de forma pró_ativa e responsável na gestão do seu estado de saúde e bem_estar; incentivar a transparência e a cultura da prestação de cuidados de saúde em equipa multidisciplinar e multiprofissional, promovendo a efetiva articulação e coordenação e uma resposta centrada no utente; consolidar os processos de afiliação e de trabalho em rede colaborativa no sns, centrando a organização dos cuidados nas necessidades e percursos do utente e incentivando a cooperação entre instituições; No decurso do ano de 2016, se concetualizou uma nova abordagem na literacia em saúde e integração de cuidados, se iniciando em 2017 o desenho dos instrumentos organizacionais, informativos e comunicacionais necessários para este fim e criando as condições para que seja possível ensaiar um salto qualitativo significativo no sns: um sns mais próximo, melhor centrado nas pessoas, mais qualificado, com maior capacidade de resolução. O projeto piloto sns_mais_proximidade iniciou a sua implementação prática nos primeiros meses de 2017, tendo como principais objetivos: Desenvolver, ensaiar e avaliar um conjunto de procedimentos e instrumentos necessários para iniciar uma transformação qualitativa no sistema_de_saúde português através de uma melhoria significativa: Da integração de cuidados de saúde; Da gestão dos percursos das pessoas nos cuidados de saúde, quer em relação à doença aguda quer naquilo que diz respeito a pessoas com múltiplos problemas de saúde e do investimento na capacitação do cidadão na promoção e proteção da sua saúde e na boa utilização dos serviços de saúde. Ainda no âmbito deste projeto, e de forma a promover a literacia em saúde, a área do cidadão do portal sns já disponibiliza o acesso à biblioteca de literacia em saúde e a livros digitais. Esta área disponibiliza igualmente novos plano_individual_de_cuidados que permitem ao cidadão, em conjunto com a sua equipa de saúde, criar um plano com metas bem definidas, tendo como finalidade a promoção da saúde, a prevenção da doença, a prevenção das agudizações da doença crónica e uma melhoria da qualidade_de_vida. O Programa de Incentivo à Integração de Cuidados tem como principais objetivos: Colocar as pessoas, as suas famílias e os seus cuidadores no centro das intervenções no sns; Reforçar a prevenção, o diagnóstico precoce, a continuidade e a proximidade das respostas do sns; levar os níveis de acesso, qualidade e eficiência no sns; Promover o alinhamento, a articulação e a coordenação entre as entidades, mais parcerias com a comunidade; Incentivar a Governação Clínica e de Saúde ao longo do percurso de vida dos cidadãos; Valorizar o envolvimento, a iniciativa e o bom desempenho dos profissional_de_saúde. A área do cidadão do portal sns foi melhorada e aumentada a disponibilização de informação ao cidadão, permitindo uma navegação mais intuitiva, uma mais clara alocação dos serviços eletrónicos por categorias e foi iniciado o processo de revisão dos serviços à luz conceito da centralização no cidadão e no seu percurso de vida, criando a componente de Planos de Cuidados e Percurso de Vida, que será ampliada e corporizada durante 2018. Princípios orientadores do sns_24: O sns_24 se rege pelos princípios e valores diretamente relacionados com a prestação de cuidados no sns, nomeadamente: Foco no utente, centrado nas necessidades do cidadão, privilegiando a atenção e relação personalizada com o cidadão; simplicidade e acessibilidade; integração; simplificação; universalidade e equidade; confidencialidade; proximidade. O sns_24 tem como principais objetivos: Ampliar e simplificar o acesso da população à informação e aos serviços de saúde; Orientar o cidadão para os serviços de saúde mais adequados às suas necessidades, contribuindo para a diminuição de situações de congestionamento dos serviços de saúde, nomeadamente as urgências e os serviços administrativos, marcação de consultas; Promover o envolvimento do cidadão na gestão ativa da saúde, respondendo de forma esclarecedora e em tempo útil às suas necessidades; Colaborar no aumento da eficácia e da eficiência operativa do setor da saúde, promovendo a articulação necessária entre as várias entidades do sns e a integração dos Sistemas de Informação do ministério_da_saúde; Contribuir para uma visão integrada sobre as diferentes medidas e programas de saúde. A implementação do siga sns se encontra em curso, na sequência da publicação da portaria número 147_2017, de 27 de abril, se constituindo como um sistema de acompanhamento, controlo e disponibilização de informação integrada, destinado a permitir um conhecimento transversal e global sobre o acesso à rede de prestação de cuidados de saúde no sns. Se trata de uma abordagem inovadora do acesso ao sns, centrada no cidadão, que permite a monitorização integral do acesso a cuidados de saúde, através da articulação dos diversos níveis, serviços e tipos de resposta, de forma transversal e integrada, permitindo uma visão completa do percurso do utente no sistema, desde a identificação de um problema de saúde, até à sua resolução.

**** *id_57 *doc_2 *ano_2019 *autor_2 *área_3

Os hospitais do sns têm respondido positivamente ao aumento crescente da procura, implementando diversas medidas de reorganização interna que permitam obter melhores resultados em saúde para os utentes e que promovam elevados níveis de eficácia e eficiência na gestão e na governação clínica, com maior transparência, responsabilização, prestação de contas e centralidade no cidadão e na sua família. As melhorias da resposta tiveram por base várias reformas com incidência na organização interna dos hospitais, se destacando a implementação de diversos mecanismos de responsabilização e avaliação, a garantia da melhoria da informação clínica e de gestão, o aprofundamento das relações de parceria e complementaridade entre as várias estruturas do SNS, o reforço da coordenação e a articulação com outros níveis de cuidados e outros agentes, da saúde e sociais, entre outras. O Modelo de Certificação do ministério_da_saúde se operacionaliza em 5 dimensões: o cidadão no centro do sistema_de_saúde; a organização da atividade centrada na pessoa; os profissional_de_saúde; os processos de suporte; os resultados. O processo de contratualização hospitalar de 2018 procurou contribuir para a promoção do acesso ao sns, introduzindo um conjunto de orientações e de medidas inovadoras para a melhoria do desempenho assistencial das instituições do sns, com destaque para as seguintes: Alargar o livre acesso e circulação do utente no sns, diversificando as alternativas e aumentando a sua capacidade de intervir de forma pró_ativa e responsável na gestão do seu estado de saúde e bem_estar; Incentivar a cultura da prestação de cuidados em equipa multidisciplinar e multiprofissional, promovendo a articulação e coordenação entre os profissional_de_saúde e uma resposta centrada no utente e no seu percurso no sns; Consolidar os processos de afiliação e de trabalho em rede colaborativa no sns, centrando a organização dos cuidados nas necessidades das pessoas e promovendo a cooperação. Na perspetiva da promoção da saúde, da integração de cuidados e da centralidade do cidadão no sistema_de_saúde, foi criado através do despacho número 6429_2017, de 25 de julho, o Programa Nacional de Literacia em Saúde e Integração de Cuidados, com o desidrato de promover um sns mais próximo. No decurso do ano de 2016, se concetualizou uma nova abordagem na literacia em saúde e integração de cuidados, se iniciando em 2017 o desenho dos instrumentos organizacionais, informativos e comunicacionais necessários para este fim e criando as condições para que seja possível ensaiar um salto qualitativo significativo no sns: um sns mais próximo, mais proximidade, melhor centrado nas pessoas, mais qualificado, com maior capacidade de resolução. Princípios orientadores do sns_24: o sns_24 se rege pelos princípios e valores diretamente relacionados com a prestação de cuidados no sns, nomeadamente: foco no utente, ir ao encontro das necessidades do cidadão, privilegiando a atenção e relação personalizada; simplicidade e acessibilidade; integração; simplificação; universalidade e equidade; confidencialidade; proximidade.

**** *id_58 *doc_2 *ano_2020 *autor_2 *área_3

Os hospitais do sns têm respondido positivamente ao aumento crescente da procura, implementando diversas medidas de reorganização interna que permitam obter melhores resultados em saúde para os utentes e que promovam elevados níveis de eficácia e eficiência na gestão e na governação clínica, com maior transparência, responsabilização, prestação de contas e centralidade no cidadão e na sua família. As melhorias da resposta tiveram por base várias reformas com incidência na organização interna dos hospitais, se destacando a implementação de diversos mecanismos de responsabilização e avaliação, a garantia da melhoria da informação clínica e de gestão, o aprofundamento das relações de parceria e complementaridade entre as várias estruturas do sns, o reforço da coordenação e a articulação com outros níveis de cuidados e outros agentes, da saúde e sociais, entre outras. O processo de contratualização hospitalar de 2018 procurou contribuir para a promoção do acesso ao sns, introduzindo um conjunto de orientações e de medidas inovadoras para a melhoria do desempenho assistencial das instituições do sns, com destaque para as seguintes: Alargar o livre acesso e circulação do utente no sns, diversificando as alternativas e aumentando a sua capacidade de intervir de forma pró_ativa e responsável na gestão do seu estado de saúde e bem_estar; Incentivar a cultura da prestação de cuidados em equipa multidisciplinar e multiprofissional, promovendo a articulação e coordenação entre os profissional_de_saúde e uma resposta centrada no utente e no seu percurso no sns; Consolidar os processos de afiliação e de trabalho em rede colaborativa no sns, centrando a organização dos cuidados nas necessidades das pessoas e promovendo a cooperação. Princípios orientadores do sns_24: o sns_24 se rege pelos princípios e valores diretamente relacionados com a prestação de cuidados no sns, nomeadamente: foco no utente, ir ao encontro das necessidades do cidadão, privilegiando a atenção e relação personalizada; simplicidade e acessibilidade; integração; simplificação; universalidade e equidade; confidencialidade; proximidade.

**** *id_60 *doc_7 *ano_2017 *autor_2 *área_3

Para melhorar a saúde dos portugueses, é necessário mobilizar as comunidades locais para a proteção e promoção da sua saúde, através de estratégias locais de saúde, amplamente participadas. Para tal é importante investir na capacidade de cada cidadão de tomar decisões informadas sobre a sua saúde, ao longo o seu percurso de vida, literacia em saúde. Melhor literacia em saúde é um fator muito importante para uma boa gestão dos percursos das pessoas no sns e também para conseguir melhores resultados da prestação desses cuidados. Isso significa apostar na centralidade das pessoas no sns do futuro. Mais de 1 terço da população portuguesa tem múltiplos problemas de saúde, amiúde de evolução prolongada. Por isso, estas pessoas são também as utilizadoras mais frequentes dos serviços de saúde. Passarão agora a beneficiar de um plano_individual_de_cuidados de saúde, que facilitará a gestão dos seus percursos através dos serviços de saúde de que necessitam. Cada pessoa poderá participar, com os profissional_de_saúde que prestam cuidados, na identificação dos seus problemas de saúde mais importantes, na definição dos cuidados necessários e na avaliação periódica dos resultados obtidos. Assim o sns_mais_proximidade se centra em duas ideias fundamentais: a integração dos cuidados de saúde e a centralidade do cidadão nos sistema_de_saúde. Se tratando do sns, se optou por abordar primeiro a integração de cuidados de saúde e depois a perspetivar no contexto mais amplo da proteção e promoção da saúde e da prevenção da doença. Com isso se procura, de alguma forma, sinalizar e minimizar a inconveniente separação entre os cuidados de saúde e a saúde_pública, com o relativo isolamento desta última. Mas se trata, principalmente de olhar para os percursos nos cuidados em saúde como ocorrências inseridas num percurso de vida, com passado, presente e futuro, em todas as suas dimensões e potencialidades de bem_estar. A integração de cuidados de saúde tem múltiplas facetas. Aqui se privilegia aquilo que se designa como a gestão dos percursos das pessoas no sns. Isto significa, olhar para o acesso a cuidados de saúde de qualidade, não como uma questão pontual, mas sim numa perspetiva de continuidade, fazer com que as pessoas passem de um serviço ao outro quando dele necessitarem, sem barreiras ou descontinuidades desnecessárias, tendo sempre em vista os resultados que se pretende alcançar. Esta integração de cuidados requer uma efetiva aproximação, intercâmbio e comunicação entre as diversas organizações que prestam cuidados de saúde no âmbito do sns. E também fora deste âmbito como é o caso, nomeadamente, dos serviços sociais. A ideia da centralidade do cidadão no sistema_de_saúde passa, necessariamente, por capacitar as pessoas para tomar decisões mais inteligentes em relação à sua saúde e aos serviços de saúde. Sem isso, dificilmente os sistema_de_saúde conseguirão o desempenho desejável. Finalmente, a ideia da centralidade do cidadão não pode deixar de se refletir na forma como as pessoas são atendidas no sns. É de facto preciso fazer um novo esforço para qualificar os espaços de atendimento no sns, nas suas dimensões física, organizacional, relacional e informacional. Estas são mudanças necessárias e ambiciosas. Não se farão por decreto. Implicam gerir um exigente processo de mudança. Para isso são necessários novos instrumentos de gestão, informação e comunicação, já em preparação há cerca de um ano a esta parte. Os principais dispositivos deste processo de mudança necessitam de ser ensaiados localmente e este processo não é viável sem o interesse e mobilização de todas as lideranças locais. Requerem algum investimento financeiro e terão que ter em conta as assimetrias regionais e locais do país. Nenhum dos componentes do sns_mais_proximidade é inteiramente novo, se inserem em experiências anteriores que agora se expandem, potenciam, e sobretudo, instrumentam e integram melhor. O desenvolvimento do sns_mais_proximidade está condicionado à evolução das reformas setoriais em curso, cuidados de saúde primários, cuidados continuados, cuidados hospitalares e saúde_pública e aos progressos feitos na gestão dos recursos da saúde, humanos, financeiros, técnicos e tecnológicos. Por outro lado, o sns_mais_proximidade serve como um indispensável ponto focal para estas múltiplas inicitativas setoriais, assegurando que elas convergem para permitir uma resposta mais efetiva áquilo que pessoas esperam do seu sns. Só o sns tem de facto a capacidade de conseguir um elevado nível de integração de cuidados como também os articular com as ações que visam proteger e promover a saúde dos portugueses. O sucesso da gestão dos percursos das pessoas nos cuidados de saúde depende, em grande medida, da adoção de um conjunto de instrumentos e procedimentos: Desenvolvimento de um modelo de plano_individual_de_cuidados, a melhoria das condições de conversação e negociação necessárias para a adoção de um plano_individual_de_cuidados, progressos na classificação dos doentes com comorbilidades; Atualização de protocolos colaborativos entre todas as entidades e serviços que participam na realização dos processos de cuidados identificados no plano_individual_de_cuidados; Contribuição do siga: sistema de gestão integrada do acesso aos cuidados de saúde; Importância dos comportamentos das pessoas no sucesso do seu plano_individual_de_cuidados, articulando este processo com o da promoção da literacia em saúde; Avaliação dos resultados da gestão dos percursos, com a participação de todas as entidades envolvidas. O plano_individual_de_cuidados permite registos relativos a situações de saúde consideradas prioritárias para um horizonte temporal acordado entre a pessoa e os profissional_de_saúde que lhe proporcionam cuidados de saúde. Para cada uma dessas situações, o plano_individual_de_cuidados regista a situação atual, os objetivos que se pretendem atingir as ações e comportamentos necessários para o efeito. O plano_individual_de_cuidados faz também o acompanhamento das ações previstas e a avaliação periódica dos resultados, para cada pessoa e para o conjunto das situações seguidas. Assim, o plano_individual_de_cuidados reflete a partilha do trabalho e da responsabilidade entre os profissional_de_saúde e a pessoa, ela própria para conseguir os resultados em saúde pretendidos. O desenvolvimento do plano_individual_de_cuidados incluirá os seguintes aspetos: Acesso fácil a partir do plano_individual_de_cuidados a informação relevante à gestão de cada uma das situações identificadas como objeto de acompanhamento; Alerta em relação às datas de execução das ações previstas no plano de cuidados; Quadro_resumo dos diferentes serviços utilizados ou previstos no plano_individual_de_cuidados; Plena integração do plano_individual_de_cuidados no sistema de informação de saúde personalizada, incluindo a plataforma de dados de saúde. A boa gestão do plano_individual_de_cuidados e os seus resultados dependem em grande parte da qualidade da interação, conversação e negociação, entre as pessoas e os profissional_de_saúde que lhes proporcionam cuidados de saúde. Para assegurar essa qualidade é frequentemente necessário melhorar aspetos críticos da organização da prática clínica nos cuidados de saúde primários, como por exemplo a disponibilidade para promover a narrativa das pessoas sobre a sua situação de saúde, a relação entre o tempo de conversação e o tempo de registo e gestão informática e os recursos humanos e materiais necessários para o efeito. A avaliação da gestão dos percursos das pessoas nos cuidados de saúde terá lugar a 3 níveis: Grau de realização dos objetivos estabelecidos no plano_individual_de_cuidados: esta avaliação é da responsabilidade da equipa que gere o respetivo plano_individual_de_cuidados, nomeadamente a própria pessoa e os profissional_de_saúde que o iniciaram, habitualmente a equipa dos cuidados de saúde primários; Resultados dos plano_individual_de_cuidados em curso em cada agrupamento de centros de saúde: esta avaliação é da responsabilidade do conselho clínico e de saúde dos agrupamento de centros de saúde; Conjunto dos objetivos do sns_mais_proximidade, da responsabilidade do ministério_da_saúde. O cuidar em casa deslocaliza os cuidados das instituições para a casa das pessoas. Isso obriga a uma redefinição do processo de cuidados, que deve ter em conta os seguintes aspetos, que não sendo exclusivos dos cuidados no domicílio, são aqui particularmente relevantes: A casa das pessoas como o local privilegiado de prestação de cuidados e o doente e o seu cuidador como foco dos cuidados; Diversos aspetos associados ao processo de cuidados, destacando, as condições habitacionais e comunitárias, as redes de afetos, as condições socioeconómicas, entre outras; coprodução de cuidados, significando o envolvimento de todos os atores no processo de cuidados; Integração de cuidados, não só entre os diferentes tipos de cuidados, mas também com a segurança social e outros atores na comunidade, em que a coordenação tem um papel particularmente relevante; Pró_atividade e a continuidade dos cuidados através do plano_individual_de_cuidados, como o instrumento privilegiado e expressão da centralidade dos cuidados, do envolvimento do doente e família e da comunicação entre todos os cuidadores. A informação de saúde não pode continuar fragmentada segundo as várias modalidades de cuidados de saúde que chegam a casa das pessoas. O plano_individual_de_cuidados passará a ser o registo comum para todas essas modalidades de prestação de cuidados de saúde e para tal, o seu desenvolvimento durante 2018 deverá permitir que se configure nesse sentido. Quando se põe a enfâse na centralidade do cidadão no sistema_de_saúde, isso significa que para além de este ser o objeto da atenção dos serviços de saúde, ele deve também passar a ser o sujeito, o ator principal, no sistema_de_saúde. Centralidade do cidadão também significa o seu envolvimento e participação naquilo que diz respeito à proteção e promoção da sua saúde e à dos seus concidadãos. Aqui também, para além da bondade do princípio, é necessário instrumentar a sua aplicação. Nem sempre existe nas unidades do sns a preocupação de proporcionar o melhor atendimento possível às pessoas que as procuram. Não existe centralidade do cidadão sem assegurar que este é atendido no sns, em todas as circunstâncias, da melhor forma possível, qualidade das instalações, das relações no atendimento, da informação nos espaços de atendimento e espera. Neste contexto o sns_mais_proximidade, nesta primeira fase do seu desenvolvimento, privilegia essencialmente 3 aspetos associados à centralidade do cidadão no sistema_de_saúde português: uma nova geração de estratégias locais de saúde; a promoção da literacia em saúde; e a qualificação do atendimento no sns. Os 2 primeiros têm a ver com a saúde_pública portuguesa. Existe em Portugal um vasto conjunto de programas de saúde, enquadrados por um plano_nacional_de_saúde. É necessário aprofundar a sua implantação através dos planos locais de saúde. A promoção da literacia em saúde é indispensável para esse fim. É importante notar que a questão da centralidade do cidadão, particularmente na sua vertente de saúde_pública, se situa para além do âmbito do sns. Nesta primeira fase de desenvolvimento do sns_mais_proximidade, as populações abrangidas pelos projetos piloto beneficiarão de uma nova geração de estratégias locais de saúde. Estas, tendo em conta a experiência já havida com planos locais de saúde, dão especial atenção a um conjunto de aspetos particularmente significativos, atualmente, para a proteção e promoção da saúde: Abordar a saúde no decurso dos percursos de vida das pessoas; Incluir a governança local como fator crítico para o desenvolvimento das estratégias locais de saúde, prestar maior atenção à inter_setorialidade, conseguir uma melhor integração na dinâmica das instituição locais, principalmente naquilo que diz respeito ao bem_estar, facilitar e promover a participação das pessoas; Assegurar, desde o início, que os resultados esperados possam ser avaliados a curto e médio prazo. A capacidade de os cidadãos tomarem decisões informadas sobre a sua saúde e sobre a adequada utilização dos serviços de saúde, constitui fator crítico para a promoção da saúde e para uma boa gestão dos percursos nos cuidados de saúde. Daqui o papel que a literacia em saúde desempenha na integração de cuidados de saúde, mas também na proteção e promoção da saúde ao longo do percurso de vida. Para além da emissão de informação de saúde é necessário equipar o recetor, as pessoas, para incorporar e personalizar essa informação. A minha_agenda_de_saúde constitui esse equipamento de personalização, que ajuda a ativar o cidadão a se interessar pela sua saúde e pela boa utilização dos cuidados de saúde. Lhe permite importar, diretamente, conteúdos dos livros digitais temáticos, interagir com múltiplas fontes de informação, incluindo o portal do sns e as mensagens que este passará a emitir, organizando para uso próprio, segundo as suas preferências temáticas e o tipo de uso que deseja dar à minha_agenda_de_saúde. A atual versão da minha_agenda_de_saúde deve ser vista como o primeiro passo num processo de desenvolvimento que tem como finalidade apoiar cada pessoa a encontrar a melhor solução para organizar e gerir a sua informação de saúde. Vários aspetos merecem especial atenção na gestão da mudança necessária para realizar o sns_mais_proximidade: Mobilizar as lideranças locais para a inovação, desenvolvendo novos dispositivos e instrumentos de gestão, informação e comunicação; Promover processos consultivos e participativos, envolvendo os atores sociais da saúde e organizações representativas dos cidadãos; Alinhar a gestão dos recursos humanos e financeiros com os objetivos do sns_mais_proximidade; Fazer a transformação digital do sns como um dos principais veículos para implementação do sns_mais_proximidade; Passar de um número limitado de experiências regionais, necessárias para ensaiar e avaliar muitos dos novos instrumentos indispensáveis neste tipo de mudança, para o conjunto do país; Aprender com a experiência, monitorizando e avaliando processos e resultados, ao mesmo tempo que se promove uma articulação estimulante com iniciativas internacionais semelhantes. O sns_mais_proximidade fará sentir aos seus profissional_de_saúde que dá importância às pessoas, que as suas condições de trabalho e satisfação profissional, contam. Mas é igualmente necessário que os profissional_de_saúde aprofundem formas de atuação e colaboração entre si, centradas na contribuição de cada perfil profissional para realizar melhores resultados em saúde nas pessoas. É indispensável interessar e envolver adequadamente os profissional_de_saúde no desenvolvimento do sns_mais_proximidade. Isso implica entre outras coisas: Proporcionar informação precisa sobre os objetivos e as ações previstas no desenvolvimento dos projetos propostos; Proceder à sua auscultação sobre aspetos do funcionamento dos serviços que podem ser melhorados a curto e médio prazo e que são de importância crítica para o bom desenvolvimento do sns_mais_proximidade; Promover reuniões preparatórias com os profissional_de_saúde envolvidos antes do início da implementação de novos projetos; Estabelecer uma linha verde de comunicação entre os profissional_de_saúde, os dirigentes e a gestão de cada projeto; Não descurar os aspetos relacionados com as exigências da integração de cuidados e da centralidade do cidadão nas ações de formação profissional. A transformação digital do sns está em curso. Nesta transformação há essencialmente 2 aspetos a considerar: o primeiro tem a ver com a digitalização da informação de saúde; o segundo diz respeito à profunda alteração dos processos das relações de trabalho no sns. Esta transformação permite aprofundar e acelerar a recolha contínua, expedita e segura de informação relevante para a saúde e pôr essa informação à disposição das pessoas e dos profissional_de_saúde para que possam tomar decisões inteligentes e oportunas na sua vida de todos os dias. Desta forma a transformação digital deverá constituir um dos principais elementos integradores do sns_mais_proximidade, superando a multiplicação de aplicações informáticas fragmentárias, que não comunicam entre si e dificultam o pleno usufruto por parte dos profissional_de_saúde e dos cidadãos, dos benefícios mais desejados da transformação digital. Estes projetos têm como objetivo apoiar tecnicamente o desenvolvimento do sns_mais_proximidade no período 2017_2018. plano_individual_de_cuidados: Dispositivo de gestão da informação de saúde, com um papel essencial na gestão do percurso das pessoas nos cuidados de saúde. Iniciado na segunda metade de 2016. Uma terceira versão esperada em janeiro de 2018. Classificação dos doentes: Importante para a integração de cuidados e para o plano_individual_de_cuidados cada família dos cuidados de saúde tem a sua própria classificação. Conversação e negociação como ponto de partida para o plano_individual_de_cuidados. Vários aspetos da rotina da consulta nos cuidados de saúde primários, tendem a limitar o pleno desenvolvimento do tipo de conversação e negociação necessária para o plano_individual_de_cuidados. Cuidar em casa: Integrar todos os aspetos de cuidados de saúde ao domicílio e assegurar a sua efetiva coordenação. Estratégias locais de saúde: É indispensável estabelecer um conjunto de orientações atualizadas e desenvolver instrumentos de gestão, informação e comunicação que facilitem a sua elaboração, monitorização e avaliação de estratégias locais de saúde. Biblioteca digital, incluindo livros digitais temáticos: A biblioteca, no que diz respeito à plataforma informática, estará estabilizada no início de 2018. Até ao final de 2018 contará com uma coleção de cerca de 20 livros digitais temáticos. minha_agenda_de_saúde: Esta agenda é um elemento central na estratégia da promoção da literacia: iniciada em 2017, necessita ser melhor integrada na área do cidadão e de comunicar melhor com os restantes dispositivos de personalização da informação de saúde. São necessários ensaios de ativação com distintos mediadores, sns, farmácias, bibliotecas e escolas, em 2017 e 2018. Qualificação dos espaços de atendimento do sns: Iniciado em 2016, mas com poucos progressos em 2017. Necessita de um novo começo em 2017. Requer um melhor reconhecimento das boas práticas existentes, da institucionalização dos responsáveis pela qualidade do atendimento em todas as unidades do sns, novos instrumentos de informação e comunicação e de um módulo de apoio, valorização capacitação daqueles que, nas receções das unidades de saúde, atendem o público. Integração dos instrumentos de informação personalizada sobre cuidados de saúde, incluindo o plano_individual_de_cuidados e a minha_agenda_de_saúde. É importante para a coerência do sistema informação de saúde e para aquelas que o utilizam, profissional_de_saúde e cidadão, que se inicie este projeto natureza integradora com metas calendarizadas para 2017 e 2018. Avaliação: Este projeto se destina a desenhar e implementar um processo de avaliação para o sns_mais_proximidade, que inclua a avaliação tanto de processos como de resultados. Iniciado em 2016_2017, para continuidade em 2018_2019.

**** *id_61 *doc_5 *ano_2023 *autor_3 *área_1

Os cuidados paliativos, enquanto cuidados holísticos, desempenham um papel vital no curso das doenças crónicas e ameaçadoras da vida de muitos portugueses, melhorando a qualidade_de_vida para estas pessoas, mas também para os seus cuidadores, familiares e amigos que os apoiam. São cuidados ativos, aplicáveis em qualquer idade, em diferentes doenças e devem estar disponíveis em todos os níveis de cuidados de saúde. Portanto, no contexto português, e em alinhamento com a oms, se define os cuidados paliativos como: Os cuidados paliativos são uma parte crucial dos serviços de saúde integrados e centrados nas pessoas. Aliviar o sofrimento, seja ele físico, psicológico, social ou espiritual, é uma responsabilidade ética global. Assim, quer a causa do sofrimento seja a doença cardiovascular, o cancro, a falência grave dos órgãos, a tuberculose resistente aos medicamentos, as queimaduras graves, as doenças crónicas em fase terminal, os traumas agudos, a prematuridade extrema à nascença ou a extrema fragilidade da velhice, os cuidados paliativos podem ser necessários e têm de estar disponíveis em todos os níveis de cuidados. Visão: Todas as pessoas com doença limitante de vida recebem os cuidados de que precisam, no momento em que deles necessitam, para viver com melhor qualidade_de_vida. Princípios Orientadores: Cuidados paliativos são cuidados centrados na pessoa, sua família e cuidadores e se baseiam na comunicação eficaz, na tomada de decisão compartilhada, na autonomia pessoal e se prolongam no processo de luto; Os cuidados paliativos devem estar disponíveis e serem prestados a todas as pessoas que vivem com uma doença ativa, avançada e progressiva, independentemente do diagnóstico; Os cuidados paliativos afirmam a vida enquanto reconhecem que morrer é uma parte inevitável da vida; Os familiares e cuidadores são valorizados e recebem os cuidados que se adequam às suas necessidades; Os cuidados são especializados e baseados em evidência científica. As pessoas com necessidades de saúde complexas necessitam de cuidados de múltiplos prestadores em vários ambientes de saúde, pelo que o risco de receberem cuidados fragmentados é elevado. No processo atual de reorganização do sns, é imprescindível a agregação das Equipas de Cuidados Paliativos nos Serviço Integrado de Cuidados Paliativos das Unidades Locais de Saúde garantindo a prestação de cuidados de forma integrada à pessoa com necessidades paliativas e sua família, centrados nas suas necessidades, com inclusão dos mesmos nas tomadas de decisão, perspetivando a simplificação de processos, a qualificação das respostas e melhoria dos resultados. Os cuidados paliativos, como cuidados coordenados e personalizados, reconhecem as necessidades individuais das pessoas, possibilitando que desenvolvam conhecimentos, capacidades e a confiança de que necessitam para tomar decisões informadas e gerir de forma mais competente o seu projeto de saúde. Assim, existem 4 princípios para o desenvolvimento dos cuidados centrados na pessoa: proporcionar às pessoas dignidade, compaixão e respeito; oferecer cuidados, apoio e tratamento coordenados; oferecer atendimento, suporte e tratamento personalizado; apoiar as pessoas no reconhecimento e desenvolvimento dos seus próprios pontos fortes e competências para os capacitar a viver uma vida independente e plena. Deste modo, incorporar a experiência da pessoa doente, da família e do cuidador, é parte integrante e essencial da prestação de cuidados de alta qualidade e, como tal, a estratégia deste Plano Estratégico para o Desenvolvimento dos Cuidados Paliativos é que os Cuidados Paliativos devem ser disponibilizados universalmente no sns como um direito inerente à condição humana, intimamente ligado ao Direito à Saúde consagrado na Declaração Universal dos Direitos Humanos. O papel desempenhado pelos familiares e cuidadores da pessoa com necessidades paliativas é indispensável, insubstituível e complementar aos cuidados prestados pelas equipas locais de cuidados paliativos. Num propósito de capacitação, para que estes familiares e cuidadores assumam maior nível de consciencialização sobre: a dimensão da doença; objetivos de cuidados; princípios e filosofia dos cuidados paliativos e sobre os recursos disponíveis, é necessário que as suas necessidades psicológicas, emocionais e espirituais sejam também atendidas. Eixo Prioritário Cuidados Centrados na Pessoa: Elaboração Critérios Referenciação para Equipas Especializadas de Cuidados Paliativos Adultos; Elaboração Critérios Referenciação para Equipas Especializadas de Cuidados Paliativos Pediátricas; Plano de Cuidados Pediátrico Proposta de modelo de plano de cuidados para utilização pelas equipas; Plano de Cuidados Adultos Integração no grupo de trabalho da Administração Central do sistema_de_saúde sobre Integrated care for complex chronic patients: Personalized Care Action Plans.

**** *id_62 *doc_4 *ano_2013 *autor_1 *área_1

A alta hospitalar ocorre no momento em que a puérpera deixa o hospital com destino ao domicílio ou ao local onde planeou o seu pós_parto. A maioria dos autores consultados define alta precoce quando esta ocorre antes das 48 horas pós_parto. Para que isto ocorra, o hospital e os recursos na comunidade devem desenvolver um robusto sistema de apoio que assegure que a transferência das mulheres e seus bebés para casa ou para o local onde vão ser apoiados seja centrada na mulher ou casal e nas suas necessidades, contribuindo para uma transição suave para o exercício da parentalidade. Melhorar as redes de articulação entre os diferentes níveis de cuidados que permitam incentivar e apoiar a transferência de cuidados da pessoa com doença crónica do hospital para a comunidade. Implementação de um modelo de prestação de cuidados centrado no cidadão, com níveis de oferta ajustados às necessidades efetivas em saúde. A compartimentação das unidades de saúde, que impede que o doente circule agilmente pelo sns, deve ser combatida. A pessoa deve poder circular com agilidade entre os cuidados de saúde primários, entrada no sistema, e as unidades de cuidados secundários e terciários, tendo um profissional que o acompanha no percurso, se sugere o enfermeiro que o acompanha nos cuidados de saúde primários ou o enfermeiro de família, quando criado.

**** *id_67 *doc_2 *ano_2021 *autor_3 *área_3

A finalidade do presente texto é a de contribuir para prospetivar um sns atento aos desafios atuais e futuros, centrado no cidadão, nos seus percursos de vida e de saúde, nas comunidades, na saúde_pública. As principais fragilidades residem na dispersão, fragmentação e desconexão de muitos dos investimentos previstos. A concretização das mudanças necessárias e desejadas depende certamente da cultura e das competências de gestão e de governação institucional dominantes. A expressão integração de cuidados centrada na pessoa tem sido largamente utilizada, embora com fraca concretização sistémica. No entanto, a nível de alguns projetos locais já em curso e da ação de equipas de saúde no cuidado a pessoas concretas, é possível identificar processos, procedimentos e práticas alinhadas e ilustrativas daquele conceito. A pessoa, o doente, o cidadão é uma entidade infinitamente heterogénea. Essa imensa diversidade e heterogeneidade diz respeito a: fase de vida, circunstâncias biológicas e funcionais, psicossociais, familiares, socioeconómicas, sócio ocupacionais, socioculturais, existenciais e de condição de saúde e bem_estar. As experiências vividas em serviços e unidades com equipas multiprofissionais dedicadamente pró_ativa, evidenciam atitudes e práticas complexas e variáveis para mobilizar meios e cuidados necessários para cada doente num dado momento e situação, com colaboração e passagens de testemunho na transição entre equipas, serviços e unidades. O problema essencial é que se deparam com barreiras e obstáculos ao adequado cumprimento da sua missão. Os casos bem_sucedidos, onde foi possível remover montanhas, ilustram que a função de gestão de caso é, naturalmente, móvel, dinâmica, flexível e profundamente entranhada no núcleo de competências de cada profissional e equipa. Emerge da qualidade técnica e científica e organizacional de cada equipa. Não é suscetível de ser fixada rigidamente num profissional. Cabe aos decisores e aos organizadores sistémicos facilitar a vida dos profissional_de_saúde e das equipas, em vez de lhes dificultar a ação e de lhes limitar a efetividade. O processo clínico eletrónico pessoal único, centrado no cidadão e controlado por este, é um elemento_chave para a gestão dos percursos de saúde de forma integrada e coordenada. Este conceito ultrapassa a atual conceção de registo_de_saúde_eletrónico, que funciona como repositório de informação clínica, de várias fontes, fragmentada, sem acessibilidade ao cidadão. Uma componente essencial do processo clínico eletrónico pessoal único é o resumo clínico eletrónico. Por sua vez este Resumo conterá o processo_assistencial_integrado ou plano_individual_de_cuidados. O novo processo deverá ser acessível ao próprio cidadão, e em todos os pontos da rede de prestação, sempre com autorização deste. A participação das populações e das diversas organizações de cidadãos é importante para identificar necessidades e potenciais respostas, mas é também fundamental reconhecer que o motor da transformação reside nos profissional_de_saúde e na sua determinação para implementar as melhores práticas no cuidado às pessoas e famílias e para incrementar a saúde coletiva.

**** *id_68 *doc_2 *ano_2022 *autor_3 *área_1

A ideia da saúde centrada nas pessoas pouco mais vai sendo que um lema sem ancoragem na realidade ou então uma meta, sincera talvez, que fica longe. Assim, se preconiza um modelo de cuidados que responda simultaneamente a 3 princípios fundamentais: garanta cuidados centrados na pessoa, integração e continuidade de cuidados. Dito por outras palavras, a domiciliação de cuidados pode ser, simultaneamente, uma forma de garantir o envelhecimento em casa, elevados níveis de satisfação com os cuidados e redução de custos. Resumimos alguns dos pressupostos dessa redefinição: assumir o doente e o seu cuidador como foco dos cuidados; assumir o cuidador informal, também, como parceiro de cuidados; assumir a coprodução de cuidados como garante do envolvimento dos diversos atores no processo de cuidados; assumir o plano_individual_de_cuidados como o instrumento privilegiado e expressão da centralidade dos cuidados, do envolvimento do doente e família e da comunicação entre todos os cuidadores; garantir a integração de cuidados; garantir a permanência e a continuidade dos cuidados e fazer uma gestão pró_ativa das situações através da adoção da figura de coordenador de cuidados; utilização de recursos tecnológicos e de telecuidados, devidamente integrados no modelo de cuidados. Tudo isto integrado numa rede de respostas que garanta a equidade no acesso. Tal como já foi referido, a domiciliação de cuidados, cuidar em casa, tem potencial para incrementar a satisfação com os cuidados, promover o envelhecimento em contexto familiar e, por esta via, contribuir para melhores indicadores de qualidade_de_vida e de saúde. Todavia, para isso precisamos dispor, não apenas de uma visão política estratégica que defina e regule o processo sob múltiplas perspetivas, mas também um modelo de cuidados que garanta que os mesmos são centrados na pessoa e sua família, são integrados e contínuos. A aplicação crescente de tecnologias digitais no setor da saúde tem permitido a implementação de modelos de cuidados mais centrados no utente, promotores de integração de cuidados, de que são exemplo os programas de autogestão do processo de saúde_doença e os cuidados domiciliários, cujos benefícios ao nível da experiência do utente, redução de custos e manutenção da qualidade e segurança têm sido amplamente reconhecidos. Centralidade no cliente: monitorização da experiência dos utentes e previsão das suas necessidades, na área da saúde este ponto pode ser promovido através da utilização de medidas de resultados e experiências relatados pelo utente, bem como o seu envolvimento no desenho das tecnologias digitais. Um dos modelos assistenciais que tem surgido no sentido de prestar cuidados mais centrados no utente, com benefício custo_efetivo é a hospitalização domiciliária. O princípio base da hospitalização domiciliária é o tratamento de utentes com doença em fase aguda ou crónica agudizada que requerem cuidados de nível hospitalar, mas que reúnem condições para ser tratados no seu domicílio. A saúde digital, ao permitir armazenar, analisar e partilhar informação relevante, promove o estabelecimento de uma rede de partilha e colaboração, fundamental na implementação de projetos de integração de cuidados, contribuindo para a prestação de cuidados mais centrados no utente, atendendo às suas necessidades específicas, e para a melhoria da efetividade, eficiência e qualidade dos cuidados. Mais uma vez afirmamos que não faz sentido apenas aumentarmos o número de respostas sem previamente definirmos uma estratégia construída com base nos princípios já referidos: cuidados centrados na pessoa e família, integrados e que garantam continuidade. Por tal razão, começamos por um breve enquadramento concetual, onde se aborda a necessidade da definição de um modelo de cuidados domiciliários. Dado o seu caráter estrutural e, ao mesmo tempo, a sua promessa de futuro, dedicamos um capítulo à inovação tecnológica e terapêutica. De facto, a inovação, de forma ampla, e a digitalização em particular são sistematicamente apresentadas como um contributo inquestionável para o incremento da capacidade de resposta do sns aos desafios com que se confronta. Porque tal como se afirma a saúde digital, ao permitir armazenar, analisar e partilhar informação relevante, promove o estabelecimento de uma rede de partilha e colaboração, fundamental na implementação de projetos de integração de cuidados, contribuindo para a prestação de cuidados mais centrados no cidadão, atendendo às suas necessidades específicas, e para a melhoria da efetividade, eficiência e qualidade dos cuidados. Porém, sendo isto verdade, também precisamos apelar à história e recordar o que correu menos bem com a primeira vaga de digitalização da saúde e que ainda persistem. Nos referimos a um sistema de informação em saúde com proliferação de aplicativos e evidentes lacunas de interoperabilidade entre níveis de cuidados e que, em boa verdade, pouco contribui para a integração e continuidade de cuidados.

**** *id_69 *doc_2 *ano_2020 *autor_3 *área_1

É necessária uma mudança de paradigma cultural: todos os intervenientes no sistema_de_saúde devem reconhecer o papel importante do cidadão enquanto principal interessado, mas também parceiro, que pode contribuir, com o seu conhecimento e evidência de experiência de vida, a todos os níveis do sistema. Promover contextos onde o envolvimento e a participação dos cidadãos possam ser facilitados, desenvolvendo uma cultura verdadeiramente centrada na pessoa e nas comunidades. Isso implica criar mecanismos e espaços participativos, responsivos às necessidades dos cidadãos. Instituir maior transparência a todos os níveis do sistema_de_saúde, disponibilizando, aos cidadãos, acesso a informações claras e aos seus próprios dados de saúde. Incentivar a capacitação de todos os intervenientes nos processos participativos em saúde: cidadãos e seus representantes, incluindo as suas famílias, profissional_de_saúde, decisores e governantes. Desenvolver mecanismos que incentivem a aplicação dos enquadramentos legais existentes, designadamente considerar a existência de incentivos. Os enquadramentos legais são importantes, mas a sua implementação tem sido insuficiente, como é observável pelo ainda débil funcionamento dos Conselhos da Comunidade dos Agrupamento de Centros de Saúde e dos Conselhos Consultivos dos hospitais ou pela ausência de regulamentação da Carta da Participação Pública em Saúde. Considerar a existência de financiamento específico e sustentado para a promoção da participação pública em saúde, implementando os mecanismos e canais necessários e adequados, e assegurando também a independência das organizações representativas dos cidadãos que participam. Avaliar os processos participativos, para obter evidência sobre as melhores práticas nesta área, o que poderá, por seu turno, contribuir para a mudança de paradigma que se pretende promover. A promoção de uma cultura e literacia em participação pública implica que, por um lado, os cidadãos entendam a importância do seu papel nos processos participativos e, por outro, que os diferentes níveis de decisão e governação, onde essa participação deverá ser incentivada, estejam sensibilizados e capacitados para promover e gerir esses processos. A oms tem vindo a reconhecer o papel fundamental dos cidadãos nos processos de planeamento e decisão em saúde, ao contribuírem com a sua experiência de vida diária, com ou sem existência de doença, que constitui evidência adicional aos dados técnicos e científicos. Todos os atores do sistema_de_saúde devem compreender e pôr em prática este reconhecimento de que o cidadão detém um conhecimento importante e válido, que deve ser partilhado com profissional_de_saúde, técnicos e decisores como parte dos processos de coconstrução da saúde. Do ponto de vista da organização dos processos participativos, é importante ter em conta que, na maioria das situações, os cidadãos poderão não participar individualmente, mas através de organizações formais que os representam, como organizações diversas da sociedade civil, associações de doentes, organizações representativas dos utentes ou consumidores. É necessário promover contextos onde este envolvimento é facilitado, desenvolvendo uma cultura verdadeiramente centrada na pessoa. Isso implica criar mecanismos e espaços participativos, responsivos às necessidades dos cidadãos. A nível organizacional, esta parceria com os utentes, famílias e comunidades na conceção de processos, serviços e políticas deverá orientar a estruturação das instituições e do sistema_de_saúde, para que reflitam melhor as suas expectativas e necessidades e se promovam oportunidades específicas para o seu envolvimento, garantindo mais ganhos em saúde e possibilitando melhores experiências para todos os intervenientes. Mudanças legislativas e normativas podem aumentar o envolvimento dos cidadãos e das comunidades, melhorando os mecanismos institucionais para aumentar a participação dos cidadãos e comunidades. Contudo, o estabelecimento de enquadramentos legais é insuficiente para assegurar a participação pública, em particular se não forem proporcionadas as condições necessárias à sua existência, designadamente a capacitação dos atores envolvidos nos processos participativos e a existência de financiamento específico para a participação. O desenvolvimento e o alinhamento de políticas e incentivos poderão encorajar cidadãos, famílias, profissional_de_saúde e organizações e sistema_de_saúde a facilitarem e promoverem a participação em saúde. Portugal apresenta um enquadramento legislativo bastante robusto nesta área, uma vez que a participação e a participação em saúde estão previstas a diferentes níveis da legislação: Constituição, Lei de Bases da Saúde e Lei da Participação Pública em Saúde. No entanto, embora os fundamentos legais existam, a implementação continua a ser insuficiente, como no caso dos Conselhos da Comunidade dos Agrupamento de Centros de Saúde e dos Conselhos Consultivos dos hospitais, que não funcionam de forma efetiva, na maioria das situações. Adicionalmente, a ausência de regulamentação da Carta da Participação Pública em Saúde impede a sua aplicação prática e efetiva. Para que exista uma participação efetiva dos cidadãos e a tradução real das suas expectativas e necessidades nos processos participativos é necessário assegurar que quem participa, designadamente as organizações da sociedade civil, o faz num contexto de independência. Como tal, é desejável que se estabeleçam mecanismos de financiamento específicos e adequados aos processos participativos, assegurando que a participação destas organizações não fica dependente de outras fontes de financiamento que possam determinar a existência de conflitos de interesses.

**** *id_70 *doc_4 *ano_2018 *autor_2 *área_1

Os processo_assistencial_integrado são processos organizacionais que colocam o cidadão, com as suas necessidades e expectativas, no centro do sistema. A continuidade assistencial e a coordenação entre os diferentes níveis de cuidados, são reconhecidos como elementos essenciais para garantir que o doente recebe de forma atempada e efetiva os melhores cuidados de saúde. A abordagem dos processo_assistencial_integrado é uma abordagem multidisciplinar, integral e integrada que pressupõe a reanálise de todas as atuações de que o doente é alvo em qualquer ponto do sistema_de_saúde, do início ao fim do processo_assistencial_integrado. Por outro lado, as atividades assistenciais baseadas na melhor evidência científica disponível, respeitam o princípio do uso racional de tecnologias da saúde e orientam a adoção de atuações terapêuticas custo_efetivo, ao mesmo tempo que se garante ao cidadão a qualidade clínica que é consagrada como um dos seus principais direitos. Se pretende proporcionar a mudança organizacional, com base no envolvimento de todos os profissional_de_saúde implicados na prestação de cuidados, quer a nível hospitalar, quer a nível dos cuidados primários ou continuados, acreditando na sua capacidade e vontade de melhorar continuamente a qualidade e de centrar os seus esforços nas pessoas. Os processo_assistencial_integrado são ainda uma ferramenta que permite analisar as diferentes componentes que intervêm na prestação de cuidados de saúde e ordenar os diferentes fluxos de trabalho, integrando o conhecimento atualizado, homogeneizando as atuações e colocando ênfase nos resultados, a fim de dar resposta às expectativas quer dos cidadãos quer dos profissional_de_saúde. É necessário orientar a prática clínica para os resultados e para a qualidade, tendo também como objetivo a utilização mais efetiva dos recursos. A gestão da prática clínica, tal como definida nos processo_assistencial_integrado, apela para uma melhor organização dos cuidados prestados às pessoas pelo sistema_de_saúde, os centrando no cidadão e, neste caso particular, nas necessidades e expectativas da pessoa com asma e na antecipação e planeamento integral da continuidade de cuidados.

**** *id_81 *doc_5 *ano_2024 *autor_2 *área_3

A organização e o funcionamento dos cuidados de saúde prestados pelo sns têm vindo a acrescentar à melhoria cumulativa da qualidade técnica e científica, a preocupação com a centralidade da pessoa, sejam os utentes do sns ou os seus profissional_de_saúde, e com os espaços destinados à prestação de cuidados de saúde. Esta preocupação impõe a capacitação para o exercício de práticas éticas, deontológicas, técnicas e científicas, para uma consequente melhoria da qualidade, eficácia e eficiência do sns. E requer o desenvolvimento e aprofundamento da humanização dos cuidados de saúde no sns. A preocupação com o reforço da humanização dos cuidados de saúde é o resultado de uma exigência maior por parte dos cidadãos em geral e dos utentes, mas também dos profissional_de_saúde quando valorizam a dimensão humana presente na prestação de cuidados de saúde e o valor desta dimensão para o bem_estar das pessoas. Uma das respostas do sns à preocupação com a centralidade da pessoa e o consequente desenvolvimento da humanização, teve lugar em 2019, com a elaboração do Compromisso para Humanização Hospitalar, por um Grupo de Trabalho criado pela Coordenação para a Reforma do sns na Área dos Cuidados de Saúde Hospitalares. O compromisso foi aprovado pelo ministério_da_saúde e subscrito por todos os hospitais e centros hospitalares portugueses, incluindo as unidades hospitalares privadas.

Por força do decreto_lei número 102_2023 de 7 de novembro, e nos termos previstos no Estatuto do sns, aprovados pelo decreto_lei número 52_2022 de 4 de agosto, foi adotado o modelo de organização e funcionamento em unidades locais de saúde, para integrar os hospitais, centros hospitalares e agrupamentos de centros de saúde. Por este decreto_lei, foi também aprovado um modelo de gestão integrado da prestação de cuidados de saúde aos utentes do sns, incorporando, na mesma entidade pública empresarial, duas dimensões jurídicas e organizativas, os cuidados de saúde hospitalares e os cuidados de saúde primários. Esta alteração visa a prestação integrada de cuidados de saúde primários e hospitalares, o reforço dos cuidados primários na resposta de proximidade e continuidade na assistência em saúde e a aposta na promoção da saúde. Esta nova realidade organizativa, gestionária e funcional tem como desiderato final contribuir, de forma mais efetiva, para a organização das respostas em saúde em função das pessoas, ou seja, da centralidade da pessoa como vertente determinante na definição da organização e funcionamento do sns. Para cumprir a sua missão, a Comissão Nacional para a Humanização dos Cuidados de Saúde sns irá desenvolver a sua ação, elegendo, como pilares essenciais: o respeito pela dignidade da pessoa; o reconhecimento da individualidade, humanidade e singularidade de cada utente e de cada profissional, com correlativo respeito pela autonomia, intimidade, crenças, valores, sentimentos, estados emocionais e circunstâncias pessoais; o reconhecimento da centralidade da pessoa e da pessoa doente em todas as ações do âmbito da saúde; a vulnerabilidade da pessoa doente, em termos de equilíbrio emocional e ou físico; a relevância da empatia, escuta ativa e compaixão no relacionamento dos profissional_de_saúde com os utentes e entre si; a relevância da adequação e das condições dos espaços físicos à tipologia dos cuidados de saúde prestados; a relevância da disponibilidade de recursos humanos e materiais em número e qualidade ajustados às necessidades. Para a definição dos objetivos major da ação a desenvolver pela Comissão Nacional para a Humanização dos Cuidados de Saúde sns, o plano de ação terá em consideração as atribuições que lhe foram cometidas por força da deliberação que a criou: Promover e dinamizar a reflexão sobre os ganhos de qualidade e eficiência que advêm de uma prestação de cuidados de saúde centrados na pessoa e no respetivo percurso de saúde e episódios de doença no sns; Promover e dinamizar a reflexão sobre a centralidade dos profissional_de_saúde nos processos de mudança conducentes a uma cultura organizacional centrada em práticas clínicas e de relações interpessoais e interprofissionais humanizadas; Fomentar ações que estimulem e consolidem o bom relacionamento interpessoal e interprofissional nos locais de trabalho, e o compromisso com uma constante melhoria dos processos e dos resultados em saúde; Identificar boas práticas de humanização de cuidados de saúde já em curso no País e promover ações destinadas a ampliar o seu conhecimento e disseminação; Fazer um levantamento de necessidades, estabelecer prioridades e definir e apoiar intervenções de melhoria adequadas a cada situação, em interação próxima com os responsáveis das instituições e no respeito pela autonomia que lhes cabe; Promover ações de formação no âmbito da humanização em saúde e proporcionar o contacto com experiências e resultados alcançados noutros países; Fomentar a criação de canais de comunicação entre as instituições do sns dedicadas à divulgação e partilha de iniciativas e programas do âmbito da humanização dos cuidados de saúde; Criar e manter ativo um Fórum de Reflexão sobre a humanização em saúde; Apoiar as unidades locais de saúde em iniciativas destinadas a desenvolver e aprofundar a humanização dos cuidados de saúde; Fomentar e apoiar iniciativas que congreguem a participação de diversas instituições e patamares de prestação de cuidados de saúde; Realizar avaliações periódicas sobre os progressos registados no âmbito da humanização em saúde. O presente plano de ação tem como objetivo maior definir os trajetos e as atividades conducentes à melhoria contínua da humanização dos cuidados de saúde prestados nos serviços de saúde do sns. Como plano de ação desenhado para fomentar a humanização no âmbito dos cuidados de saúde prestados aos utentes do sns, por profissional_de_saúde, valoriza: a centralidade da pessoa no seu trajeto como utente de serviços de saúde do sns, ou sejam, as suas perceções, vivências, necessidades e expectativas; os profissional_de_saúde como agentes de uma prestação de cuidados de saúde humanizados e os cuidados que lhes devem ser dirigidos; o respeito e a cordialidade nas relações humanas entre os utentes, seus familiares ou acompanhantes e os profissional_de_saúde que trabalham nas instituições de saúde; o respeito e a cordialidade na relação entre todos os profissional_de_saúde que trabalham nas instituições de saúde; o local em que ocorre a relação: a cultura organizacional; a visão gestionária e a governação; a informação e a comunicação; as infraestruturas e a sua adequação. Os objetivos pretendidos com a atividade da comissão, devem visar resultados que sejam antevistos como genericamente aceites pelas partes. Avaliar a capacidade de resposta da Comissão Nacional para a Humanização dos Cuidados de Saúde sns, balizar a sua atividade e criar condições que potenciem a sua ação; Promover uma cultura de humanização dos cuidados de saúde no sns: a humanização dos cuidados de saúde prestados por instituições do sns como valor organizacional; a adoção pelos órgãos de gestão de compromissos dirigidos para a satisfação dos utentes e familiares; o acompanhamento do utente por um familiar ou acompanhante por ele designado face à sua mais_valia para o diagnóstico, para a terapêutica e em termos de apoio e conforto; Fazer o levantamento de atividades de humanização já implementadas nas instituições e de necessidades a colmatar; Conhecer e caracterizar as boas práticas de humanização já em curso e proceder à sua disseminação; Criar uma equipa com formação específica para tratamento dos dados obtidos com os inquéritos aplicados para conhecimento da realidade, como sejam as boas práticas e as necessidades; Proceder ao mapeamento de estruturas institucionais e não institucionais associadas a dinâmicas de humanização dos cuidados de saúde, que possam concorrer sinergicamente para a implementação de procedimentos humanizadores: Serviços de Humanização, Comissões de Humanização, Gabinetes do Cidadão; Ligas de Amigos, Grupos de Voluntariado, Associações de Doentes; Fomentar uma identidade institucional na linha da humanização dos cuidados: Estimular a elaboração de uma Carta de Humanização da instituição de saúde, a aprovar pelo respetivo Conselho de Administração, vinculativa de todos os agentes de saúde na instituição; Fomentar a articulação com o Provedor do doente e utente nos serviços de saúde em que exista, divulgar a sua existência e promover sinergias decorrentes das respetivas atividades; Sensibilizar para a implementação de ferramentas e mudanças locais que facultem e promovam o desenvolvimento operacional da humanização: Realização de inquéritos de satisfação, com modelos testados, dirigidos para utentes e doentes e familiares durante os seus trajetos em serviços do sns, bem como para os profissional_de_saúde; Comunicação melhorada no sns, mais humanizada e eficaz, através da escuta ativa dos utentes, estimular a conversação com o utente e ouvir e valorizar o que é dito; Criação de canais de comunicação para os utentes de cada instituição de saúde, acessíveis e ágeis, baseados nas novas tecnologias de informação e comunicação; Integração de um representante dos utentes na Comissão de Humanização das instituições de saúde; Promover ações de formação no âmbito da humanização dos cuidados de saúde: Fomentar a formação dos profissional_de_saúde dirigida para a melhoria de competências promotoras da centralidade da pessoa; Sensibilização dos profissional_de_saúde sobre os direitos e deveres dos utentes do sns; Fomento da criação ou mobilização de equipas de teatro para representação de peças existentes ou escritas que expressem situações concretas de falta de humanização e respetiva correção. Promover e apoiar iniciativas e projetos inovadores de intervenção institucional e junto das populações: Fomentar mudanças relevantes para a humanização, a nível das infraestruturas dedicadas à prestação de cuidados de saúde, organização da prestação, meios materiais e humanos, e ambiente laboral; Promover o acesso a medidas de prevenção e tratamento do burnout por parte dos profissional_de_saúde; Implementação de uma cultura de melhoria contínua com foco na melhoria do acesso e no envolvimento dos utentes; Melhoria do percurso e da experiência dos doentes e utentes e garantia de que a mesma é semelhante nas diferentes instituições de saúde; Promoção do acompanhamento dos utentes dos serviços de saúde por um familiar ou pessoa por ele indicada. Organizar encontros de nível nacional: Para partilha de práticas em curso nas instituições de saúde e de dificuldades sentidas; Reflexão e procura conjunta de soluções; Contacto com personalidades convidadas, nacionais ou internacionais, com trabalho reconhecido na área da humanização de cuidados de saúde; Criar e manter ativo um fórum de reflexão permanente, sobre a humanização em saúde, motivando as comissões de humanização e os gabinetes do cidadão a aderirem; Criar um prémio anual para a melhor prática de humanização, com regras claras e disseminação da boa prática pelas restantes unidades locais de saúde; Promover a qualidade da informação disponibilizada aos utentes e familiares, como componente da cultural organizacional e via para a sua participação ativa na vida das instituições de saúde; Definir, redigir e disponibilizar conteúdos de apoio a intervenções de melhoria do âmbito da humanização dos cuidados de saúde, e apoiar a produção de normas de ação pelas instituições; Participar em iniciativas internacionais que permitam conhecer e implementar práticas inovadoras e novos modelos de abordagem dos processos de humanização. Personalização do cuidar e do tratar: o profissional_de_saúde valoriza e pratica a empatia e a escuta ativa, e trata o outro como um seu igual em dignidade; o doente é tratado pelo nome que deseja, com simpatia, cortesia e lealdade, como direito e dever de todos; é proporcionado a cada doente o nome de um profissional, condição primordial para a criação de um vínculo de diálogo e de um vínculo de confiança, privilegiados e invioláveis; é possibilitado aos doentes e ou aos seus cuidadores, o acompanhamento do evoluir da doença através da informação dada pelos profissional_de_saúde, de forma sensível e ajustada ao patamar de compreensão e momento psicológico, devendo os utentes ser capacitados para a tomada de decisões informadas; existem condições para proporcionar cuidados domiciliários e cuidados pós_alta hospitalar e, quando indicado, é dado apoio empenhado e robusto no acesso a cuidados continuados; no exercício quotidiano de um hospital ou de uma unidade de cuidados de saúde primários são considerados os ditames da Carta de Direitos e Deveres dos Doentes e, bem assim, da Carta dos Direitos das Crianças Internadas. Psicologia positiva, promoção da saúde e bem_estar: cria condições para a aplicação de técnicas de psicologia positiva na gestão de recursos humanos, estímulo ao trabalho colaborativo, motivação, recompensa, e promoção da saúde e bem_estar dos recursos humanos em contexto laboral. Normas de relacionamento interpessoal e interprofissional: dispõe de normas de comportamento para o relacionamento entre os profissional_de_saúde, baseados no respeito de cada um por si e pelo outro, na partilha de informação relevante para a prestação de cuidados multiprofissionais, nos deveres de cidadania, na deontologia profissional e nas regras que decorrem das relações laborais. Formação profissional: os profissional_de_saúde se obrigam, porque indispensável, a criar disponibilidades para a formação; assegura e cria, na base de uma ponderada gestão de recursos humanos, oportunidades para a formação profissional ao longo da vida que considere necessária para a melhoria do exercício profissional e respetiva humanização; especificamente, assegura e cria, formação em comunicação, empatia e relacionamento interpessoal. Avaliação da satisfação: monitoriza regularmente a satisfação dos recursos humanos e a facilidade ou dificuldade de comunicação dos utentes com os profissional_de_saúde e o hospital ou centro hospitalar e utiliza os resultados obtidos. Participação do cidadão: estão instituídas formas de auscultação dos utentes, para além do acesso ao livro amarelo, através de inquéritos de satisfação regulares aos doentes e do fomento de grupos de reflexão constituídos por membros da comunidade envolvente, que devem ser ouvidos no auxílio à tomada de decisão por parte dos Conselhos de Administração.

**** *id_84 *doc_5 *ano_2013 *autor_2 *área_3

O cidadão é o centro do sistema_de_saúde. As estratégias para reforço da cidadania em saúde assentam: No reforço do poder e da responsabilidade do cidadão em contribuir para a melhoria da saúde individual e coletiva. Na promoção de uma dinâmica contínua de desenvolvimento que integre a produção e partilha de informação e conhecimento, literacia em saúde. Numa cultura de pró_atividade, compromisso e autocontrolo do cidadão, capacitação, participação ativa, para a máxima responsabilidade e autonomia individual e coletiva, empowerment. A nível político, se deve: Promover uma cultura de cidadania, assente no desenvolvimento de iniciativas dirigidas à comunidade ou a grupo populacionais, visando a promoção da literacia, capacitação, empowerment e participação, tendo como eixos a difusão da informação, o desenvolvimento de competências, na decisão individual, institucional e política, criando condições para que os cidadãos se tornem mais autónomos e responsáveis em relação à sua saúde e à saúde de quem deles depende, bem como uma visão positiva em saúde. Promulgar e divulgar a carta de direitos e deveres do cidadão no domínio da saúde. Desenvolver o planeamento, intervenções, monitorização e avaliação na área da Cidadania em Saúde: sistemas de informação e monitorização, elaboração de evidência e recomendações, avaliação e identificação de boas práticas, promoção de uma agenda de investigação e inovação. Promover a participação ativa das organizações representativas dos interesses do cidadão. Assegurar que os profissional_de_saúde desenvolvem competências promotoras da cidadania, a nível pré_graduado e pós_graduado, e monitorizar e avaliar as práticas adotadas. Promover, a nível institucional, processos de melhoria contínua do exercício da cidadania, por exemplo avaliações regulares das necessidades do cidadão, intervenções promotoras de participação e literacia. A nível organizacional, as instituições devem: Melhorar os conhecimentos dos cidadãos sobre direitos e deveres e promover as condições para o respetivo exercício; Melhorar a confiança dos cidadãos nas instituições e as condições para a sua valorização, através da auscultação regular das necessidades, expectativas, satisfação e vivências dos utilizadores; Divulgar informação institucional, de forma transparente, publicando os indicadores de evolução do desempenho e dos resultados dos serviços e dos profissional_de_saúde; Promover, monitorizar e avaliar o exercício da cidadania no processo de tomada de decisão, desenvolvimento estratégico e na avaliação institucional, implementando mecanismos de auscultação da satisfação; Desenvolver programas de educação para a saúde e autogestão da doença; Promover o voluntariado para uma cidadania mais ativa. Na sua prática, os profissional_de_saúde devem: Incrementar a prestação de cuidados individualizados e personalizados, com a participação do doente no processo de decisão terapêutica; Considerar e avaliar o contexto socioeconómico e cultural e adequar os cuidados de saúde à realidade do cidadão, família e comunidade. A nível individual, os cidadãos devem: melhorar pró_ativa os conhecimentos e a capacidade de exercer as responsabilidades e os direitos, assim como cumprir os deveres em saúde; Assumir a responsabilidade pela promoção da saúde e por estilos de vida saudáveis e participar ativamente nas decisões referentes à saúde pessoal, da família e comunidade; Estabelecer alianças terapêuticas com os profissional_de_saúde, formando parcerias na gestão da doença; Promover a utilização racional e adequada dos serviços de saúde. Estratégias para reforço da equidade e do acesso adequado aos cuidados de saúde: Na sua prática, os profissional_de_saúde devem: Desenvolver e protocolar a articulação de cuidados e investir de forma pró_ativa na comunicação entre prestadores dentro e entre instituições e serviços. Intervir sobre os determinantes associados ao acesso como fator_chave das iniquidades em saúde, promovendo estratégias de melhoria do acesso, adequando os serviços, flexibilizando a resposta, diversificando as práticas, trocando experiências e avaliando o desempenho. Promover a confiança do cidadão no seu médico e enfermeiro de família numa relação que promova a proximidade e continuidade de cuidados personalizados, como principais gestores da sua situação de saúde, e responsáveis pela mobilidade entre os vários serviços de saúde. A nível individual, os cidadãos devem: Utilizar os mecanismos de acesso de forma adequada às suas necessidades de saúde, compreendendo as vantagens de recorrer a orientações rápidas e urgentes e cuidados personalizados e continuados, em detrimento da utilização inadequada da urgência hospitalar.

**** *id_85 *doc_5 *ano_2022 *autor_2 *área_3

Num mundo complexo, a ética e a ciência nos ajudam a compreender e a trabalhar para o desenvolvimento sustentável do País e do mundo, privilegiando valores como a paz, a prosperidade, o entendimento global, a equidade, a humanização e centralidade nas pessoas, a participação para a cidadania e a transparência dos nossos propósitos e ações. De modo a tornar mais explícitos os alvos que Portugal terá de perseguir, individual e coletivamente, na etapa de implementação do plano_nacional_de_saúde 2030, foram, também, selecionados objetivos estratégicos. Reduzir as desigualdades: Promover a equidade em saúde; Promover a paz, a justiça e a prosperidade; Dinamizar as parcerias entre todos os setores da sociedade. Promover o desenvolvimento de comportamentos, culturas e comunidades saudáveis: Promover a literacia em saúde; Dinamizar ambientes promotores de saúde; Promover a longevidade e o envelhecimento ativo e saudável. Reduzir de um modo integrado a carga das doenças transmissíveis e das não transmissíveis: Reforçar cuidados de saúde sustentáveis; Fortalecer o acesso a cuidados de saúde de qualidade; Dinamizar a integração de cuidados centrados na pessoa. Mais do que um documento, o plano_nacional_de_saúde 2030 é um processo participativo, cocriativo, estruturado e integrador que, partindo da identificação conjunta das necessidades de saúde da população presente em Portugal, decorrentes dos problemas de saúde e dos respetivos determinantes, seleciona as estratégias de saúde adequadas à mudança, visando particularmente a redução das iniquidades em saúde, para uma saúde sustentável de todos para todos. O plano_nacional_de_saúde 2030 segue um modelo de planeamento em saúde sustentável, de base populacional, de natureza trans e multissetorial, tendo por elemento_chave as pessoas, individuais ou coletivas, a participação e o compromisso. Participação: Garantir o envolvimento e compromisso de todos, para a criação de valor e de resultados em saúde; Equidade: Intervir sobre as desigualdades em saúde evitáveis, injustas ou remediáveis, em contextos socioeconómicos, geográficos e demográficos ou por outras dimensões de desigualdades diversas; sustentabilidade: criar e preservar comunidades saudáveis, económica e socialmente justas e ambientalmente adequadas; transparência: promover o acesso a informação de qualidade para a valorização da saúde e para o exercício da cidadania; centralidade nas pessoas: valorizar a diversidade, as necessidades e as expectativas das pessoas. O objeto de um sistema_de_saúde e das políticas de saúde será sempre o cidadão e também a comunidade onde este se insere. Quando se põe a ênfase na centralidade do cidadão no sistema_de_saúde, isso significa que para além de este ser o objeto da atenção dos serviços de saúde, ele deve também passar a ser o sujeito, o ator principal, no sistema_de_saúde. Esta intenção de democratizar a saúde não é nova. No entanto, para transformar essa intenção em realidade, é necessário desenvolver instrumentos apropriados para esse fim. Centralidade do cidadão também significa o seu envolvimento e participação naquilo que diz respeito à proteção e promoção da sua saúde e à dos seus concidadãos. Aqui também, para além da bondade do princípio, é necessário instrumentar a sua aplicação. De natureza multissetorial e transdisciplinar, é transversal a todas as estratégias de intervenção e necessidades de saúde identificadas e permite intervir sobre todos os determinantes de saúde. Implica reforçar ou implementar as estratégias de promoção da saúde avaliadas como mais custo_efetivo. Implica também instituir a análise sistemática das políticas e estratégias de intervenção dos diferentes setores, quanto ao seu impacto na saúde. Neste sentido, se mostra essencial que o sns colabore com as outras instituições da saúde, mas também com os setores da educação, da economia, da segurança social, do ambiente e com os poderes locais, públicos e privados, para proteger e promover a saúde e prevenir a doença, prestar atenção ao indivíduo, mas também à comunidade onde este se insere. O desenvolvimento de projetos e de uma cultura de transição digital, centrados no cidadão e inseridos no Plano de Recuperação e Resiliência, são uma aposta estratégica de que é exemplo a plataforma tecnológica para promover a atividade física integrada no suava, entre outros. A adoção e implementação do digital contribuirá para melhorar, de forma efetiva a literacia e o acesso a cuidados de saúde de qualidade, mitigando desigualdades, reduzindo distâncias geográficas e reforçando a confiança do cidadão no sistema_de_saúde e, em particular, no sns.

**Textual Corpus**

English version

**** *id_9 *doc_2 *year_2018 *author_1 *area_3

The nhs aims to ensure the protection of health and guarantee access to care for all citizens. Therefore, the analysis of any change or measure to be implemented in the health sector should naturally be client centered, focusing on potential effects on health and on the population’s access to healthcare. It must ensure that the measures to be adopted are not only aligned with the primary objectives of the nhs but also have the potential to contribute to their better achievement. The analysis of the potential effects of specialization and advanced nursing education should be guided in this direction, seeking to determine whether specialized nursing practice may or may not contribute to achieving health gains and improving access to healthcare.

**** *id_11 *doc_4 *year_2013 *author_2 *area_1

Integrated_care_process place the citizen, with their needs and expectations, at the center of the system. Continuity of care and coordination between different levels of care are recognized as essential elements to ensure that the patient receives the best healthcare, timely and effective. The approach of integrated_care_process is multidisciplinary approach, comprehensive, and integrated, requiring the reanalysis of all actions directed at the patient at any point in the nhs, from the beginning to the end of the integrated_care_process. Moreover, care activities based on the best available scientific evidence respect the principle of rational use of health technologies and guide the adoption of cost_effective therapeutic actions, while ensuring the citizen the clinical quality enshrined as one of their main rights. This approach aims to promote organizational change, based on the involvement of all healthcare_professional engaged in care delivery, believing in their ability and willingness to continuously improve quality and to focus their efforts on people. Integrated_care_process are also a tool that allows analyzing the different components involved in healthcare delivery and organizing the various workflows, integrating current knowledge, standardizing actions, and emphasizing results, in order to meet the expectations of both citizens and healthcare_professional. It is therefore necessary to orient clinical practice toward results and quality, aiming at the most effective use of resources. Management through integrated_care_process calls for the reorganization of patient care by health services, focusing it on the citizen, in this particular case, on the needs and expectations of the person with type 2 diabetes, while always considering 2 key questions that must be answered: For whom are we doing this, and how should we do it correctly. This approach seeks a flexible interpretation of what is defined on paper, taking into account available resources, to achieve better adaptation and improved care delivery for people with type 2 diabetes. This document was thus conceived as a working material, adaptable to the specific local context of each healthcare unit. Type 2 diabetes is consistent with the strategic objectives and main indicators defined in the national diabetes program but has a specifically circumscribed scope: it focuses on the care pathway of the person diagnosed with diabetes, as is characteristic of the general design of integrated_care_process. The needs and expectations of the person with diabetes, and their families and caregivers, are the core element and the starting point for the development of this integrated_care_process. These needs and expectations were identified through several sources of information, namely satisfaction surveys, suggestions, complaints, and all the experience gathered from the development of national programs, particularly the national diabetes program and focus groups. They were taken into account in the development of the various components of this integrated_care_process. Likewise, healthcare_professional who care for people with diabetes have their own expectations and needs, whose fulfillment influences the quality of care provided. At each level of care or field of action, the healthcare_professional involved should seek and identify specific expectations of the person with diabetes and their families related to the moment of care delivery and the concrete environment in which it occurs. The family doctor and nurse, supported by the rest of the multidisciplinary team working in a coordinated way, promote, collaborate, and carry out the annual screening for chronic complications listed below. The multidisciplinary team works in a coordinated and articulated manner, promotes collaboration, and ensures continuity of care within the scope of the specific competences of each healthcare_professional. The multidisciplinary team ensures among its members the appropriate exchange of all clinical and non_clinical information related to the provision of quality healthcare to the person with diabetes. The multidisciplinary team evaluates all factors that influence health habits, in order to intervene on those determinants of healthy lifestyles, to carry out therapeutic education, and to adapt the therapeutic and clinical monitoring plan to the individual characteristics of the person with diabetes: knowledge, experiences, and beliefs about diabetes and its treatment, including the scheduling of all tasks inherent to proper monitoring; level of education and understanding; mood, anxiety, and sense of well_being; physical condition: visual acuity, psychomotor abilities; lifestyle: social life, leisure time, tobacco, alcohol; healthy eating: intolerance, taste and/or preferences, type, number and timing of daily meals; work: schedule, physical activity, travel; family: relationships and family support; economic situation: financial balance; self_care and therapy: barriers and/or difficulties. The multidisciplinary team ensures therapeutic education, providing the necessary and appropriate information and education to increase knowledge about diabetes and to train the skills required for active collaboration in metabolic control. This phase should take place as early as possible and must be personalized according to the individual characteristics of each person with diabetes, during follow_up consultations, being adjusted and adapted over time. Psychological and social support should be provided, with special emphasis on social and family integration. The multidisciplinary team participates in therapeutic education and in the promotion of healthy lifestyles, diet, and physical exercise. The nutritionist and dietitian ensure education for the practice of balanced, varied, and complete nutrition for people at risk and those with diabetes. The multidisciplinary team ensures therapeutic education regarding the recognition and prevention of hypoglycemia, as well as of the chronic complications of diabetes. It also provides therapeutic education in self_administration and self_monitoring techniques. The multidisciplinary team ensures the communication of all information and training necessary to enable the person with diabetes to record, describe, interpret, and register all data in the guide. The multidisciplinary team promotes the creation of associations of people with diabetes, at a level of proximity to their social environment, with community involvement. The doctor and nurse work as a team, coordinating whenever necessary with other technicians and healthcare_professional, and promoting an adequate and timely provision of healthcare to the person with diabetes referred from primary healthcare, within the scope of a specific diabetes consultation. In particular, regarding complications such as diabetic retinopathy, diabetic nephropathy, diabetic foot, and gestational diabetes, hospital healthcare_professional ensure continuity of healthcare according to the standards and guidelines issued by the directorate general of health, in order to respond to the needs of people with diabetes. Within hospital care, responsibilities include ensuring, whether in consultation or during hospitalization, regardless of the clinical reason, among others: personalized support, appropriate emotional support and empathy; assessment of metabolic control, glycemic profile, HbA1c, lipids, as well as evaluation of body mass index and blood pressure; evaluation, screening, and treatment of intercurrent conditions. The doctor and nurse, coordinating whenever necessary with other healthcare_professional, work in a coordinated manner to ensure a comprehensive assessment, including the evaluation of cardiovascular risk, retinopathy, nephropathy, and diabetic foot. The discharge report sent to the hospital or long_term care team must include the activities carried out during hospitalization, the diagnoses and clinical decisions, as well as the therapeutic and care plan. The nursing discharge note must contain information on the educational activities undertaken and the monitoring plan and care recommended for the next level of care. The doctor, nurse, social worker, and therapist work as a team and promote timely referral in order to ensure the continuity of healthcare delivery to the person with diabetes coming from another level of care. The multidisciplinary team establishes and defines an personalized_care_plan for each person with diabetes. The team identifies, through clinical and social assessments, the presence of limitations to self_care. The team provides the person with diabetes with the appropriate tools to facilitate self_care: in sensory limitations, in mobility limitations, and in cognitive limitations.

**** *id_12 *doc_4 *year_2014 *author_2 *area_1

The integrated_care_process place the citizen, with their needs and expectations, at the center of the system. Continuity of care and coordination between different levels of care are recognized as essential elements to ensure that the patient receives the best healthcare, timely and effective. The approach of the integrated_care_process is a multidisciplinary, comprehensive, and integrated approach that requires the reanalysis of all actions directed at the patient at any point within the nhs, from the beginning to the end of the integrated_care_process. Furthermore, care activities based on the best available scientific evidence respect the principle of the rational use of health technologies and guide the adoption of cost_effective therapeutic actions, while guaranteeing citizens the clinical quality enshrined as one of their fundamental rights. This approach aims to promote organizational change, based on the involvement of all healthcare_profissional involved in care delivery, trusting in their ability and willingness to continuously improve quality and to focus their efforts on people. The integrated_care_process are also a tool that allows the analysis of the different components involved in the provision of healthcare and the organization of various workflows, integrating current knowledge, standardizing practices, and emphasizing results, in order to respond to the expectations of both citizens and healthcare_profissional. It is necessary to orient clinical practice toward results and quality, aiming for the most effective use of resources. The management of clinical practice, as defined in the integrated_care_process, calls for the reorganization of the care provided to people by the healthcare_system, centering it on the citizen and, in this particular case, on the needs and expectations of the person with vascular risk, and on the anticipation and comprehensive planning of continuity of care. There are 2 parameters to consider: for whom we do things and how to do them correctly. The healthcare_profissional provides the information necessary to promote the participation and decision_making of the person with vascular risk, including the family member or caregiver, enabling them to exercise their rights. Interventions aimed at lifestyle modification are as important as pharmacological measures and, in many cases, more effective, improving the control of vascular risk factors and vascular morbidity and mortality, and therefore must be implemented in a personalized manner for the person with cardiovascular risk. This set of activities identifies, in the adult person, the presence or absence of vascular risk factors and or known vascular disease, evaluates the overall cardiovascular risk, and, based on this, plans and schedules, together with the person and or caregiver, preventive activities, therapeutic follow_up, monitoring, and healthcare delivery. All clinical action implies continuity of care and coordination among different healthcare_profissional and between the different levels of healthcare. The needs and expectations of the person with cardiovascular risk, and of their family members and caregivers, are the core element and starting point for the development of this integrated_care_process. These needs and expectations were identified through various sources of information, namely, satisfaction surveys, suggestions, complaints, the development of national programs, and focus groups. They are reflected in the different components of this integrated_care_process. Likewise, the healthcare_profissional who provide care to the person with vascular risk have their own expectations and needs, whose fulfillment determines the quality of the care provided. At each level of care or field of action, the healthcare_profissional involved should seek and identify specific expectations of the person with vascular risk and their families and caregivers, related to the timing of care delivery and the concrete environment in which it takes place. The active co_responsibility of people in the treatment and management of their disease is essential to the success of clinical activity based on existing scientific recommendations and consensus. The intervention of all healthcare_profissional and of the community in the implementation of prevention measures for cerebro_cardiovascular diseases is fundamental. For all these reasons, the effectiveness and efficiency of interventions directed at the person with vascular risk require the action of multidisciplinary teams of healthcare_profissional, demanding adequate communication and cooperation to avoid unconnected, episodic activities, duplication of acts, and waste of resources, which result in disorganized, poorly controlled care and the inability to find the best solution for people’s problems, leading to their dissatisfaction. The healthcare_profissional provides the necessary information to promote the participation and decision_making of the person with vascular risk, including the family member or caregiver, enabling them to exercise their rights. The healthcare_profissional ensures the most effective communication with each person with vascular risk, applying the communication techniques appropriate to their professional scope. The healthcare_profissional ensures quality clinical care, updated and based on the best scientific evidence, within the professional scope of their specialty. The healthcare_profissional ensures the continuity of healthcare to the person with vascular risk, in accordance with this integrated_care_process.

**** *id_14 *doc_4 *year_2016 *author_2 *area_1

The integrated_care_process place the citizen, with their needs and expectations, at the center of the nhs. Continuity of care and coordination between different levels of care are recognized as essential elements to ensure that the patient receives the best healthcare, timely and effective. The integrated_care_process approach is multidisciplinary, comprehensive, and integrated, requiring the reanalysis of all actions directed at the patient at any point within the nhs, from the beginning to the end of the integrated_care_process. Furthermore, care activities based on the best available scientific evidence respect the principle of the rational use of health technologies and guide the adoption of cost_effective therapeutic actions, while ensuring citizens the clinical quality enshrined as one of their main rights. This approach aims to promote organizational change, based on the involvement of all healthcare_profissional involved in care delivery, trusting in their ability and willingness to continuously improve quality and to focus their efforts on people. The integrated_care_process are also a tool that allows the analysis of the different components involved in the provision of healthcare and the organization of various workflows, integrating current knowledge, standardizing practices, and emphasizing results, in order to meet the expectations of both citizens and healthcare_profissional. It is necessary to orient clinical practice toward results and quality, aiming at the most effective use of resources. The management of clinical practice, as defined in the integrated_care_process, calls for the reorganization of care provided to people by the nhs, centering it on the citizen and, in this particular case, on the needs and expectations of the adult person with pre_obesity, and on the anticipation and comprehensive planning of continuity of care. There are 2 parameters to consider: for whom we do things and how to do them correctly. The integrated_care_process for adult pre_obesity is aligned with the strategic objectives and main indicators defined in the National Program for the Promotion of Healthy Eating, but it has, specifically, a limited scope: it focuses on the pathway of the adult person diagnosed with pre_obesity, as is characteristic of the general design of the integrated_care_process. The needs and expectations of the adult person with pre_obesity, and of their family members and caregivers, are the core element and starting point for the development of this integrated_care_process. These needs and expectations were identified through several sources of information, namely satisfaction surveys, suggestions, complaints, and all the experience gathered from the development of national programs, particularly the national program for the promotion of healthy eating, and focus groups. They were taken into account in the development of the various components of this integrated_care_process. Likewise, concerning the healthcare_profissional who provide care to adults with pre_obesity, their own expectations and needs, whose satisfaction determines the quality of care provided, were also considered. At each level of care or field of practice, the healthcare_profissional involved should seek and identify specific expectations of the adult person with pre_obesity and their families, related to the timing of care delivery and the specific environment in which it occurs. The healthcare_profissional provides the information necessary to promote the participation and decision_making of the adult person with pre_obesity, including the family member or caregiver, enabling them to exercise their rights. The healthcare_profissional ensures effective communication with each adult person with pre_obesity, applying the communication techniques appropriate to their professional scope. The healthcare_profissional ensures quality clinical care in pre_obesity, up_to_date and based on the best scientific evidence, within the professional scope of their specialty. The healthcare_profissional ensures continuity of healthcare to the adult person with pre_obesity in accordance with this.

**** *id_16 *doc_5 *year_2015 *author_2 *area_3

The report developed by the who on the implementation of the health_national_plan concludes that the health_national_plan 2012_2016 is aligned with the who health_2020 strategy, although implementation instruments should be strengthened. The report A Future for Health was based on the challenge of creating a vision for health and healthcare in Portugal for the next 25 years, describing what this could mean in practice, particularly in terms of implementation and sustainability. This document proposes a simple vision characterized by citizen empowerment, active participation of society, and a continuous pursuit of quality, calling for a new pact for health. The model of health co_production states that, on one hand, governance for health is shared by the different sectors of society, including public administration, and, on the other, health governance and healthcare delivery simultaneously contribute to the population’s state of health and health gains. This model serves as an inspiration for the formulation of the guidelines of this health_national_plan. The who health_2020 strategy is the reference framework for European health policies. Its strategic priorities are: investing in health throughout the life cycle by empowering citizens; combating communicable and non_communicable diseases; strengthening people centered health_system and public_health response capacity, particularly surveillance, preparedness, and response to threats; and developing resilient communities and protective environments. The health_national_plan is guided by the values and principles of transparency and accountability, which enable trust and appreciation of all stakeholders, as well as a system that evolves through learning. In addition, it emphasizes the involvement and participation of all actors in the processes of health creation; the reduction of health inequalities as the foundation for the promotion of equity and social justice; the integration and continuity of care provided to citizens; a health_system that responds quickly to needs, making the best use of available resources to avoid waste; and sustainability, to preserve these values for the future. This sustainability should combine: a healthy population; resilient communities with a strong informal care network; health policies and practices well integrated into other social and economic policies and practices; and a well designed, people centered healthcare system, adequate to its objectives, efficient, with qualified human resources working in teams to provide integrated healthcare. The citizen, understood as central to the health_system, is an important agent of participation and change. Therefore, they have both the right and the duty to influence health policy decisions that collectively affect the population, in their different roles: as a patient with specific needs; as a consumer with expectations and the right to safe and quality care; and as a contributor to the nhs. Likewise, the citizen must be empowered to take responsibility for safeguarding their own health and the collective health. To do so, the citizen must be informed, must internalize such information, and translate it into changes in less healthy behaviors and, when applicable, in the management of their disease. Only then will the citizen, individually or collectively, be empowered to be heard and to participate in decisions that concern them, contributing to consensus on health priorities and to a stable and broad political commitment that allows achieving the objectives of this health_national_plan. The health_national_plan proposes: promoting a culture of citizenship aimed at improving literacy and empowering citizens to become more autonomous and responsible regarding their own health and the health of those who depend on them; promoting the active participation of organizations representing citizens’ interests; developing skills among healthcare_profissional that allow them to promote health citizenship actions; developing programs for health education and disease self_management; and developing programs for the rational and appropriate use of health services.

**** *id_17 *doc_5 *year_2022 *author_2 *area_3

According to the who, leadership commitment involves several requirements, such as transparency on the part of both patients and healthcare_profissional, in the sharing of information as well as in the reduction of a hierarchical approach, promoting a transversal perspective, open and respectful communication, the development of a culture of learning from errors and best practices, teamwork, willingness to learn, valuing and supporting healthcare_profissional, together with a judicious balance between a no_blame policy and accountability. Therefore, these requirements become indispensable to a culture of safety and stem from leadership, bearing in mind that the patient must be at the center of care and of the system. This process requires strong leadership at all levels, from the ministry_of_health, health institutions, partners, and all teams involved in care delivery. The introduction and development of telehealth tools is already a national reality in some contexts. However, its expansion to different levels of care still represents a challenge, since the implementation of telehealth models will only make sense if they add value to people’s health, thereby ensuring the principles of humanization, quality, and safety of care. The introduction of innovative mechanisms should contribute to the model of personalized care, person_centered, accompanying demographic changes and biotechnological innovation, people’s expectations, and the empowerment of services to demonstrate outcomes based on principles of health quality. In this context, within the scope of health innovation, the development and implementation of successful teleconsultation and telemonitoring projects may take into account the following assumptions: greater humanization in healthcare delivery; rapid and timely integration of the best scientific evidence; clear and unambiguous definition of care pathways to facilitate citizen navigation through the different levels of care; maximum safety in healthcare delivery, which includes the elaboration of procedures, records, risk assessment, and safe monitoring of data, among others. The integration of concepts and guidelines aimed at increasing health quality and safety, such as telehealth models, is conditioned by variables external to the technological development process, which should be interpreted as challenges to the implementation of this tool. These include: the level of citizens’ digital literacy; the articulation between information technology professionals and healthcare_profissional; the creation of qualification programs for healthcare_profissional in this field; the definition of an personalized_care_plan together with the patient; the promotion of the home as a level of care; the definition of criteria that ensure the quality of care provided and supported by telemonitoring; the preservation of personal interaction between healthcare_profissional and patient, caregiver, and family; the establishment of safe processes; and the protection of personal data. The national plan for patient safety constitutes a support tool for senior managers, intermediate leadership, members of quality and safety commissions, patient safety managers, and healthcare_profissional, requiring active involvement, governance responsibility, coordination, and operationalization at different levels of care, in order to increase the safety of healthcare delivery, keeping the focus on the patient and on caregivers.

**** *id_18 *doc_5 *year_2014 *author_2 *area_3

Based on the 2 main domains of demand and supply, it is understood that, in the domain of demand, the citizen constitutes the center of the conceptualization of policies and interventions in addictive_and_dependency_behaviors, based on the premise that it is essential to respond to individuals’ needs, viewed dynamically throughout their life cycle. The goal is to develop global and comprehensive interventions that integrate a continuum ranging from health promotion, prevention, deterrence, risk reduction and harm minimization, to treatment and social reintegration. This domain also includes 2 structuring measures: the operational plan of integrated responses and the referral or articulation network within the scope of addictive_and_dependency_behaviors, through which it seeks to respond effectively and sustainably to current needs in this area. The recognition that the citizen is diverse, in life cycle stages, gender, nationality, economic, social, educational, cultural, and family status, personality styles, knowledge, conceptions of well_being, among other possible dimensions, and has different experiences in the use of these mediators of well_being depending on internal and external variables, the product or activity, and the life stage, is reflected in the prioritization of areas such as updating information, knowledge, and skills; developing conditions for innovation and for adapting interventions to diversity; and promoting cooperation in the domains of knowledge and action, aiming for a personalized, quality, and integrated intervention with other sectors at local and national levels. Centrality of the citizen: In the national plan against drugs and drug addiction 2005_2012, it was considered that intervention in drug addiction is not an end in itself, and should move away from focusing on substances to assume the centrality of the citizen and their objective and subjective needs. For the next strategic cycle, this principle remains, broadening the focus beyond drug addiction to encompass the wider range of addictive_and_dependency_behaviors. Considering the current reorganization of services in this field, it is the state’s responsibility to ensure the streamlining of bureaucracy and processes that aim to promote and provide healthcare, according to the overall satisfaction of individuals’ needs, recognizing that they have an active voice and shared responsibility in defining their life project, with equitable rights and duties of citizenship. Services should therefore act as managers of citizens’ health capital, with citizens deciding whether to access them. Thus, for the current strategic cycle, the centrality of the citizen is emphasized, from a dynamic perspective of their life cycle, developing across its different stages. The individual is co_responsible and manager of their choices and behaviors aimed at health, quality_of_life and well_being, as a citizen and user of services, as well as an active participant promoting the exercise of citizenship in the contexts they inhabit throughout their life. Integrated intervention: The processes leading to addictive_and_dependency_behaviors are diverse and often cumulative. They are of a structuring nature, and their manifestations may be economic, social, political, or even cultural. Integration must therefore be understood as a global, concerted, and collective response, encompassing the various dimensions of the phenomena and allowing the strategic articulation of actions to be developed. Thus, the complexity inherent to addictive_and_dependency_behaviors requires a holistic and developmental vision, in which the individual is viewed as the center of the approach, shifting focus away from the substance used or the manifested behaviors. Addressing this complexity requires multidisciplinary approaches and a range of responses and mechanisms that, in an articulated and coherent manner, act on the various dimensions of this phenomenon. Accordingly, the consolidation of an integrated model of responses presupposes an interdependent continuum of responses, namely prevention, deterrence, risk reduction and harm minimization, treatment, and reintegration. The integrated responses model is therefore based on a multidimensional reading of the reality of addictive_and_dependency_behaviors and on a proximity_based, multisectoral, and cross_sectoral intervention, which allows maximizing results and achieving social and health gains. This conception moves away from the partial view of mere summation and juxtaposition of interventions, making it essential to invest strongly in interinstitutional articulation and in the formulation of cross_cutting strategic objectives across interventions, thus avoiding dispersion, optimizing available resources, and leveraging potential synergies. Within the scope of treatment, intervention should focus on individualized diagnosis and responses based on the availability of a network that ensures adequate and continuous care, according to the pathology presented and any comorbidities. Hence, the relevance of its comprehensive and cross_cutting nature throughout all intervention in the problem of drug addiction is emphasized. Therefore, it is important to adapt intervention strategies to the individual’s situation, which translates into the integration of interventions across the various types of intervention, always seeking to ensure an approach based on the logic of satisfying the individual’s needs and adjusting available responses to the interventions identified as necessary. The focus of intervention should center on the different approaches, namely in the areas of prevention, risk reduction and harm minimization, treatment, and reintegration, favoring proximity and accessibility of responses to the individuals who need them. For this purpose, it is essential to raise awareness and build capacity among healthcare_profissional and other stakeholders and to create conditions for the development of an articulated and intersectoral intervention, necessary for specialized response services, particularly primary healthcare and the social network, to provide adequate follow_up for this issue in the final stage of the life cycle. Considering once again the centrality of the citizen as a fundamental value of intervention, training needs should be identified by the services through rigorous diagnostics, aiming at improving the effectiveness and efficiency of public service.

**** *id_23 *doc_3 *year_2019 *author_2 *area_3

The right to health protection is the right of all people to enjoy the best possible state of physical, mental, and social health, implying the creation and development of economic, social, cultural, and environmental conditions that ensure sufficient and healthy levels of living, work, and leisure. The right to health protection constitutes a joint responsibility of individuals, society, and the state, and includes lifelong access to health promotion, prevention, treatment, and rehabilitation, as well as to long_term care and palliative care. Base 2: All people have the right to: protection of health, with respect for the principles of equality, non_discrimination, confidentiality, and privacy; access to healthcare appropriate to their condition, promptly and within a clinically acceptable timeframe, in a dignified manner, according to the best available scientific evidence and following good practices of quality and safety in health; freely choose the healthcare provider, within the limits of available resources; receive information about the response time for the healthcare they require; be informed adequately, accessibly, objectively, completely, and intelligibly about their condition, the objective, nature, possible alternatives, benefits and risks of the proposed interventions, and the probable evolution of their health status according to the care plan to be adopted; decide freely and in an informed way, at all times, about the healthcare proposed to them, except in exceptional cases provided by law; issue advance directives of will and appoint a healthcare proxy; freely access information concerning themselves without the need for mediation by a healthcare_profissional, except when requested; be accompanied by a family member or another person of their choice and receive religious or spiritual assistance; submit suggestions and complaints and obtain a response from the responsible entities; participate in decision_making processes in health and in the participatory management of nhs institutions; form entities that represent and defend their rights and interests, namely in the form of associations for health promotion and disease prevention, leagues of friends, and other forms of participation provided by law; promotion of well_being and quality_of_life during aging, in an inclusive and active perspective that promotes decision_making capacity and control over one’s life, through the creation of adaptive mechanisms of acceptance, autonomy, and independence, with socioeconomic, environmental, social, and healthcare factors being determinant. The foundations of health policy are: health promotion and disease prevention, which must be considered in the definition and implementation of other public policies; improvement of the population’s health status through a public_health approach, epidemiological monitoring and surveillance, and implementation of national, regional, and local health plans; people as the central element in the design, organization, and operation of health establishments, services, and responses; equality and non_discrimination in access to quality healthcare in a timely manner; ensuring equity in the distribution of resources and in the use of services; adoption of positive differentiation measures for people and groups in situations of greater vulnerability; promotion of health education and health literacy, enabling free and informed choices for adopting healthy lifestyles; participation of individuals, communities, healthcare_profissional, and municipal bodies in the definition, monitoring, and evaluation of health policies; management of available resources according to criteria of effectiveness, efficiency, and quality; all healthcare_profissional working in the nhs have the right to a professional career that recognizes their differentiation within the health field. Base 13: The state promotes the improvement of the mental health of individuals and society in general, particularly through the promotion of mental well_being, prevention, and timely identification of mental illnesses and the risks associated with them. Mental healthcare must be person_centered, recognizing individuality, specific needs, and level of autonomy, and must be provided through an interdisciplinary and integrated approach, primarily at the community level. People affected by mental illness must not be stigmatized, discriminated against, or disrespected in the context of healthcare because of their condition. Base 17: The use of health technologies must reinforce humanization and the dignity of the person. Base 20: The nhs conducts its activity according to the following principles: Quality, aiming at effective, safe, and efficient healthcare, based on evidence, provided in a humanized manner, with technical accuracy and attention to the individuality of the person.

**** *id_24 *doc_3 *year_2019 *author_2 *area_3

The charter for public participation in health, hereinafter referred to as the charter, aims to promote participation by individuals, with or without illness, and their representatives, in decisions that affect the health of the population, and to encourage health decision_making based on broad public participation. The charter also seeks to promote and consolidate public participation at the political level and within the various bodies and entities of the state in Portugal, through the strengthening of existing participation processes and the creation of new participatory spaces and mechanisms. In this way, the charter contributes to: promoting and defending the rights of people with or without illness, particularly regarding the protection of health, information, and participation; informing public entities about the priorities, needs, and concerns of people with or without illness and their representatives; making health policies more effective and, consequently, achieving better health outcomes. Public participation in health must be based on the following principles: recognition of public participation as a right of people with or without illness and their representatives; recognition of people with or without illness and their representatives as partners in decision_making processes; recognition of the importance of the specific knowledge and experience of people with or without illness; autonomy and independence of people with or without illness and their representatives in these processes; transparency and public disclosure of participatory processes; creation of the necessary conditions for participation; complementarity and integration between institutions and mechanisms of representative democracy and participatory democracy. Public participation must also be implemented systematically through various mechanisms, in order to address the specificities of all interested and affected parties and to promote broad and diverse participation, namely through: public meetings; public hearings; public consultations; representation in advisory councils, committees, or specialized or sectoral working groups within the scope of health policy and related policies, both at the national, regional, and municipal levels; community councils within the various relevant entities and services in the field of health and related policies; user committees; municipal health councils; the national council for participation in health; the national forum on participation in health; and digital platforms for public participation in health.

**** *id_25 *doc_3 *year_2020 *author_2 *area_3

More than 10 years after the implementation of the integrated management model for chronic kidney disease, it is necessary to revalidate the model and advance with a renewed strategic agenda focused on the better management of the patient and their care pathway, from the prevention of chronic kidney disease to its diagnosis and therapeutic approach, in order to improve the quality and safety of healthcare delivery to the chronic kidney patient, within a truly integrated, effective, and person_centered model. Thus, considering that the current integrated governance model, through its multidisciplinary and intersectoral intervention, aims to respond to the new demographic and epidemiological challenges of providing healthcare to people with chronic kidney disease, I hereby determine the following: the national dialysis monitoring committee has the mission to: promote person_centered innovation, transforming processes for the effective integration of dialysis modalities, individualization, home_based treatments, empowerment of the patient, and capacity building of the clinical team; promote continuous improvement in the management of access to dialysis, allocation to dialysis, and transition between treatment methods, ensuring better clinical outcomes and greater value perceived by the patient; promote the continuous improvement of information systems supporting clinical governance and of technological means of health communication; assess health gains resulting from this clinical governance model, from a perspective of continuous quality improvement; monitor and evaluate healthcare delivery to people with chronic kidney disease, particularly regarding access and provision of specific healthcare, the quality of care and patient safety, the satisfaction level of patients in chronic dialysis, financing models for care, and healthcare performance outcomes; issue technical and scientific opinions whenever requested; and provide technical support for the implementation of the integrated management model for chronic kidney disease, which includes the comprehensive price payment model.

**** *id_26 *doc_5 *year_2020 *author_2 *area_3

To consolidate a strategic and holistic approach to prevention and intervention focused on people in situations of homelessness, so that no one has to remain on the streets due to the absence of alternatives. Principles: promotion of an approach based on human rights and the realization of human dignity; fulfillment of citizenship rights and duties; promotion of non_discrimination and equality, namely equality between women and men; promotion of reflective and current knowledge about the dimension and nature of the phenomenon to support the development of intervention strategies; promotion of the recognition and deepening of the multidimensionality and complexity of the phenomenon and the consequent need for adequacy and persistence in implementing measures; definition and implementation of prevention, intervention, and follow_up measures; co_responsibility and mobilization of all public and private entities, in a logic of subsidiarity, for an integrated and consistent intervention, ensuring accessibility to existing services, responses, and care; recognition and adaptation to local specificities and the diversity of groups that make up the population in situations of homelessness; recognition and adaptation to the specificities of women and men; guarantee of quality intervention centered on the person, safeguarding their privacy throughout the entire process of support and follow_up; proactive participation and promotion of the empowerment of the person in a situation of homelessness at all levels of the process of social inclusion; education and community engagement; monitoring of the process and evaluation of the results of the implementation of enipssa 2017_2023. the intervention model to be used in the implementation of enipssa 2017_2023 is based on the centrality of the person as a whole and within their life context, which is intended to be integrated and comprehensive, aiming at the prevention of new situations and close follow_up, and is based on the premise of qualification and optimization of human and financial resources, namely to avoid duplication of responses. This model requires a multidimensional approach in the elaboration of situation diagnoses and case follow_up, with the design of an individual life project aimed at social integration and autonomy from support services whenever possible, built through the relationship between the user and the case manager, with whom they maintain a privileged relationship. Axis 2: Strengthening an intervention promoting the integration of people in situations of homelessness. The measures included in this axis aim to strengthen intervention promoting the integration of people in situations of homelessness, ensuring quality, effectiveness, and efficiency in 2 key dimensions: Technical intervention, through the training of technicians and managers of social response structures and public service units, based on the adoption of integrated intervention methodologies developed from a specific model. The implementation of a specific training framework for intervention in the multidimensionality of this issue requires that it be designed and directed to different levels of stakeholders, not only to managers and healthcare_professional who directly work with people in situations of homelessness, but also to those who can ensure accessibility to services. The integrated intervention and follow_up methodology presupposes coordination among different local services and the promotion and guarantee of effective and efficient intervention, optimizing the existing community resources through the integrated application of measures and programs from different areas of action, always centered on the person in a situation of homelessness.

**** *id_28 *doc_3 *year_2022 *author_2 *area_3

The recent covid_19 pandemic, to which the public_health services responded to the challenge of identifying cases, isolating contacts, testing and vaccinating people, and treating patients without exception, reinforced the importance of investing in a strong healthcare system, one that places people at its center and has as an essential pillar a public_health service that is accessible to all and tends to be free of charge. Currently, the nhs faces significant challenges associated with the evolution of health needs and the growing demands and expectations of the population. In a context of pandemic recovery, the proposals for the nhs are framed within an approach centered on people’s needs, in order to protect and improve their quality_of_life from birth until the end of life. Also with this objective, and considering the relevance that human resources have within the nhs, as the main guarantee of its quality, a strategic management program for the nhs human resources was approved, based on a multidimensional vision with particular focus on valuing human resources and the planned recruitment of the healthcare_professional necessary to meet organizational demands. Aiming for a fairer and more inclusive nhs that better responds to the population’s needs, the Government will: improve access to medical appointments and health promotion and disease prevention activities through multidisciplinary interventions suited to each citizen’s characteristics, namely in accordance with the strategies and guidelines of the national_health_plan 21_30; improve the organization and coordination of public_health services by creating mechanisms for greater integration between nhs structures, civil protection, the social sector, and civil society actors with direct and indirect involvement in health; foster the use of telehealth as a proximity_based response to citizens’ needs and create a national telemedicine center and a national telemedicine network; optimize access to medicines, bringing them closer to users, and ensure efficiency throughout the entire medication chain by strengthening the role of pharmacy and therapeutics committees, supporting prescribers, and involving users and their representatives in the different phases of the process; promote integrated and continuous care centered on the user through information systems, particularly by creating a single electronic_health_record that integrates the different levels and sectors of care provision, enables access to relevant clinical information anywhere within the nhs network, and promotes citizens’ autonomy in managing their health processes; promote integrated management projects for users’ care pathways within the nhs, reinforcing continuity of care and mechanisms of service integration for the follow_up of patients with chronic diseases; strengthen autonomy in hospital management, particularly regarding the recruitment of healthcare_professional, with greater accountability and assessment of satisfaction by both users and healthcare_professional.

**** *id_31 *doc_3 *year_2023 *author_2 *area_1

Long_term care comprises a range of combined and integrated care services and activities that adopt a holistic view of the processes of aging and dependency protection, through which both the needs and the care provided by different actors are aligned according to the functional capacity of the person, who is the focus of care, and the capacity of the caregivers, both formal and informal. The care referred to in the previous paragraph constitutes a community_based response and must include: reintegration to preserve or recreate the relational network of belonging; rehabilitation and/or readaptation to restore autonomy and independence; maintenance and delay of the loss of autonomy and independence; promotion of well_being and quality_of_life.
Specialized integrated care services may be attached to existing or future residential facilities for older people and to long_term and maintenance units integrated into the network of continued care, thereby increasing their social and health relevance. The general objective of long_term care in the Autonomous Region of Madeira is to ensure the best possible care for people, with effectiveness and efficiency in terms of adequacy and integration, adaptable and targeted at any time to the functional needs arising from the processes of aging or progressive loss of autonomy. Long_term care is developed according to the following guiding principles: Long_term care in the Autonomous Region of Madeira respects the dignity and other fundamental rights and freedoms of people requiring such care, as well as those of their families and caregivers; Long_term care in the Autonomous Region of Madeira aims to restore, as far as possible, or prevent the deterioration of the physical and/or mental health status of people in need of long_term care and to strengthen their capacity to live independently, while alleviating their experience of loneliness or social isolation; Person_centeredness: Long_term care services in the Autonomous Region of Madeira are provided without any form of discrimination and address each individual’s specific needs, respecting personal integrity and taking into account gender, physical condition, and intellectual, cultural, ethnic, religious, linguistic, and social diversity, and, when appropriate, that of their families or immediate social circle; Comprehensiveness and continuity: Long_term care in the Autonomous Region of Madeira is designed and delivered in an integrated manner with all other services, including healthcare and telehealth, ensuring effective coordination between regional and local levels. It is organized so that people requiring such care can rely on an uninterrupted range of services whenever necessary and for as long as needed, with transitions between different care services being smooth and avoiding service disruption or any negative impact on care received; Outcome focus: Long_term care in the Autonomous Region of Madeira is centered primarily on the benefits for care recipients in terms of their quality_of_life and ability to live independently, while also considering, when appropriate, the benefits for their families, informal caregivers, and community; Transparency: Information on the available long_term care options and providers in the Autonomous Region of Madeira, including quality, standards, and quality assurance agreements, must be fully provided in an accessible and comprehensible manner to people in need of specialized and prolonged care, their families, or caregivers. Qualified provision: Long_term care in the Autonomous Region of Madeira is delivered by qualified workers, with appropriate staffing ratios reflecting the number and needs of care recipients in different settings. The rights of workers, confidentiality, professional ethics, and professional autonomy must be respected, and continuous training made available to all healthcare_professional involved.

**** *id_32 *doc_3 *year_2023 *author_2 *area_3

The increase in the population’s health and well_being needs, associated with aging, the burden of disease, as well as their growing demands and expectations, requires the nhs to continue to increase access and efficiency in the provision of healthcare, fostering organizational models that promote the integrated management of primary healthcare and hospital care, ensuring a focus on people. This change aims at the integrated provision of primary and hospital healthcare, the strengthening of primary care in providing proximity and continuity in health assistance, and an emphasis on health promotion. It should be noted that the maturation of this organizational model allows local health units to benefit from new management instruments, namely: risk stratification that identifies the distribution of the burden of disease in the population; information systems that enhance care integration, such as the single electronic_health_record; performance incentives, financial and nonfinancial, focused on outcomes and value creation; and innovative models of care delivery, based on teams that assume commitments centered on responding to people, with emphasis on family health units and community care units in the scope of primary care, or, in the hospital area, integrated responsibility centers. The integration of existing primary care groups, hospitals, and hospital centers into the local health unit model constitutes a qualification of the nhs response, simplifying processes, increasing articulation between teams of healthcare_professional, focusing on the experience and on the pathways between different levels of care, increasing managerial autonomy, improving the participation of citizens, communities, healthcare_professional, and local_authorities in defining, monitoring, and evaluating health policies, maximizing access and efficiency of the nhs.

**** *id_33 *doc_5 *year_2023 *author_2 *area_1

The 23rd Constitutional Government assumed in its program the commitment to a fairer and more inclusive nhs that better responds to the needs of the population, reinforcing the importance of maintaining a strong health_system, with an approach centered on people’s needs, in order to protect and improve their quality_of_life from birth to the end of life. The integrated management of citizens’ pathways in the nhs, reinforcing continuity of care and mechanisms for service integration, plays an essential role in person centrality. The United Nations 2030 Sustainable Development Agenda presented, as one of its objectives, reducing the impact of noncommunicable diseases on individuals and society, namely reducing premature mortality from noncommunicable diseases by one third by 2030, for which it is essential to provide an integrated response that focuses on health promotion and disease prevention in relevant sectors, of which chronic kidney disease is a typical example. Considering scientific evidence and best practices, it becomes necessary to implement preventive and therapeutic care plans that enable greater personalization, integration, and proximity, greater use of home treatments, greater access to renal transplantation, better information management, timely use of digital health, as well as legal instruments for financing and contracting of production and quality, aligned with a citizen_centered service vision. With a view to promoting person_centered innovation, with process transformation for the effective integration and individualization of responses for people with chronic kidney failure, the strategy is defined according to the following 5 axes: prevention and access; personalization and safety in care delivery; integration of care; information management and outcome evaluation; health communication. In personalization and safety in care delivery, with a view to ensuring a care model centered on the person with chronic kidney disease, it is necessary to promote therapeutic individualization, with effective quality management of the processes of patient choice and consent for the treatment modality, alongside a safe surgical pathway for creating vascular and peritoneal access for dialysis, and safety and personalization both in dialysis initiation and in the transition between treatment modalities, always adjusted to the objectives and to the personalized_care_plan. It is also important to differentiate the therapeutic offer in light of the most recent scientific information and value creation in health, promoting greater development of home dialysis programs and living donor transplantation. In care integration, for the sustainability of care delivery in chronic kidney disease, and of its quality, it is necessary to implement organizational and clinical quality lines that promote care integration. The care model must take into account a group of variables that, unequivocally, being foundational in care integration, condition the quality of the patient pathway: referral to hospital care, follow_up of patients after hospital discharge, avoidable hospitalizations, avoidable emergency visits, telemedicine, personalized_care_plan, integrated_care_pathway, referral to continued care, referral to palliative care, referral to mental health care, referral to physical and rehabilitation medicine care, networked care, end_of_life support, decentralized hospital consultations, and home consultations. For better management of transitions between treatment modalities in the pathway of the chronic dialysis patient, with resource optimization, integrated dialysis units are desirable, which offer personalized_care_plan integrating in_center hemodialysis, home hemodialysis, peritoneal dialysis, assisted treatments in stages of loss of autonomy, and conservative nondialytic treatment in elective patients, thereby promoting pathways that improve the quality of care provided and the response to the needs of patients and families. Information management and outcome evaluation: The collection and management of health data are crucial requirements for quality governance and appropriate decision_making in health_system. Information management must support financing models based on quality and value in health, aligned with recognition of the relevance of the national health ecosystem for the health economy, in the sector of chronic kidney disease, in a person_centered integrated care model. The strategy defines objectives and targets for the 3 year period that enhance the improvement of clinical quality, the efficiency of delivery, and centrality of the patient in the care pathway in health units, corresponding to the strategic axes. Objective: personalization and safety in care delivery: promote the improvement of care quality in hospital nephrology services with a view to increasing dialysis_free survival, reducing the number of hospitalizations, and reducing unplanned dialysis initiation in the trajectory of advanced chronic kidney disease. Ensure access to chronic renal replacement modalities. Implement the management of processes of choice, access, and initiation of chronic renal replacement therapy, focusing on improving clinical outcomes and on the promotion, supported by evidence, of renal transplantation and home dialysis. Define treatment plans and therapeutic individualization within a shared decision_making process. Care integration: Promote, within the nhs, the integration of healthcare in chronic kidney disease and the continuous improvement of processes, focused on avoiding hospitalizations and adverse events. Strengthen home hospitalization in the treatment of people with chronic kidney disease. Promote integrated dialysis treatment units offering hemodialysis, peritoneal dialysis, and conservative nondialytic treatment, in elective patients. Promote patient empowerment using proximity services and home dialysis, including assisted treatments in stages of loss of autonomy and end of life. Promote continuity of care and the management of transitions between treatment modalities, in the patient pathway with resource optimization. Enhance primary healthcare, the nhs mental health program, the national network of continued integrated care, and the nhs palliative care network, in the scope of treatments for chronic kidney disease and in end_of_life management. Information management and outcome evaluation: Ensure knowledge of the epidemiology of chronic kidney disease and risk stratification. Promote the use of telemonitoring in service delivery to patients with advanced renal failure and in home treatment, with a view to greater proximity and efficiency. Invest in digital interoperability in information management for chronic kidney disease. Integrate patient_reported outcome measures into assessments of quality and cost_benefit of treatments. Contract production and quality in the chronic kidney disease sector, within primary care, hospital care, and dialysis units. Review the financing model for dialysis units and adjust it to care differentiation and integration. Obtain recognition of the relevance of the national health ecosystem for the health economy and populations, in the chronic kidney disease sector. Health communication: Implement risk management measures for acute kidney injury and chronic kidney disease in primary healthcare, with information campaigns and alerts. Promote continuous training for clinicians who intervene in the pathway of the person with chronic kidney disease. Use digital media with information validated by scientific societies directed at patients and citizens in general. Ensure the quality of the process of choosing renal replacement modalities in advanced chronic kidney disease.

**** *id_34 *doc_3 *year_2023 *author_2 *area_3

Aiming for a fairer and more inclusive nhs that better responds to the population’s needs, the Government will continue its action to: Improve access to consultations and to health promotion and disease prevention activities through multidisciplinary interventions suited to each citizen’s characteristics, namely in accordance with the strategies and guidelines of the national_health_plan 21_30. Create a national structure for health promotion. Improve the organization and articulation of public_health services by creating mechanisms for greater integration between nhs structures, civil protection, the social sector, and civil society actors with direct and indirect intervention in health. Approve a new model for organizing responses to addictive_and_dependency_behaviors by integrating planning, coordination, and intervention competencies. Foster the use of telehealth as a proximity response to citizens’ needs, create a national telemedicine center and a national telemedicine network, as well as promote the use of the nhs_24 contact center as an entry and referral point in the nhs. Promote the integration and continuity of user_centered care through information systems, especially through the creation of a single electronic_health_record that integrates different levels and sectors of care delivery, allows access to the citizen’s relevant clinical information at any point in the nhs network, and promotes the citizen’s autonomy in managing their own health process. Strengthen community_care_units due to their relevance in supporting the most vulnerable people at home and in the community.

**** *id_36 *doc_5 *year_2023 *author_2 *area_3

The scope of the national plan for the addictive_and_dependency_behaviors reduction 2030 is the addictive_and_dependency_behaviors, taking into account the new challenges that arise in this area and also the plan’s core principle, which is citizen centrality. Included in the concept of addictive_and_dependency_behaviors are: the use of illicit psychoactive substances and new psychoactive substances; the use of licit psychoactive substances such as alcohol, tobacco, or medications; excessive behavioral patterns associated with gambling, whether for money or not; problematic use of the internet; and other potentially addictive behaviors that may come to be enshrined as such in disease classifications, given the evolution of consumption and observed behavioral addictions. National policy on intervention in addictive_and_dependency_behaviors has been characterized by the principles of humanism and pragmatism as the basis of all guidelines, and by the quality of the intervention developed by public and private institutions in the scope of addictive_and_dependency_behaviors. Considering citizens’ rights, namely access to information and to specialized and appropriate services for each situation, the approach is developed with a special focus on citizen centrality, while also guaranteeing the principles of equality and equity. Strengthen literacy in addictive_and_dependency_behaviors, equipping the citizen with information, knowledge, and skills with a view to making informed decisions and generating healthy and safe behaviors. The promotion of literacy in addictive_and_dependency_behaviors is decisive for the citizen to be empowered to make appropriate health decisions, providing more efficient and rational use of health_system resources. Literacy in addictive_and_dependency_behaviors must be established as a priority today, insofar as it contributes to changes in behaviors and attitudes and to the healthy and safe development of children, adolescents, and young adults, and consequently to a reduction in the consumption of licit and illicit psychoactive substances. Promote social inclusion and empowerment for the exercise of informed citizenship among the most vulnerable populations with addictive_and_dependency_behaviors. Citizens with problems of addictive_and_dependency_behaviors are often in situations of social exclusion, marked isolation, and social rejection in relation to the environment in which they live. In promoting their social inclusion, the existence of positive interactions with society is fundamental, which implies the establishment of connections between people and their social context, in order to create or repair social bonds between citizens and social systems. Over recent years, integrated intervention models centered on the real needs of the citizen at risk of exclusion have been advocated. Create conditions in the external environment to promote lower risk or healthier behaviors. Literacy initiatives and an inclusive and diverse culture at the level of the external environment are an essential complement to more citizen_centered interventions, contributing decisively to the removal of barriers and to promoting access to true citizenship, with full incorporation of human rights.

**** *id_37 *doc_3 *year_2024 *author_2 *area_3

The enipssa 2017_2023, created by the Resolution of the Council of Ministers number 107.2017, aimed to consolidate a strategic and holistic approach to prevention and intervention, centered on people in situations of homelessness, so that no one has to remain on the street due to a lack of alternatives. The enipssa, presented below, intends to introduce a set of improvements compared to previous programmatic cycles, particularly regarding: focusing on preventive measures, since this domain of prevention of the phenomenon is as important as the way we collectively intervene if such a situation occurs; advancing the changes already initiated, avoiding disruptions in the implementation of a comprehensive policy of prevention and combat against this phenomenon, namely by adopting a people_centered model, ensuring the expansion, creation, and innovation of community_based services, the identification and proliferation of awareness_raising actions, innovative ideas, and the sharing of inspiring practices; strengthening, diversifying, and updating the territorial support networks for people at risk or experiencing homelessness, through the planning and intervention units for homelessness, resizing their intervention toward prevention, ensuring their close relationship with other services and teams operating on the ground; ensuring specialized intervention with particularly vulnerable groups such as the elderly, people in dependency situations, people with disabilities, people in need of mental health care, people who use and are dependent on alcohol or illicit substances, lgbti_plus people, Roma people, migrant populations, among others; promoting the assumption of collective and intersectoral responsibilities and competences, including those related to prevention, ensuring multilevel monitoring of the implementation of the enipssa, awareness of social dynamics, and continuous strengthening or updating of measures and their interrelation, as well as effective sharing of resources; Redefining, strengthening, and simplifying the governance model to allow effective monitoring of the enipssa and its progressive and flexible implementation; Improving monitoring and evaluation mechanisms and instruments and developing methodologies that allow continuous and transparent reporting of measure implementation. Vision: Consolidate a strategic, integrated, multidisciplinary, and holistic approach to prevention and intervention in relation to the phenomenon, ensuring an efficient and effective action centered on people, so that no one experiences homelessness, much less remains on the street due to lack of alternatives. Ensure that the population has access to support measures and services capable of promoting the prevention of homelessness or, whenever it occurs, to integrated support in the different biopsychosocial and environmental dimensions, so that, through quality care, social inclusion is achieved. Mission: Create and implement an ecosystem close to people at risk or experiencing homelessness, ensuring equitable access, in quantity and quality, to support services, through territorialized responses and personalized intervention. General objective and principles: Multilevel prevention of the phenomenon, promoting the definition and adoption of preventive and intervention measures for people at risk or experiencing homelessness, aiming at early intervention to prevent or minimize risk and its effects; Development of an approach centered on the person, on human rights, and on the realization of self_determination and human dignity; Active participation of people at risk or experiencing homelessness in the entire planning, intervention, and evaluation process; Guarantee of citizenship rights and duties; Promotion of non_discrimination and equality, namely safeguarding gender identity and combating discrimination based on sexual orientation, gender identity and expression, and sexual characteristics; Strengthening of reflective, updated, and innovative knowledge strategies on the dimension and nature of the phenomenon that support the development of sustainable prevention and intervention strategies; Promotion of recognition and deepening of the multidimensionality and complexity of the phenomenon and the consequent need for adaptation and persistence in the implementation of measures; Definition, implementation, and permanent updating of prevention, combat, intervention, and follow_up measures, ensuring their equity; shared responsibility and mobilization of the set of resources and actors, namely public and private entities of civil society, in a logic of complementarity and subsidiarity, for integrated and comprehensive, consistent, and effective prevention and intervention, to ensure accessibility to existing resources, services, responses, and care, including ensuring a rational and sustainable distribution of resources and combating waste, particularly food waste; Recognition and adaptation to the specificities and heterogeneity of the various groups that make up people at risk or experiencing homelessness, including according to gender, sex, disability, and vulnerability; Guarantee of quality prevention and intervention centered on the person, safeguarding the reservation of their privacy throughout the entire support and follow_up process, personalizing the intervention and ensuring the provision of territorialized solutions according to each person’s needs and potential; Proactive participation and promotion of the empowerment of people at risk or experiencing homelessness at all levels of the social inclusion process; Prioritize a housing_oriented approach, centered on facilitating access to stable, preferably permanent solutions, and their suitability to the person’s life project; Ensure planned deinstitutionalization, promoting processes of transition to community life and self_determination of people upon leaving institutions, including prisons, health units, shelters, and others; Awareness, education, and mobilization of the community regarding the phenomenon; Continuous monitoring of the process and evaluation of the implementation results of the enipssa. The proposed model is therefore based on the centrality of the person as a whole and in their life context, which is intended to be integrated and comprehensive, and aims at preventing new situations, providing close follow_up, based on the premise of qualification and optimization of human and financial resources, namely to avoid duplication of responses.

**** *id_38 *doc_5 *year_2024 *author_2 *area_3

The Action Plan for Active and Healthy Ageing constitutes the guide and will be the driving force of transformation in Portuguese society, with the purpose of ensuring the best conditions for all, aiming to maximize longevity and quality_of_life, leaving no one behind and focusing on people. Subpillar integrated and long_term care: measures are highlighted to facilitate access to care through new technologies and helplines, such as the nhs_24 line and nhs_24 service desks, in addition to the creation of new responses such as the 60_plus line, to address the specific needs of senior citizens, and the 60_plus managers. Concrete measures are intended to improve the quality of services provided with a focus on the user, including some innovations for this networked structure, such as the inclusion of external automatic defibrillators, the training and empowerment of healthcare_professional, improvement of working conditions, and more training and differentiation of teams.

**** *id_40 *doc_5 *year_2024 *author_2 *area_3

Thus, the experience gained during the pandemic demonstrated the importance of care integration, the need for articulation, coordination, and collaboration among the different stakeholders of the regional health service and society, particularly the social sector. Compared to 2020, it was found that closer coordination of services is possible, coordination centered on the user. The improvement of some digital responses, such as the use of the Azores health line, telemedicine, digital issuance of prophylactic isolation, the notification system for positive cases, among others, opened the doors for the unification of the user’s clinical record, for the improvement of epidemiological surveillance of communicable diseases, and for the diagnosis of the health situation of Azoreans, allowing for the possibility of health planning that effectively responds to citizens’ health needs and is not centered on health services. The conceptual model of the Regional Health Plan 2030 is inspired by Myosotis maritima, an endemic plant of the Azores found on the rocks and sea cliffs of our islands. At the center are the person, the family, and the community. It is intended that all planning and action focus on this triad and its concrete needs. Hence, the first step to improving the health of Azoreans is the identification of each person’s concrete needs. Only with this identification can what is offered be properly planned, meeting their needs with efficient and effective use of resources. For this, it is important to ask What matters to you? and not What is wrong with you? It is also important that the needs of this central triad be the focus of attention not only of the health sector but also of society as a whole, in the perspective that health depends on a complex interaction of factors. The immediately following layer is that of care integration. In the current complexity of health_system and the individual circumstances of health and disease, also increasingly complex, it is imperative to act in an integrated manner among all participants, allowing a continuum of care in which the user feels guided, safe, and confident that the entire system works and is organized around their needs. For the health_system, care integration allows each healthcare_professional to feel valued and integrated into a value chain, resulting in better healthcare. Efficiency gains can be achieved by eliminating duplications or unnecessary interventions. It is also possible to increase safety in care delivery by integrating information, allowing for more informed and supported decisions. Due to the negative impact caused in Azorean society by addictive_and_dependency_behaviors, the existence of a Regional Plan for the Reduction of addictive_and_dependency_behaviors, was defined as strategic. This plan has the vision of consolidating and deepening an integrated and effective public policy in the field of Prevention of addictive_and_dependency_behaviors, based on intersectoral coordination, aiming at health and well_being gains in society. Its guiding principles are citizen centrality, humanism and pragmatism, integrated intervention, and knowledge and innovation. It employs different intervention strategies, universal, selective, indicated, global, specific, and targeted_organized along the life cycle. The local health plan has the mission to: Be a mechanism for operationalizing regional health strategies, particularly regarding priority interventions considered in each locally eligible program; Identify the local health problems and needs of the population; Serve as a reference framework for community health policies, adopting strategies suited to the population’s health needs; Promote and consolidate collaboration among the various local health entities; Integrate and coordinate partners’ efforts around common objectives, strengthening an intersectoral approach and health in all policies; Be assumed as a social commitment, encouraging citizens to be co_producers of health policies; Enable monitoring and evaluation of the regional health plan as a whole. In the current context, the promotion of health literacy has gained renewed importance among people, communities, organizations, and healthcare_professional, constituting a necessary response and a tool of public_health. Thus, health literacy constitutes in this plan both a goal and an opportunity to promote health throughout the life cycle, being operationalized in its own program, with the vision of empowering and holding each individual accountable for their health journey.

**** *id_46 *doc_3 *year_2021 *author_1 *area_1

The interpersonal relationship between a nurse and a person or group of people, family, or communities constitutes the core of professional practice. In this context, both the nurse person and the client person possess frameworks of values, beliefs, and desires of an individual nature that make them unique beings and each interaction singular and distinct. The person, client of care, is understood as an intentional agent with behaviors based on values, beliefs, and desires of an individual nature, with inherent dignity and the right to self_determine. Within the scope of specialized care in maternal and obstetric health nursing, the client person who immediately stands out is the woman. In the Regulation of the Specific Competencies of the Specialist Nurse in Maternal and Obstetric Health Nursing, it is asserted that nursing care focuses on promoting the health projects that each woman defines for herself, understood both in an individual and collective perspective. The woman is, therefore, understood as the person as a whole, considering the interaction with significant others and with the environment in which she lives and develops. The environment is composed of human, physical, political, economic, cultural, and organizational elements that influence health. As a client of the specialist nurse in maternal and obstetric health nursing, the woman may assume different specificities in terms of care needs: pregnant woman, parturient woman, mother, puerperal woman, adolescent or adult woman, in the process of aging. In this context, the woman influences and is influenced by the environment in which she grows and develops, by the people with whom she interacts, and by the society in which she is integrated; respect for the capacities, beliefs, values, and desires of the individual nature of the woman and of the people who are significant to her; respect for the expectations related to labor and the birth of the child; respect for the expectations and desires of an individual nature related to the maternity and paternity project; establishing partnerships with the client in the planning of the care process; establishing a therapeutic relationship with the woman and the people who are significant to her, having as references the competencies of clinical communication. Throughout the pregnancy period, the man_father is also understood as a client of care, considered individually, also with values, beliefs, and desires, with inherent dignity and the right to self_determine. In this context, we may also consider the pregnant couple as a client of care, as a unit of 2 people with their own and individual needs, but who share the project of parenthood and conjugal life. During the postpartum period, the puerperal woman and the mother constitute 2 relevant aspects of care needs centered on the woman. In this period, the newborn and the man_father and or partner also emerge as clients of care. Nursing care aims at promoting the achievement of each client’s health project. The therapeutic relationship between the specialist nurse in maternal and obstetric health nursing and the client is one of the central elements so that the client may be proactive in promoting her health, preventing disease, and adapting to new life circumstances. Another key element is the mastery of competencies to respond to the specific needs of each client, at each moment and context of care. In the continuous pursuit of excellence in professional practice, the specialist nurse in maternal and obstetric health nursing helps clients reach their maximum health potential, with relevant elements for health promotion, among others: assessment and diagnosis of the specific care needs of clients; identification of available health and social resources. The specialist nurse in maternal and obstetric health nursing is the one who demonstrates high levels of clinical judgment and decision_making, reflected in a set of specific competencies in maternal and obstetric health nursing and who assumes responsibility for differential diagnosis and implementation of interventions, within the following domains: establishing partnership with the significant person as an ally in care planning centered on the client; health promotion aims at empowering and enabling each person to act in improving their quality_of_life and health. The prenatal care model is centered on the pregnant woman, the person with whom she shares the maternity project, and the family. General principles are assumed that guide the conception of specialized care in prenatal assistance centered on the pregnant woman and the person with whom she shares the maternity and family project. Communication is the foundation of the therapeutic relationship; clinical communication between the specialist nurse in maternal and obstetric health nursing and the client is essential and must always be conducted with kindness, respect, and dignity. Opinions, beliefs, and values related to her self_care and the care of her child must be identified and respected at all times. The woman must have the opportunity to make informed decisions, in partnership with the specialist nurse in maternal and obstetric health nursing, regarding prescribed care, supported by evidence_based information and adapted to specific needs. Quality standards of specialized care in maternal and obstetric health nursing: respect for the capacities, beliefs, values, and desires of the individual nature of the woman and of the people who are significant to her; respect for the expectations related to labor and the birth of the child; respect for the expectations and desires of an individual nature related to the maternity or paternity project; the commitment of the specialist nurse in maternal and obstetric health nursing to empower the client for decision_making and action; the commitment of the specialist nurse in maternal and obstetric health nursing to establish partnerships with the client and the significant person for empowerment in decision_making and action; the involvement of the significant person in the care process; the commitment of the specialist nurse in maternal and obstetric health nursing to consider in the care process the specific needs of the significant person as a client of care; establishing partnership with the significant person as an ally in care planning centered on the client; taking every opportunity to provide the woman, the person with whom she shares the maternity and family project, with the information and support they need. Care must be woman_centered: the focus of care must be to meet the needs of the woman and her child; each woman must negotiate the level of involvement she desires from the significant person and family or friends. Prenatal nursing consultation is defined as a care context, designed and implemented by a specialist nurse in maternal and obstetric health nursing, centered on the needs of the pregnant woman and the person with whom she shares the maternity and family project. In each consultation, it is important to define, in partnership, an personalized_care_plan that promotes a healthy pregnancy experience, facilitates the transition to parenthood, and favors the active participation of the pregnant couple throughout the process.

**** *id_48 *doc_4 *year_2022 *author_3 *area_1

Assuming the protection of the older person and their care needs as the central element of this strand of a system that brings together social protection and health protection for a particularly vulnerable population, this framework intends to contribute to implementing, in each erpi, an approach aimed at the recovery and maintenance of the best possible levels of health for each older person, as well as the development of a learning culture in this area. Health simultaneously involves different dimensions: biological, psychological, social, cultural, among others, and components, requiring a multicausal and multidimensional approach, based on a provider system organized around a central element, the older person, understood as a whole in the context of a holistic vision and a broader approach directed at the recovery or maintenance of the best possible levels of health in view of the processes of aging. To achieve an improvement in the impact of care, investment in coordination between health and social security is essential, in a coordinated manner and centered on the person and their care needs, so that the best response is found to satisfy their needs. Integrated intervention at the local level also allows for better knowledge of reality, faster and more efficient responses, and the development of more appropriate and proximity solutions, which are essential for decision_making processes related to the well_being of older people. The various levels of care should not be regarded as substitutes for each other, but as complementary, interconnected, effectively covering the individualized needs of each user, who should be able to move within an interdisciplinary dynamic, according to health needs. Depending on the specific needs of each person and the underlying conditions that lead to loss of functionality, a combination of all or some of these services will be necessary, ranging from outpatient and home support, through residential institutions for older people, health services, and integrated continued care units. Good practices in this field recognize that older people are not a homogeneous group, have different needs, preferences, and opportunities throughout their lives, that violence against these people can manifest itself in various ways, including abandonment and neglect, often occurring in the home environment or in institutional contexts, and that the expectations and needs of older people should be considered at different levels of planning and decision_making, as well as ensuring access to proximity, individualized, person_centered care integrated at different levels of care, with their participation and involvement. The desired integrated approach to aging implies not only combating ageism in its various forms, but also promoting access to quality and long_term care, centered on the wholeness of people and not on disease, ensuring integrated intervention by the areas of health and social security that also contemplates the measures set out in the strategy for the rights of persons with disabilities 2021_2030, thus paying particular attention to older persons with disabilities. To ensure access to care centered on older people, systems must know and organize around their needs and preferences, ensure family involvement and consequent assumption of their responsibility, of other caregivers and the community, integrate different services, and ensure long_term care. The main actions that can help achieve this goal are: comprehensive assessment of the health of older people, preparation of an personalized_care_plan to optimize their capacity, development of proximity services near their place of personal or institutional residence, provision of community services, creation of service structures that promote care by multidisciplinary teams, support for self_care, and increased health literacy. To provide quality and integrated healthcare, healthcare_professional must develop gerontogeriatric competencies, as well as the competencies necessary to work with the health_system, including teamwork and information and communication technologies. The erpi should, in their operation, articulate with different community mental health services or teams, thereby contributing to ensuring that the provision of mental healthcare is centered on the older person, recognizing their individuality, specific needs, and level of autonomy, as well as avoiding their stigmatization, negative discrimination, or disrespect in the health context, as set out in the law number 113_2021, of 14 December. In order to improve the quality of healthcare provided in the erpi, the need to implement an effective change in the care delivery model has been evidenced, abandoning a reactive vision and implementing a proactive approach centered on the needs of the older person, their families, and the caregiver and healthcare_professional involved, defined by the erpi health team, permanently updated and reflected in the records existing in each person’s personalized_care_plan. This vision presupposes recognition of the importance of articulation with primary healthcare in whose geographical area the erpi are located, particularly with regard to identifying and understanding the health needs of the population residing in the different erpi, thereby enabling the response capacity to the identified health needs to be enhanced. Information systems constitute a central instrument in the provision of healthcare, contributing to its continuous quality improvement through clearer and more complete records. The integration of health information and clinical information into information systems by duly qualified healthcare_professional also contributes to the continuity of care centered on the needs of the older population and of each resident individually considered. The erpi are, in view of population aging and the growing need for continued care in a home context, a growing component in the social protection and health_system. In this context, it will be essential to develop and implement a digital platform that allows, through an access policy designed in accordance with legislation on the protection of and access to personal and health data, contribution to the centralization of health, clinical, and social information necessary to support care and decision_making processes. In a model proposed as centered on the needs of the population residing in erpi, information systems should integrate 3 essential areas. Clinical records are a central essential element in the provision of healthcare, constituting a deontological duty of the different health professions, which are obliged to record in a clear, complete, and precise manner the observations, diagnosis, interventions, and information considered appropriate for each person. With regard to the erpi, and given the centrality of healthcare, information systems should include data resulting from assessment from the older person, the formulation of diagnoses, objectives, and interventions. The complete recording of observations and interventions carried out constitutes an essential duty for the quality, safety, and continuity of care.

**** *id_49 *doc_2 *year_2014 *author_3 *area_1

In this report a new approach to health promotion is proposed, decisively centered on the initiative of citizens and society at large. It aims to reduce the incidence and duration of chronic diseases such as diabetes, both parameters are higher in Portugal than in most Western European countries, and to show how methodologies for quality improvement and increased access to scientific evidence improve health services and reduce expenditure. The report proposes a transition from the current system, centered on the hospital and on disease, in which all actions have the patient as object and target, to a system centered on people and based on health, in which citizens are partners in health promotion and in healthcare. The system will use the most up_to_date knowledge and technologies and will provide high_quality counselling and services at home and in the community, as well as in hospitals and specialized centers. This vision incorporates the founding values of the nhs and is developed based on the strengths of the current system, on the competence of healthcare_professional and on past achievements, but it requires new approaches, a different infrastructure, and a lower and more sustainable cost base. A change of this scale requires visionary and courageous leadership, capable of uniting people around a new pact for health, setting the route to follow, and rallying political and public support. It also requires a broad change program, led by an alliance grounded in the various sectors of society, with new systems of continuous learning, evaluation of scientific evidence, and implementation of improvements. This change must be supported by a temporary transition fund to finance the cost of redundancy of facilities during the development of the new system. Citizen participation. Health begins at home: people will have to intervene much more actively in managing their own health and contribute to shaping the entire system. Citizens will have to be in possession of their health records, have information on the quality and costs of services, and participate in decision_making processes. A people_centered and team_based health_system. New service models are needed to provide integrated healthcare to all individuals, with special emphasis on chronic disease management; development of more home and proximity support services; and creation of specialty networks, associated with reference centers but covering all regions of the country through technology and shared protocols. New roles and strengthened leadership at all levels. Healthcare_professional are oriented to become agents of change and improvement, but leadership is needed in communities and in health services, and leaders must cooperate with each other. All healthcare_professional must come to perform new roles, and their training should be adapted accordingly, while patients and community organizations need support to assume expanded leadership roles. This report proposes the transition from the current system, centered on the hospital and on disease, in which all actions have the patient as object and target, to a system centered on people and on health, in which citizens are partners in health promotion and in healthcare. The system will use the most up_to_date knowledge and technologies and will provide high_quality counselling and services at home and in the community, as well as in hospitals and specialized centers. A change of this scale requires visionary and courageous leadership, capable of uniting people around a new pact for health, establishing the route to follow, and rallying political and public support. Vision: The Commission found broad consensus both on the general contours of the vision for the future and on the need for radical reforms. Those consulted referred in particular to: the need to place greater emphasis on disease prevention; the shift from a system based on hospitals and healthcare_professional to a system based on the community and on people; citizen empowerment and health literacy; telemedicine and personalized medicine; healthy societies and environments; the cultural transition from pure medical paternalism to a responsible partnership. Many people also advocated the need for better information and greater transparency. Citizens are in a position to play a much greater role in relation to their own health and that of their families, as well as in improving society’s health. All structures and sectors of society, education, environment, social security, commerce and employment, as well as health, work together to promote health and well_being. There is a commitment to continuous quality improvement and to the systematic application of scientific knowledge and technologies in support of health and well_being. This vision represents a transformation of the current system, centered on hospitals and on disease, in which all actions have the patient as object and target, to a system centered on people and based on health, in which citizens are partners in health promotion and in healthcare. This system will use the most up_to_date knowledge and technologies to support the promotion and improvement of health and to provide high_quality, reliable services throughout the country. These are the key_strategies for change, with the first 3 explicitly embedded in this vision: A new pact for health. A change of this scale requires visionary and courageous leadership, capable of uniting people around a new pact for health, setting the route to follow, and rallying political and public support. It also requires a broad program of management change, supported by new systems of continuous learning, evaluation of scientific evidence, and implementation of improvements. This change must also be supported by a temporary transition fund to finance the cost of redundancies during the development of the new system. Citizen participation. Health begins at home; people will have to intervene much more actively in managing their own health and contribute to shaping the entire system. Citizens will have to be in possession of their health records, have information on the quality and costs of services, and participate in decision_making processes. Contribution of different sectors of society. Modern societies actively promote the consumption of unhealthy lifestyles, and modern environments make healthy choices difficult. To improve health, initiatives are needed from all sectors of society and from citizens, as well as from the Government and local authorities. Timely, high_quality, evidence_based treatment services benefit patients and, moreover, reduce waste and expenditure. The emphasis should be on continuous quality improvement, systematically applying evidence of proven practices, everywhere. Portugal must position itself to take maximum advantage of future advances in knowledge and technology and further develop its own biomedical research. A people_centered and team_based health_system. New service models are needed to provide integrated healthcare to all individuals, with special emphasis on: chronic disease management; development of more home and proximity support services; and creation of specialty networks associated with reference centers and covering all regions of the country through technology and shared protocols. New roles and strengthened leadership at all levels. Healthcare_professional are oriented to become agents of change and improvement, but leadership is needed in communities and in health services, and leaders must cooperate with each other. All healthcare_professional must come to perform new roles, and their training should be adapted accordingly, while patients and community organizations need support to assume expanded leadership roles. The main share of health_system costs arises above all from care for people with long_term chronic diseases. Financial sustainability will only be achieved by reducing the incidence of these diseases and the morbidity associated with them, developing new models of healthcare for them, and ensuring that scientific evidence is applied systematically everywhere and that waste is minimized. The introduction of new financial mechanisms and results_based incentives, if well managed, may be useful; but the financial viability of the system will depend on the will to include health in all policies, on effective health promotion, and on concerted initiatives by citizens, society at large, and healthcare_professional. In the future, as this report argues, citizens should be active partners in the promotion and protection of health and in the provision and design of healthcare. The former implicit social contract, under which the population was the object and the target of all actions, should be replaced by a new pact for health in which everyone takes an active part. The commission suggests that the Government initiate the creation of a new pact, but that the proposed national_health_council be tasked with its development and act as adviser regarding its final form. Citizens as partners and active participants; healthcare_professional who make their knowledge and skills available: ensuring the application of scientific evidence and rigor, as well as operating with high quality standards; Organizations across society that align their policies and practices to promote health and prevent disease; Creation by the Government of the structure and framework for a sustainable, high_quality health_system; national_health_council: an alliance of the whole society, which will have stewardship of the pact and the vision for the future and will provide advice on health policies. This proposal to create the national_health_council complements the roles played by the ministry_of_health and other entities in the leadership and management of the nhs. A final recommendation of this report calls for clarification of the accountability of the supervisory and management structures of the health_system. It is necessary that these 2 elements, an advisory body bringing together all these sectors and an executive structure that supervises and manages, function well, in order to provide the leadership and guidance necessary for the entire health_system, implementing a broad program of change. A new pact in health should be established, configuring the new interactions and performances necessary for the transformation of the current system, centered on the hospital and on disease, in which the patient is the object and target of all actions, into a system centered on people and based on health, in which citizens are partners in health promotion and in their care. The national_health_council should be established as an alliance of the whole society with the responsibility of stewarding the pact for health and defining the vision for the future, having an overall perspective of the system and acting as adviser for policies that fit within that vision. This body should be representative of citizens and of all sectors of society, be politically independent, and be accountable to the Assembly of the Republic, the ministry_of_health, and the population at large. The nhs_evidence will be a new body that will combine the existing program of clinical practice guidelines with new processes for assessing new technologies and therapies, ensuring that the system provides, in an always up_to_date and critical way, the best scientific knowledge available. This body should disseminate its decisions and the results it holds to citizens so that they, as well as doctors and other healthcare_professional, can know this evidence. Create a transition fund that can help bear the costs of redundancy and other provisional costs in the development of a health_system with better management of chronic diseases, more home and proximity services, and specialty networks covering all regions of the country. This chapter focuses on creating health and on what individuals and their families, caregivers, and friends can do. These 2 objectives, that citizens become more active in relation to health and that the structures and sectors of society collaborate to promote health and well_being, are at the heart of the Commission’s vision for the future. Together they can co_produce better health in Portugal. This report underlines the importance of citizens assuming greater control and responsibility. It recognizes, however, that there are cultural, market, and environmental issues that may pose difficulties and constitute barriers that must be overcome. Almost all of the Commission’s prior contacts highlighted these and other related aspects, but also reflected on the difficulty of effecting this change in practice. The Commission was informed that in Portugal the population is generally very passive in its relations with the health_system; it does not feel able to question doctors and makes very limited use of sources of health information. There were many calls to promote a change in this culture, both from the population and from healthcare_professional. Commission working group 2 advocated a paradigm shift, from a paternalistic health_system to a system that places citizens at its center. The commission was also informed that, in practice, there is very little information available to citizens, for example with regard to the quality of services and to transparency in statistical data and clinical practices. This lack of statistical data and transparency is a relevant problem in Portugal and will be referred to frequently in this report. The way we have approached medicine over the last 100 or more years, as we have increasingly handed control to healthcare_professional, has consisted of seeking safeguards from the State through regulation and oversight, relying on healthcare_professional, on their Hippocratic Oath, and on their social mission. This approach is no longer sufficient on its own and requires greater transparency and more active participation by citizens and patients in the governance of health_system and in the support and definition of their priorities. Many healthcare_professional and patient representatives told the Commission that it is necessary to abandon the traditional paternalism of healthcare_professional and of the health_system, evolving toward a more equitable relationship. This will have to be supported by changes in professional training and in the academic curriculum, and will also require higher levels of health literacy by all citizens, with health education starting early in children’s lives. This new approach will undoubtedly arouse resistance from some healthcare_professional, who were trained in a different environment and who may view it as a threat to their position and authority. Others, however, will see it as a positive evolution and will recognize the potential of better informed citizens willing to play a broader role in their own healthcare. These 4 recommendations will help create a new environment and a new culture in which citizens are at the center of the health_system, as referred to by the national_health_plan, and where they can be at the center of managing their own lives, as suggested by Working Group 2. There should be a new intersectoral Government program for health education and literacy that prepares citizens, in practical terms, to stay healthy and to participate in decision_making when ill. This program should have high visibility and be led by a group of Ambassadors, including media personalities, in coordination with the ministry_of_health and the ministry of education. High priority should be given to the full implementation of an electronic_health_record, as an essential basis for a high_quality integrated service that provides citizens, in electronic or other format, with all information about their health. There should be a single source of accredited information accessible to all citizens, possibly integrated into the Health Portal. It will contain information on health, disease prevention, services provided, and their quality. Health organizations should be obliged to provide certain information for this purpose, and regulatory entities should ensure that citizens have access to it. The ministry_of_health should appoint non_professionals to represent the perspective of citizens and patients before the management bodies of health institutions. The integration of services and the personalization of healthcare are recurring themes in this report. These themes become even more important as the population ages and more people suffer from chronic diseases and comorbidities. It is essential that healthcare for all individuals be well planned and coordinated, and not fragmented and incongruent. This chapter addresses how truly integrated, people_centered healthcare can be developed through 3 main groups of strategic actions: Creation of new service delivery models; Restructuring of the health_system; Partnerships in the planning and delivery of services. A health_system for the future must be centered on people and have the capacity to provide integrated healthcare to all individuals, which entails major changes. These changes provide the opportunity for great creativity in the development of new service models and for a new design of the health_system, in order to meet the needs of the 21st century. As this chapter has shown, Portugal already has many of the key_characteristics that will enable the improvement of services and systems. Some immediate measures are needed, however, to accelerate and consolidate the necessary changes. This chapter was based largely on the deliberations of Working Group 1 and on contributions from various stakeholders. Following discussions with this Group and the ensuing deliberations, the Commission decided to make 3 recommendations in this area. More leadership programs must be developed within the health_system, and these should occur alongside the development of leadership among the general population. Education professionals, citizens, and municipal leaders, for example, can contribute to improvements in health and well_being. Everyone, including healthcare_professional, can also contribute to broader improvements in society. Better management capacity is also needed across the system. Although senior managers may have the skills and experience necessary to perform their functions, it is generally middle and junior managers, involved in day_to_day decisions, who affect the service actually provided to patients. The quality improvement approach described in Chapter 4 will require the participation of these managers, who should be given decision_making powers, as well as of qualified and change_open healthcare_professional. In addition, more physicians need to have management skills and take on an enhanced role in organizing their services. Characteristics of transformative leaders or change agents: People_centered; Passion for improvement; Willingness to take responsibility and lead; Use of scientific evidence; Creative thinking able to overcome obstacles; Management of impacts on other individuals and practices; Use the system to achieve success. The Commission prepared 7 groups of recommendations intended to put Portugal on the path to creating the future of a sustainable, high_quality health_system. A new pact for health: A new pact in Health should be established, configuring the new interactions and performances necessary for the transformation of the current system, centered on the hospital and on disease, in which the patient is the object and target of all actions, into a system centered on people and based on health, in which citizens are partners in health promotion and in their care. national_health_council: The national_health_council should be established as an alliance of the whole society with the responsibility of stewarding the pact for health and defining the vision for the future, having an overall perspective of the system and acting as adviser for policies that fit within that vision. This body should be representative of citizens and of all sectors of society, be politically independent, and be accountable to the Assembly of the Republic, the Minister of Health, and the population at large. nhs_evidence: the nhs_evidence will be a new body that will combine the existing program of clinical practice guidelines with new processes for evaluating new technologies and therapies, ensuring that the system provides, in an always up_to_date and critical way, the best scientific knowledge available, the scientific evidence. This body should disseminate its decisions and the results it holds to citizens so that they, as well as doctors and other healthcare_professional, can know this evidence. Transition Fund: Create a transition fund that can help bear the costs of redundancy and other provisional costs in the development of a health_system with better management of chronic diseases, more home and proximity services, and specialty networks covering all regions of the country. Health literacy: There should be a new intersectoral Government program for health education and literacy that prepares citizens, in practical terms, to stay healthy and to participate in decision_making when ill. This program should have high visibility and be led by a group of Ambassadors, including media personalities, in coordination with the ministry_of_health and ministry of education. Ownership of personal health information. High priority should be given to the full implementation of an electronic_health_record, as an essential basis for a high_quality integrated service that provides citizens, in electronic or other format, with all information about their health. Access to information. There should be a single source of accredited information accessible to all citizens, possibly integrated into the Health Portal. It will contain information on health, disease prevention, services provided, and their quality. Health organizations should be obliged to provide certain information for this purpose, and regulatory entities should ensure that citizens have access to it. Representation: The ministry_of_health should appoint non_professionals to represent the perspective of citizens and patients before the management bodies of health institutions. Municipalities. Municipalities, some of which play leadership roles in the well_being of the populations they represent, should include health and health organizations in their local social care partnerships and support intersectoral work and planning. Civil society. Municipalities, health organizations, market organizations, and the appropriate ministries should work together to find better ways to promote, support, and involve in the health_system and caregivers. public_health. The Government, the relevant ministries, the nhs, and public_health institutions and associations should work together to strengthen public_health intervention at all levels and throughout the health_system. Public_health information and skills must be strengthened in the training and qualification of all healthcare_professional. New public_health models and legislative frameworks may be needed to achieve this purpose. Continuous quality improvement. All partners in the health_system, from the ministry_of_health and citizens to the various nhs bodies, municipalities, universities, and industry—must ensure that the approach to continuous quality improvement is adopted throughout the health_system as a means of promoting its quality and ensuring that scientific and technological advances are integrated and implemented in a systematic and widespread manner. Quality improvement competencies should be part of the training and education programs of all healthcare_professional. To this end, an expert body should be created, responsible for promoting quality improvement, identifying and disseminating good practices, and supporting their implementation. It should coordinate with other bodies, in health and in other areas of governance, to create models for implementing quality improvement, promote training, advice, and support, which should be accompanied by the standardization of accreditation, registration, and reporting processes. Collaboration of the nhs with research and industry. Leaders of the nhs, the scientific community, and industry should collaborate to create national centers that, working together with citizens, promote the development of new practices, technologies, and services. New models for healthcare. Policymakers, planners, and service providers should work together to create integrated care services with special emphasis on those aimed at chronic disease management, the provision of more home and proximity care, and the creation of specialty networks associated with reference centers and covering all areas of the country through technology and shared protocols. Accountability and administrative costs. Responsibilities need to be well established throughout the system, and an analysis is needed of the number and functions of many public bodies associated with health and care, reducing their number and respective costs by at least 25 percent, thereby freeing up funding for investment in other areas. Public_private agreement. A public_private agreement should be established as a framework for contracting private services by the nhs, safeguarding the public interest while simultaneously bringing new resources and potential innovations to the health_system. Professional training. The Ministries of Education and the ministry_of_health should establish a comprehensive project to review professional training in light of current and future developments in health and ensure that this redesign includes new needs for partnership with patients, quality improvement, and strengthening of public_health. Nursing. In Portugal, the status of nurses should be promoted through the appointment of a general director of Nursing, strengthening the role of these professionals in organizations and, as far as the budget allows, increasing their number. Following the introduction of the newly proposed figure of the family nurse, there should also be reflection on expanding the role of nurses and other professionals in other areas. Sustainability strategy. Adopt a sustainability strategy to improve quality and reduce costs, based on: Definition of 3 main areas of change: Reduction of morbidity, disease prevention, and shortening of illness duration; Effective application of scientific evidence and adoption of continuous quality improvement, with the goal of decreasing waste in health; Change in the infrastructure of the health_system. Develop the contracting and procurement process: focus measures on outcomes; continuously analyze international experience associated with the introduction of competition; and ensure sufficient flexibility of these mechanisms to meet the demands of evolving healthcare through new methods and forms of provision. Financial management. Improve financial management, adopt better governance, widely provide statistical data information, and strengthen accountability. It is further proposed to create a 5_year working capital fund, the nhs Stabilization Fund, which will serve as planning support, and to establish independent audits to evaluate services from a cost_benefit perspective, which will report to Parliament and to the national_health_council.

**** *id_51 *doc_2 *year_2024 *author_3 *area_1

The health information system is an essential instrument for achieving the desired health outcomes, both at the individual and population levels. It must serve the people who receive care and the healthcare_professional who provide it, meeting the needs and requirements of its users, and based on health units with greater effectiveness and efficiency. Humanization, personalization, safety, quality, and the integration of health data and information are essential principles to be respected. Currently, the health information of each person is dispersed across disconnected silos. The flow of information must be improved. Data and information must flow securely within the same organization and between different levels and types of care and different organizations. The centralization of healthcare on the user will only be achieved if the information circulates with the user. Considering the path of people within the health_system, the information system must be a facilitating instrument of integration, continuity, and coordination of care, especially when the person lives with chronic disease, regardless of the services and institutions that provide the care. It must also be an instrument for the continuous improvement of the quality of that care and its outcomes and promote self_care. The currently designated electronic_health_record may become a starting point and foundation for a future intelligent, integrative, and summarized personal clinical record. However, it is still far from the desired vision, both from the perspective of the user and of healthcare_professional, in their great disciplinary diversity and in the requirements of multidisciplinary work centered on the needs and objectives of care plans for each person. Achieving this vision requires a complex implementation process that must be accompanied step by step by feedback from the also complex and diverse universe of its users. The person is the main protagonist of their health and must necessarily be at the center of information and decision_making processes. That is, information collected about the person must always be available, regardless of where it was produced. It is thus considered essential that people have access to their information and make conscious choices about their health and well_being. With the premise of person_centeredness, and regarding information for health, it is important to reflect on certain aspects: health data accompany the life of the person, their collection presents opportunities but also risks, and data quality is critical for information to be reliable. In a collaborative and participatory health model, it is essential that people have access to their information and make choices about their health. The importance of the person being the main protagonist and partner of healthcare_professional and services in decision_making processes is increasingly recognized. But it is not enough for the person to be involved or to be a partner. They must be the center of attention and the main protagonist in any health and well_being model. The concept of person_centeredness is being used with increasing frequency and identified as a fundamental principle of all processes related to healthcare. It is also important to refer to the sustainable development goals, namely Sustainable Development Goal 3, which aims to ensure healthy lives and promote well_being for all, at all ages. Essential to this goal is the empowerment of the person, access to their information, fostering their individual capacity in decision_making and in changing behaviors for a healthy life. Based on the premise of person_centeredness and regarding information for health, it is important to safeguard and reflect on some aspects. Specific recommendations: Ensure, in practice, that the person is at the center of the system, participates in the process and or decision, and has access to information and to various health resources throughout life. Ensure that the person understands and uses the various resources for their own health or for those who depend on them, with the involvement of healthcare_professional or various actors, for better navigability in the health_system and active management of their health_disease and accurate decision_making, for better health outcomes. Ensure that the person has control over their health data and that effective mechanisms exist to ensure confidentiality and maintain the trust placed in healthcare_professional and services in the health sector. The digital transformation of healthcare can be disruptive; however, technologies such as the internet of things, virtual consultations, remote monitoring, artificial intelligence, big data analytics, blockchain, smart wearables, platforms, and other tools that allow data exchange and storage, remote collection, and sharing of relevant information throughout the health ecosystem that create a continuum of care have proven potential to improve health outcomes. Whether through improved diagnosis, data_driven decisions, clinical trials, self_management of care and person_centered care, or through the creation of more evidence_based knowledge and data and increased competencies of healthcare_professional. The linking of health data, both among different data sources in the health field and with external datasets, offers vast potential to advance understanding, management, and promotion of health. This integrated approach allows for a comprehensive and holistic view, enabling relevant information and improvements in healthcare and public policies. Comprehensive information on the user’s path: by linking data from different sources, such as electronic_health_record, clinical histories, and test results, it is possible to obtain a more complete view of the user’s path. This facilitates a deeper understanding of diseases, previous treatments, and individual responses to interventions by healthcare_professional. Personalization of healthcare: with data linkage, it is possible to develop personalized approaches to healthcare. By better understanding individual user characteristics, such as genetics, lifestyles, and health history, healthcare_professional can tailor interventions to meet the specific needs of each person. A health indicator can be conceptualized as a bridge between scientific knowledge and health policy. Adequate guidelines must exist to interpret the trends revealed by these indicators. A conceptual model of health and well_being is necessary to facilitate this interpretation, a model that focuses on people and communities, on health gains, on equitable access to the system, and on continuous qualitative development. Specific recommendations: Ensure greater integration of interoperability principles for indicators and health information, involving all actors of the health_system, and the acquisition of the most efficient informatic tools. Consider in this context the integration of health and social care, particularly regarding integrated continued care, since obstacles persist in the health pathways of patients discharged from hospital but without placement for rehabilitation, continued, or palliative care. Define new health indicators as a basis for system evaluation, moving away from the exclusive accounting of acts performed and focusing on observed health gains and integrating the perspective of the person and the community; include social indicators in the health information system. Effectively involve the person in building their own health, integrating a salutogenic and behavioral modification perspective, essential for the involvement of people and communities in the management of the health_system. Identify the information necessary and technically considered essential for the effective management of rare, chronic, or complex diseases and their comorbidities, to adapt the system to the needs and expectations of people and communities; equip the health_system with an effective medium and long_term planning strategy for the healthcare_professional required by the system, in number and competencies. A data governance model centered on people is fundamental, one that can ensure the effective portability of their information and rigorous security and confidentiality mechanisms. This data governance model centered on people is also the one that best serves its secondary use, ensuring the necessary trust in governmental structures to guarantee the confidentiality and protection of sensitive data, such as health data. See, recently, the launch of the HealthData_PT infrastructure. Specific recommendations: Ensure that the person can access and hold control over their health data, allowing healthcare_professional access and interaction in managing their health_disease. Ensure the implementation of a data governance model centered on people that can ensure the effective portability of their information and rigorous security and confidentiality mechanisms. There must be a clear political and social commitment to transform the nhs, as well as the health_system as a whole, based on evidence derived from available information and scientific knowledge, stimulating innovation and efficiency in a decentralized manner and with maximum adaptation to both national and local needs, with active involvement of people. A national vision, bringing an excerpt from the chapter of the Spring Report of the Portuguese Observatory of the health_system, 2022, on digital health. It states that digital health, by enabling the storage, analysis, and sharing of relevant information, promotes the establishment of a network of sharing and collaboration, fundamental in the implementation of care integration projects, contributing to the provision of care more centered on the user, addressing their specific needs, and improving the effectiveness, efficiency, and quality of care. Highlight noted by organizations of people living with disease: that the health information system be centered on and oriented toward responding to the concrete needs of people in each situation and moment. Need for redesign, improvement, and generalization of the use of the personalized_care_plan: The urgent need to develop and implement, in daily practice, the personalized_care_plan was identified. This is an indispensable component of the personal clinical process. It is naturally grounded and based on the electronic_health_record. It was mentioned to have been a project initiated years ago but interrupted. Its redesign and implementation are expected to be carried out, even if progressively, in a systematic and universal way. Each person must be able to have, know, and participate, with professional mediation, if necessary, in their personalized_care_plan. This must be the basic guide to promote, protect, and maintain their health. This includes correctly following treatments in progress. The personalized_care_plan is applicable to any person, with or without disease.

**** *id_52 *doc_2 *year_2014 *author_2 *area_1

At this level, it is important to highlight, as initiatives that allow the user to exercise their right to informed freedom of choice, the partnership established between the Central Administration of the health_system and the National Association of Family Health Units, for the purpose of making available and comparing the results achieved by the different primary healthcare provider units, the creation of the nhs Monitoring microsite, which has enabled the dissemination of information on the different dimensions of analysis of the health_system, access, efficiency, effectiveness, production, and satisfaction, and the health dashboards that provide information allowing monthly monitoring of the health status of the Portuguese population. The effort to produce information and knowledge about providers is therefore essential for the future development of a public healthcare market in which users are intended to be the central piece of the system as informed consumers.

**** *id_54 *doc_2 *year_2016 *author_2 *area_3

On the other hand, it has become essential to promote the use of good practices in the use of instruments and information systems that are important for access to healthcare, embodied in the creation of siga nhs, which in the coming years will make it possible to improve the integration and articulation of clinical management systems, citizen information, and overall institutional management. The effort to produce information and knowledge about providers is therefore essential so that users are truly central agents of the health_system in general and of the nhs in particular.

**** *id_55 *doc_2 *year_2017 *author_2 *area_3

nhs hospitals have responded positively to the steadily increasing demand, implementing various internal reorganization measures that make it possible to obtain better health outcomes for users and that promote high levels of effectiveness and efficiency in management and in clinical governance, with greater transparency, accountability, answerability, and centrality on the citizen and their family. In fact, during 2016 several reforms were initiated that focus on the internal organization of hospitals, notably the implementation of various accountability and evaluation mechanisms, the guarantee of improved clinical and management information, the deepening of partnership and complementarity relationships among the various nhs structures, the strengthening of coordination and articulation with other levels of care and other actors, in health and social sectors, among others. Integrated Continued Care is centered on the global recovery of the person, promoting their autonomy and functionality within the scope of their situation of dependency. The National Network for Integrated Continued Care, created by law number 101_2006, of 6 June, has as its main objectives the provision of continued and integrated healthcare and social support to people who, regardless of age, are in a situation of dependency and with loss of autonomy. This innovative model of care provision traverses the nhs and the institutional responses of the social sector transversally, based on network operation, where the different response typologies articulate between the hospital level and the primary care level, thus creating a network that interrelates the classic segmented organization that until the emergence of the National Network for Integrated Continued Care characterized the responses of the social and health sectors. With the National Network for Integrated Continued Care, people’s autonomy and functionality are promoted through rehabilitation, readaptation, and family and social reintegration, and therefore for each situation an integrated health and social action approach is recommended. The nhs_more_proximity pilot project began to be prepared in 2016 and started its practical implementation in the first months of 2017, with the main objectives of: Developing, testing, and evaluating a set of procedures and instruments necessary to initiate a qualitative transformation in the Portuguese health_system through a significant improvement in: The integration of healthcare; The management of people’s pathways in healthcare, both in relation to acute illness and with regard to people with multiple health problems, and investment in empowering the citizen in the promotion and protection of their health and in the proper use of health services. Still within the scope of this project, and in order to promote health literacy, the citizen area of the nhs portal already provides access to the health literacy library and to digital books. The citizen area also provides new personalized_care_plan that allow the citizen, together with their health team, to create a plan with well_defined goals, with the purpose of promoting health, preventing disease, and improving quality_of_life. In September 2016, the citizen area of the nhs portal was revised, improved, and expanded, allowing more intuitive navigation, a clearer allocation of electronic services by category, and the process of reviewing services in light of the concept of centralization on the citizen and on their life course was initiated, creating the component of Care Plans and Life Course, which will be expanded and embodied during 2017. The professional area is a user_centered platform that allows access by healthcare_professional, physicians and nurses, to their clinical information. The information that the user makes available on the health data platform in the citizen area, and whose consultation is authorized by them, allows the healthcare_professional to obtain some indicators that can help in better knowledge, diagnosis, and treatment of the user. Access to this professional area is made through the IT system used by the healthcare provider and is available in public and private institutions, providing access to information contained in local databases. This portal allows intercommunication between the information systems of each of the nhs health institutions, thus enabling the aggregation and visualization of the registered users’ health information whenever and wherever necessary. The Strategic Plan for the Development of Palliative Care for the 2017_2018 biennium recommends the development of a functional national palliative care network, fully integrated into the nhs and implemented at all levels of healthcare, allowing equity in access to quality palliative care, appropriate to the holistic, physical, psychological, social, and spiritual needs and preferences of patients and their families.

**** *id_56 *doc_2 *year_2018 *author_2 *area_3

nhs hospitals have responded positively to the steadily increasing demand, implementing various internal reorganization measures that make it possible to obtain better health outcomes for users and that promote high levels of effectiveness and efficiency in management and in clinical governance, with greater transparency, accountability, answerability, and centrality on the citizen and their family. The improvements in response were based on several reforms focusing on the internal organization of hospitals, notably the implementation of various accountability and evaluation mechanisms, the guarantee of improved clinical and management information, the deepening of partnership and complementarity relationships among the various nhs structures, the strengthening of coordination and articulation with other levels of care and other actors, in health and social sectors, among others. The 2017 hospital contracting process sought to contribute to promoting access to the nhs by introducing a set of guidelines and innovative measures to improve the care performance of nhs institutions, with emphasis on the following: expanding free access and circulation of the user within the nhs, diversifying alternatives and increasing their capacity to intervene proactively and responsibly in managing their health status and well_being; encouraging transparency and a culture of multidisciplinary and multiprofessional team healthcare, promoting effective articulation and coordination and a user_centered response; consolidating affiliation processes and collaborative network work within the nhs, centering the organization of care on the user’s needs and pathways and encouraging cooperation among institutions; In the course of 2016, a new approach to health literacy and care integration was conceptualized, and in 2017 the design of the organizational, informational, and communication instruments necessary for this purpose was begun, creating the conditions to make it possible to test a significant qualitative leap in the nhs: an nhs closer to people, better centered on them, more qualified, with greater problem_solving capacity. The nhs_more_proximity pilot project began its practical implementation in the first months of 2017, with the main objectives of: Developing, testing, and evaluating a set of procedures and instruments necessary to initiate a qualitative transformation in the Portuguese health_system through a significant improvement in: The integration of healthcare; The management of people’s pathways in healthcare, both in relation to acute illness and with regard to people with multiple health problems, and investment in empowering the citizen in the promotion and protection of their health and in the proper use of health services. Still within the scope of this project, and in order to promote health literacy, the citizen area of the nhs portal already provides access to the health literacy library and to digital books. This area also provides new personaized_care_plan that allow the citizen, together with their health team, to create a plan with well_defined goals, with the purpose of promoting health, preventing disease, preventing exacerbations of chronic disease, and improving quality_of_life. The Incentive Program for Care Integration has as its main objectives: Placing people, their families, and their caregivers at the center of interventions in the nhs; Strengthening prevention, early diagnosis, continuity, and proximity of nhs responses; raising levels of access, quality, and efficiency in the nhs; Promoting alignment, articulation, and coordination among entities, more partnerships with the community; Encouraging Clinical and Health Governance along the citizens’ life course; Valuing the engagement, initiative, and good performance of healthcare_professional. The citizen area of the nhs portal was improved and the provision of information to citizens increased, allowing more intuitive navigation, a clearer allocation of electronic services by category, and the process of reviewing services in light of the concept of centralization on the citizen and on their life course was initiated, creating the component of Care Plans and Life Course, which will be expanded and embodied during 2018. Guiding principles of nhs_24: nhs_24 is governed by principles and values directly related to care provision within the nhs, namely: Focus on the user, centered on the citizen’s needs, privileging personalized attention and relationship with the citizen; simplicity and accessibility; integration; simplification; universality and equity; confidentiality; proximity. nhs_24 has as its main objectives: Expanding and simplifying the population’s access to health information and services; Guiding the citizen to the health services most appropriate to their needs, contributing to reducing situations of congestion in health services, namely emergency rooms and administrative services, appointment scheduling; Promoting citizen engagement in active health management, responding clearly and in a timely manner to their needs; Collaborating to increase the effectiveness and operational efficiency of the health sector, promoting the necessary articulation among the various nhs entities and the integration of the Information Systems of the ministry_of_health; Contributing to an integrated view of the different health measures and programs. The implementation of siga nhs is underway, following the publication number 147_2017, of 27 April, constituting a system of monitoring, control, and provision of integrated information, intended to allow comprehensive and global knowledge of access to the healthcare delivery network within the nhs. This is an innovative approach to access to the nhs, centered on the citizen, which allows full monitoring of access to healthcare through the articulation of the various levels, services, and types of response, in a transversal and integrated way, enabling a complete view of the user’s path in the system, from the identification of a health problem to its resolution.

**** *id_57 *doc_2 *year_2019 *author_2 *area_3

nhs hospitals have responded positively to the steadily increasing demand, implementing various internal reorganization measures that make it possible to obtain better health outcomes for users and that promote high levels of effectiveness and efficiency in management and in clinical governance, with greater transparency, accountability, answerability, and centrality on the citizen and their family. The improvements in response were based on several reforms focusing on the internal organization of hospitals, notably the implementation of various accountability and evaluation mechanisms, the guarantee of improved clinical and management information, the deepening of partnership and complementarity relationships among the various structures of the nhs, the strengthening of coordination and articulation with other levels of care and other actors, in health and social sectors, among others. The Certification Model of the ministry_of_health is operationalized in 5 dimensions: the citizen at the center of the health_system; the organization of activity centered on the person; the healthcare_professional; the support processes; the results. The 2018 hospital contracting process sought to contribute to promoting access to the nhs by introducing a set of guidelines and innovative measures to improve the care performance of nhs institutions, with emphasis on the following: Expanding free access and circulation of the user within the nhs, diversifying alternatives and increasing their capacity to intervene proactively and responsibly in managing their health status and well_being; Encouraging a culture of care delivery by multidisciplinary and multiprofessional teams, promoting articulation and coordination among healthcare_professional and a response centered on the user and on their pathway within the nhs; Consolidating affiliation processes and collaborative network work within the nhs, centering the organization of care on people’s needs and promoting cooperation. From the perspective of health promotion, care integration, and citizen centrality in the health_system, the National Program for Health Literacy and Care Integration was created through law number 6429_2017, of 25 July, with the aim of promoting a closer nhs. In the course of 2016, a new approach to health literacy and care integration was conceptualized, and in 2017 the design of the organizational, informational, and communication instruments necessary for this purpose was begun, creating the conditions to make it possible to test a significant qualitative leap in the nhs: a closer nhs, more proximity, better centered on people, more qualified, with greater problem_solving capacity. Guiding principles of nhs_24: nhs_24 is governed by principles and values directly related to care provision within the nhs, namely: focus on the user, meeting the citizen’s needs, privileging personalized attention and relationship; simplicity and accessibility; integration; simplification; universality and equity; confidentiality; proximity.

**** *id_58 *doc_2 *year_2020 *author_2 *area_3

nhs hospitals have responded positively to the steadily increasing demand, implementing various internal reorganization measures that make it possible to obtain better health outcomes for users and that promote high levels of effectiveness and efficiency in management and in clinical governance, with greater transparency, accountability, answerability, and centrality on the citizen and their family. The improvements in response were based on several reforms focusing on the internal organization of hospitals, notably the implementation of various accountability and evaluation mechanisms, the guarantee of improved clinical and management information, the deepening of partnership and complementarity relationships among the various structures of the nhs, the strengthening of coordination and articulation with other levels of care and other actors, in health and social sectors, among others. The 2018 hospital contracting process sought to contribute to promoting access to the nhs by introducing a set of guidelines and innovative measures to improve the care performance of nhs institutions, with emphasis on the following: Expanding free access and circulation of the user within the nhs, diversifying alternatives and increasing their capacity to intervene proactively and responsibly in managing their health status and well_being; Encouraging a culture of care delivery by multidisciplinary and multiprofessional teams, promoting articulation and coordination among healthcare_professional and a response centered on the user and on their pathway within the nhs; Consolidating affiliation processes and collaborative network work within the nhs, centering the organization of care on people’s needs and promoting cooperation. Guiding principles of nhs_24: nhs_24 is governed by principles and values directly related to care provision within the nhs, namely: focus on the user, meeting the citizen’s needs, privileging personalized attention and relationship; simplicity and accessibility; integration; simplification; universality and equity; confidentiality; proximity.

**** *id_60 *doc_7 *year_2017 *author_2 *area_3

To improve the health of the Portuguese, it is necessary to mobilize local communities for the protection and promotion of their health, through widely participatory local health strategies. For this it is important to invest in each citizen’s capacity to make informed decisions about their health over their life course, health literacy. Better health literacy is a very important factor for good management of people’s pathways in the nhs and also for achieving better results in the provision of that care. This means betting on the centrality of people in the nhs of the future. More than 1 third of the Portuguese population has multiple health problems, often of prolonged evolution. Therefore, these people are also the most frequent users of health services. They will now benefit from a personalized_care_plan, which will facilitate the management of their pathways through the health services they need. Each person will be able to participate, with the healthcare_professional who provide care, in identifying their most important health problems, in defining the necessary care, and in periodically evaluating the results obtained. Thus nhs_more_proximity focuses on 2 fundamental ideas: the integration of healthcare and the centrality of the citizen in health_system. In dealing with the nhs, it was decided to address first the integration of healthcare and then to consider it in the broader context of health protection and promotion and disease prevention. With this we seek, in some way, to signal and minimize the inconvenient separation between healthcare and public_health, with the relative isolation of the latter. But it is mainly about looking at pathways in healthcare as occurrences inserted in a life course, with past, present, and future, in all its dimensions and well_being potentialities. The integration of healthcare has multiple facets. Here priority is given to what is called the management of people’s pathways in the nhs. This means looking at access to quality healthcare not as a one_off issue, but rather from a perspective of continuity, making people move from one service to another when they need it, without unnecessary barriers or discontinuities, always keeping in view the results intended to be achieved. This care integration requires an effective approach, exchange, and communication among the various organizations that provide healthcare within the nhs. And also outside this scope, as is the case, namely, of social services. The idea of the centrality of the citizen in the health_system necessarily involves empowering people to make smarter decisions regarding their health and health services. Without this, health_system will hardly achieve the desired performance. Finally, the idea of citizen centrality must be reflected in the way people are attended to in the nhs. It is indeed necessary to make a new effort to qualify the service spaces in the nhs, in their physical, organizational, relational, and informational dimensions. These are necessary and ambitious changes. They will not be made by decree. They imply managing a demanding change process. For this, new management, information, and communication instruments are needed, already in preparation for about a year now. The main devices of this change process need to be tested locally and this process is not viable without the interest and mobilization of all local leaderships. They require some financial investment and will have to take into account the country’s regional and local asymmetries. None of the components of nhs_more_proximity is entirely new, they are inserted in previous experiences that now expand, are leveraged, and above all, are better instrumented and integrated. The development of nhs_more_proximity is conditioned by the evolution of the sectoral reforms underway, primary healthcare, continued care, hospital care, and public_health, and by the progress made in the management of health resources, human, financial, technical, and technological. On the other hand, nhs_more_proximity serves as an indispensable focal point for these multiple sectoral initiatives, ensuring that they converge to allow a more effective response to what people expect from their nhs. Only the nhs in fact has the capacity to achieve a high level of care integration while also articulating it with actions aimed at protecting and promoting the health of the Portuguese. The success of the management of people’s pathways in healthcare depends, to a large extent, on the adoption of a set of instruments and procedures: Development of a model of personalized_care_plan, improvement of the conditions for conversation and negotiation necessary for the adoption of a personalized_care_plan, progress in the classification of patients with comorbidities; Updating of collaborative protocols among all entities and services that participate in the execution of care processes identified in the personalized_care_plan; Contribution of siga: integrated management system of access to healthcare; Importance of people’s behaviors in the success of their personalized_care_plan, articulating this process with that of promoting health literacy; Evaluation of the results of pathway management, with the participation of all entities involved. The personalized_care_plan allows records related to health situations considered priorities for a time horizon agreed between the person and the healthcare_professional who provide healthcare. For each of these situations, the personalized_care_plan records the current situation, the objectives to be achieved, and the actions and behaviors necessary for that purpose. The personalized_care_plan also monitors the planned actions and the periodic evaluation of results, for each person and for the set of situations followed. Thus, the personalized_care_plan reflects the sharing of work and responsibility between healthcare_professional and the person themselves to achieve the intended health results. The development of the personalized_care_plan will include the following aspects: Easy access from the personalized_care_plan to information relevant to the management of each of the situations identified as the object of monitoring; Alert regarding the dates for executing the actions planned in the care plan; summary of the different services used or planned in the personalized_care_plan; Full integration of the personalized_care_plan into the personalized health information system, including the health data platform. Good management of the personalized_care_plan and its results depend largely on the quality of interaction, conversation, and negotiation between people and the healthcare_professional who provide them with healthcare. To ensure this quality it is often necessary to improve critical aspects of the organization of clinical practice in primary healthcare, such as the availability to promote people’s narrative about their health situation, the relationship between conversation time and time for recording and IT management, and the human and material resources needed for this purpose. The evaluation of the management of people’s pathways in healthcare will take place at 3 levels: Degree of achievement of the objectives established in the personalized_care_plan: this evaluation is the responsibility of the team that manages the respective personalized_care_plan, namely the person themselves and the healthcare_professional who initiated it, usually the primary healthcare team; Results of the personalized_care_plan in progress in each Primary Health Care Clusters: this evaluation is the responsibility of the clinical and health council of the primary health care clusters; Set of objectives of nhs_more_proximity, the responsibility of the ministry_of_health. Care at home relocates care from institutions to people’s homes. This requires a redefinition of the care process, which must take into account the following aspects, which, while not exclusive to home care, are particularly relevant here: People’s homes as the privileged place for providing care and the patient and their caregiver as the focus of care; Various aspects associated with the care process, highlighting housing and community conditions, affective networks, socioeconomic conditions, among others; co_production of care, meaning the involvement of all actors in the care process; Integration of care, not only among different types of care, but also with social security and other actors in the community, in which coordination has a particularly relevant role; Pro_activity and continuity of care through the personalized_care_plan, as the privileged instrument and expression of the centrality of care, of the involvement of the patient and family, and of communication among all caregivers. Health information cannot continue to be fragmented according to the various modalities of healthcare that reach people’s homes. The personalized_care_plan will become the common record for all these modalities of healthcare provision and, for this purpose, its development during 2018 should allow it to be configured accordingly. When emphasis is placed on the centrality of the citizen in the health_system, this means that, beyond being the object of the attention of health services, they must also become the subject, the main actor, in the health_system. Citizen centrality also means their involvement and participation in what concerns the protection and promotion of their health and that of their fellow citizens. Here too, beyond the goodness of the principle, it is necessary to instrument its application. Not always in nhs units is there concern to provide the best possible service to the people who seek them. There is no citizen centrality without ensuring that they are attended to in the nhs, in all circumstances, in the best possible way—quality of facilities, of relationships in service, of information in service and waiting spaces. In this context nhs_more_proximity, in this first phase of its development, essentially privileges 3 aspects associated with the centrality of the citizen in the Portuguese health_system: a new generation of local health strategies; the promotion of health literacy; and the qualification of service in the nhs. The first 2 are related to Portuguese public_health. There is in Portugal a vast set of health programs, framed by a national_health_plan. It is necessary to deepen their implementation through local health plans. The promotion of health literacy is indispensable for this purpose. It is important to note that the issue of citizen centrality, particularly in its public_health dimension, lies beyond the scope of the nhs. In this first phase of development of nhs_more_proximity, the populations covered by the pilot projects will benefit from a new generation of local health strategies. These, taking into account the experience already had with local health plans, pay special attention to a set of particularly significant aspects, currently, for the protection and promotion of health: Addressing health over the course of people’s life pathways; Including local governance as a critical factor for the development of local health strategies, paying greater attention to inter_sectorality, achieving better integration into the dynamics of local institutions, mainly in what concerns well_being, facilitating and promoting people’s participation; Ensuring, from the outset, that the expected results can be evaluated in the short and medium term. The capacity of citizens to make informed decisions about their health and about the appropriate use of health services is a critical factor for health promotion and for good management of pathways in healthcare. Hence the role that health literacy plays in the integration of healthcare, but also in health protection and promotion throughout the life course. Beyond the issuance of health information, it is necessary to equip the receiver, people, to incorporate and personalize this information. my_health_diary constitutes this personalization tool, which helps activate the citizen to take an interest in their health and in the proper use of healthcare. It allows them to import content directly from thematic digital books, interact with multiple sources of information, including the nhs portal and the messages it will start to issue, organizing for their own use, according to their thematic preferences and the type of use they wish to make of my_health_diary. The current version of my_health_diary should be seen as the first step in a development process that aims to support each person in finding the best solution to organize and manage their health information. Several aspects deserve special attention in managing the change needed to implement nhs_more_proximity: Mobilizing local leaderships for innovation, developing new management, information, and communication devices and instruments; Promoting consultative and participatory processes, involving social health actors and organizations representing citizens; Aligning the management of human and financial resources with the objectives of nhs_more_proximity; Carrying out the digital transformation of the nhs as one of the main vehicles for implementing nhs_more_proximity; Moving from a limited number of regional experiences, necessary to test and evaluate many of the new instruments indispensable in this type of change, to the whole country; Learning from experience, monitoring and evaluating processes and results, while promoting stimulating articulation with similar international initiatives. nhs_more_proximity will make its healthcare_professional feel that it values people, that their working conditions and professional satisfaction matter. But it is equally necessary for healthcare_professional to deepen ways of acting and collaborating among themselves, centered on the contribution of each professional profile to achieve better health results in people. It is indispensable to interest and adequately involve healthcare_professional in the development of nhs_more_proximity. This implies, among other things: Providing accurate information on the objectives and actions planned in the development of the proposed projects; Conducting consultations on aspects of service functioning that can be improved in the short and medium term and that are of critical importance for the good development of nhs_more_proximity; Promoting preparatory meetings with the healthcare_professional involved before the start of the implementation of new projects; Establishing a green communication line between healthcare_professional, leaders, and the management of each project; Not neglecting aspects related to the demands of care integration and citizen centrality in professional training actions. The digital transformation of the nhs is underway. In this transformation there are essentially 2 aspects to consider: the first has to do with the digitization of health information; the second concerns the profound change in the processes of working relationships in the nhs. This transformation allows deepening and accelerating the continuous, expeditious, and secure collection of information relevant to health and making this information available to people and healthcare_professional so that they can make intelligent and timely decisions in their everyday lives. In this way the digital transformation should constitute one of the main integrating elements of nhs_more_proximity, overcoming the multiplication of fragmentary IT applications that do not communicate with each other and hinder the full enjoyment by healthcare_professional and citizens of the most desired benefits of the digital transformation. These projects aim to technically support the development of nhs_more_proximity in the period 2017_2018. personalized_care_plan: Information management device with an essential role in managing people’s pathways in healthcare. Started in the second half of 2016. A third version expected in January 2018. Classification of patients: Important for care integration and for the personalized_care_plan; each family of healthcare has its own classification. Conversation and negotiation as a starting point for the personalized_care_plan. Various aspects of the routine primary care consultation tend to limit the full development of the type of conversation and negotiation necessary for the personalized_care_plan. Caring at home: Integrate all aspects of home healthcare and ensure its effective coordination. Local health strategies: It is indispensable to establish a set of updated guidelines and develop management, information, and communication instruments that facilitate the elaboration, monitoring, and evaluation of local health strategies. Digital library, including thematic digital books: The library, with regard to the IT platform, will be stabilized at the beginning of 2018. By the end of 2018 it will have a collection of about 20 thematic digital books. my_health_diary: This agenda is a central element in the health literacy promotion strategy: started in 2017, it needs to be better integrated into the citizen area and to communicate better with the other devices for personalizing health information. Activation trials with different mediators, nhs, pharmacies, libraries, and schools, are needed in 2017 and 2018. Qualification of nhs service spaces: Started in 2016, but with little progress in 2017. Needs a new start in 2017. Requires better recognition of existing good practices, institutionalization of those responsible for service quality in all nhs units, new information and communication instruments, and a support module, valuing and empowering those who, at health unit receptions, attend to the public. Integration of personalized health information instruments, including the personalized_care_plan and my_health_diary. It is important for the coherence of the health information system and for those who use it, healthcare_professional and citizen, to start this integrative project with scheduled goals for 2017 and 2018. Evaluation: This project is intended to design and implement an evaluation process for nhs_more_proximity, which includes the evaluation of both processes and results. Started in 2016_2017, to continue in 2018_2019.

**** *id_61 *doc_5 *year_2023 *author_3 *area_1

Palliative care, as holistic care, play a vital role in the course of chronic and life_threatening illnesses of many Portuguese people, improving quality_of_life for these people, but also for their caregivers, family members, and friends who support them. They are active care, applicable at any age, in different diseases, and must be available at all levels of healthcare. Therefore, in the Portuguese context, and in alignment with the oms, palliative care are defined as follows: Palliative care are a crucial part of integrated and person_centered health services. Relieving suffering, whether physical, psychological, social, or spiritual, is a global ethical responsibility. Thus, whether the cause of suffering is cardiovascular disease, cancer, severe organ failure, drug_resistant tuberculosis, severe burns, terminal_phase chronic diseases, acute trauma, extreme prematurity at birth, or the extreme frailty of old age, palliative care may be necessary and must be available at all levels of care. Vision: All people with life_limiting illness receive the care they need, at the moment they need it, to live with better quality_of_life. Guiding Principles: Palliative care are care centered on the person, their family, and caregivers and are based on effective communication, shared decision_making, personal autonomy and extend into the bereavement process; Palliative care must be available and provided to all people living with an active, advanced, and progressive disease, regardless of diagnosis; Palliative care affirm life while recognizing that dying is an inevitable part of life; Family members and caregivers are valued and receive care appropriate to their needs; Care are specialized and based on scientific evidence. People with complex health needs require care from multiple providers in various healthcare settings, which means the risk of receiving fragmented care is high. In the current process of reorganization of the nhs, it is essential to aggregate the Palliative Care Teams into the Integrated Palliative Care Service of the Local Health Units, ensuring the provision of care in an integrated manner to the person with palliative needs and their family, centered on their needs, with inclusion of them in decision_making, aiming at the simplification of processes, the qualification of responses, and improvement of outcomes. Palliative care, as coordinated and personalized care, recognize people’s individual needs, enabling them to develop the knowledge, skills, and confidence they need to make informed decisions and more competently manage their health project. Thus, there are 4 principles for the development of person_centered care: providing people with dignity, compassion, and respect; offering coordinated care, support, and treatment; offering personalized care, support, and treatment; supporting people in recognizing and developing their own strengths and competencies to enable them to live an independent and full life. In this way, incorporating the experience of the sick person, the family, and the caregiver is an integral and essential part of the provision of high_quality care and, as such, the strategy of this Strategic Plan for the Development of Palliative Care is that Palliative Care must be made universally available in the nhs as a right inherent to the human condition, closely linked to the Right to Health enshrined in the Universal Declaration of Human Rights. The role played by family members and caregivers of the person with palliative needs is indispensable, irreplaceable, and complementary to the care provided by local palliative care teams. With the purpose of empowerment, so that these family members and caregivers assume a higher level of awareness about: the dimension of the disease; care goals; principles and philosophy of palliative care and about the available resources, it is necessary that their psychological, emotional, and spiritual needs are also addressed. Priority Axis Person_Centered Care: Preparation Referral Criteria for Specialized Adult Palliative Care Teams; Preparation Referral Criteria for Specialized Pediatric Palliative Care Teams; Pediatric Care Plan Proposal of a model care plan for use by teams; Adult Care Plan Integration in the working group of the Central administration of the health_system on Integrated care for complex chronic patients: Personalized Care Action Plans.

**** *id_62 *doc_4 *year_2013 *author_1 *area_1

Hospital discharge occurs at the moment when the puerperal woman leaves the hospital for home or the place where she has planned her post_partum. Most authors consulted define early discharge when it occurs before 48 hours post_partum. For this to occur, the hospital and community resources must develop a robust support system that ensures that the transfer of women and their babies to home or to the place where they will be supported is centered on the woman or couple and on their needs, contributing to a smooth transition to the exercise of parenthood. Improve the articulation networks between the different levels of care that allow encouraging and supporting the transfer of care of the person with chronic disease from the hospital to the community. Implementation of a model of care provision centered on the citizen, with levels of supply adjusted to actual health needs. The compartmentalization of health units, which prevents the patient from moving nimbly through the nhs, must be combated. The person must be able to move nimbly between primary healthcare, entry into the system, and secondary and tertiary care units, having a professional who accompanies them along the pathway, the nurse who accompanies them in primary healthcare or the family nurse, when created, is suggested.

**** *id_67 *doc_2 *year_2021 *author_3 *area_3

The purpose of this text is to contribute to anticipating an nhs attentive to current and future challenges, centered on the citizen, on their life and health pathways, on communities, on public_health. The main weaknesses lie in the dispersion, fragmentation, and disconnection of many of the planned investments. The achievement of the necessary and desired changes certainly depends on the prevailing culture and on management and institutional governance competencies. The expression person_centered integrated care has been widely used, although with weak systemic implementation. However, at the level of some local projects already underway and of the action of health teams in caring for concrete people, it is possible to identify processes, procedures, and practices aligned with and illustrative of that concept. The person, the patient, the citizen is an infinitely heterogeneous entity. This immense diversity and heterogeneity concern: life stage, biological and functional circumstances, psychosocial, family, socioeconomic, socio_occupational, sociocultural, existential, and health and well_being condition. The experiences lived in services and units with dedicatedly proactive multiprofessional teams show complex and variable attitudes and practices to mobilize the means and care needed for each patient at a given moment and situation, with collaboration and handovers in the transition between teams, services, and units. The essential problem is that they face barriers and obstacles to the proper fulfillment of their mission. The successful cases, where it was possible to move mountains, illustrate that the case management function is, naturally, mobile, dynamic, flexible, and deeply embedded in the core competencies of each professional and team. It emerges from the technical, scientific, and organizational quality of each team. It is not susceptible to being rigidly fixed in one professional. It is up to decision_makers and systemic organizers to make the lives of healthcare_professional and teams easier, rather than hindering their action and limiting their effectiveness. The single personal electronic clinical process, centered on the citizen and controlled by them, is a key_element for the management of health pathways in an integrated and coordinated manner. This concept goes beyond the current conception of electronic_health_record, which functions as a repository of clinical information, from various sources, fragmented, without accessibility to the citizen. An essential component of the single personal electronic clinical process is the electronic clinical summary. In turn this Summary will contain the integrated_care_process or personalized_care_plan. The new process should be accessible to the citizen themselves, and at all points of the delivery network, always with their authorization. The participation of populations and of the various citizens’ organizations is important to identify needs and potential responses, but it is also fundamental to recognize that the engine of transformation lies in healthcare_professional and in their determination to implement best practices in the care of people and families and to increase collective health.

**** *id_68 *doc_2 *year_2022 *author_3 *area_1

The idea of people_centered health is little more than a slogan without anchoring in reality or else a goal, perhaps sincere, that remains distant. Thus, a model of care is advocated that simultaneously responds to 3 fundamental principles: guarantee person_centered care, integration, and continuity of care. In other words, domiciliation of care can simultaneously be a way to guarantee ageing at home, high levels of satisfaction with care, and cost reduction. We summarize some of the assumptions of this redefinition: assume the patient and their caregiver as the focus of care; assume the informal caregiver also as a care partner; assume the co_production of care as a guarantee of the involvement of the various actors in the care process; assume the personalized_care_plan as the privileged instrument and expression of the centrality of care, of the involvement of the patient and family, and of communication among all caregivers; ensure care integration; ensure permanence and continuity of care and perform proactive management of situations through the adoption of the figure of a care coordinator; use technological resources and telecare, duly integrated into the care model. All this integrated into a network of responses that guarantees equity in access. As already mentioned, domiciliation of care, caring at home, has the potential to increase satisfaction with care, promote ageing in a family context and, thereby, contribute to better indicators of quality_of_life and health. However, for this we need to have not only a strategic political vision that defines and regulates the process from multiple perspectives, but also a model of care that ensures that care is centered on the person and their family, is integrated and continuous. The increasing application of digital technologies in the health sector has allowed the implementation of care models that are more centered on the user, that promote care integration, examples of which are self_management programs for the health_disease process and home care, whose benefits at the level of the user experience, cost reduction, and maintenance of quality and safety have been widely recognized. Client centrality: monitoring user experience and forecasting their needs, in the health area this point can be promoted through the use of user_reported outcome and experience measures, as well as their involvement in the design of digital technologies. One of the care models that has emerged in order to provide care more centered on the user, with cost_effective benefit, is hospitalization at home. The basic principle of hospitalization at home is the treatment of users with disease in an acute phase or exacerbated chronic phase who require hospital_level care, but who meet the conditions to be treated at home. Digital health, by allowing the storage, analysis, and sharing of relevant information, promotes the establishment of a network of sharing and collaboration, fundamental in the implementation of care integration projects, contributing to the provision of care more centered on the user, addressing their specific needs, and to the improvement of the effectiveness, efficiency, and quality of care. Once again we affirm that it does not make sense to only increase the number of responses without first defining a strategy built on the principles already mentioned: care centered on the person and family, integrated and guaranteeing continuity. For this reason, we begin with a brief conceptual framework, which addresses the need to define a model of home care. Given its structural character and, at the same time, its promise for the future, we dedicate a chapter to technological and therapeutic innovation. In fact, innovation, broadly, and digitalization in particular are systematically presented as an unquestionable contribution to increasing the response capacity of the nhs to the challenges it faces. Because, as is stated, digital health, by allowing the storage, analysis, and sharing of relevant information, promotes the establishment of a network of sharing and collaboration, fundamental in the implementation of care integration projects, contributing to the provision of care more centered on the citizen, addressing their specific needs, and to the improvement of the effectiveness, efficiency, and quality of care. However, while this is true, we also need to appeal to history and recall what went less well with the first wave of health digitalization and what still persists. We refer to a health information system with proliferation of applications and evident gaps in interoperability between levels of care and which, in truth, contributes little to the integration and continuity of care.

**** *id_69 *doc_2 *year_2020 *author_3 *area_1

A cultural paradigm shift is necessary: all stakeholders in the health_system must recognize the important role of the citizen as a primary stakeholder, but also as a partner, who can contribute, with their knowledge and evidence from life experience, at all levels of the system. Promote contexts where citizen involvement and participation can be facilitated, developing a truly person and community_centered culture. This implies creating participatory mechanisms and spaces, responsive to citizens’ needs. Institute greater transparency at all levels of the health_system, providing citizens with access to clear information and to their own health data. Encourage the empowerment of all stakeholders in participatory processes in health: citizens and their representatives, including their families, healthcare_professional, decision_makers and rulers. Develop mechanisms that encourage the application of existing legal frameworks, namely considering the existence of incentives. Legal frameworks are important, but their implementation has been insufficient, as can be observed from the still weak functioning of the Community Councils of the Primary Health Care Clusters and the Advisory Councils of hospitals, or from the absence of regulation of the Charter of Public Participation in Health. Consider the existence of specific and sustained funding for the promotion of public participation in health, implementing the necessary and appropriate mechanisms and channels, and also ensuring the independence of the citizens’ representative organizations that participate. Evaluate participatory processes, to obtain evidence on best practices in this area, which may, in turn, contribute to the paradigm shift that is intended to be promoted. The promotion of a culture and literacy in public participation implies that, on the one hand, citizens understand the importance of their role in participatory processes and, on the other, that the different levels of decision and governance, where this participation should be encouraged, are sensitized and empowered to promote and manage these processes. The who has been recognizing the fundamental role of citizens in health planning and decision_making processes, by contributing their daily life experience, with or without the existence of disease, which constitutes additional evidence to technical and scientific data. All actors in the health_system must understand and put into practice this recognition that the citizen holds important and valid knowledge, which must be shared with healthcare_professional, technicians and decision_makers as part of the co_construction processes of health. From the point of view of organizing participatory processes, it is important to take into account that, in most situations, citizens may not participate individually, but through formal organizations that represent them, such as various civil society organizations, patient associations, organizations representing users or consumers. It is necessary to promote contexts where this involvement is facilitated, developing a truly person_centered culture. This implies creating participatory mechanisms and spaces, responsive to citizens’ needs. At the organizational level, this partnership with users, families and communities in the design of processes, services and policies should guide the structuring of institutions and of the health_system, so that they better reflect their expectations and needs and promote specific opportunities for their involvement, guaranteeing more health gains and enabling better experiences for all stakeholders. Legislative and regulatory changes can increase the involvement of citizens and communities, improving institutional mechanisms to increase citizen and community participation. However, establishing legal frameworks is insufficient to ensure public participation, particularly if the necessary conditions for its existence are not provided, namely the empowerment of the actors involved in participatory processes and the existence of specific funding for participation. The development and alignment of policies and incentives may encourage citizens, families, healthcare_professional and organizations and the health_system to facilitate and promote participation in health. Portugal has a fairly robust legislative framework in this area, since participation and participation in health are provided for at different levels of legislation: Constitution, Law on Public Participation in Health. However, although the legal foundations exist, implementation continues to be insufficient, as in the case of the Community Councils of the Primary Health Care Clusters and the Advisory Councils of hospitals, which do not function effectively in most situations. Additionally, the absence of regulation of the Charter of Public Participation in Health prevents its practical and effective application. For there to be effective citizen participation and the real translation of their expectations and needs in participatory processes, it is necessary to ensure that those who participate, namely civil society organizations, do so in a context of independence. As such, it is desirable that specific financing mechanisms be established, appropriate to participatory processes, ensuring that the participation of these organizations does not depend on other funding sources that may determine the existence of conflicts of interest.

**** *id_70 *doc_4 *year_2018 *author_2 *area_1

The integrated_care_process are organizational processes that place the citizen, with their needs and expectations, at the center of the system. Care continuity and coordination between different levels of care are recognized as essential elements to ensure that the patient receives, in a timely and effective manner, the best healthcare. The approach of the integrated_care_process is a multidisciplinary, comprehensive and integrated approach that presupposes the reanalysis of all actions to which the patient is subject at any point in the health_system, from the beginning to the end of the integrated_care_process. On the other hand, care activities based on the best available scientific evidence respect the principle of rational use of health technologies and guide the adoption of cost_effective therapeutic actions, while ensuring the citizen the clinical quality that is enshrined as one of their main rights. It is intended to provide organizational change, based on the involvement of all healthcare_professional involved in care delivery, whether at hospital level or at the level of primary or continuing care, believing in their capacity and willingness to continuously improve quality and focus their efforts on people. The integrated_care_process are also a tool that allows analyzing the different components that intervene in the provision of healthcare and organizing the different workflows, integrating up_to_date knowledge, standardizing actions and placing emphasis on results, in order to respond to the expectations of both citizens and healthcare_professional. Clinical practice needs to be oriented toward results and quality, also aiming at more effective use of resources. The management of clinical practice, as defined in the integrated_care_process, calls for better organization of the care provided to people by the health_system, centering them on the citizen and, in this particular case, on the needs and expectations of the person with asthma and on the anticipation and comprehensive planning of care continuity.

**** *id_81 *doc_5 *year_2024 *author_2 *area_3

The organization and functioning of the healthcare provided by the nhs have been adding to the cumulative improvement of technical and scientific quality the concern with the centrality of the person, whether nhs users or its healthcare_professional, and with the spaces intended for the provision of healthcare. This concern imposes empowerment for the exercise of ethical, deontological, technical and scientific practices, for a consequent improvement in the quality, effectiveness and efficiency of the nhs. And it requires the development and deepening of the humanization of healthcare in the nhs. The concern with strengthening the humanization of healthcare is the result of greater demands by citizens in general and users, but also by healthcare_professional when they value the human dimension present in the provision of healthcare and the value of this dimension for people’s well_being. One of the nhs responses to the concern with the centrality of the person and the consequent development of humanization took place in 2019, with the drafting of the Commitment to Hospital Humanization, by a Working Group created by the Coordination for the Reform of the nhs in the Area of Hospital Healthcare. The commitment was approved by the ministry_of_health and signed by all Portuguese hospitals and hospital centers, including private hospital units. By force of law number 102_2023 of November 7, and under the terms provided for in the Statute of nhs, approved by law number 52_2022 of August 4, the organizational and operational model in local health units was adopted, to integrate hospitals, hospital centers and primary health care clusters. By this law, an integrated management model for the provision of healthcare to nhs users was also approved, incorporating, in the same public corporate entity, two legal and organizational dimensions, hospital healthcare and primary healthcare. This change aims at the integrated provision of primary and hospital healthcare, the strengthening of primary care in the response of proximity and continuity in health assistance and the commitment to health promotion. This new organizational, managerial and functional reality has as its final desideratum to contribute, in a more effective way, to the organization of health responses according to people, that is, the centrality of the person as a determining aspect in defining the organization and functioning of the nhs. To fulfill its mission, the nhs National Commission for the Humanization of Health Care will develop its action, choosing as essential pillars: respect for the dignity of the person; recognition of the individuality, humanity and uniqueness of each user and each professional, with correlative respect for autonomy, privacy, beliefs, values, feelings, emotional states and personal circumstances; recognition of the centrality of the person and of the sick person in all actions within the scope of health; the vulnerability of the sick person, in terms of emotional and or physical balance; the relevance of empathy, active listening and compassion in the relationship of healthcare_professional with users and among themselves; the relevance of the adequacy and conditions of physical spaces to the typology of the healthcare provided; the relevance of the availability of human and material resources in number and quality adjusted to needs. For defining the major objectives of the action to be developed by the nhs National Commission for the Humanization of Health Care, the action plan will take into consideration the attributions assigned to it by virtue of the deliberation that created it: Promote and energize reflection on the quality and efficiency gains that arise from the provision of person_centered healthcare and on the respective health pathway and episodes of disease in the nhs; Promote and energize reflection on the centrality of healthcare_professional in the processes of change leading to an organizational culture centered on humanized clinical practices and interpersonal and interprofessional relationships; Foster actions that stimulate and consolidate good interpersonal and interprofessional relationships in workplaces, and the commitment to constant improvement of processes and health outcomes; Identify good practices of healthcare humanization already underway in the Country and promote actions aimed at expanding their knowledge and dissemination; Survey needs, establish priorities and define and support improvement interventions appropriate to each situation, in close interaction with the heads of institutions and with respect for the autonomy that is theirs; Promote training actions in the field of humanization in health and provide contact with experiences and results achieved in other countries; Foster the creation of communication channels between nhs institutions dedicated to the dissemination and sharing of initiatives and programs within the scope of the humanization of healthcare; Create and keep active a Forum for Reflection on humanization in health; Support local health units in initiatives aimed at developing and deepening the humanization of healthcare; Foster and support initiatives that bring together the participation of various institutions and levels of care provision; Carry out periodic evaluations of the progress recorded in the field of humanization in health. This action plan has as its major objective to define the paths and activities leading to the continuous improvement of the humanization of healthcare provided in nhs health services. As an action plan designed to promote humanization within the scope of healthcare provided to nhs users, by healthcare_professional, it values: the centrality of the person in their trajectory as a user of nhs health services, that is, their perceptions, experiences, needs and expectations; healthcare_professional as agents of a provision of humanized healthcare and the care that should be directed to them; respect and cordiality in human relationships between users, their family members or companions and the healthcare_professional who work in health institutions; respect and cordiality in the relationship between all healthcare_professional who work in health institutions; the place where the relationship occurs: the organizational culture; the managerial vision and governance; information and communication; infrastructures and their adequacy. The objectives intended with the activity of the commission must aim at results that are anticipated as generally accepted by the parties. Assess the response capacity of the nhs National Commission for the Humanization of Health Care, delimit its activity and create conditions that enhance its action; Promote a culture of humanization of healthcare in the nhs: the humanization of healthcare provided by nhs institutions as an organizational value; the adoption by management bodies of commitments aimed at user and family satisfaction; the accompaniment of the user by a family member or companion designated by them given its added_value for diagnosis, for therapy and in terms of support and comfort; Survey humanization activities already implemented in institutions and needs to be filled; Know and characterize good humanization practices already underway and proceed to their dissemination; Create a team with specific training to process the data obtained with the surveys applied to learn about the reality, such as good practices and needs; Map institutional and non_institutional structures associated with humanization dynamics of healthcare, which may synergistically contribute to the implementation of humanizing procedures: Humanization Services, Humanization Committees, Citizens’ Offices, Friends’ Leagues, Volunteer Groups, Patient Associations; Foster an institutional identity in line with the humanization of care: Encourage the drafting of a Humanization Charter of the health institution, to be approved by the respective Board of Directors, binding on all health agents in the institution; Foster articulation with the patient and user ombudsman. in health services where it exists, publicize its existence and promote synergies arising from respective activities; Raise awareness for the implementation of tools and local changes that facilitate and promote the operational development of humanization: Conduct satisfaction surveys, with tested models, aimed at users and patients and family members during their pathways in nhs services, as well as for healthcare_professional; Improved communication in the nhs, more humanized and effective, through active listening to users, stimulating conversation with the user and listening to and valuing what is said; Creation of communication channels for users of each health institution, accessible and agile, based on new information and communication technologies; Integration of a users’ representative in the Humanization Committee of health institutions; Promote training actions within the scope of humanization of healthcare: Foster the training of healthcare_professional aimed at improving competencies that promote the centrality of the person; Raise awareness among healthcare_professional about the rights and duties of nhs users; Foster the creation or mobilization of theater teams for the performance of existing or written plays that express concrete situations of lack of humanization and respective correction. Promote and support innovative initiatives and projects of institutional intervention and with populations: Foster relevant changes for humanization, at the level of infrastructures dedicated to the provision of healthcare, organization of provision, material and human means, and work environment; Promote access to measures for prevention and treatment of burnout by healthcare_professional; Implementation of a culture of continuous improvement focused on improving access and on the involvement of users; Improvement of the pathway and experience of patients and users and guarantee that it is similar in different health institutions; Promotion of accompaniment of health service users by a family member or person indicated by them. Organize national_level meetings: For sharing practices underway in health institutions and difficulties encountered; Reflection and joint search for solutions; Contact with invited personalities, national or international, with recognized work in the area of humanization of healthcare; Create and keep active a permanent forum for reflection on humanization in health, motivating humanization committees and citizen offices to join; Create an annual award for the best humanization practice, with clear rules and dissemination of good practice by the remaining local health units; Promote the quality of information made available to users and family members, as a component of organizational culture and a way for their active participation in the life of health institutions; Define, draft and make available support content for improvement interventions within the scope of the humanization of healthcare, and support the production of action standards by institutions; Participate in international initiatives that allow knowing and implementing innovative practices and new models of approach to humanization processes. Personalization of caring and treating: the healthcare_professional values and practices empathy and active listening, and treats the other as an equal in dignity; the patient is addressed by the name they desire, with friendliness, courtesy and loyalty, as a right and duty of all; each patient is provided with the name of a professional, a primordial condition for the creation of a privileged and inviolable bond of dialogue and trust; patients and or their caregivers are enabled to follow the progression of the disease through the information given by healthcare_professional, in a sensitive way and adjusted to the level of understanding and psychological moment, and users must be empowered to make informed decisions; conditions exist to provide home care and post_discharge hospital care and, when indicated, committed and robust support is given in access to continuing care; in the daily exercise of a hospital or a primary healthcare unit the dictates of the Charter of Patients’ Rights and Duties and Charter of the Rights of Hospitalized Children are considered. Positive psychology, health and well_being promotion: creates conditions for the application of positive psychology techniques in human resources management, stimulus to collaborative work, motivation, reward, and promotion of health and well_being of human resources in the work context. Norms of interpersonal and interprofessional relationship: has behavioral norms for the relationship between healthcare_professional, based on each one’s respect for themselves and for the other, on the sharing of information relevant to the provision of multiprofessional care, on the duties of citizenship, on professional deontology and on the rules that arise from labor relations. Professional training: healthcare_professional oblige themselves, because it is indispensable, to create availability for training; ensures and creates, on the basis of a considered management of human resources, opportunities for lifelong professional training deemed necessary for the improvement of professional practice and its humanization; specifically, ensures and creates training in communication, empathy and interpersonal relationship. Satisfaction assessment: regularly monitors the satisfaction of human resources and the ease or difficulty of communication of users with healthcare_professional and the hospital or hospital center and uses the results obtained. Citizen participation: forms of listening to users are instituted, in addition to access to the yellow book, through regular satisfaction surveys of patients and users and the promotion of reflection groups made up of members of the surrounding community, who should be heard in assisting decision_making by Boards of Directors.

**** *id_84 *doc_5 *year_2013 *author_2 *area_3

The citizen is the center of the health_system. Strategies to strengthen citizenship in health are based on: Strengthening the citizen’s power and responsibility to contribute to improving individual and collective health. Promoting a continuous development dynamic that integrates the production and sharing of information and knowledge, health literacy. A culture of pro_activity, commitment and self_control by the citizen, empowerment, active participation, for maximum individual and collective responsibility and autonomy, empowerment. At the political level, one should: Promote a culture of citizenship, based on the development of initiatives aimed at the community or population groups, aiming at promoting literacy, empowerment, empowerment and participation, having as axes the dissemination of information, the development of skills, in individual, institutional and political decision_making, creating conditions for citizens to become more autonomous and responsible in relation to their health and the health of those who depend on them, as well as a positive view of health. Enact and disseminate the charter of rights and duties of the citizen in the field of health. Develop planning, interventions, monitoring and evaluation in the area of Citizenship in Health: information and monitoring systems, development of evidence and recommendations, evaluation and identification of good practices, promotion of a research and innovation agenda. Promote the active participation of organizations representing the interests of the citizen. Ensure that healthcare_professional develop competencies that promote citizenship, at pre_graduate and post_graduate level, and monitor and evaluate the practices adopted. Promote, at the institutional level, processes of continuous improvement of the exercise of citizenship, for example regular assessments of citizen needs, interventions that promote participation and literacy. At the organizational level, institutions should: Improve citizens’ knowledge of rights and duties and promote the conditions for their exercise; Improve citizens’ trust in institutions and the conditions for their appreciation, through regular listening to the needs, expectations, satisfaction and experiences of users; Disseminate institutional information, in a transparent manner, publishing indicators of performance evolution and the results of services and healthcare_professional; Promote, monitor and evaluate the exercise of citizenship in the decision_making process, strategic development and institutional evaluation, implementing mechanisms for listening to satisfaction; Develop health education and disease self_management programs; Promote volunteering for more active citizenship. In their practice, healthcare_professional should: Increase the provision of individualized and personalized care, with patient participation in the therapeutic decision_making process; Consider and assess the socioeconomic and cultural context and adapt healthcare to the reality of the citizen, family and community. At the individual level, citizens should: proactively improve knowledge and the ability to exercise responsibilities and rights, as well as fulfill duties in health; Assume responsibility for health promotion and healthy lifestyles and actively participate in decisions regarding personal, family and community health; Establish therapeutic alliances with healthcare_professional, forming partnerships in disease management; Promote the rational and appropriate use of health services. Strategies to strengthen equity and appropriate access to healthcare: In their practice, healthcare_professional should: Develop and standardize care coordination and proactively invest in communication between providers within and between institutions and services. Intervene on determinants associated with access as a key_factor of health inequities, promoting strategies to improve access, adapting services, making responses more flexible, diversifying practices, exchanging experiences and evaluating performance. Promote citizen trust in their family doctor and nurse in a relationship that promotes proximity and continuity of personalized care, as the main managers of their health situation, and responsible for mobility between the various health services. At the individual level, citizens should: Use access mechanisms appropriately to their health needs, understanding the advantages of using quick and urgent guidance and personalized and continuous care, rather than the inappropriate use of hospital emergency.

**** *id_85 *doc_5 *year_2022 *author_2 *area_3

In a complex world, ethics and science help us understand and work for the sustainable development of the Country and the world, privileging values such as peace, prosperity, global understanding, equity, humanization and centrality in people, participation for citizenship and the transparency of our purposes and actions. In order to make more explicit the targets that Portugal will have to pursue, individually and collectively, in the implementation stage of the national_health_plan 2030, strategic objectives were also selected. Reduce inequalities: Promote equity in health; Promote peace, justice and prosperity; Energize partnerships between all sectors of society. Promote the development of healthy behaviors, cultures and communities: Promote health literacy; Energize health_promoting environments; Promote longevity and active and healthy ageing. Reduce in an integrated manner the burden of communicable and non_communicable diseases: Strengthen sustainable healthcare; Strengthen access to quality healthcare; Energize the integration of person_centered care. More than a document, the national_health_plan 2030 is a participatory, co_creative, structured and integrating process that, starting from the joint identification of the health needs of the population present in Portugal, arising from health problems and their determinants, selects the health strategies appropriate to change, particularly aiming at reducing health inequities, for a sustainable health of all for all. The national_health_plan 2030 follows a sustainable health planning model, population_based, trans and multisectoral in nature, having as a key_element people, individual or collective, participation and commitment. Participation: Ensure the involvement and commitment of all, for the creation of value and health outcomes; Equity: Intervene on avoidable, unfair or remediable health inequalities, in socioeconomic, geographic and demographic contexts or by other various dimensions of inequality; sustainability: create and preserve healthy communities, economically and socially just and environmentally adequate; transparency: promote access to quality information for the appreciation of health and for the exercise of citizenship; centrality in people: value the diversity, needs and expectations of people. The object of a health_system and of health policies will always be the citizen and also the community in which they are inserted. When emphasis is placed on the centrality of the citizen in the health_system, this means that beyond being the object of the attention of health services, they must also become the subject, the main actor, in the health_system. This intention to democratize health is not new. However, to transform this intention into reality, it is necessary to develop appropriate instruments for that purpose. Centrality of the citizen also means their involvement and participation in what concerns the protection and promotion of their health and that of their fellow citizens. Here too, beyond the goodness of the principle, it is necessary to instrument its application. Multisectoral and transdisciplinary in nature, it is transversal to all intervention strategies and identified health needs and allows intervention on all health determinants. It implies strengthening or implementing health promotion strategies assessed as most cost_effective. It also implies instituting the systematic analysis of policies and intervention strategies of different sectors, as to their impact on health. In this sense, it is essential that the nhs collaborate with other health institutions, but also with the sectors of education, economy, social security, environment and with local powers, public and private, to protect and promote health and prevent disease, pay attention to the individual, but also to the community in which they are inserted. The development of projects and of a digital transition culture, centered on the citizen and embedded in the resilience and recovery plan, are a strategic bet of which the technological platform to promote physical activity integrated in the suava, among others, is an example. The adoption and implementation of digital will contribute to effectively improving literacy and access to quality healthcare, mitigating inequalities, reducing geographic distances and strengthening citizen confidence in the health_system and, in particular, in the nhs.
