# Supplementary material for: Person-centered practice in the Portuguese healthcare system: A documentary study
Source: PLoS One. 2026 Mar 3;21(3):e0343419. doi: 10.1371/journal.pone.0343419 (PMC12956081; doi:10.1371/journal.pone.0343419)

**Factorial representation of the semantic structure of the classes**

The figure below shows a correspondence factorial analysis (CFA) of active forms (words) associated with the descending hierarchical classification (DHC) classes. Words are colored by the DHC class with which they are most strongly associated, and word size indicates their frequency in the corpus (with larger sizes indicating more frequent occurrences). The axes (Factor 1, Factor 2) summarize the main semantic contrasts, and the percentages indicate how much of the overall association pattern each dimension captures. Words that are plotted closer together tend to co-occur more frequently in the corpus.
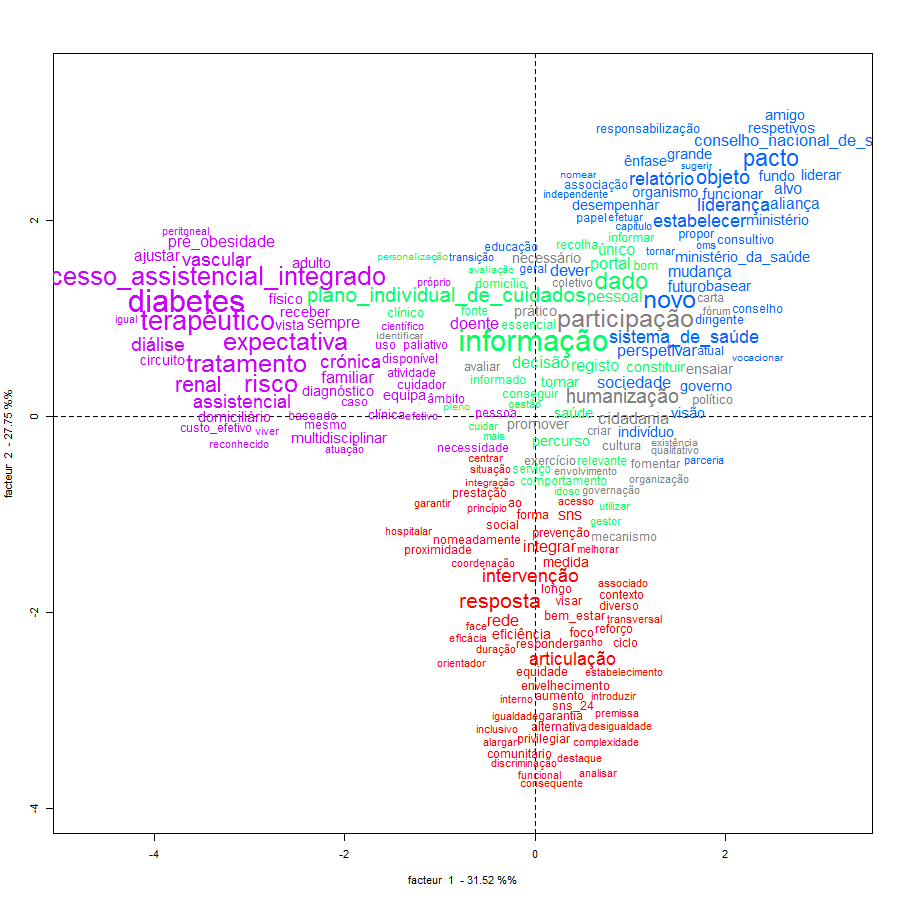

Supplement: S3 Appendix — (DOCX) [file pone.0343419.s004.docx]
