# Supplementary material for: Person-centered practice in the Portuguese healthcare system: A documentary study
Source: PLoS One. 2026 Mar 3;21(3):e0343419. doi: 10.1371/journal.pone.0343419 (PMC12956081; doi:10.1371/journal.pone.0343419)
Supplement: S4 Appendix — (DOCX) [file pone.0343419.s005.docx]

**Factorial representation of the semantic structure of the variables of interest**

The figure below shows a correspondence factorial analysis (CFA) in which the variables of interest are projected as points onto the factorial plane defined by the lexical structure. Each point represents a category of a variable, namely document type, year, author, and thematic area.

Colors group categories by the descending hierarchical classification (DHC) class with which they are most strongly associated*,* and size indicates their frequency in the corpus (with larger sizes indicating more frequent occurrences). The axes (Factor 1, Factor 2) summarize the main semantic contrasts; the percentages indicate how much of the overall association pattern each dimension captures.

Categories plotted closer together are more strongly associated with those lexical environments.

**
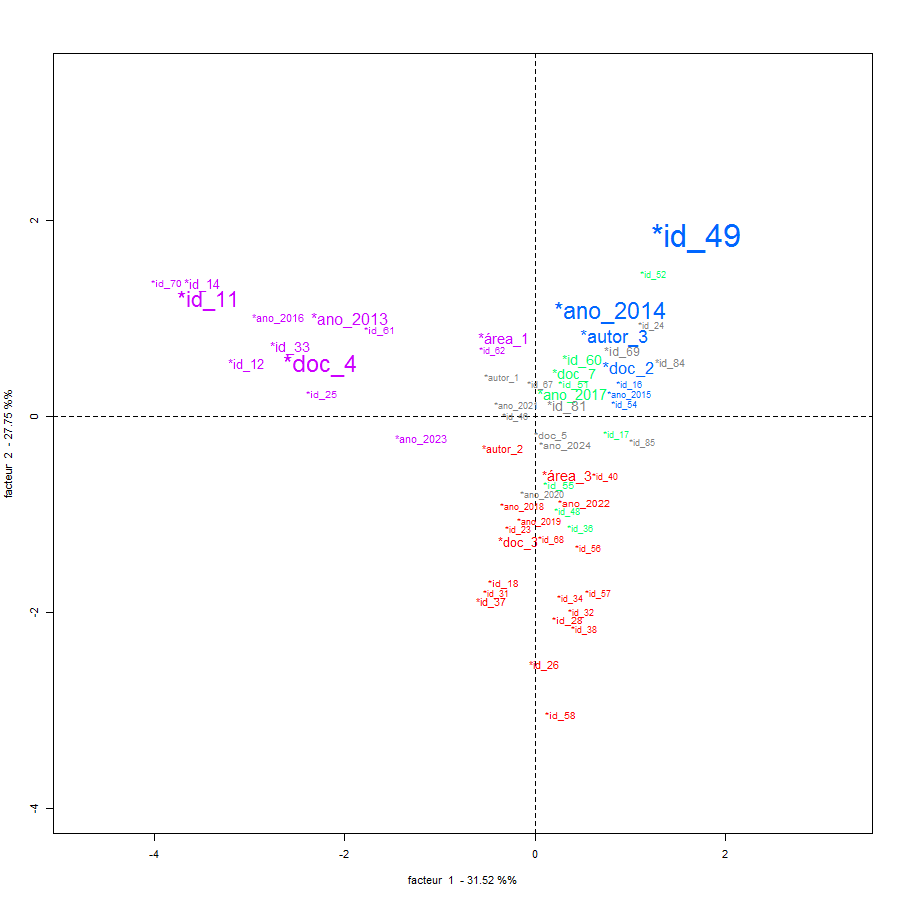
**
