# Supplementary material for: Person-centered practice in the Portuguese healthcare system: A documentary study
Source: PLoS One. 2026 Mar 3;21(3):e0343419. doi: 10.1371/journal.pone.0343419 (PMC12956081; doi:10.1371/journal.pone.0343419)
Supplement: S5 Appendix — (DOCX) [file pone.0343419.s006.docx]

**Similarity graph**


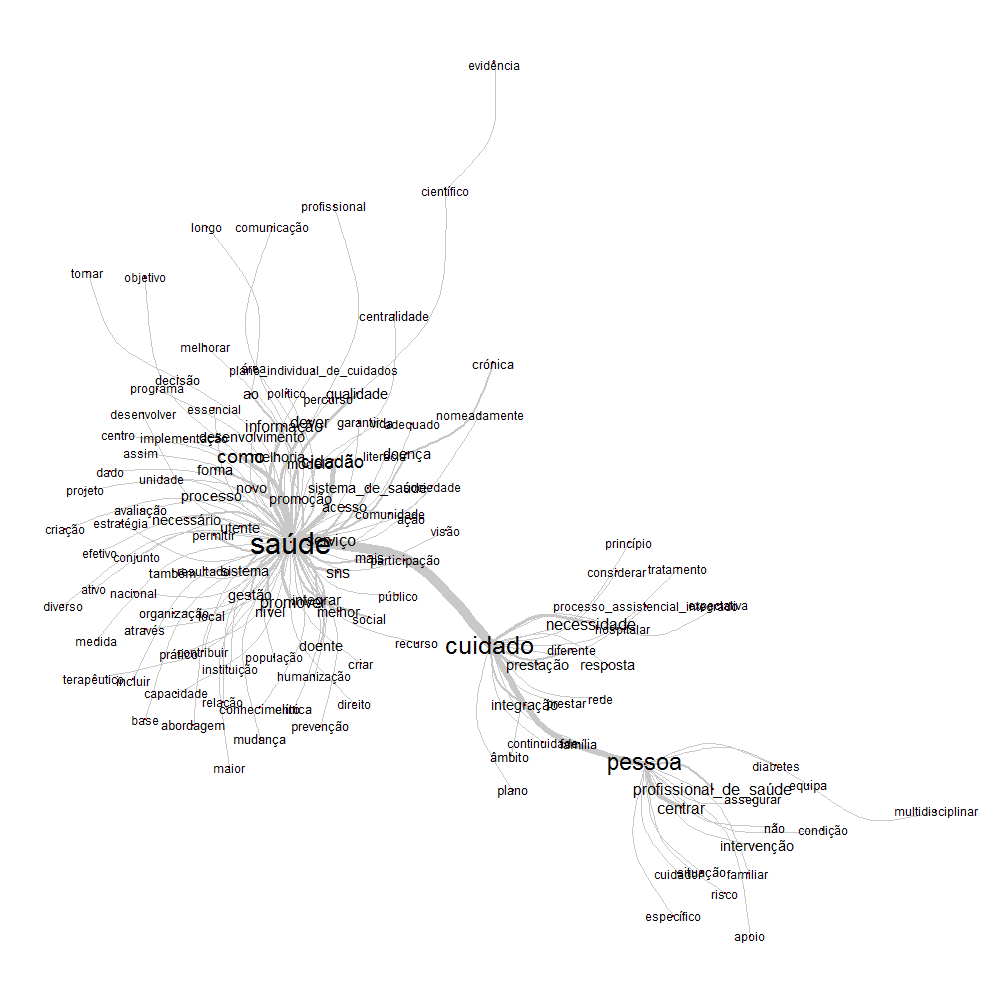
The figure presents a similarity analysis mapping word co-occurrences across text segments. Lines connect words that co-occur within the same segments; thicker lines indicate stronger co-occurrence. Word size reflects its frequency in the corpus (the larger, the more frequent). From central nuclei of highly connected terms, peripheral branches extend, indicating more specific lexical contexts. Bridge words linking branches point to terms shared across themes.
